# Synthesis and evaluation of imidazole-4,5- and pyrazine-2,3-dicarboxamides targeting dengue and yellow fever virus^☆^

**DOI:** 10.1016/j.ejmech.2014.09.062

**Published:** 2014-11-24

**Authors:** Milind Saudi, Joanna Zmurko, Suzanne Kaptein, Jef Rozenski, Johan Neyts, Arthur Van Aerschot

**Affiliations:** a Medicinal Chemistry, Rega Institute for Medical Research, KU Leuven, Minderbroedersstraat 10, 3000 Leuven, Belgium; b Laboratory of Virology and Chemotherapy, Rega Institute for Medical Research, KU Leuven, Minderbroedersstraat 10, 3000 Leuven, Belgium

**Keywords:** Flavivirus inhibitors, Dengue virus, Yellow fever virus, Imidazole dicarboxylic acid, Pyrazine dicarboxylic acid

## Abstract

The results of a high-throughput screening assay using the dengue virus-2 replicon showed that the imidazole 4,5-dicarboxamide (I45DC) derivative (**15a**) has a high dengue virus inhibitory activity. Based on **15a** as a lead compound, a novel class of both disubstituted I45DCs and the resembling pyrazine 2,3-dicarboxamides (P23DCs) were synthesized. Here, we report on their *in vitro* inhibitory activity against dengue virus (DENV) and yellow fever virus (YFV). Some of these first generation compounds have shown activity against both viruses in the micromolar range. Within this series, compound **15b** was observed to display the highest antiviral potency against YFV with an EC_50_ = 1.85 μM. In addition, compounds **20a** and **20b** both potently inhibited replication of DENV (EC_50_ = 0.93 μM) in Vero cells.

## Highlights

• Two new series of heterocycles were evaluated for Flavivirus inhibition.
• Activities at micromolar levels were noted for inhibition of dengue virus.
• Remarkable selective inhibitory properties for yellow fever virus were recorded.
• Imidazole-4,5-dicarboxylic amides provide an interesting scaffold for antivirals.
• Pyrazine-2,3-dicarboxylic amides likewise are endowed with anti-flavivirus activities.

## Graphical abstract

**Figure:**
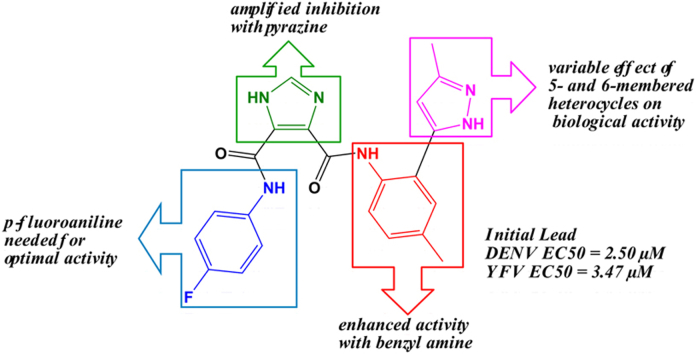

## 1 Introduction

**Fig. 1:**
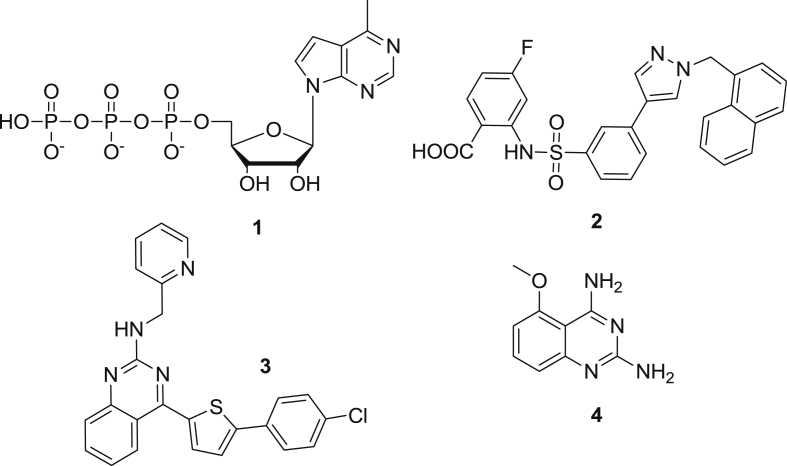
Structures of some recently described antiviral compounds being inhibitory for dengue virus.

Dengue is the most common arthropod-borne viral infection in the world and is estimated to transmit 390 million new infections every year [3]. Dengue virus (DENV) belongs to the Flavivirus genus of the Flaviviridae. The genome of DENV is comprised of a 10.7 kb, single, positive-stranded RNA with at least four different circulating serotypes (DENV-1 to DENV-4) [4]. Reportedly, a fifth serotype now complicates vaccine development [4]. With increased levels of population growth, urbanization, and international travel, DENV illness has increased 30-fold in the last 50 years [5]. Most of the current research on dengue infections is focused on the treatment of symptoms, which often can be a tedious and intensive process. As there is neither a drug nor any vaccine available to combat DENV infections till date [6], it is highly important to explore and uncover small molecules that have potent anti-dengue activity. In the past half-decade, a number of antiviral agents with such inhibitory properties have been discovered. These include an adenosine analog **1** [7], a *N*-sulfonylanthranilic acid derivate **2** [8], the chlorophenyl-thiophene derivate **3** [9], and most recently some 2,4-diaminoquinazoline derivatives **4** [10] (see Fig. 1). Unfortunately, none of these substances have entered into clinical trials. Hence, further development of new chemical entities endowed with dengue inhibitory properties is warranted. Our previous efforts herein, focused on tritylated and alkylated nucleoside analogs, resulting in compounds endowed with strong anti-flavivirus inhibitory properties. However, no clear SAR could be determined [1], [2]. Hence we now turned our attention to another lead molecule.

**Fig. 2:**
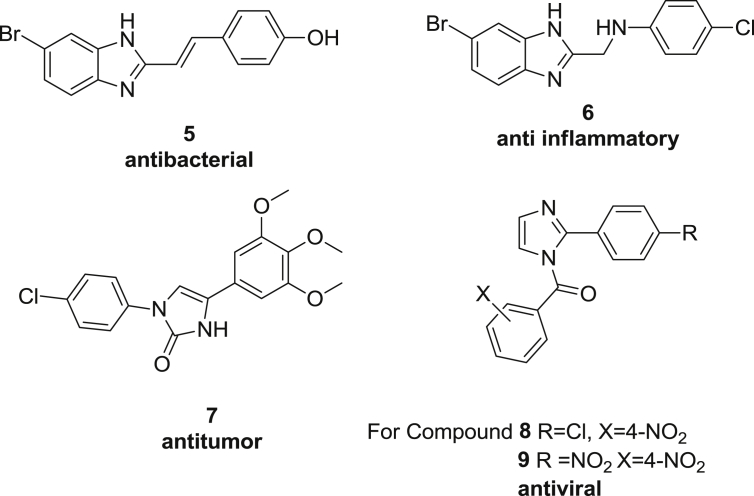
Some examples of imidazole containing structures endowed with pronounced biological activity.

The five-membered imidazole ring is a structural unit found in many biologically active compounds. The strong therapeutic properties of imidazole containing drugs have encouraged medicinal chemists to synthesize a large number of novel chemotherapeutic agents comprising this entity. Amongst others, imidazole core structures are found in different carboxypeptidase, hemeoxygenase and lactamase inhibitors, as well as among anti-inflammatory, anticancer, antibacterial, antifungal, antitubercular, antidiabetic and antiviral products, further highlighted with some examples. Ramya et al. synthesized a series of novel 5-(nitro/bromo)-styryl-2-benzimidazole derivatives (**5**) and tested them for their antibacterial activity against *Staphylococcus aureus*, *Escherichia coli* and likewise evaluated the antifungal activity against *Candida albicans* and *Aspergillus fumigates* [11]. Biological activities proved comparable to ciprofloxacin. Kavitha C.S. et al. synthesized a series of 2-methylaminobenzimidazole derivatives in which compound **6** showed analgesic and anti-inflammatory activity comparable with the standard drug nimesulide [12]. Cenzo et al. synthesized a series of 1, 4-diarylimidazole-2(3H)-one derivatives **7** and their 2-thione analogs and found antitumor activity [13]. Finally, Sharma et al. synthesized imidazole derivatives for antiviral screening and different [2-(substituted phenyl)-imidazol-1-yl]-benzamides like **8** and **9** were selected as the most promising antiviral agents [14] (see Fig. 2).

Imidazole-4,5-dicarboxylic acid (I45DC) and its derivatives such as those bearing primary or secondary amides have previously been reported to be active against HIV-1 protease [15]. Likewise, some derivatives of this scaffold were uncovered as potential kinase inhibitors with antiproliferative activity against HL-60 cells [16]. In addition, inhibition of protein–protein interaction between Hepatitis C glycoprotein E2 and CD81 has been reported previously [17]. The I45DCs are known as well to be a structural component of some antibiotics [18] and also to affect memory [19]. Substitution on the imidazole moiety herein has been reported, both in solution phase as well as in a combinatorial fashion on solid support.

**Fig. 3:**
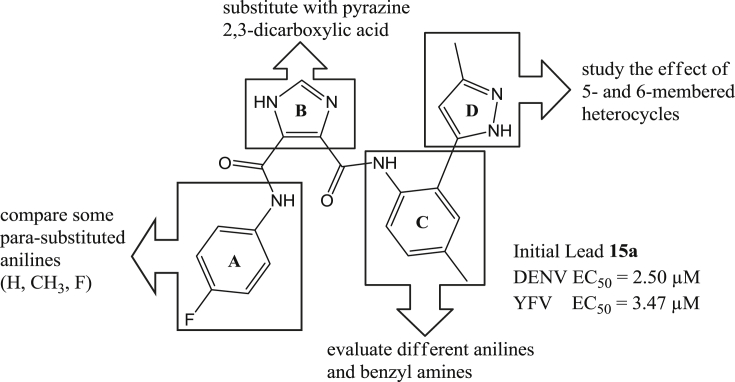
Lead compound **15a** and envisaged modifications of different parts of the antiviral lead for optimization of the structure.

High throughput screening of a compound library led to the identification of *N*^5^-(4-fluorophenyl)-*N*^4^-(4-methyl-2-(3-methyl-1H-pyrazol-5-yl)phenyl)-1H-imidazole-4,5-dicarboxamide **15a** (see Fig. 3) with an EC_50_ of 2.50 μM and 3.47 μM against DENV and YFV, respectively. In search for new compounds with potential for clinical use as antiviral agents, a series of compounds based on this I45DC scaffold were synthesized with regiospecific attachment of the substituents and these analogs were investigated for their inhibitory properties against DENV and YFV. In addition, the imidazole core was substituted for a pyrazine ring leading to a series of pyrazine-2,3-dicarboxylic acids (P23DC). The intended substitution of the central imidazole ring would result in slightly different orientation of the attached amide groups, and in a change of the hydrogen bonding pattern exchanging the combination of a hydrogen donating and a hydrogen accepting nitrogen into two hydrogen accepting nitrogen atoms. In this communication, we report on the synthesis and biological activity of both new series of dissymmetric I45DCs and P23DCs, which we studied for their inhibitory properties against YFV and DENV.

## 2 Results and discussion

### 2.1 Synthetic aspects

**Scheme 1:**
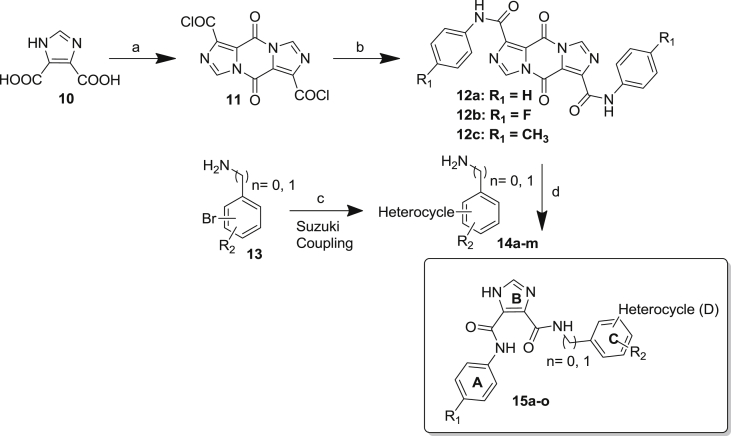
General reaction scheme for synthesis of the I45DC analogs. Conditions: a) SOCl_2_, cat. DMF, Toluene, 85 °C; b) DIPEA, substituted aniline, DCM, −10 °C; c) acetonitrile:water (1:1), K_2_CO_3_, PdCl_2_TPP_2_, boronic acid; d) DIPEA, substituted aniline or benzyl amine, DCM, room temperature (rt).

The general procedure employed for the synthesis of dissymmetrically disubstituted I45DCs is shown in Scheme 1. Imidazole-4,5-dicarboxylic acid **10** was allowed to reflux with SOCl_2_ in toluene with catalytic DMF to afford pyrazinedione diacid dichloride **11** in 92% yield [20]. The literature route for amide formation suggests hydrolysis of the acid chlorides to carboxylic acids by the addition of water. Amines are then added to open both acyl imidazole bonds affording two identical imidazole analogs. Baures et al., replaced the water with phenol, thereby modulating the reactivity of the acid chloride in comparison with an acyl imidazole bond for the subsequent addition of two different amines [20]. The difficulty in preparing such analogs is that their synthesis is highly dependent upon the substituents to be introduced. Primary benzyl amines, for example, are too reactive to provide selectivity in the reaction, whereas anilines can be added in a proper stoichiometric ratio in order to provide a pyrazine dione intermediate **12** which can often be purified by crystallization. In the case of aniline derivatives, the resulting pyrazine intermediates are often insoluble in the reaction solvent and can be isolated simply by vacuum filtration. In parallel, different bromoanilines or benzylamines were subsequently coupled with commercially available heterocyclic boronic acids through palladium catalyzed Suzuki reaction to give the desired second aniline in 60–75% yield. Addition of this second aniline to the pyrazine intermediates **12a**–**c** resulted in opening of the acyl imidazole bond to obtain the expected product. Based on this approach, a series of novel molecules was prepared within this I45DC family containing various substituted phenyl rings. For some of the final products **15**, the reaction sequence was changed with prior attachment of the aniline carrying a heterocycle.

**Scheme 2:**
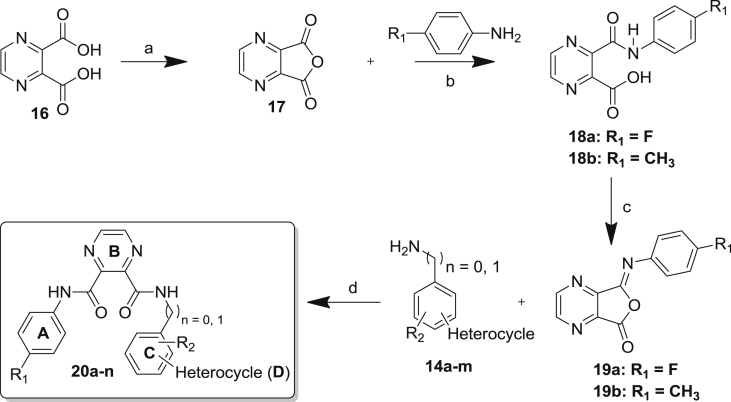
General scheme for assembly of different P23DC analogs. Conditions: a) acetic anhydride; b) acetonitrile:water (1:1), substituted aniline, dodecyl hydrogen sulfate sodium salt, rt; c) trifluoroacetic anhydride, TEA, THF; d) substituted aniline or benzyl amine, THF, rt.

Pyrazines are important pharmacophores present in a number of biologically active compounds such as antimycobacterial, antibacterial, antidiabetic, and hypnotic agents. To further explore previous structure–activity relationship, we planned to substitute the five-membered imidazole ring with a pyrazine ring. Hereto, regioselective functionalization of the pyrazine started from commercial pyrazine-2,3-dicarboxylic acid (P23DA) and a four-step synthesis of the target compounds has been described in Scheme 2. P23DA was converted to its corresponding anhydride **17** by treatment with acetic anhydride at room temperature.

Reaction with *p*-fluoroaniline or *p-*toluidine resulted in opening of the five-membered ring with concomitant formation of the first amide bond (**18a**,**b**). Reaction with trifluoroacetic anhydride at 0 °C afforded the anhydric activated product **19a** or **19b** allowing introduction of the second amide. Hereto, the intermediate was reacted with preformed anilines or benzyl amines **14a**–**m** to give the target compounds **20a**–**n** in 60–80% overall yields. All final products were purified by silica gel chromatography and characterized by ^1^H NMR, ^13^C NMR and HRMS before evaluation of their inhibitory properties against DENV and YFV.

### 2.2 Assessment of antiviral activity

As shown in Fig. 3 and Scheme 1, Scheme 2, four main structural domains were chosen for optimization of hit **15a**: the aromatic moiety (ring A), the central pyrazine/imidazole core (ring B), the substituted aromatic moiety (ring C) and the heterocyclic group (ring D). These efforts have provided a few compounds that exhibited an improved biological profile with respect to the initial lead compound **15a**. Overall, improvement or reduction of the inhibitory activities for either DENV or YFV is mostly running in parallel.

The overall structure of **15a** comprises an imidazole core carrying two aniline moieties coupled via an amide bond, with one of the aniline rings substituted with an additional heterocyclic ring (ring D). Therefore, our efforts to understand the structure–activity relationship of the initial lead started with modifying the heterocyclic moiety as shown in Table 1. Using *p*-fluoroaniline (ring A) and the I45DC core as a fixed fragment, substitution of the five-membered pyrazole moiety (ring D) with a thienyl or furanyl ring mostly led to considerable loss of activity (i.e., compare **15h**, **15i**, **15j** versus **15a**). However, activity remarkably was restored upon substitution of a methyl group for the para-fluorine in ring A (**15k**, **15l**). The benzylamine substitution for ring A likewise annihilated the inhibitory properties (**15m**), while in contrast a p-fluoro-phenylethylamine again displayed nice activity (**15n**). Exchanging aniline ring C for a benzylamine preserved the activity when the supplementary heterocycle D was attached at the ortho position (**15c**, **15d**), while para substitution led to considerable loss of activity (**15e**–**g**) within this context. A single attempt with an aminopyridine ring at site C proved inactive (**15o**). Overall, the compounds **15b** (EC_50_ = 1.85 μM, YFV), **15c** (EC_50_ = 1.93 μM, DENV), **15d** (EC_50_ = 2.61 μM, YFV) and **15k** (EC_50_ = 2.02 μM, DENV) are endowed with the best inhibitory properties within this imidazole series.

**Table 1:**
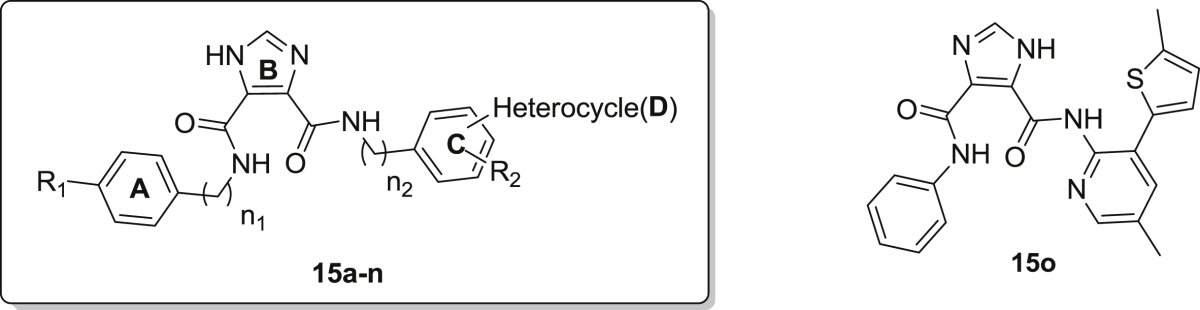
Inhibitory properties for yellow fever virus and dengue virus of the imidazole analogs **15a**–**15o**.

Sr. No.	*n*_1_	*n*_2_	R_1_	R_2_	Heterocycle (position^a^)	Dengue			Yellow fever		
						EC_50_ (μM)	CC_50_ (μM)	SI	EC_50_ (μM)	CC_50_ (μM)	SI
**15a**	0	0	F	p-Methyl	3-Methyl-1H-pyrazol-5-yl (ortho) *Initial Lead*	2.50	>120	48	3.47	>120	34.5
**15b**	0	0	F	H	3-Methyl-1H-pyrazol-5-yl (ortho)	6.03	21.7	3.6	1.85	>121	67
**15c**	0	1	F	H	Pyridin-4-yl (ortho)	1.93	50.1	26	3.90	>120	31
**15d**	0	1	F	H	Thien-3-yl (ortho)	8.88	17.32	1.95	2.61	34.5	13.2
**15e**	0	1	F	H	Pyridin-3-yl (para)	42.10	>120	2.85	11.81	>118	10
**15f**	0	1	F	H	Thien-3-yl (para)	33.69	20.21	0.60	28.77	>119	4.13
**15g**	0	1	F	H	Pyridin-4-yl (para)	>120	>120	1	>120	>120	1
**15h**	0	0	F	p-Methyl	Thien-3-yl (ortho)	48.75	>117	2.4	>119	>119	1
**15i**	0	0	F	p-Methyl	Furan-2-yl (ortho)	>123.6	>124	1	ND	–	–
**15j**	0	0	H	p-Methyl	Thien-3-yl (ortho)	>124	>124	1	9.20	>120	13
**15k**	0	0	CH_3_	p-Methyl	Thien-3-yl (ortho)	2.02	>50	24.5	>120	>120	1
**15l**	0	0	CH_3_	p-Methyl	Furan-2-yl (ortho)	3.47	>125	36.2	ND	–	–
**15m**	1	0	H	p-Methyl	Furan-2-yl (ortho)	>125	>125	1	ND	–	–
**15n**	2	0	F	p-Methyl	Furan-2-yl (ortho)	3.77	>116.5	30.9	ND	–	–
**15o**	0	0	H	p-Methyl	See structure above	>120	26.40	0.22	>120	>119	0.97

a Indicates the attachment point of the heterocycle on the phenyl ring.

With these results in mind, we hoped to further improve the potency via replacement of the central five-membered imidazole (ring B) with a pyrazine scaffold, both rings containing two nitrogen atoms but providing a slightly different orientation for the aniline substituents. Focusing on compound **15c** as most potent representative for inhibition of DENV (EC_50_ = 1.92 μM), we synthesized a series of pyrazine analogs with a pyridine substituent as the heterocyclic moiety D, attached at different positions of ring C. The inhibitory potency of these compounds was likewise examined against DENV and YFV and is summarized in Table 2 (compounds **20a**−**20n**).

**Table 2:**
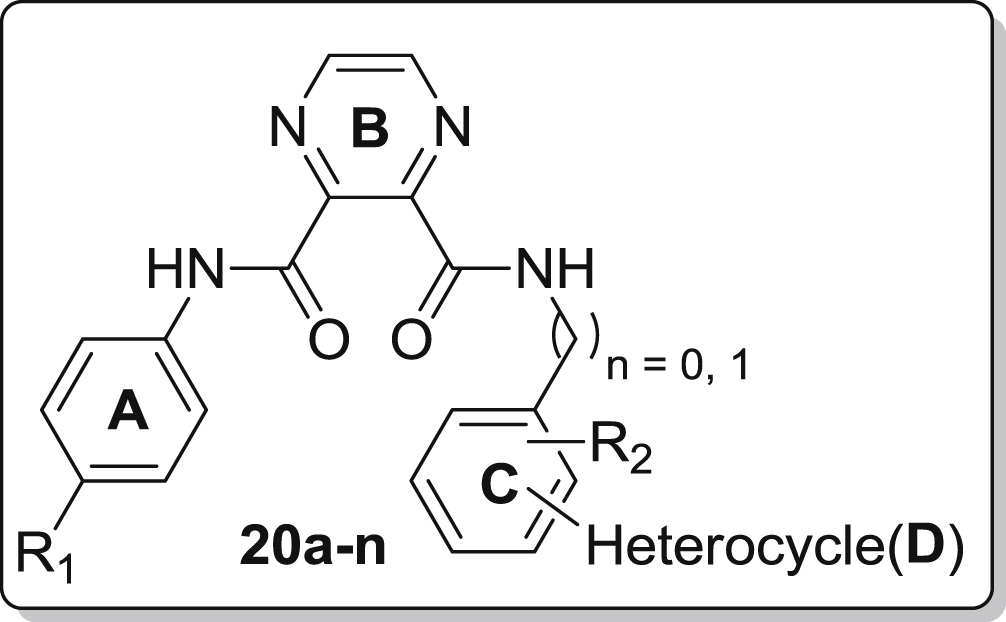
Inhibitory properties for yellow fever virus and dengue virus of the pyrazine containing compounds **20a**–**20n**.

Sr. No.	*n*	R_1_	R_2_	Heterocycle (position^a^)	Dengue			Yellow fever		
					EC_50_ (μM)	CC_50_ (μM)	SI	EC_50_ (μM)	CC_50_ (μM)	SI
**20a**	1	F	H	Pyridin-4-yl (para)	0.94	>117.5	>125	7.49	>120	16
**20b**	0	F	p-Methyl	Pyridin-3-yl (ortho)	0.94	>117.5	>125	13.10	>118	9
**20c**	1	F	H	Pyridin-3-yl (para)	2.99	>115.1	38.5	8.90	>116	13
**20d**	0	F	H	Pyridin-4-yl (ortho)	11.71	>117.1	10.4	8.22	>123.3	15
**20e**	0	F	H	Pyridin-3-yl (ortho)	23.19	>120.5	5.2	47.53	>119	2.5
**20f**	1	F	H	Pyridin-4-yl (ortho)	40.70	>118.0	2.9	21.29	>127	6
**20g**	0	F	p-Methyl	Pyridin-4-yl (ortho)	>117	>117	1	48.43	>125	2.6
**20h**	1	CH_3_	H	Pyridin-4-yl (para)	16.60	74.7	4.5	36.83	>110.5	3
**20i**	0	CH_3_	p-Methyl	Pyridin-3-yl (ortho)	10.55	47.5	4.5	18.41	75.5	4.1
**20j**	1	CH_3_	H	Pyridin-3-yl (para)	6.75	>116.1	17.2	73.08	>124	1.7
**20k**	0	CH_3_	H	Pyridin-4-yl (ortho)	4.71	>122.5	26	12.46	>125	10
**20l**	0	CH_3_	H	Pyridin-3-yl (ortho)	14.56	>128.13	8.8	>122	>122	1
**20m**	1	CH_3_	H	Pyridin-4-yl (ortho)	57.86	>118	2.04	70.61	>120	1.7
**20n**	0	CH_3_	p-Methyl	Pyridin-4-yl (ortho)	11.90	>119	10	30.93	>117.5	3.8

a Indicates the attachment point of the heterocycle on the phenyl ring.

Unfortunately, the compound **20f** corresponding to **15c**, exhibited 20-fold decrease in activity against DENV (EC_50_ = 40.70 μM) and 6-fold against YFV (EC_50_ = 21.29 μM). Concentrating on anti-DENV activity, we noticed within the imidazole series that the para attachment of the heterocycle to ring C proved deleterious for the biological activity (see **15e**–**g**). In contrast with these results, the para-pyrimidin-4-yl substituent on ring C proved advantageous within the pyrazine series and led to compound **20a** displaying a 2-fold increase in anti-DENV potency with EC_50_ of 0.93 μM. A distinction however can be made having either a benzylamine (*n* = 1) or an aniline (*n* = 0) aromatic ring C. Within the benzylamine series, the heterocycle is preferred at the para position with preference for concomitant presence of fluoroaniline at the A-site (**20a**, **20c**) over toluidine (**20h**, **20j**). However, ortho attachment of the heterocycle on the benzylamine ring seriously reduced the activity (**20f**, **20m**). Having an aniline ring at site C the results were less clear with mostly intermediate inhibitory properties (5–25 μM), except for the strongly inhibiting compound **20b** and the non-active analog **20e**. Within this pyrazine series (**20a**–**n**) the YFV inhibitory properties in general were slightly weaker compared to DENV inhibition by these compounds.

## 3 Conclusion

**Fig. 4:**
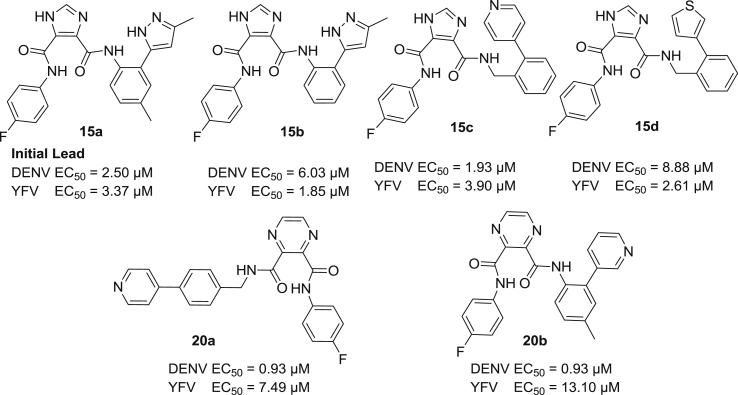
Overview of all new compounds most inhibitory to either DENV or YFV as reported in this communication.

A series of tetracyclic inhibitors were prepared using I45CD and P23DC scaffolds and which further were part of a structure−activity investigation. The SAR results of both series, I45DCs and P23DCs derivatives, as potent anti-dengue and anti-yellow fever virus agents are described above. Our investigation led to the discovery of **15b**, which is the most potent inhibitor of the series (EC_50_ = 1.85 μM) against YFV, but followed closely in potency by the quite deviating structure **15d**. The structural analog **15c** on the other hand proved most inhibitory for dengue virus (see Fig. 4). In addition, within the pyrazine series both compounds **20a** and **20b** were found to have potent DENV inhibitory properties (EC_50_ = 0.93 μM) uncovered to date. Key features of these inhibitors include the central bis-amide arrangement which is found to be a metal-chelating component in many drugs. In summary, determination of the antiviral activities shows that I45DC and P23DC are attractive candidate scaffolds for further studies and the interesting activities observed so far, warrant an in depth study of their mechanism of action.

## 4 Materials and methods

All other chemicals were provided by Aldrich or ACROS and were of the highest quality. ^1^H and ^13^C NMR spectra were determined with a 300 MHz Varian Gemini apparatus with tetramethylsilane as internal standard for the ^1^H NMR spectra (s = singlet, d = doublet, dd = double doublet, t = triplet, br. s = broad signal, br. d = broad doublet, m = multiplet) and the solvent signal – DMSO-*d*6 (*δ* = 39.6 ppm) or CDCl3 (*δ* = 76.9 ppm) – for the ^13^C NMR spectra. Exact mass measurements were performed with a quadrupole/orthogonal acceleration time-of-flight tandem mass spectrometer (qTOF2, Micromass, Manchester, UK) fitted with a standard electrospray ionization (ESI) interface. All solvents were carefully dried or bought as such.

### 4.1 Chemistry

#### 4.1.1 5,10-Dioxo-5,10-dihydrodiimidazo[1,5-a:1′,5′-d]pyrazine-1,6-dicarbonyl dichloride (**11**)

To a dry round-bottom flask was added 1.0 g of imidazole-4,5-dicarboxylic acid (**10**, 1 mmol) and 10 mL of toluene. To this stirred suspension were added 3.0 mL of thionyl chloride (6.5 mmol) and 0.250 mL of DMF (catalytic). The resulting mixture was refluxed for 16 h. After the mixture was cooled in ice bath, the solid product was collected by vacuum filtration, washed with two 20 mL portions of toluene, and dried under vacuum to yield 0.95 g of crude **11** (Yield: 95%) as a yellow solid. This product was used further without characterization.

#### 4.1.2 General procedure for **12a**–**e**

To a suspension of **11** (1.0 mmol) in 10 mL of dichloromethane was added *N*,*N*-diethylaniline (2 mmol), and corresponding substituted aniline (1.7 mmol) at −10 °C. The reaction mixture was stirred at room temperature for 3–5 h before collecting highly insoluble colored solid **12a**–**e** by vacuum filtration. The product was washed with portions of 10 mL DCM, cold water, and acetone, respectively. *Note*: Addition of excess of aniline results in opening of acyl imidazole bonds affording unwanted products.

##### 4.1.2.1 5,10-Dioxo-*N*^1^,*N*^6^-diphenyl-5,10-dihydrodiimidazo[1,5-*a*:1′,5′-*d*]pyrazine-1,6-dicarboxamide (**12a**)

Yield: 85%; Orange solid; ^1^H NMR (300 MHz, CDCl_3_): *δ* 10.74 (s, 2H), 9.09 (s, 2H), 7.80 (d, 4H, *J* = 7.8 Hz), 7.41 (t, 4H, *J* = 8.1 Hz), 7.17 (t, 2H, *J* = 7.5 Hz). ^13^C NMR (75 MHz, CDCl_3_): *δ* 162.3, 158.1, 149.2, 145.2, 138.7, 138.2, 129.0, 124.5, 120.1. HRMS calcd for C_22_H_15_N_6_O_4_ [M+H]^+^: 427.1149; found: 427.1151.

##### 4.1.2.2 *N*^1^,*N*^6^-bis(4-fluorophenyl)-5,10-dioxo-5,10-dihydrodiimidazo[1,5-*a*:1′,5′-*d*]pyrazine-1,6-dicarboxamide (**12b**)

Yield: 87%; Yellow Solid; ^1^H NMR (300 MHz, CDCl_3_): *δ* 10.76 (s, 2H), 9.09 (s, 2H), 7.85–7.81 (m, 4H), 7.25 (m, 4H, *J* = 9.0 Hz). ^13^C NMR (75 MHz, CDCl_3_): *δ* 160.3, 158.1, 157.2, 149.0, 145.0, 138.6, 134.8, 134.8, 122.1, 122.0, 120.7, 115.5. HRMS calcd for C_22_H_15_N_6_O_4_ [M+H]^+^: 427.1149; found: 427.1151. HRMS calcd for C_22_H_13_F_2_N_6_O_4_ [M+H]^+^: 463.0961; found: 463.0961.

##### 4.1.2.3 5,10-Dioxo-*N*^1^,*N*^6^-di-p-tolyl-5,10-dihydrodiimidazo[1,5-*a*:1′,5′-*d*]pyrazine-1,6-dicarboxamide (**12c**)

Yield: 89%; Red Solid; ^1^H NMR (600 MHz, DMSO): *δ* 10.62 (s, 2H), 9.07 (s, 2H), 7.67 (d, 4H, *J* = 8.4 Hz), 7.20 (d, 4H, *J* = 7.8 Hz), 2.30 (s, 6H). ^13^C NMR (125 MHz, DMSO): δ157.8, 149.2, 145.2, 138.6, 135.9, 133.5, 129.4, 120.4, 120.0, 20.6. HRMS calcd for C_22_H_15_N_6_O_4_ [M+H]^+^: 427.1149; found: 427.1151. HRMS calcd for C_24_H_19_N_6_O_4_ [M+H]^+^: 455.1462; found: 455.1461.

##### 4.1.2.4 *N*^1^,*N*^6^-bis(4-methyl-2-(thiophen-2-yl)phenyl)-5,10-dioxo-5,10-dihydrodiimidazo [1,5-a:1′,5′-d]pyrazine-1,6-dicarboxamide (**12d)**

Yield: 74%; Red Solid; ^1^H NMR (300 MHz, DMSO): 10.01 (s, 2H), 8.97 (s, 2H), 7.79–7.63 (m, 6H), 7.35–7.23 (m, 6H), 2.37 (s, 6H, 2CH_3_). HRMS calcd for C_32_H_23_N_6_O_4_S_2_ [M+H]^+^: 619.1237; found: 619.1238. In view of insufficient solubility ^13^C NMR could not be determined.

##### 4.1.2.5 *N*^1^,*N*^6^-bis(2-(furan-2-yl)-4-methylphenyl)-5,10-dioxo-5,10-dihydrodiimidazo[1,5-a:1′,5′-d]pyrazine-1,6-dicarboxamide (**12e**)

Yield: 72%; Yellow Solid; ^1^H NMR (300 MHz, DMSO): 10.46 (s, 2H), 9.07 (s, 2H), 7.81–7.61 (m, 6H), 7.25–6.63 (m, 6H), 2.38 (s, 6H, 2CH_3_). HRMS calcd for C_32_H_23_N_6_O_6_ [M+H]^+^: 587.1673; found: 587.1672. In view of insufficient solubility ^13^C NMR could not be determined.

#### 4.1.3 General procedure for **14a**–**m**

To a solution of corresponding aniline/benzyl amine (1.0 mmol) in 10 mL dioxane:water (1:1), was added anhydrous K_2_CO_3_ (1.5 mmol), aryl boronic acids (1.2 mmol) and Pd(TPP)_2_Cl_2_ (0.025 mmol) in a seal tube. The mixture was purged with argon for 30 min at rt and heated at 100 °C for 1 h in microwave. All reactions and manipulations were run under argon atmosphere. After completion, the solvent was evaporated under reduced pressure and the residue was purified by column chromatography on a silica gel to give the desired product.

##### 4.1.3.1 2-(5-Methyl-1H-pyrazol-3-yl)aniline (**14a**) [21]

Yield: 52%; White solid; ^1^H NMR (300 MHz, CDCl_3_): *δ* 7.50 (d, 1H, *J* = 7.2 Hz), 7.13 (t, 1H, *J* = 7.5 Hz), 6.79–6.75 (m, 2H), 6.39 (s, 1H), 2.37 (s, 3H, CH_3_).

##### 4.1.3.2 4-Methyl-2-(pyridin-3-yl) aniline (**14b**)

Yield: 62%; Yellow Oil; ^1^H NMR (300 MHz, CDCl_3_): *δ* 8.70 (s, 1H), 8.61–8.59 (m, 1H), 7.83–7.80 (m, 1H),7.40–7.35 (m, 1H), 7.05–7.01 (m, 1H), 6.94 (m, 1H), 6.73–6.71 (d, 1H, *J* = 8.1 Hz), 3.60 (bs, 2H, NH_2_), 2.30 (s, 3H, CH_3_). ^13^C NMR (75 MHz, CDCl_3_): *δ* 149.2, 147.4, 140.3, 135.7, 130.0, 129.0, 127.3, 122.6, 115.2, 19.5. HRMS calcd for C_12_H_13_N_2_ [M+H]^+^: 185.1073; found: 185.1078.

##### 4.1.3.3 4-Methyl-2-(pyridin-4-yl)aniline (**14c**)

Yield: 59%; White solid; ^1^H NMR (300 MHz, CDCl_3_): *δ* 8.69–8.67 (m, 2H), 7.44–7.42 (m, 2H), 7.06–6.96 (m, 2H), 6.72 (d, 1H, *J* = 8.1 Hz), 3.69 (bs, 2H, NH), 2.30 (s, 3H, CH_3_). ^13^C NMR (75 MHz, CDCl_3_): *δ* 150.4, 130.6, 130.5, 124.0, 116.4, 20.5. HRMS calcd for C_12_H_13_N_2_ [M+H]^+^: 185.1073; found: 185.1074.

##### 4.1.3.4 2-(Pyridin-3-yl)aniline (**14d**)

Yield: 72%; Yellow solid; ^1^H NMR (300 MHz, CDCl_3_): *δ* 8.73 (s, 1H), 8.62–8.60 (m, 1H), 7.84–7.80 (m, 1H), 7.41–7.36 (m, 1H), 7.24–7.11 (m, 2H), 6.89–6.79 (m, 2H), 3.75 (bs, 2H, NH_2_). ^13^C NMR (75 MHz, CDCl_3_): *δ* 150.2, 148.6, 143.9, 136.6, 135.4, 130.7, 129.5, 123.6, 119.0, 116.0. HRMS calcd for C_11_H_11_N_2_ [M+H]^+^: 171.0917; found: 171.0921.

##### 4.1.3.5 2-(Pyridin-4-yl)aniline (**14e**)

Yield: 69%; White solid; ^1^H NMR (300 MHz, CDCl_3_): *δ* 8.69–8.67 (m, 2H), 7.44–7.28 (m, 2H), 7.25–7.13 (m, 2H), 6.89–6.78 (m, 2H), 3.82 (bs, 2H, NH_2_). ^13^C NMR (75 MHz, CDCl_3_): *δ* 149.4, 129.2, 128.8, 123.0, 118.0, 115.2. HRMS calcd for C_11_H_11_N_2_ [M+H]^+^: 171.0917; found: 171.0925.

##### 4.1.3.6 (4-(Pyridin-3-yl)phenyl)methanamine (**14f**)

Yield: 55%; White solid; ^1^H NMR (300 MHz, CDCl_3_): *δ* 8.86 (m, 2H), 8.61–8.59 (m, 1H), 7.91–7.87 (m, 1H), 7.60–7.57 (m, 2H), 7.47–7.40 (m, 2H), 7.38–7.36 (m, 1H), 3.96 (s, 2H, CH_2_), 1.62 (bs, 2H, NH_2_). ^13^C NMR (75 MHz, CDCl_3_): *δ* 149.9, 127.5, 126.8, 121.1, 45.7. HRMS calcd for C_12_H_13_N_2_ [M+H]^+^: 185.1073; found: 185.1072.

##### 4.1.3.7 (4-(Pyridin-4-yl)phenyl)methanamine (**14g**) [22]

Yield: 59%; White solid; ^1^H NMR (300 MHz, CDCl_3_): *δ* 8.64–8.62 (m, 2H), 7.61–7.41 (m, 6H), 3.92 (s, 2H, CH_2_), 1.77 (bs, 2H, NH_2_). ^13^C NMR (75 MHz, CDCl_3_): *δ* 149.2, 126.9, 126.2, 120.5, 45.1.

##### 4.1.3.8 (2-(Pyridin-4-yl)phenyl)methanamine (**14h**) [23]

Yield: 65%; White solid; ^1^H NMR (300 MHz, CDCl_3_): *δ* 8.76–8.74 (m, 1H), 7.56–7.10 (m, 8H), 3.92 (s, 2H, CH_2_). ^13^C NMR (75 MHz, CDCl_3_): *δ* 149.1, 132.0, 131.3, 131.1, 131.1, 128.2, 127.7, 127.6, 127.5, 126.9, 122.7, 120.5, 46.0.

##### 4.1.3.9 4-Methyl-2-(thiophen-3-yl)aniline (**14i**)

Yield: 62%; Off-white solid; ^1^H NMR (300 MHz, CDCl_3_): *δ* 7.47–7.40 (m, 2H), 7.33–7.31 (m, 1H), 7.09 (s, 1H), 7.03–7.00 (m, 1H), 6.75–6.72 (d, 1H, *J* = 8.1 Hz), 3.73 (bs, 2H, NH_2_), 2.33 (s, 3H, CH_3_). ^13^C NMR (75 MHz, CDCl_3_): *δ* 141.5, 140.1, 130.7, 129.1, 128.5, 127.8, 126.0, 122.6, 122.5, 116.0, 20.5. HRMS calcd for C_11_H_12_N_1_S_1_ [M+H]^+^: 190.0685; found: 190.0686.

##### 4.1.3.10 (4-(Thiophen-3-yl)phenyl)methanamine (**14j**) [24]

Yield: 58%; Off-white solid; ^1^H NMR (300 MHz, CDCl_3_): *δ* 7.61–7.58 (m, 2H), 7.47–7.28 (m, 6H), 3.92 (s, 2H, CH_2_). ^13^C NMR (75 MHz, CDCl_3_): *δ* 127.7, 126.8, 126.5, 126.3, 120.2, 46.3.

##### 4.1.3.11 2-(Thiophen-3-yl)phenyl)methanamine (**14k**) [25]

Yield: 52%; White solid; ^1^H NMR (300 MHz, CDCl_3_): *δ* 7.58–7.12 (m, 6H), 3.91 (s, 2H, CH_2_). ^13^C NMR (150 MHz, CDCl_3_): *δ* 162.1, 160.8, 160.5, 142.0, 141.1, 135.8, 132.7, 130.1, 129.8, 129.3, 129.0, 128.8, 128.6, 128.4, 128.2, 127.7, 127.5, 127.4, 127.1, 127.0, 126.8, 125.5, 125.0, 123.4, 122.7, 46.8, 44.2.

##### 4.1.3.12 2-(Furan-2-yl)-4-methylaniline (**14l**)

Yield: 63%; White solid; ^1^H NMR (300 MHz, CDCl_3_): *δ* 7.52 (s, 1H), 7.32 (s, 1H), 6.97–6.94 (m, 1H), 6.71–6.52 (m, 3H), 2.30 (s, 3H, CH_3_). ^13^C NMR (75 MHz, CDCl_3_): *δ* 152.5, 141.2, 140.8, 129.5, 127.8, 127.6, 116.9, 116.2, 111.3, 106.3, 20.4. HRMS calcd for C_11_H_12_N_1_O_1_ [M+H]^+^: 174.0913; found: 174.0910.

##### 4.1.3.13 5-Methyl-3-(5-methylthiophen-2-yl)pyridin-2-amine (**14m**)

Yield: 57%; White solid; ^1^H NMR (300 MHz, CDCl_3_): *δ* 7.84 (s, 1H), 7.28 (s, 1H), 6.99 (d, *J* = 3.3 Hz, 1H), 6.73–6.72 (m, 1H), 4.96 (bs, 2H, NH_2_), 2.49 (s, 3H, CH_3_), 2.18 (s, 3H, CH_3_). ^13^C NMR (75 MHz, CDCl_3_): *δ* 153.6, 146.5, 139.8, 138.5, 137.1, 125.6, 125.5, 122.7, 114.5, 17.0, 14.9.

#### 4.1.4 General procedure for **15b**–**o**

To a stirred solution of **12a**–**e** (1.0 mmol) in 10 mL DCM, was added DIPEA (3.0 mmol), and **14a**–**m** (3.0 mmol) under argon atmosphere at room temperature. The mixture was allowed to reflux for 16–48 h. Two alternative workup procedures were followed depending on whether the product precipitated out of solution or not.

*Workup 1 (for soluble products)*: The DCM was evaporated using a rotavapor. The product was purified by flash chromatography on silica gel.

*Workup 2 (for insoluble products)*: The precipitate was collected by filtration using a Buchner funnel and washed on the frit with DCM (20–25 mL) until the yellow impurities had disappeared. The product obtained from this workup procedure did not need chromatographic purification.

##### 4.1.4.1 *N*^5^-(4-fluorophenyl)-*N*^4^-(2-(3-methyl-1H-pyrazol-5-yl)phenyl)-1H-imidazole-4,5-dicarboxamide (**15b**)

Yield: 86%; ^1^H NMR (500 MHz, DMSO): *δ* 13.69 (bs, 1H, NH), 13.30 (bs, 1H, NH), 13.05 (bs, 1H, NH), 12.78 (bs, 1H, NH), 8.65 (d, 1H, *J* = 8.0 Hz), 7.96 (s, 1H), 7.78–7.23 (m, 9H), 6.54 (s, 1H), 2.31 (s, 3H, CH_3_). ^13^C NMR (125 MHz, DMSO): *δ* 163.3, 159.6, 158.0, 156.4, 150.3, 140.2, 137.3, 135.2, 135.0, 133.9, 130.0, 128.7, 124.8, 123.2, 121.9, 121.6, 116.4, 116.2, 103.1, 10.8. HRMS calcd for C_21_H_16_F_1_N_6_O_2_ [M−H]^+^: 403.1324; found: 403.1323.

##### 4.1.4.2 *N*^5^-(4-fluorophenyl)-*N*^4^-(2-(pyridin-4-yl)benzyl)-1H-imidazole-4,5-dicarboxami-de (**15c**)

Yield: 83%; ^1^H NMR (600 MHz, DMSO): *δ* 8.76 (d, 0.5H, *J* = 6.6 Hz), 7.96–7.17 (m, 10H), 4.57 (d, 2H, *J* = 6.0 Hz, CH_2_). ^13^C NMR (150 MHz, DMSO): *δ* 159.5, 157.9, 150.0, 137.4, 127.1, 134.8, 132.7, 132.4, 131.8, 131.7, 129.3, 129.1, 129.0, 128.7, 128.1, 122.4, 122.0, 116.0, 115.8, 43.1. HRMS calcd for C_23_H_18_F_1_N_5_O_2_ [M−H]^+^: 414.1372; found: 414.1372.

##### 4.1.4.3 *N*^5^-(4-fluorophenyl)-*N*^4^-(2-(thiophen-3-yl)benzyl)-1H-imidazole-4,5-dicarbox amide (**15d**)

Yield: 86%; ^1^H NMR (600 MHz, DMSO): *δ* 13.53 (bs, 0.5H, NH), 13.45 (bs, 0.5H, NH), 9.40 (bs, 0.5H, NH), 9.23 (bs, 0.5H, NH), 7.99–7.18 (m, 12H), 4.58 (d, 2H, *J* = 6.6 Hz, CH_2_). ^13^C NMR (150 MHz, DMSO): *δ* 164.3, 156.1, 140.5, 137.3, 137.0, 132.7, 132.5, 129.9, 129.0, 128.0, 127.6, 127.1, 126.3, 123.7, 121.1, 115.9, 115.7, 42.9. HRMS calcd for C_22_H_16_F_1_N_4_O_2_S_1_ [M−H]^+^: 419.0973; found: 419.0967.

##### 4.1.4.4 *N*^5^-(4-fluorophenyl)-*N*^4^-(4-(pyridin-3-yl)benzyl)-1H-imidazole-4,5-dicarboxamide (**15e**)

Yield: 76%; ^1^H NMR (600 MHz, DMSO): *δ* 13.55 (s, 1H, NH), 13.49 (bs, 1H, NH), 10.49 (s, 1H), 10.06 (s, 1H), 9.47 (s, 1H), 8.85–7.20 (m, 9H), 4.59 (d, 2H, *J* = 6.6 Hz, CH_2_). ^13^C NMR (150 MHz, DMSO): *δ* 164.2, 156.4, 148.6, 147.7, 139.3, 137.2, 136.0, 135.6, 135.1, 134.4, 133.0, 128.9, 128.4, 128.3, 127.3, 127.2, 124.2, 121.4, 121.4, 116.1, 116.9, 42.3. HRMS calcd for C_23_H_17_F_1_N_5_O_2_ [M−H]^+^: 414.1372; found: 414.1372.

##### 4.1.4.5 *N*^5^-(4-fluorophenyl)-*N*^4^-(4-(thiophen-3-yl)benzyl)-1H-imidazole-4,5-dicarboxamide (**15f**)

Yield: 78%; ^1^H NMR (300 MHz, DMSO): *δ* 9.84 (bs, 1H, NH), 7.97 (s, 1H), 7.87–7.19 (m, 15H), 4.57 (d, 2H, *J* = 6.3 Hz, CH_2_). ^13^C NMR (75 MHz, DMSO): *δ* 160.1, 157.0, 141.4, 141.4, 138.0, 137.0, 134.9, 134.0, 128.1, 128.0, 127.6, 127.1, 126.3, 126.0, 121.7, 121.6, 120.9, 120.7, 115.9, 115.6, 42.2. HRMS calcd for C_22_H_16_F_1_N_5_O_2_ S_1_ [M−H]^+^: 419.0983; found: 419.0993.

##### 4.1.4.6 *N*^5^-(4-fluorophenyl)-*N*^4^-(4-(pyridin-4-yl)benzyl)-1H-imidazole-4,5-dicarboxamide (**15g**)

Yield: 81%; ^1^H NMR (600 MHz, DMSO) *δ* 13.56 (s, 1H, NH), 13.47 (bs, 1H, NH), 9.50 (s, 1H, NH), 8.61–7.20 (m, 13H), 4.59 (d, 2H, *J* = 6.0 Hz, CH_2_). ^13^C NMR (150 MHz, DMSO): *δ* 164.2, 156.2, 150.5, 150.3, 146.9, 140.4, 137.1, 135.9, 135.0, 133.0, 128.8, 128.3, 128.2, 127.1, 127.0, 121.3, 121.2, 116.0, 115.9, 42.2. HRMS calcd for C_23_H_17_F_1_N_5_O_2_ [M−H]^+^: 414.1372; found: 414.1369.

##### 4.1.4.7 *N*^5^-(4-fluorophenyl)-*N*^4^-(4-methyl-2-(thiophen-3-yl)phenyl)-1H-imidazole-4,5-dicarboxamide (**15h**)

Yield: 89%; ^1^H NMR (500 MHz, DMSO): *δ* 13.64 (s, 0.7H, NH), 13.56 (s, 0.3H, NH), 13.22 (s, 0.7H, NH), 12.44 (s, 0.3H, NH), 10.46 (s, 0.3H, NH), 10.12 (s, 0.7H, NH), 7.99–7.15 (m, 13H), 2.36 (s, 3H, CH_3_). ^13^C NMR (125 MHz, DMSO): *δ* 162.5, 156.0, 138.3, 137.1, 136.7, 135.2, 134.9, 132.9, 131.5, 130.7, 130.6, 130.0, 129.2, 128.6, 128.6, 128.4, 128.4, 127.0, 126.2, 124.9, 124.2, 124.0, 122.9, 199.9, 121.2, 121.2, 116.0, 115.8, 115.2, 115.1, 20.6. HRMS calcd for C_22_H_18_F_1_N_5_O_2_ S_1_ [M−H]^+^: 421.1128; found: 421.1117.

##### 4.1.4.8 *N*^5^-(4-fluorophenyl)-*N*^4^-(2-(furan-2-yl)-4-methylphenyl)-1H-imidazole-4,5-dicarboxamide (**15i**)

Yield: 77%; ^1^H NMR (300 MHz, DMSO): *δ* 13.69 (bs, 1H, NH), 13.20 (bs, 1H, NH), 10.60 (s, 1H, NH), 8.05–7.55 (m, 6H), 7.27–6.56 (m, 5H), 2.38 (s, 3H, CH_3_). HRMS calcd for C_22_H_18_F_1_N_4_O_3_ [M+H]^+^: 405.1357; found: 405.1353.

##### 4.1.4.9 *N*^4^-(4-methyl-2-(thiophen-3-yl)phenyl)-*N*^5^-phenyl-1H-imidazole-4,5-dicarboxamide (**15j**)

Yield: 81%; ^1^H NMR (600 MHz, DMSO): *δ* 13.63 (s, 0.7H, NH), 13.56 (s, 0.3H, NH), 13.18 (s, 0.7H, NH), 12.47 (s, 0.3H, NH), 10.34 (s, 0.3H, NH), 10.12 (s, 0.7H, NH), 7.96–7.12 (m, 12H), 2.36 (s, 3H, CH_3_). ^13^C NMR (125 MHz, DMSO): *δ* 162.4, 156.0, 138.5, 138.3, 137.1, 135.2, 132.8, 131.5, 130.6, 129.9, 129.3, 128.6, 128.4, 127.0, 124.2, 124.0, 120.9, 119.4, 20.6. HRMS calcd for C_22_H_17_N_4_O_2_ S_1_ [M−H]^+^: 401.1078; found: 401.1078.

##### 4.1.4.10 *N*^4^-(4-methyl-2-(thiophen-3-yl)phenyl)-*N*^5^-(p-tolyl)-1H-imidazole-4,5-dicarboxamide (**15k**)

Yield: 79%; ^1^H NMR (600 MHz, DMSO): *δ* 13.59 (s, 0.7H, NH), 13.54 (s, 0.3H, NH), 13.09 (s, 0.3H, NH), 12.52 (s, 0.7H, NH), 10.26 (s, 0.3H, NH), 10.10 (s, 0.7H, NH), 7.98–7.18 (m, 10H), 2.36 (s, 3H, CH_3_), 2.28 (s, 3H, CH_3_). ^13^C NMR (125 MHz, DMSO): *δ* 162.4, 155.8, 138.3, 137.0, 136.0, 135.1, 133.1, 132.7, 131.5, 130.6, 129.9, 129.6, 129.4, 129.0, 128.6, 128.5, 128.4, 128.4, 127.0, 124.2, 124.0, 120.9, 119.4, 20.6. HRMS calcd for C_23_H_21_N_4_O_2_S_1_ [M+H]^+^: 417.1380; found: 417.1377.

##### 4.1.4.11 *N*^4^-(2-(furan-2-yl)-4-methylphenyl)-*N*^5^-(p-tolyl)-1H-imidazole-4,5-dicarboxamide (**15l**)

Yield: 76%; 13.58 (s, 0.7H, NH), 13.52 (s, 0.3H, NH), 13.07 (s, 0.3H, NH), 12.50 (s, 0.7H, NH), 10.23 (s, 0.3H, NH), 10.08 (s, 0.7H, NH), 7.97–7.93 (m, 2H), 7.71–7.56 (m, 4H), 7.33–7.11 (m, 5H), 2.35 (s, 3H, CH_3_), 2.28 (s, 3H, CH_3_). ^13^C NMR (125 MHz, DMSO): *δ* 162.4, 155.8, 138.3, 136.9, 136.0, 135.1, 133.1, 132.7, 131.5, 130.6, 129.8, 129.6, 129.4, 129.0, 128.6, 128.5, 128.4, 128.4, 127.0, 124.2, 124.0, 120.9, 119.4, 20.6, 20.6. HRMS calcd for C_23_H_21_N_4_O_3_ [M+H]^+^: 401.1608; found: 401.1602.

##### 4.1.4.12 *N*^5^-benzyl-*N*^4^-(2-(furan-2-yl)-4-methylphenyl)-1H-imidazole-4,5-dicarboxamide (**15m**)

Yield: 83%; ^1^H NMR (300 MHz, CDCl_3_): *δ* 11.84 (bs, 1H, NH), 11.57 (bs, 1H, NH), 10.32 (bs, 1H, NH), 8.16 (d, 1H, *J* = 8.4 Hz), 7.61–7.18 (m, 10H), 6.74 (d, 1H, *J* = 3.3 Hz), 6.57–6.55 (m, 1H), 4.70 (d, 2H, *J* = 6.0 Hz, CH_2_), 2.40 (s, 3H, CH_3_). ^13^C NMR (75 MHz, CDCl_3_): *δ* 161.7, 159.0, 142.4, 134.9, 134.8, 130.5, 129.1, 128.6, 127.9, 127.4, 127.2, 123.0, 111.7, 108.5, 43.5, 21.0. HRMS calcd for C_23_H_21_N_4_O_3_ [M+H]^+^: 401.1608; found: 401.1590.

##### 4.1.4.13 *N*^5^-(4-fluorophenethyl)-*N*^4^-(2-(furan-2-yl)-4-methylphenyl)-1H-imidazole-4,5-dicarboxamide (**15n**)

Yield: 75%; ^1^H NMR (300 MHz, CDCl_3_): *δ* 11.13 (bs, 1H, NH), 10.32 (bs, 1H, NH), 8.18 (d, 1H, *J* = 8.7 Hz), 7.67–6.55 (m, 10H), 3.73 (m, 2H, CH_2_), 3.00 (t, 2H, *J* = 7.5 Hz CH_2_), 2.42 (s, 3H, CH_3_). ^13^C NMR (150 MHz, DMSO): *δ* 161.5, 157.9, 157.1, 150.6, 150.3, 143.1, 142.8, 142.3, 142.2, 138.4, 137.4, 136.8, 136.5, 135.7, 135.0, 133.3, 132.6, 130.4, 130.2, 129.0, 128.7, 128.3, 127.6, 127.3, 127.1, 125.5, 125.0, 124.5, 123.8, 118.0, 115.7, 115.4, 113.1, 112.8, 112.1, 109.1, 108.9, 77.1, 60.9, 34.81. HRMS calcd for C_24_H_22_FN_4_O_3_ [M+H]^+^: 433.1670; found: 433.1668.

##### 4.1.4.14 *N*^5^-(5-methyl-3-(5-methylthiophen-2-yl)pyridin-2-yl)-*N*^4^-phenyl-1H-imidazole-4,5-dicarboxamide (**15o**)

Yield: 76%; ^1^H NMR (600 MHz, CDCl_3_): *δ* 13.63 (bs, 1H, NH), 13.08 (bs, 0.7H, NH), 12.92 (bs, 0.3H, NH), 10.70 (bs, 0.7H, NH), 10.54 (bs, 0.3H, NH), 8.31–7.08 (m, 8H), 6.79–6.76 (m, 1H), 2.39 (s, 3H, CH_3_), 2.36 (s, 3H, CH_3_). ^13^C NMR (150 MHz, CDCl_3_): *δ* 184.6, 149.5, 147.4, 144.6, 143.7, 141.2, 138.3, 138.2, 137.4, 137.3, 135.6, 132.9, 129.3, 129.2, 129.1, 128.5, 127.1, 126.8, 126.3, 128.5, 17.5, 15.1. HRMS calcd for C_22_H_19_N_5_O_2_S_1_ [M−H]^+^: 416.1187; found: 416.1187.

#### 4.1.5 General procedure for **18a**–**b**

Sodium dodecyl sulfate (SDS, 6 mmol) was added to a stirred heterogeneous suspension of amine (5 mmol) in water (20 mL) until a homogeneous solution was formed, (in case of turbidity, the mixture was warmed to obtain a clear solution). Anhydride **17** (5 mmol) dissolved in acetonitrile (5 mL) was added to this solution in one lot. After stirring for 1 h at room temperature, the acetonitrile was evaporated and the product precipitated from the aqueous layer. To the aqueous solution containing precipitate, solid sodium hydrogen carbonate was added pinch-wise until the effervescence ceased and the pH was 6.0. The remaining precipitated product was filtered, washed with water (20 mL), dried in a vacuum desiccator. In cases where the product did not precipitate, the reaction mixture was extracted with ethyl acetate (2 × 25 mL). The combined organic extracts were dried with anhydrous Na_2_SO_4_ and the solvent was removed in a rotary evaporator under reduced pressure to yield the pure product.

##### 4.1.5.1 3-((4-Fluorophenyl)carbamoyl)pyrazine-2-carboxylic acid (**18a**) [26]

Yield: 72%; White solid; ^1^H NMR (300 MHz, DMSO): *δ* 10.86 (bs, 1H), 8.90 (s, 2H), 7.83–7.78 (m, 2H), 7.22 (t, 2H, *J* = 9.0 Hz).

##### 4.1.5.2 3-(p-Tolylcarbamoyl)pyrazine-2-carboxylic acid (**18b**) [27]

Yield: 65%; White solid; ^1^H NMR (300 MHz, DMSO): *δ* 10.68 (bs, 1H), 8.88 (s, 2H), 7.67 (d, *J* = 8.4 Hz, 2H), 7.17 (t, *J* = 8.4 Hz, 2H), 2.29 (s, 3H, CH_3_). ^13^C NMR (75 MHz, DMSO): *δ* 166.5, 162.3, 146.5, 145.8, 145.6, 144.6, 136.0, 133.4, 129.3, 120.2, 20.7.

#### 4.1.6 General procedure for **19a**–**b**

Trifluoroacetic anhydride (1.5 equiv) was added dropwise to a stirring solution of acid **18a**–**b** (1 equiv) and Et_3_N (TEA, 3 equiv) in 1,4-dioxane (20 mL) that was kept at 0 °C with an ice bath. After 15 min the yellow solution was allowed to warm to room temperature and was stirred for 30 min, then it was poured in cold H_2_O (100 mL). A precipitate formed, which was collected by filtration using a Buchner funnel and washed with H_2_O. The product was dried overnight under high vacuum.

##### 4.1.6.1 (Z)-7-((4-fluorophenyl)imino)furo[3,4-b]pyrazin-5(7H)-one (**19a**)

Yield: 70%; Off-white solid; ^1^H NMR (300 MHz, CDCl_3_): *δ* 9.06 (d, 2H, *J* = 5.1 Hz), 7.73–7.69 (m, 2H), 7.15 (t, *J* = 8.4 Hz, 2H). ^13^C NMR (75 MHz, CDCl_3_): *δ* 150.9, 149.5, 128.7, 128.6, 116.2, 116.0.

##### 4.1.6.2 (Z)-7-(p-tolylimino)furo[3,4-b]pyrazin-5(7H)-one (**19b**)

Yield: 66%; White solid; ^1^H NMR (300 MHz, CDCl_3_): *δ* 9.07–9.03 (m, 2H), 7.69–7.28 (m, 4H), 2.43 (s, 3H, CH_3_). ^13^C NMR (75 MHz, CDCl_3_): *δ* 150.5, 149.0, 129.5, 126.4, 21.0.

#### 4.1.7 General procedure for **20a**–**n**

Phthalisoimide **19a**–**b** (1 equiv) was added to stirring solution of the aniline/benzylamine **14a**–**m** (1.2 equiv) in THF. The mixture was stirred overnight at room temperature. The THF was evaporated on a rotavapor. The mixture was diluted with EtOAc (triple the volume of THF) and washed three times with 1 M HCl. The organic phase was dried with Na_2_SO_4_ and evaporated. The product was purified by flash chromatography on silica gel.

##### 4.1.7.1 *N*^2^-(4-fluorophenyl)-*N*^3^-(4-(pyridin-4-yl) benzyl) pyrazine-2,3-dicarboxamide (**20a**)

Yield: 76%; ^1^H NMR (300 MHz, DMSO): *δ* 10.65 (bs, 1H, NH), 9.45 (t, *J* = 6.6 Hz, 1H, NH), 8.88 (q, 2H, *J* = 2.4 Hz), 8.64–8.61 (m, 2H), 7.78–7.69 (m, 6H), 7.50 (d, *J* = 8.4 Hz, 2H), 7.21 (t, *J* = 9.0 Hz, 2H), 2.39 (d, *J* = 6.3 Hz, 2H, CH_2_). ^13^C NMR (75 MHz, DMSO): *δ* 164.0, 163.7, 160.1, 156.9, 150.3, 148.3, 146.9, 145.4, 145.3, 144.5, 140.5, 135.8, 135.5, 128.3, 126.9, 121.7, 121.6, 121.2, 115.7, 115.4, 42.2. HRMS calcd for C_24_H_19_F_1_N_5_O_2_ [M+H]^+^: 428.1517; found: 428.1520.

##### 4.1.7.2 *N*^2^-(4-fluorophenyl)-*N*^3^-(4-methyl-2-(pyridin-3-yl) phenyl) pyrazine-2,3-dicarboxamide (**20b**)

Yield: 74%; ^1^H NMR (300 MHz, CDCl_3_): *δ* 9.02 (bs, 1H, NH), 8.69–8.54 (m, 5H), 8.27 (d, *J* = 8.4 Hz, 1H), 7.84 (d, *J* = 8.1 Hz, 1H), 7.70–7.66 (m, 2H), 7.36–7.26 (m, 2H), 7.11–7.03 (m, 3H), 2.39 (s, 3H, CH_3_). ^13^C NMR (75 MHz, CDCl_3_): *δ* 161.8, 161.5, 150.1, 149.1, 144.3, 137.3, 135.7, 134.2, 131.8, 131.0, 130.1, 123.7, 123.2, 122.2, 122.1, 116.1, 115.8, 21.1. HRMS calcd for C_24_H_19_F_1_N_5_O_2_ [M+H]^+^: 428.1517; found: 428.1514.

##### 4.1.7.3 *N*^2^-(4-fluorophenyl)-*N*^3^-(4-(pyridin-3-yl) benzyl) pyrazine-2,3-dicarboxamide (**20c**)

Yield: 72%; ^1^H NMR (300 MHz, CDCl_3_): *δ* 9.33 (bs, 1H, NH), 8.76 (bs, 1H, NH), 8.63 (s, 2H), 8.54–8.52 (d, *J* = 4.8 Hz, 1H), 7.86–7.27 (m, 9H), 7.05 (m, *J* = 8.7 Hz, 2H), 4.76 (d, *J* = 6.0 Hz, 2H, CH_2_). ^13^C NMR (75 MHz, CDCl_3_): δ164.9, 161.6, 161.5, 148.6, 148.3, 147.6, 146.1, 144.6, 144.0, 137.8, 137.3, 136.3, 134.4, 133.6, 128.8, 127.6, 123.7, 122.1, 122.0, 116.0, 115.7, 43.7. HRMS calcd for C_24_H_19_F_1_N_5_O_2_ [M+H]^+^: 428.1517; found: 428.1517.

##### 4.1.7.4 *N*^2^-(4-fluorophenyl)-*N*^3^-(2-(pyridin-4-yl) phenyl) pyrazine-2,3-dicarboxamide (**20d**)

Yield: 66%; ^1^H NMR (300 MHz, CDCl_3_): *δ* 10.72 (bs, 1H, NH), 10.27 (bs, 1H, NH), 8.90–8.86 (m, 2H), 8.58–8.56 (m, 2H), 7.85–7.76 (m, 3H), 7.55–7.37 (m, 5H), 7.22 (t, *J* = 9.0 Hz, 2H). ^13^C NMR (75 MHz, CDCl_3_): *δ* 163.2, 163.1, 149.8, 147.0, 146.2, 145.1, 144.9, 135.2, 134.4, 133.6, 130.3, 129.3, 126.6, 126.2, 124.0, 121.9, 121.8, 115.7, 115.4, 79.3. HRMS calcd for C_23_H_17_F_1_N_5_O_2_ [M+H]^+^: 414.1361; found: 414.1363.

##### 4.1.7.5 *N*^2^-(4-fluorophenyl)-*N*^3^-(2-(pyridin-3-yl) phenyl) pyrazine-2,3-dicarboxamide (**20e**)

Yield: 76%; ^1^H NMR (300 MHz, CDCl_3_): *δ* 9.04 (bs, 1H, NH), 8.78 (bs, 1H, NH), 8.70–8.56 (m, 4H), 8.45 (d, *J* = 8.1 Hz, 1H), 7.87 (d, *J* = 7.8 Hz, 1H), 7.72–7.69 (m, 2H), 7.52–7.7.28 (m, 4H), 7.07 (t, *J* = 8.7 Hz, 2H). ^13^C NMR (75 MHz, CDCl_3_): *δ* 161.7, 161.5, 150.1, 149.2, 144.3, 137.3, 134.4, 134.0, 133.5, 130.5, 129.9, 129.5, 125.8, 123.7, 123.1, 122.2, 122.1, 116.1, 115.8. HRMS calcd for C_23_H_17_F_1_N_5_O_2_ [M+H]^+^: 414.1361; found: 414.1365.

##### 4.1.7.6 *N*^2^-(4-fluorophenyl)-*N*^3^-(2-(pyridin-4-yl) benzyl) pyrazine-2,3-dicarboxamide (**20f**)

Yield: 68%; ^1^H NMR (600 MHz, DMSO): *δ* 10.67 (bs, 1H, NH), 9.41 (t, *J* = 6.0 Hz, 1H), 8.91–8.89 (m, 2H), 7.75–7.19 (m, 8H), 4.51 (d, *J* = 6.0 Hz, 2H). ^13^C NMR (125 MHz, DMSO): *δ* 164.3, 163.6, 159.2, 157.6, 150.6, 149.0, 148.1, 145.3, 144.5, 137.4, 135.3, 132.4, 129.0, 128.5, 127.8, 122.1, 121.5, 115.5, 115.4, 42.9. HRMS calcd for C_24_H_19_F_1_N_5_O_2_ [M+H]^+^: 428.1517; found: 428.1520.

##### 4.1.7.7 *N*^2^-(4-fluorophenyl)-*N*^3^-(4-methyl-2-(pyridin-4-yl)phenyl)pyrazine-2,3-dicarbox amide (**20g**)

Yield: 76%; ^1^H NMR (300 MHz, CDCl_3_): *δ* 8.95 (bs, 1H, NH), 8.66–8.58 (m, 5H), 8.32 (d, *J* = 8.4 Hz, 1H), 7.72–7.67 (m, 2H), 7.43 (d, *J* = 8.4 Hz, 2H), 7.30 (d, *J* = 8.4 Hz, 2H), 7.13–7.7.04 (m, 3H), 2.40 (s, 3H, CH_3_). ^13^C NMR (75 MHz, CDCl_3_): *δ* 150.0, 145.9, 144.0, 143.8, 130.9, 130.0, 129.9, 124.0, 122.6, 121.7, 121.6, 115.6, 115.3, 20.6. HRMS calcd for C_24_H_19_F_1_N_5_O_2_ [M+H]^+^: 428.1517; found: 428.1518.

##### 4.1.7.8 *N*^2^-(4-(pyridin-4-yl) benzyl)-*N*^3^-(p-tolyl) pyrazine-2,3-dicarboxamide (**20h**)

Yield: 78%; ^1^H NMR (300 MHz, CDCl_3_): *δ* 9.19 (bs, 1H, NH), 8.66–8.62 (m, 4H), 7.64–7.47 (m, 8H), 7.17 (d, *J* = 8.1 Hz, 3H), 4.78 (d, *J* = 6.0 Hz, 2H, CH_2_), 2.36 (s, 3H, CH_3_). ^13^C NMR (75 MHz, CDCl_3_): *δ* 165.1, 161.2, 150.4, 148.1, 148.0, 145.8, 144.8, 143.8, 139.0, 137.6, 134.9, 134.8, 129.7, 128.8, 127.5, 121.7, 120.3, 43.7, 21.1. HRMS calcd for C_25_H_22_N_5_O_2_ [M+H]^+^: 424.1768; found: 424.1765.

##### 4.1.7.9 *N*^2^-(4-methyl-2-(pyridin-3-yl) phenyl)-*N*^3^-(p-tolyl) pyrazine-2,3-dicarboxamide (**20i**)

Yield: 76%; ^1^H NMR (300 MHz, CDCl_3_): *δ* 8.95 (bs, 1H, NH), 8.72–8.56 (m, 5H), 8.33 (d, *J* = 8.1 Hz, 1H), 7.89 (d, *J* = 7.5 Hz, 1H), 7.62 (d, *J* = 8.1 Hz, 2H), 7.38–7.14 (m, 4H), 2.42 (s, 3H, CH_3_), 2.37 (s, 3H, CH_3_). ^13^C NMR (75 MHz, CDCl_3_): *δ* 150.2, 149.2, 144.5, 144.1, 137.3, 135.5, 134.8, 134.2, 131.9, 131.0, 130.1, 123.6, 123.3, 120.3, 21.1. HRMS calcd for C_25_H_22_N_5_O_2_ [M+H]^+^: 424.1768; found: 424.1767.

##### 4.1.7.10 *N*^2^-(4-(pyridin-3-yl) benzyl)-*N*^3^-(p-tolyl) pyrazine-2,3-dicarboxamide (**20j**)

Yield: 71%; ^1^H NMR (300 MHz, CDCl_3_): *δ* 9.24 (bs, 1H, NH), 8.78–8.54 (m, 4H), 7.88–7.84 (m, 1H), 7.63 (d, *J* = 8.4 Hz, 2H), 7.56–7.17 (m, 8H), 4.79 (d, *J* = 6.0 Hz, 2H, CH_2_), 2.36 (s, 3H, CH_3_). ^13^C NMR (75 MHz, CDCl_3_): *δ* 165.2, 161.2, 148.6, 148.3, 148.1, 145.7, 144.6, 143.8, 137.9, 137.2, 136.4, 134.9, 134.7, 134.4, 129.7, 128.9, 127.6, 123.7, 120.3, 43.7, 21.1. HRMS calcd for C_25_H_22_N_5_O_2_ [M+H]^+^: 424.1768; found: 424.1769.

##### 4.1.7.11 *N*^2^-(2-(pyridin-4-yl) phenyl)-*N*^3^-(p-tolyl) pyrazine-2,3-dicarboxamide (**20k**)

Yield: 72%; ^1^H NMR (300 MHz, CDCl_3_): *δ* 8.95 (bs, 1H, NH), 8.68–8.51 (m, 6H), 7.63 (d, *J* = 8.4 Hz, 2H), 7.50–7.19 (m, 7H), 2.37 (s, 3H, CH_3_). ^13^C NMR (75 MHz, CDCl_3_): *δ* 150.5, 146.2, 144.6, 144.1, 134.9, 134.1, 129.9, 129.8, 125.7, 124.5, 123.0, 120.3, 21.1. HRMS calcd for C_24_H_20_N_5_O_2_ [M+H]^+^: 410.1611; found: 410.1608.

##### 4.1.7.12 *N*^2^-(2-(pyridin-3-yl) phenyl)-*N*^3^-(p-tolyl) pyrazine-2,3-dicarboxamide (**20l**)

Yield: 68%; ^1^H NMR (300 MHz, CDCl_3_): *δ* 8.70 (bs, 1H, NH), 8.70–8.47 (m, 6H), 7.90 (d, *J* = 7.8 Hz, 2H), 7.63–7.18 (m, 8H), 2.36 (s, 3H, CH_3_). ^13^C NMR (75 MHz, CDCl_3_): *δ* 162.0, 160.1, 149.6, 148.5, 144.0, 143.7, 137.0, 123.2, 134.0, 133.6, 120.0, 129.2, 129.1, 125.2, 123.2, 122.8, 119.8, 20.6. HRMS calcd for C_24_H_20_N_5_O_2_ [M+H]^+^: 410.1611; found: 410.1606.

##### 4.1.7.13 *N*^2^-(4-(pyridin-4-yl) benzyl)-*N*^3^-(p-tolyl) pyrazine-2,3-dicarboxamide (**20m**)

^1^H NMR (300 MHz, CDCl_3_): *δ* 9.13 (bs, 1H, NH), 8.77–8.66 (m, 3H), 7.71–7.16 (m, 11H), 4.82 (s, 2H, CH_2_), 2.36 (s, 3H, CH_3_). ^13^C NMR (75 MHz, CDCl_3_): *δ* 161.3, 150.8, 144.6, 143.9, 136.9, 132.9, 130.5, 129.7, 129.4, 128.7, 128.6, 128.0, 121.6, 120.3, 44.3, 21.1. HRMS calcd for C_25_H_22_N_5_O_2_ [M+H]^+^: 424.1768; found: 424.1768.

##### 4.1.7.14 *N*^2^-(4-methyl-2-(pyridin-4-yl) phenyl)-*N*^3^-(p-tolyl) pyrazine-2,3-dicarboxamide (**20n**)

Yield: 73%; ^1^H NMR (300 MHz, CDCl_3_): *δ* 8.94 (bs, 1H, NH), 8.68–8.62 (m, 4H), 8.49 (bs, 1H, NH), 8.37 (d, *J* = 8.4 Hz, 1H), 7.63 (d, *J* = 8.4 Hz, 2H), 7.48–7.14 (m, 6H), 2.42 (s, 3H, CH_3_), 2.37 (s, 3H, CH_3_). ^13^C NMR (75 MHz, CDCl_3_): *δ* 161.3, 150.5, 146.4, 144.5, 144.1, 134.8, 131.5, 130.4, 129.8, 124.5, 123.2, 120.3, 21.1. HRMS calcd for C_25_H_22_N_5_O_2_ [M+H]^+^: 424.1768; found: 424.1770.

### 4.2 Antiviral activity determination for DENV and YFV

Green monkey kidney cells [Vero-B cells as obtained from the European Collection of Cell Cultures (ECACC)] were grown in minimum essential medium (MEM; Gibco, Merelbeke, Belgium) supplemented with 10% fetal calf serum (FCS), 1% l-glutamine and 1% sodium bicarbonate. Vero-B cells were seeded at a density 7 × 10^3^ cells/well in 100 μL assay medium and allowed to adhere overnight. Antiviral assays were performed in medium supplemented with 2% FCS, 1% l-glutamine and 1% sodium bicarbonate. After washing cells twice with 2% FCS medium, serial compound dilutions (1:2) were added to each well (starting concentration 100 μg/mL), following by adding 100 μL of 2% of phosphate buffered saline (PBS) culture medium containing 100 μL 50% cell culture infectious doses (i.e., CCID_50_) of virus. After 7 days of incubation, 2% FCS culture medium was discarded and cells were fixed with ethanol and stained with 1% methylene blue, and EC_50_ and CC_50_ were determinate visually. The 50% effective concentration (EC50), which is defined as the compound concentration that is required to inhibit the virus-induced cytopathogenic effect (CPE) by 50%, and 50% cytotoxic concentration (CC_50_), which is defined as the compound concentration that is required to inhibit the cell growth by 50%.

## Appendix A Supplementary data

### MOL files

The following ZIP file contains the MOL files of the most important compound referred to in this article.

MOL file ZIP file containing the MOL files of the most important compound in this article.
