# Supplementary material for: Synthesis and evaluation of imidazole-4,5- and pyrazine-2,3-dicarboxamides targeting dengue and yellow fever virus
Source: Eur J Med Chem. 2014 Nov 24;87:529–39. doi: 10.1016/j.ejmech.2014.09.062 (PMC4237513; doi:10.1016/j.ejmech.2014.09.062)

Supporting Information

Synthesis and evaluation of imidazole-4,5- and pyrazine-2,3-dicarboxamides targeting Dengue and Yellow fever virus

Milind Saudi[a], Joanna Zmurko[b], Suzanne Kaptein[b], Jef Rozenski[a], Johan Neyts[b], Arthur Van Aerschot[a*]

[a]Medicinal Chemistry and [b]Virology and Chemotherapy, Rega Institute for Medical Research, KU Leuven, Minderbroedersstraat 10, BE-3000 Leuven, Belgium

List of contents

General Information……………….…………….…………………….…....S2

HRMS, ^{1}H and ^{13}C NMR spectra ……...……..…..…...…………S3-S70

General Information

Reagents and solvents were purchased from commercial suppliers (Acros, Sigma-Aldrich, Bachem, Novabiochem) and used as provided, unless indicated otherwise. All the solvents were of analytical grade and were stored over 4Å molecular sieves. Reactions were carried out in oven-dried glassware under a nitrogen atmosphere with stirring at 85ºC.

^{1}H and ^{13}C NMR spectra of the compounds dissolved in $CDCl_3$, MeOD or DMSO-d_6 were recorded on a Bruker UltraShield Avance 300 MHz or 500 MHz spectrometer. The chemical shifts are expressed as δ values in parts per million (ppm), using the residual solvent peaks ($CDCl_3$: ^{1}H, 7.26 ppm; ^{13}C, 77.00 ppm; MeOD: ^{1}H, 3.31 ppm; ^{13}C, 49.00 ppm) as a reference. Coupling constants are given in Hertz (Hz). The peak patterns are indicated by the following abbreviations: bs = broad singlet, d = doublet, m = multiplet, q = quadruplet, s = singlet and t = triplet. High resolution mass spectra were recorded on a quadrupole time-of-flight mass spectrometer (Q-Tof-2, Micromass, Manchester, UK) equipped with a standard ESI interface; samples were infused in 2-propanol/H_2O (1:1) at 3 µL.min^{-1}.

For TLC, precoated aluminium sheets were used (Merck, Silica gel 60 F_{254}). The spots were visualized by UV light at 254 nm. Column chromatography was performed on ICN silica gel 60Aº 40-60 µM.

This supporting information provides the ^{1}H and ^{13}C NMR and HRMS spectra for all new final compounds with numbering according to the manuscript.

HRMS, ^{1}H and ^{13}C NMR Spectra

5,10-dioxo-*N*^{1},*N*^{6}-diphenyl-5,10-dihydrodiimidazo[1,5-*a*:1',5'-*d*]pyrazine-1,6-dicarboxamide (12a)

HRMS (ES+)

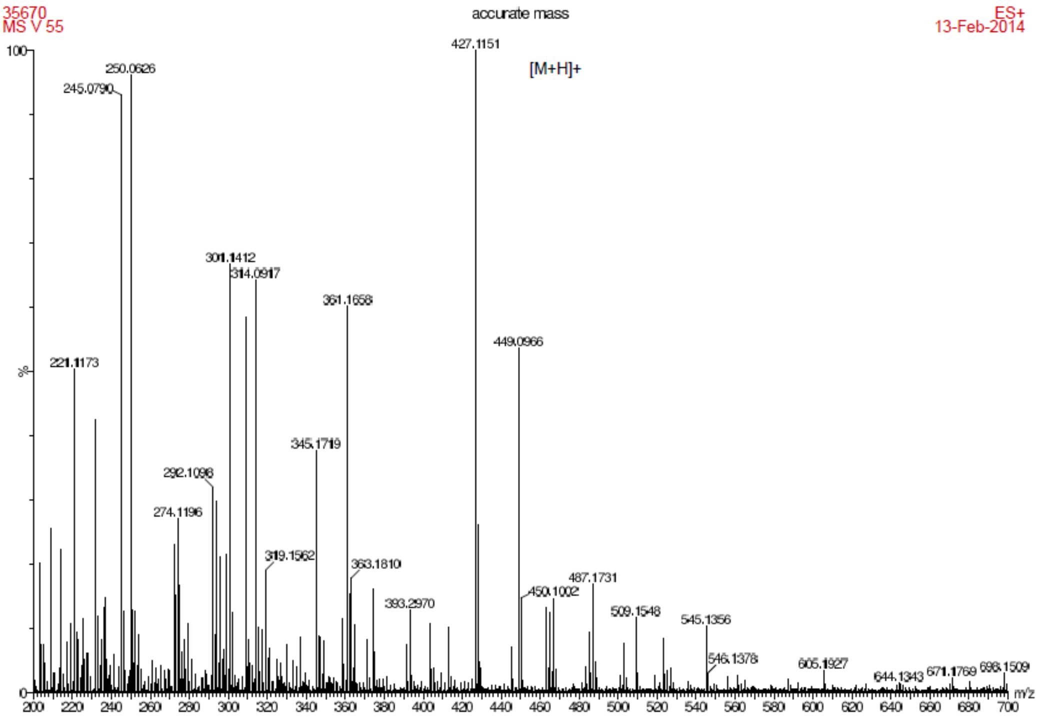

^{1}H NMR (300 MHz, DMSO)

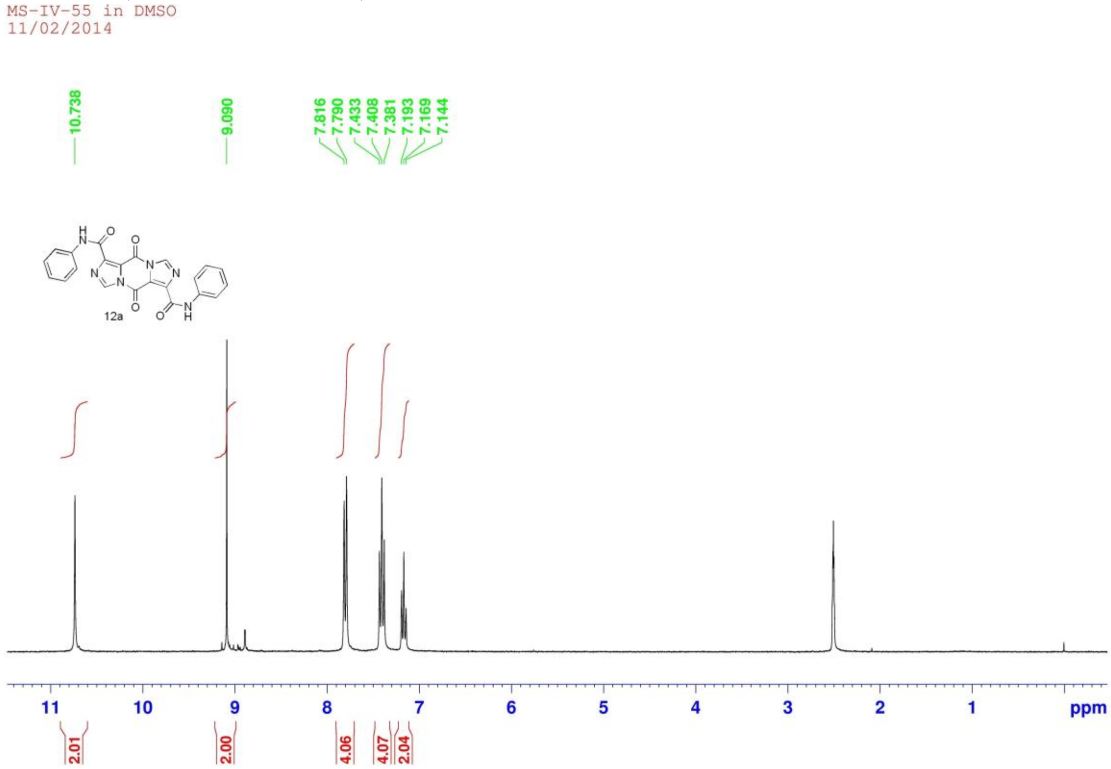

^{13}C NMR (75 MHz, DMSO)

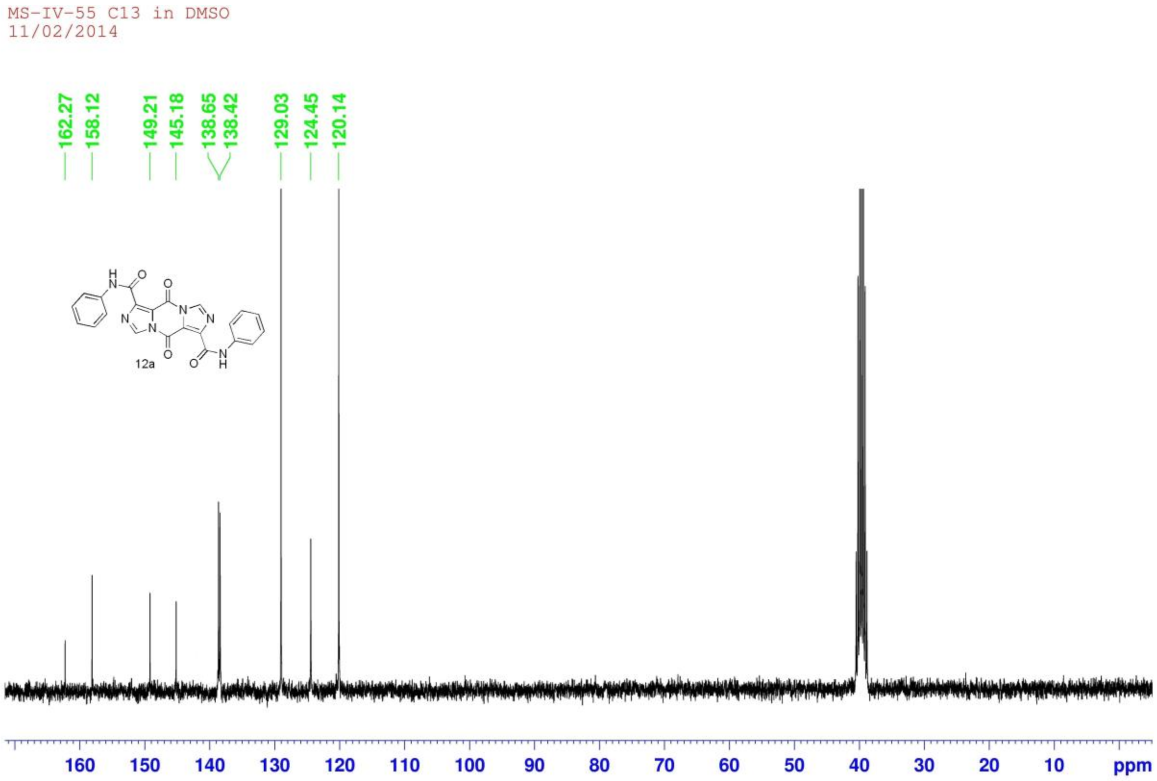

N^1,N^6-bis(4-fluorophenyl)-5,10-dioxo-5,10-dihydrodiimidazo[1,5-*a*:1',5'-*d*]pyrazine-1,6-dicarboxamide (12b)

HRMS (ES+)

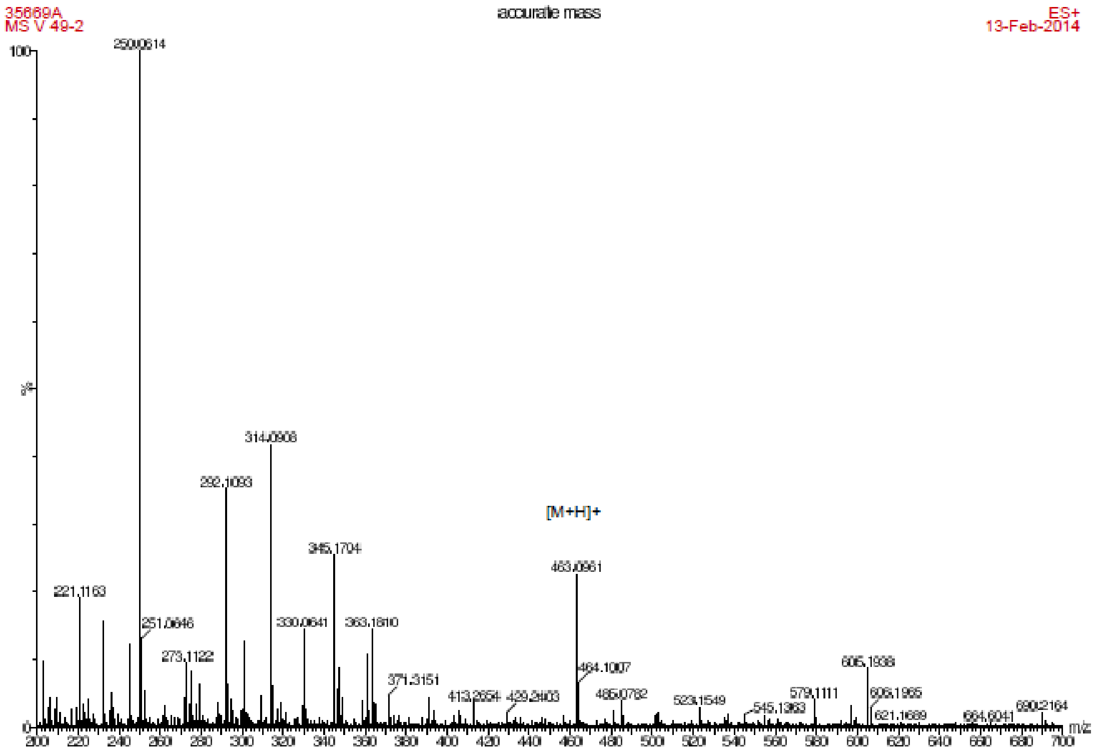

^{1}H NMR (300 MHz, DMSO)

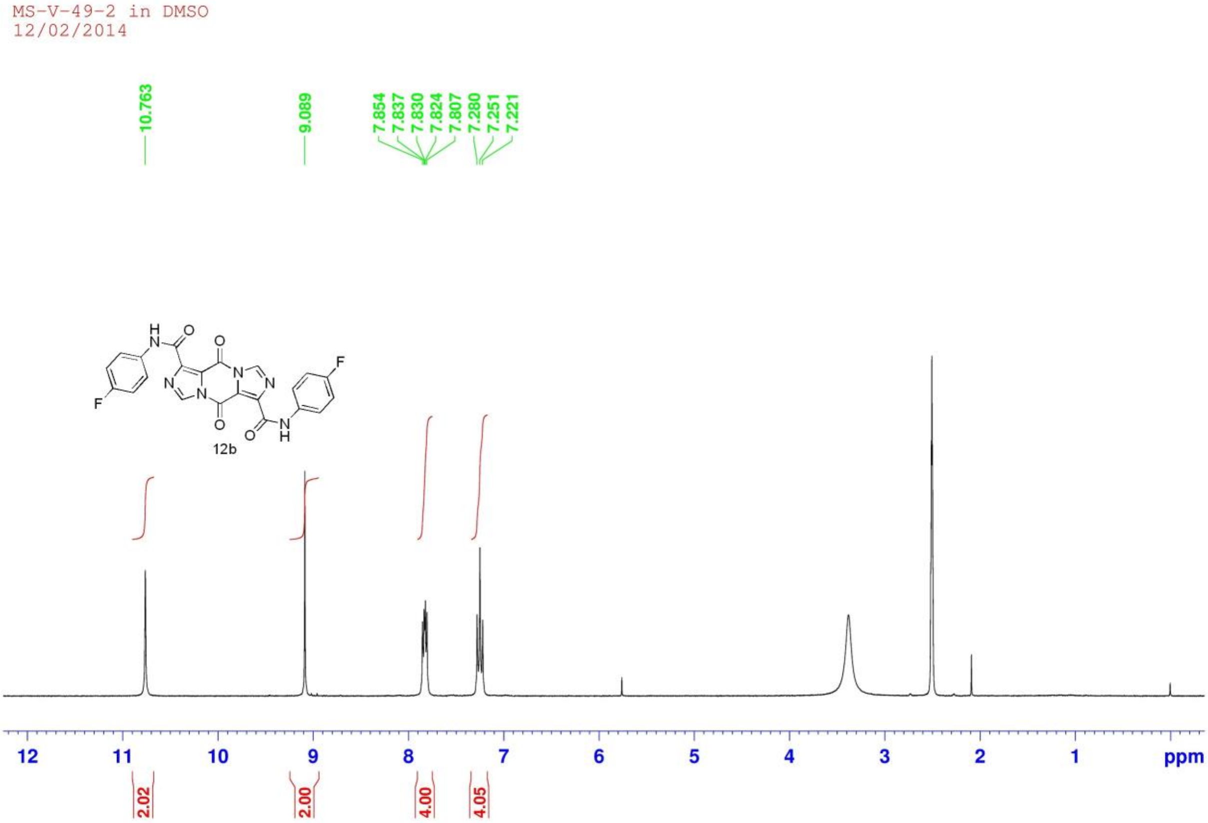

^{13}C NMR (75 MHz, DMSO)

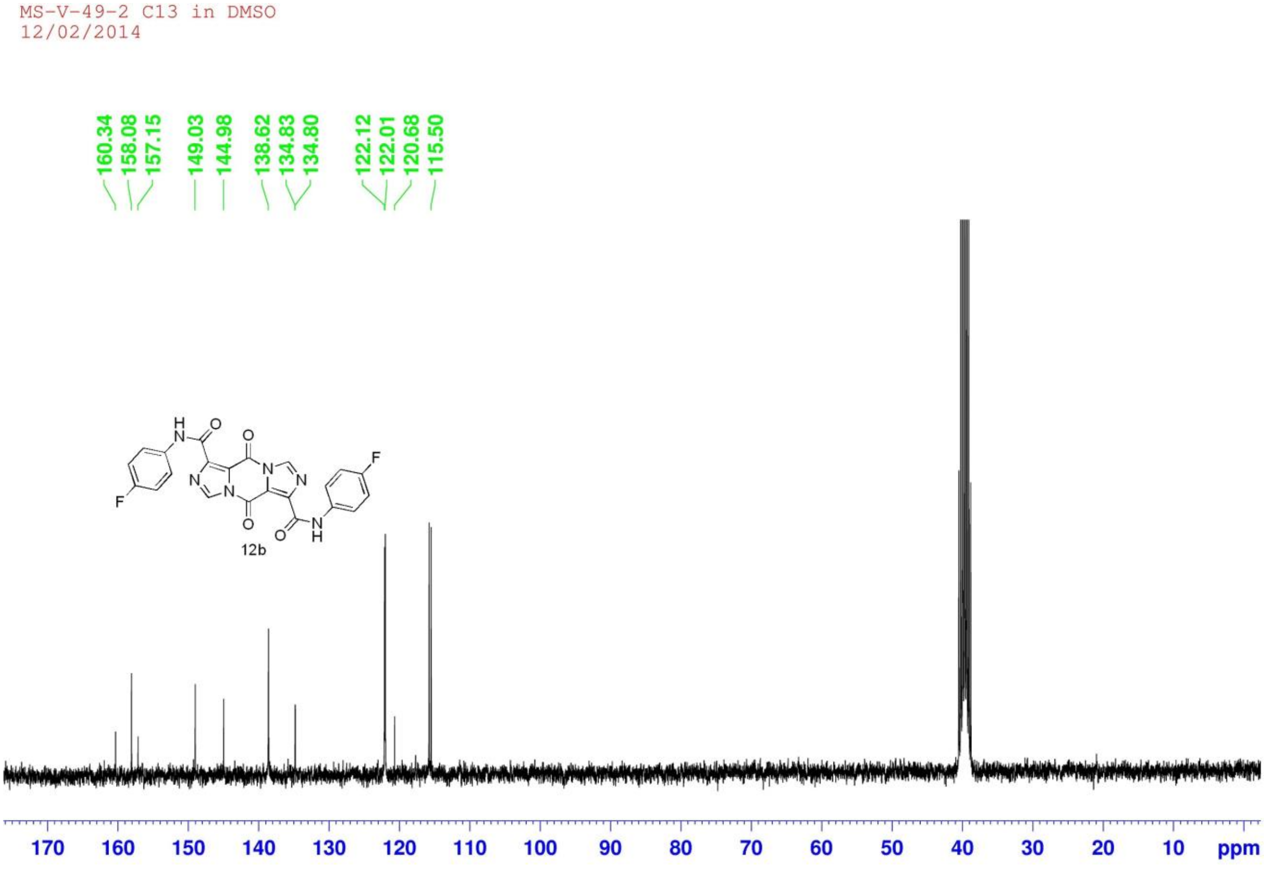

5,10-dioxo-*N*[1],*N*[6]-di-p-tolyl-5,10-dihydrodiimidazo[1,5-*a*:1',5'-*d*]pyrazine-1,6-dicarboxamide (12c)

HRMS (ES+)

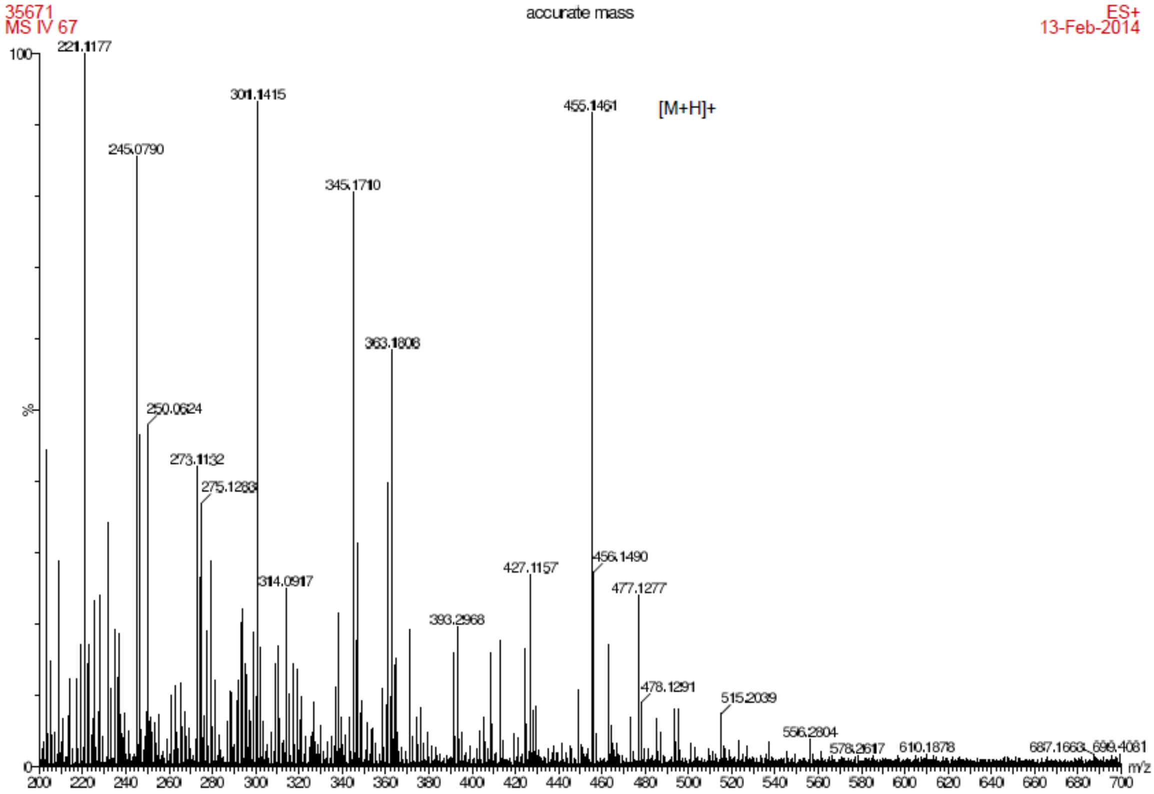

^{1}H NMR (600 MHz, DMSO)

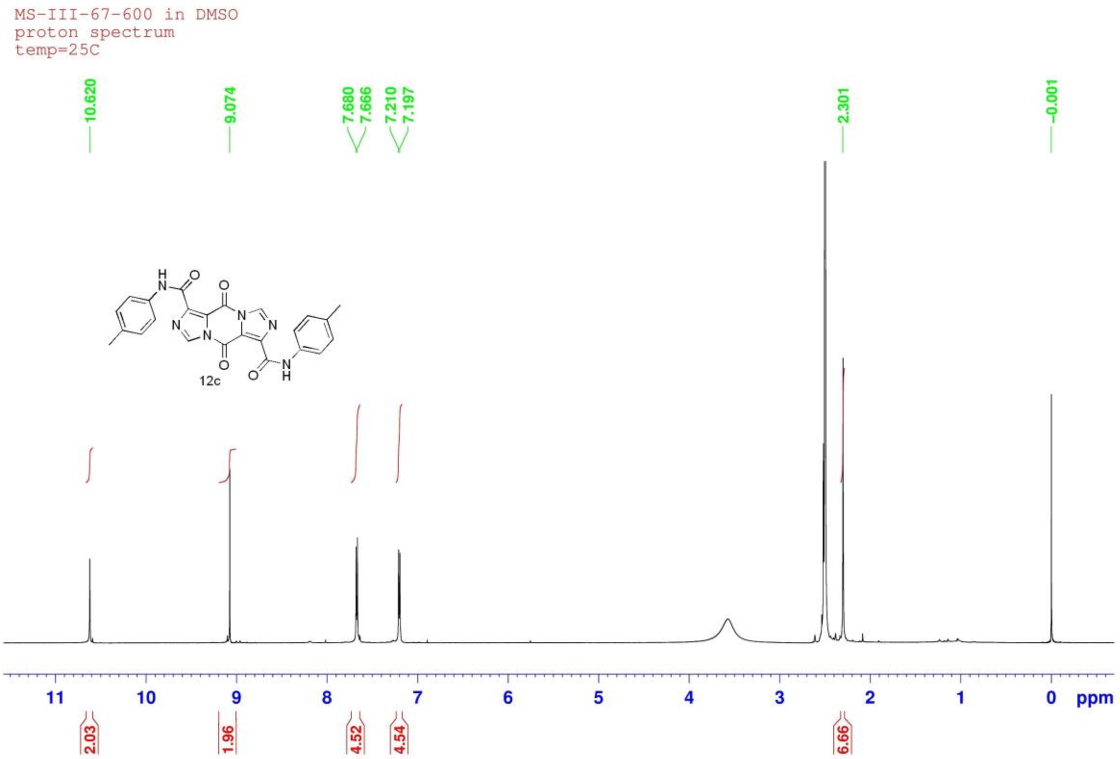

^{13}C NMR (150 MHz, DMSO)

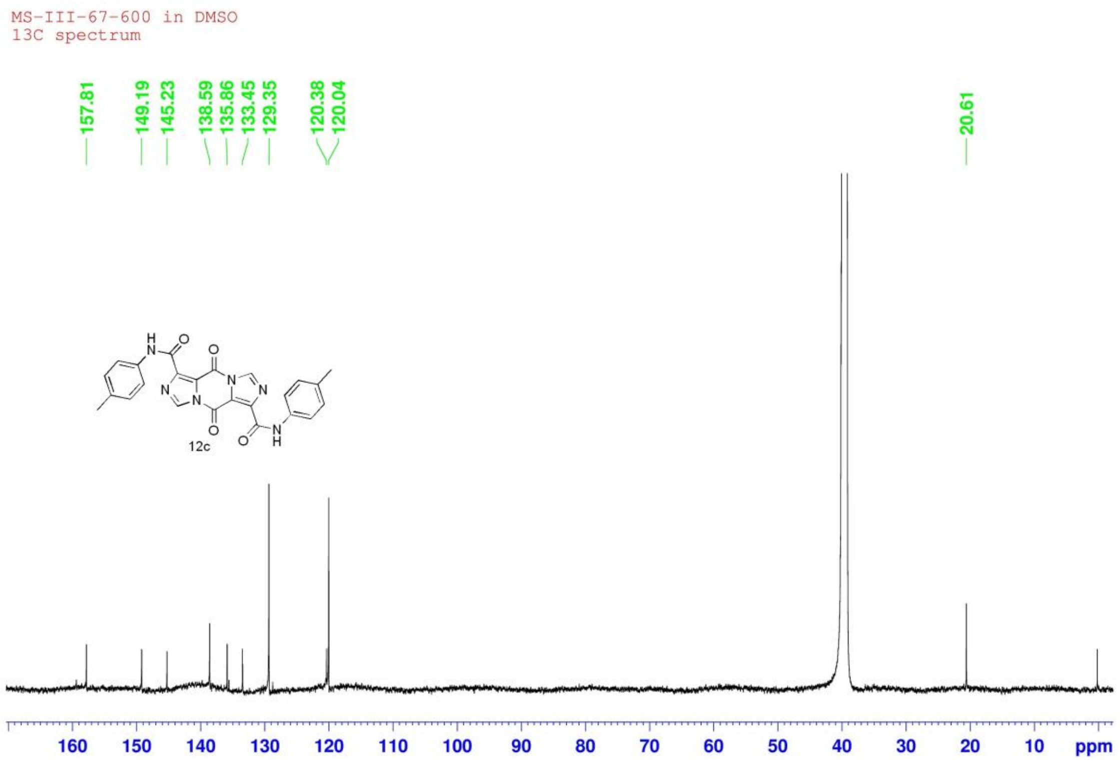

N^1,N^6-bis(4-methyl-2-(thiophen-2-yl)phenyl)-5,10-dioxo-5,10-dihydrodiimidazo [1,5-a:1',5'-d]pyrazine-1,6-dicarboxamide (12d)

HRMS (ES+)

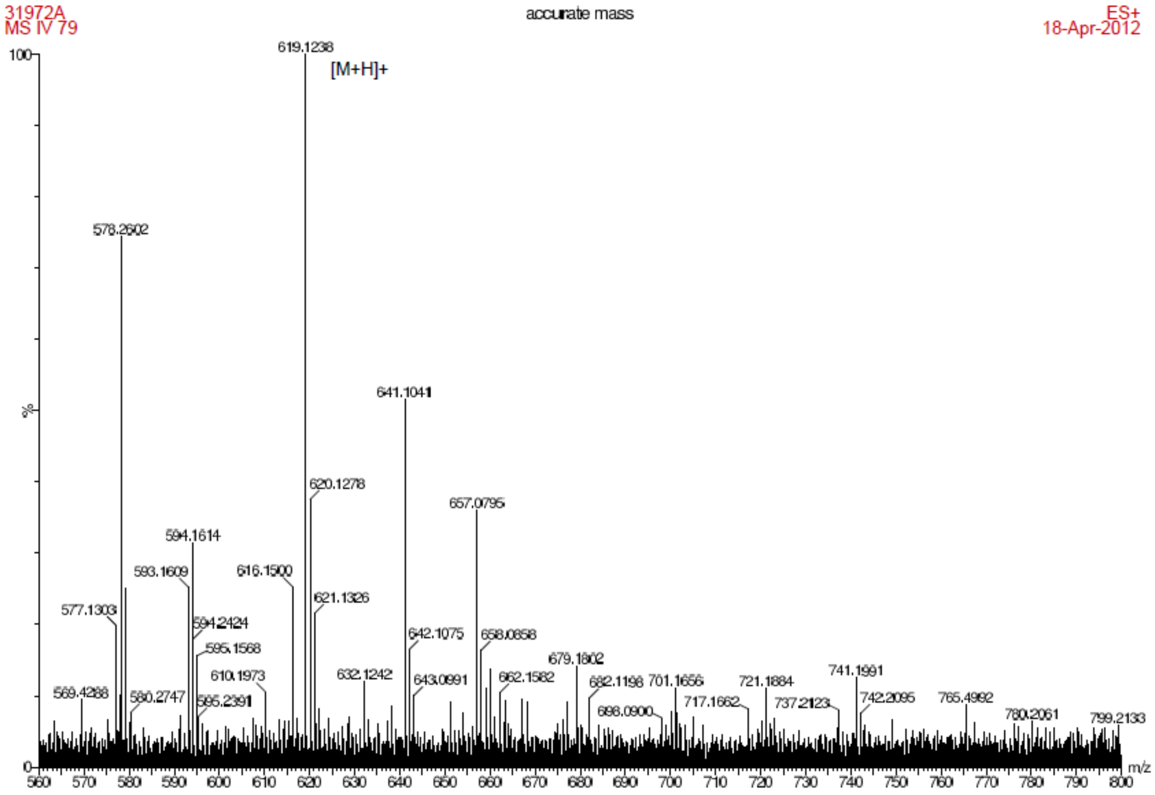

^{1}H NMR (500 MHz, CDCl$_3$)

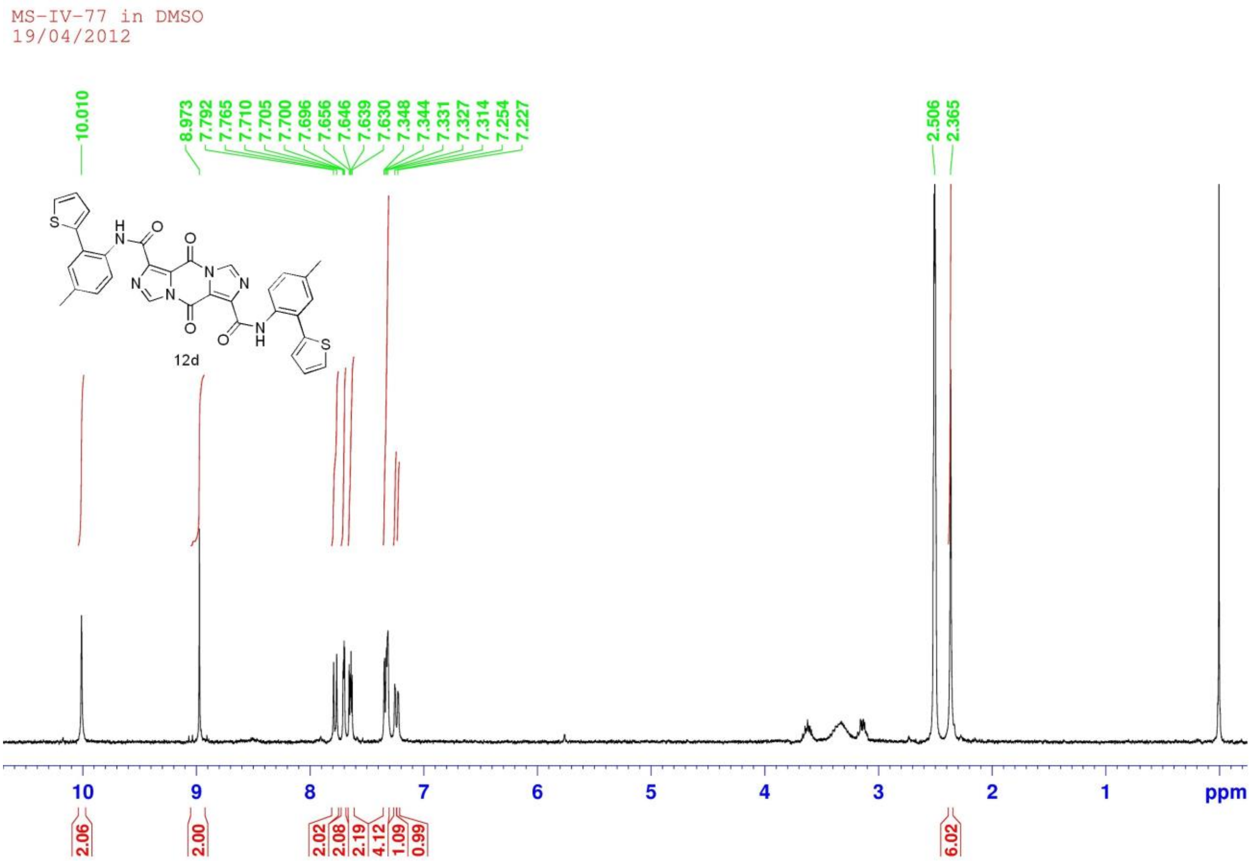

N^1,N^6-bis(2-(furan-2-yl)-4-methylphenyl)-5,10-dioxo-5,10-dihydrodiimidazo [1,5-a:1',5'-d]pyrazine-1,6-dicarboxamide (12e)

HRMS (ES+)

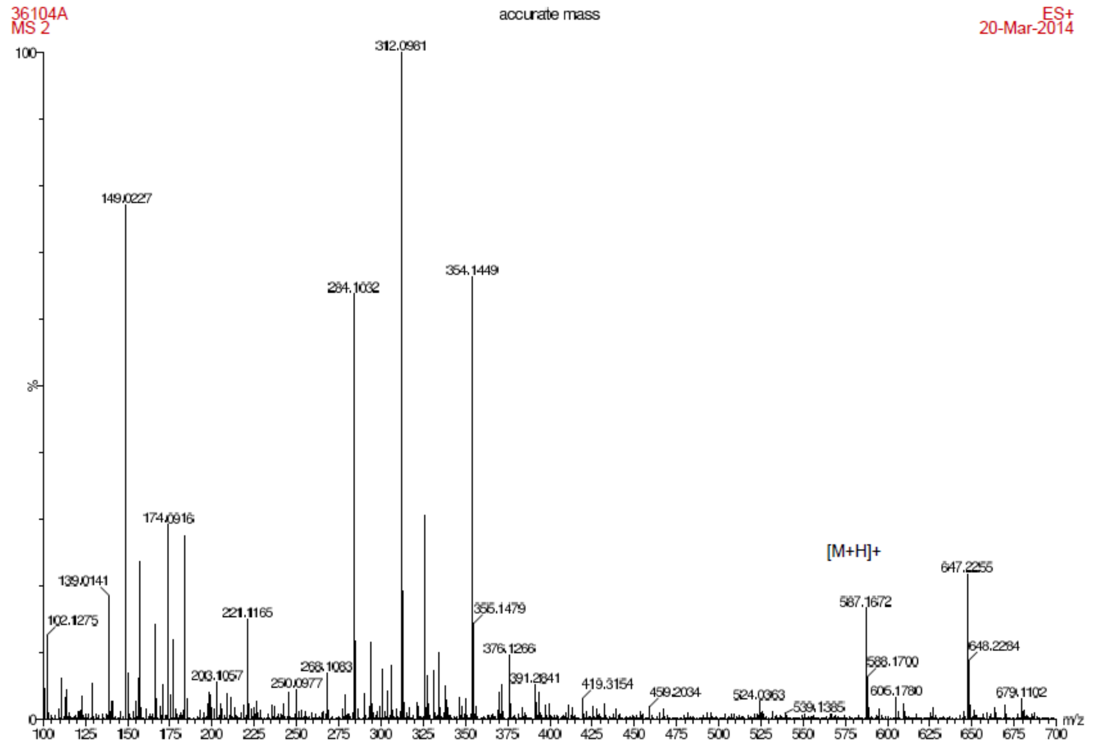

^{1}H NMR (500 MHz, $CDCl_3$)

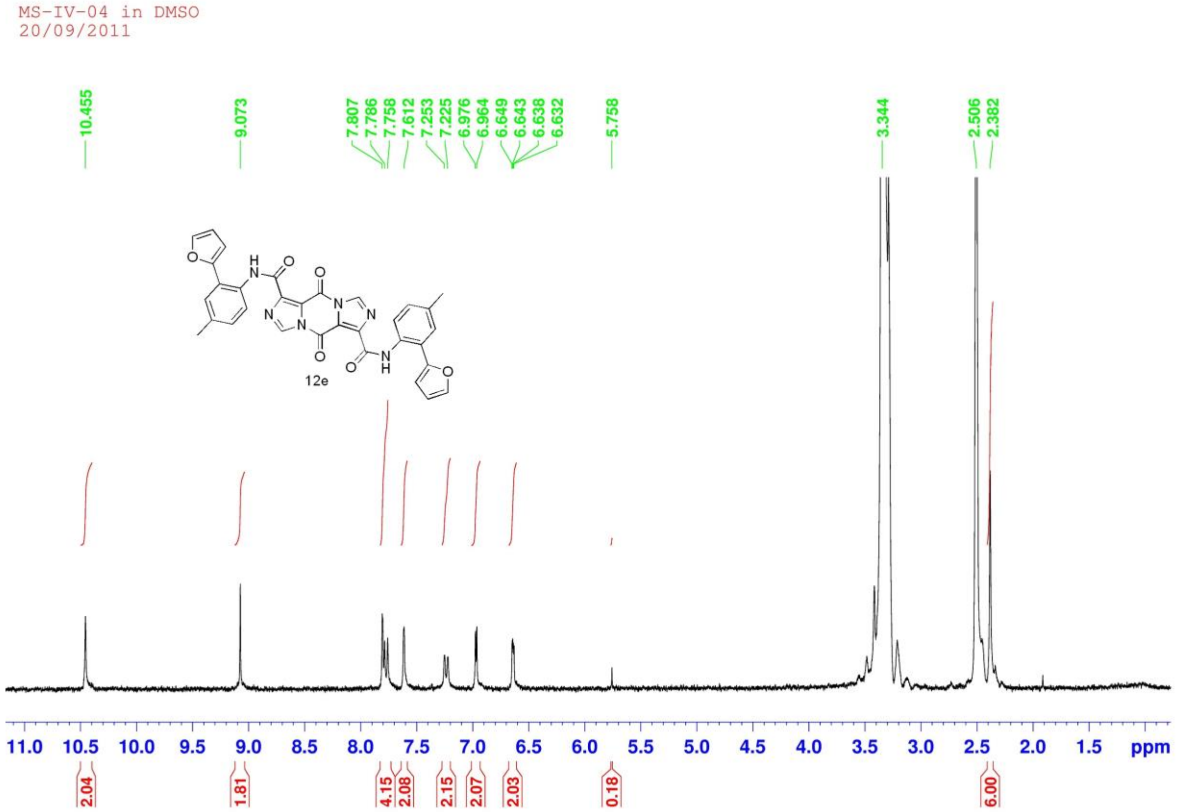

4-methyl-2-(pyridin-3-yl) aniline (14b)

HRMS (ES+)

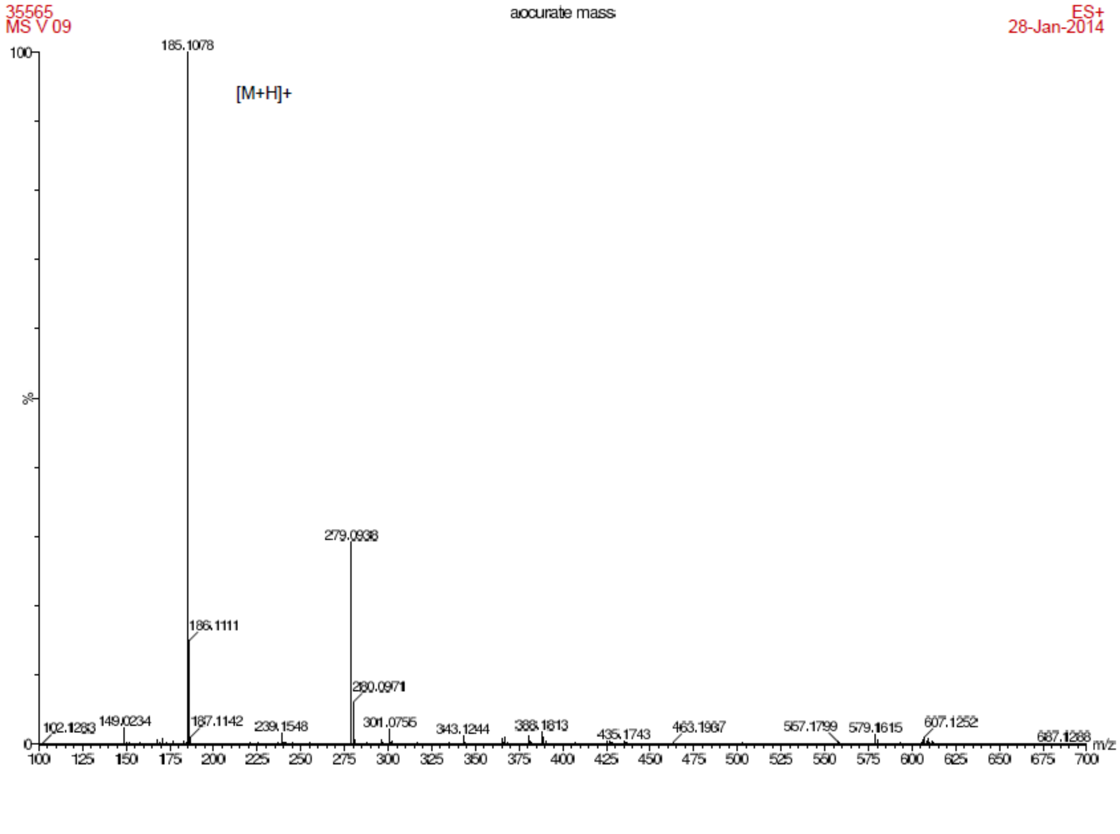

^{1}H NMR (300 MHz, $CDCl_3$)

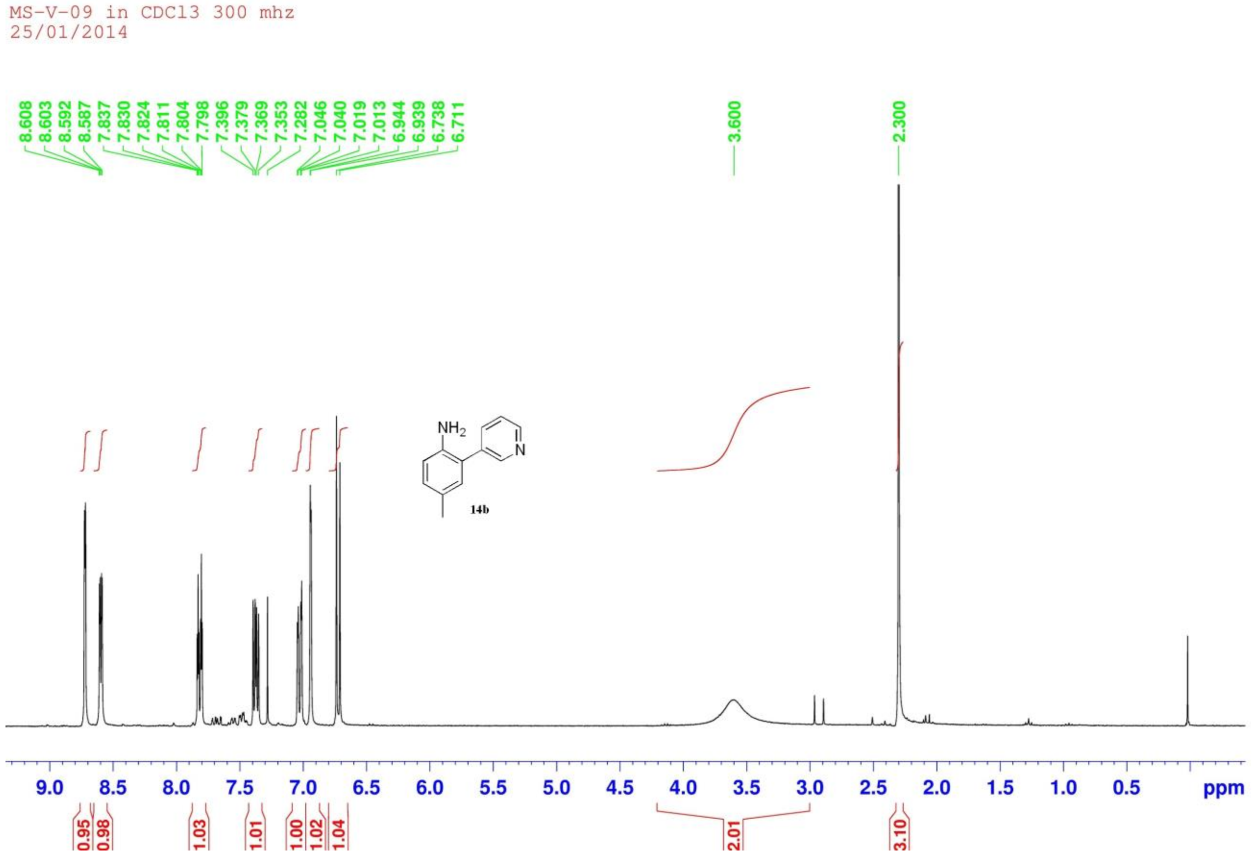

^{13}C NMR (75 MHz, $CDCl_3$)

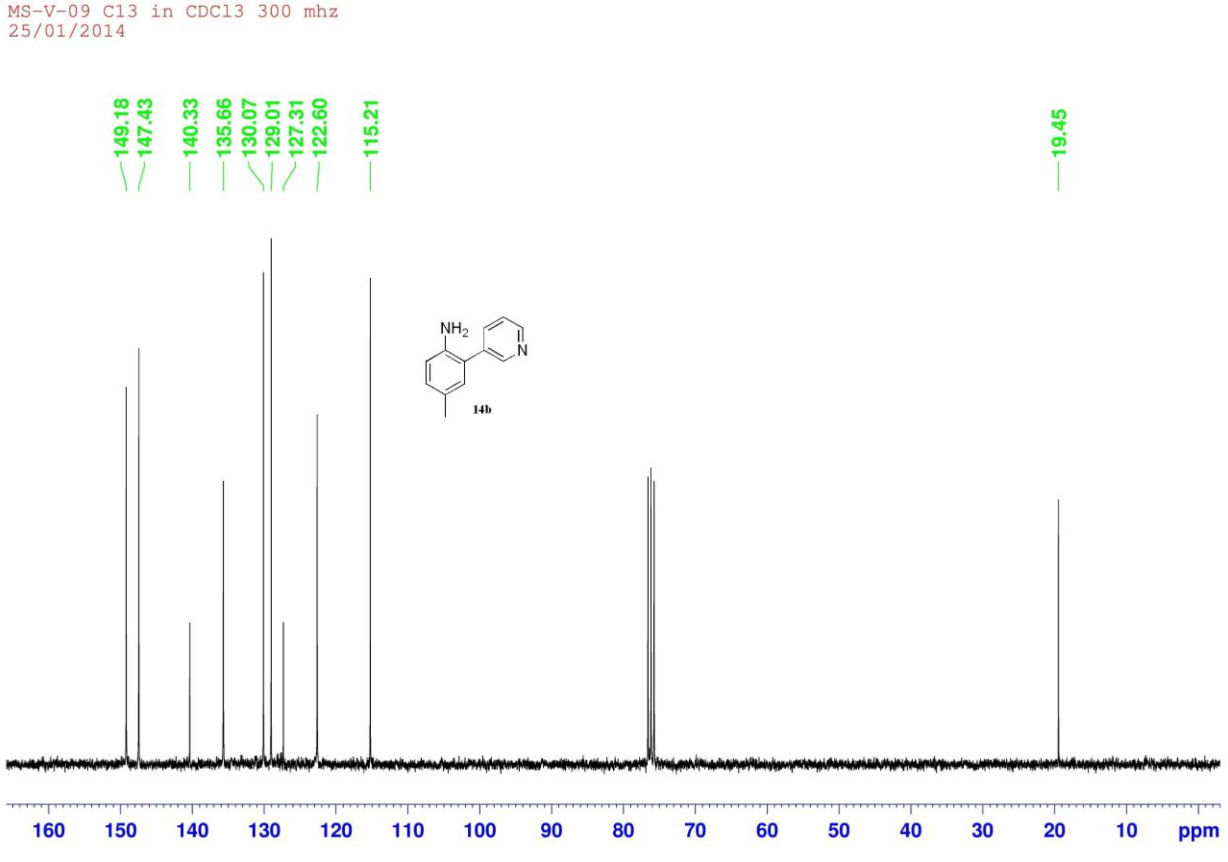

4-methyl-2-(pyridin-4-yl)aniline (14c)

HRMS (ES+)

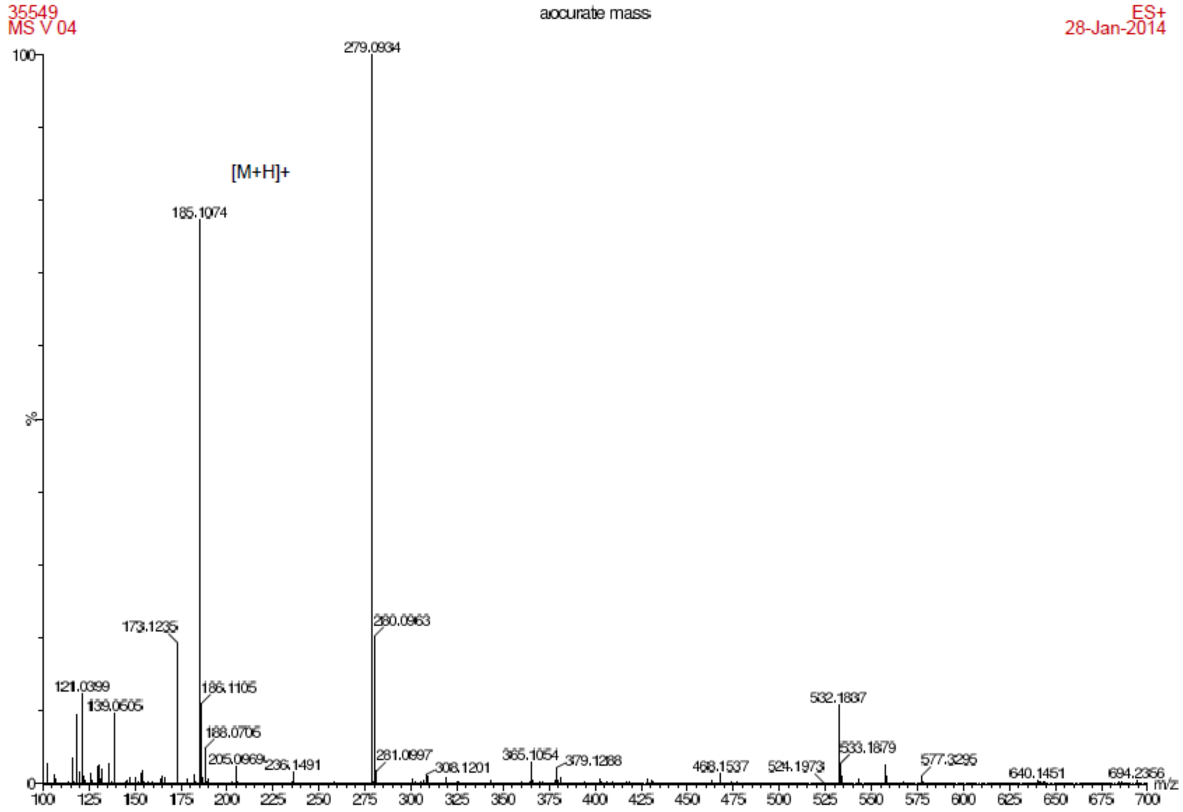

^{1}H NMR (300 MHz, $CDCl_3$)

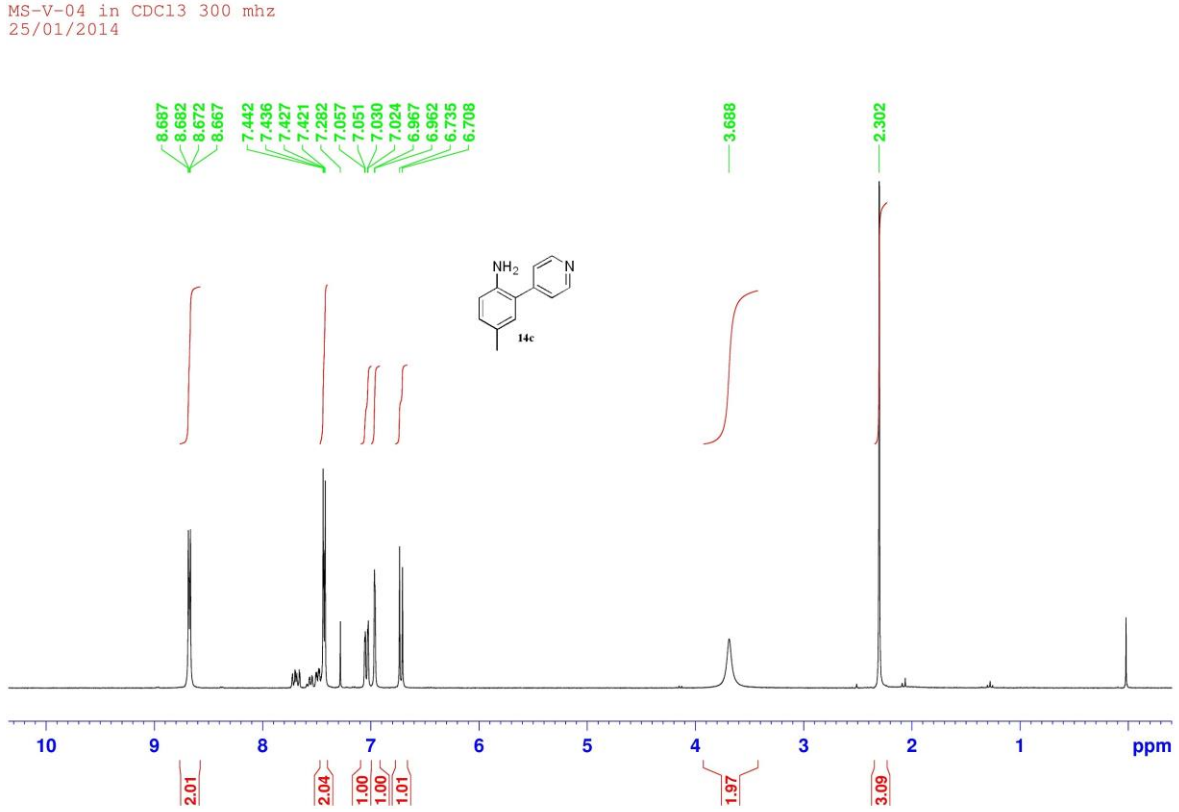

^{13}C NMR (75 MHz, $CDCl_3$)

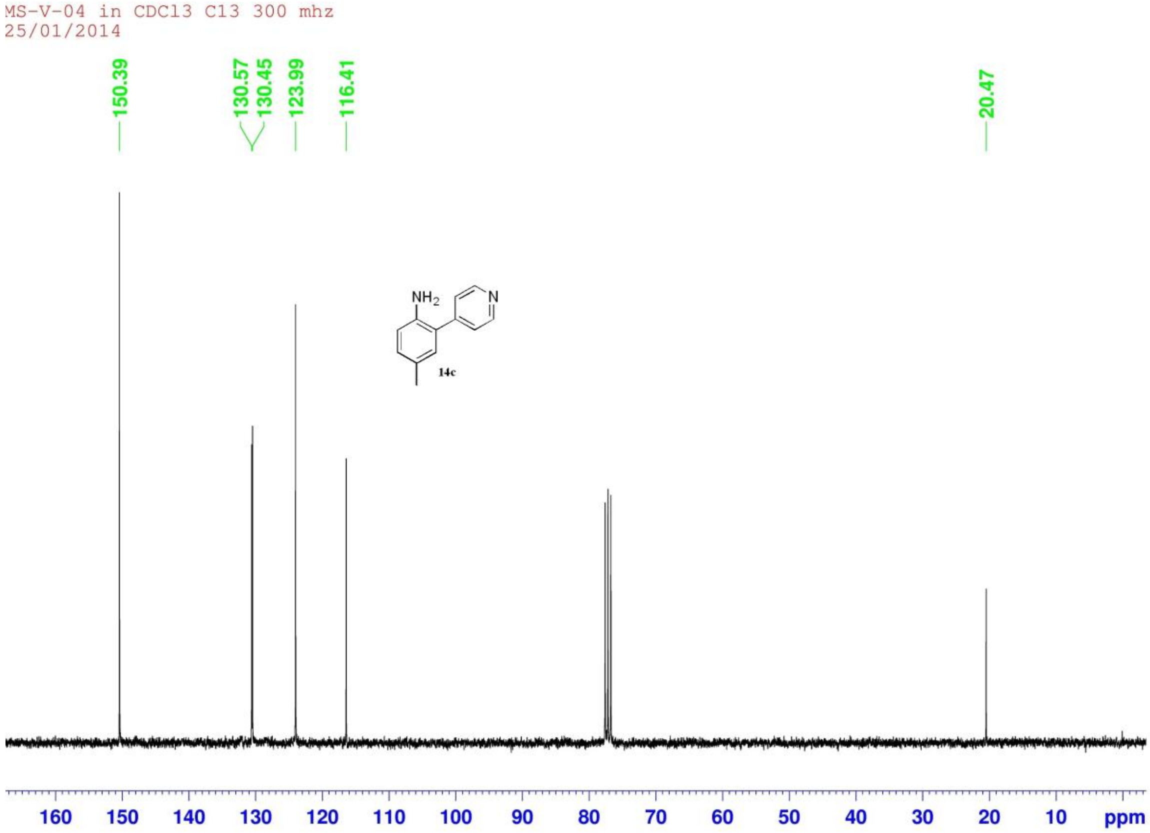

2-(pyridin-3-yl)aniline (14d)

HRMS (ES+)

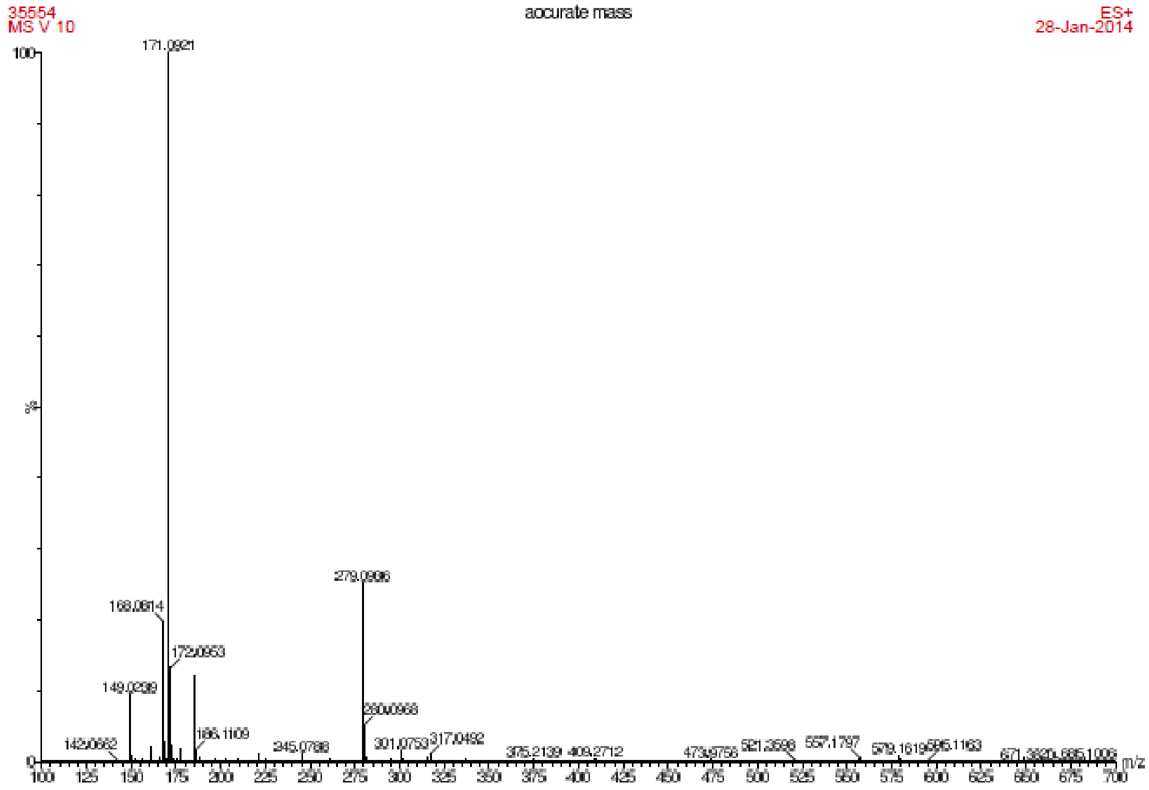

^{1}H NMR (300 MHz, $CDCl_3$)

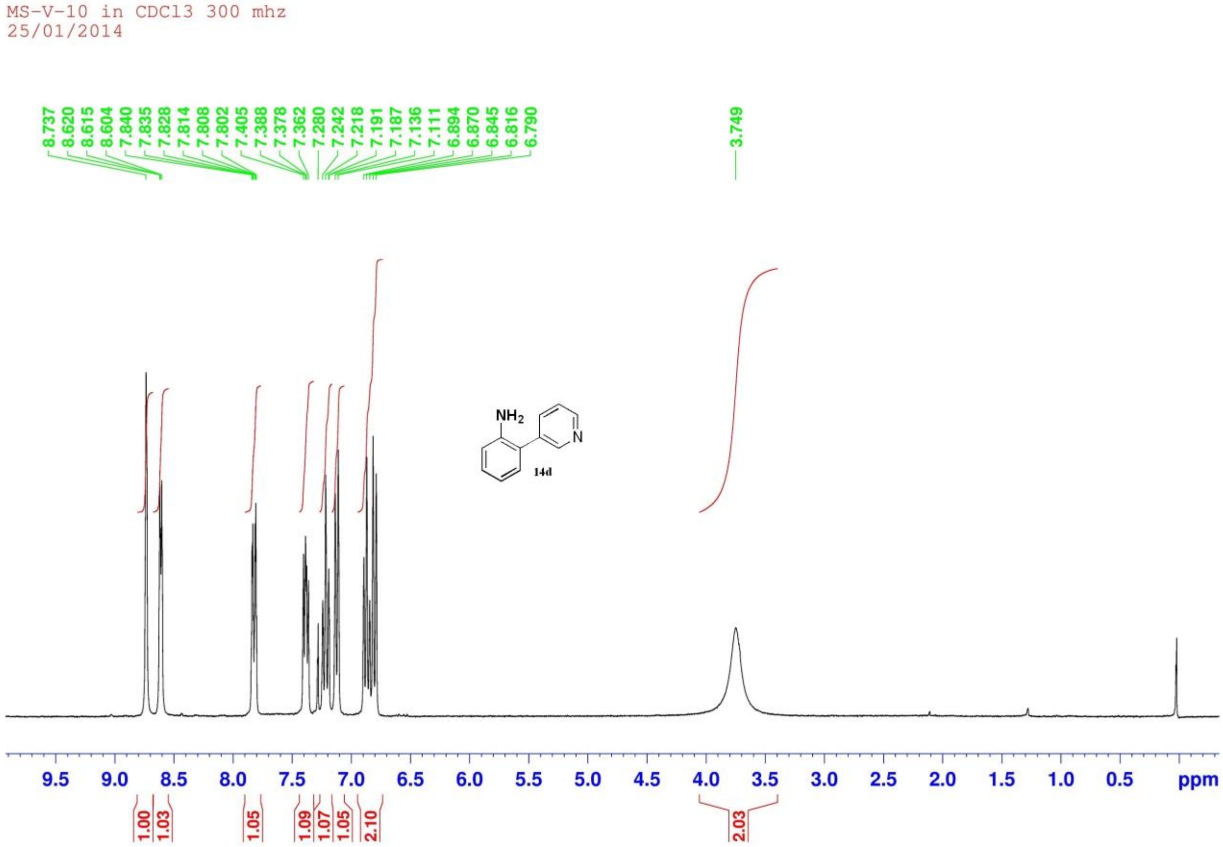

^{13}C NMR (75 MHz, $CDCl_3$)

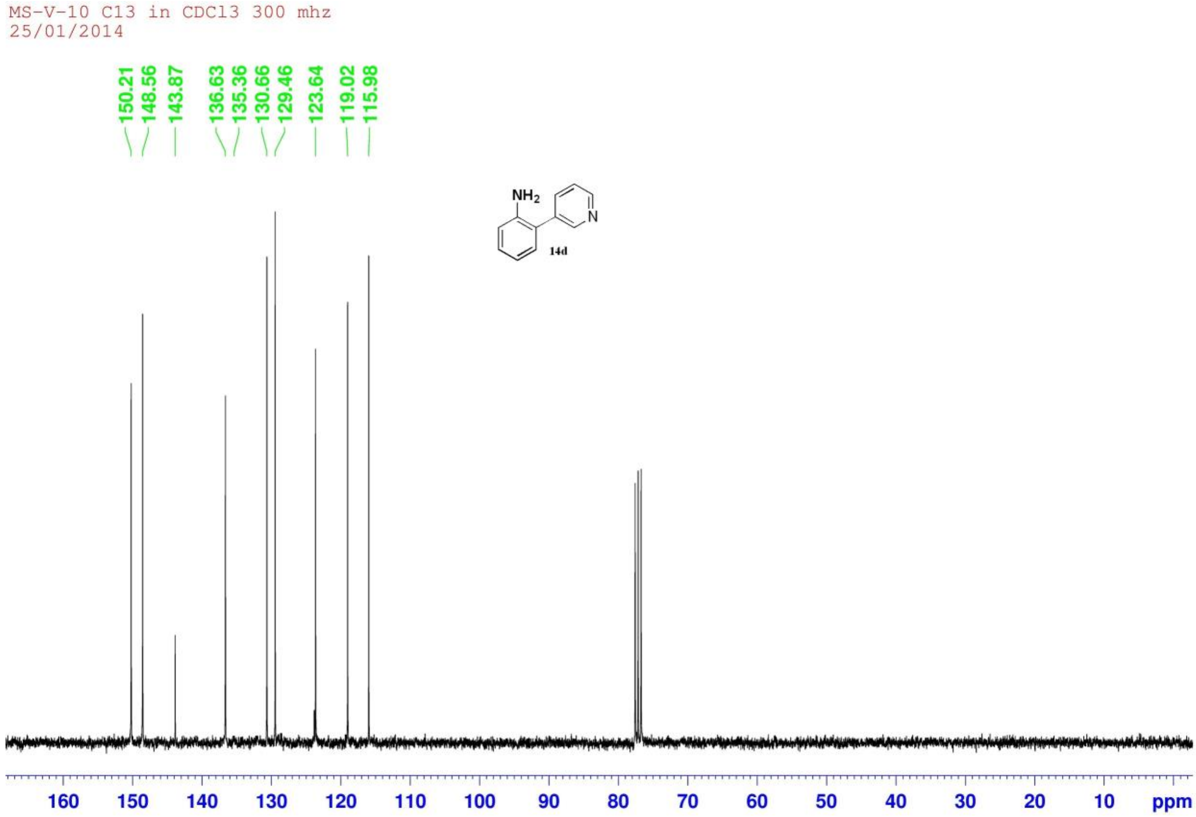

2-(pyridin-4-yl)aniline (14e)

HRMS (ES+)

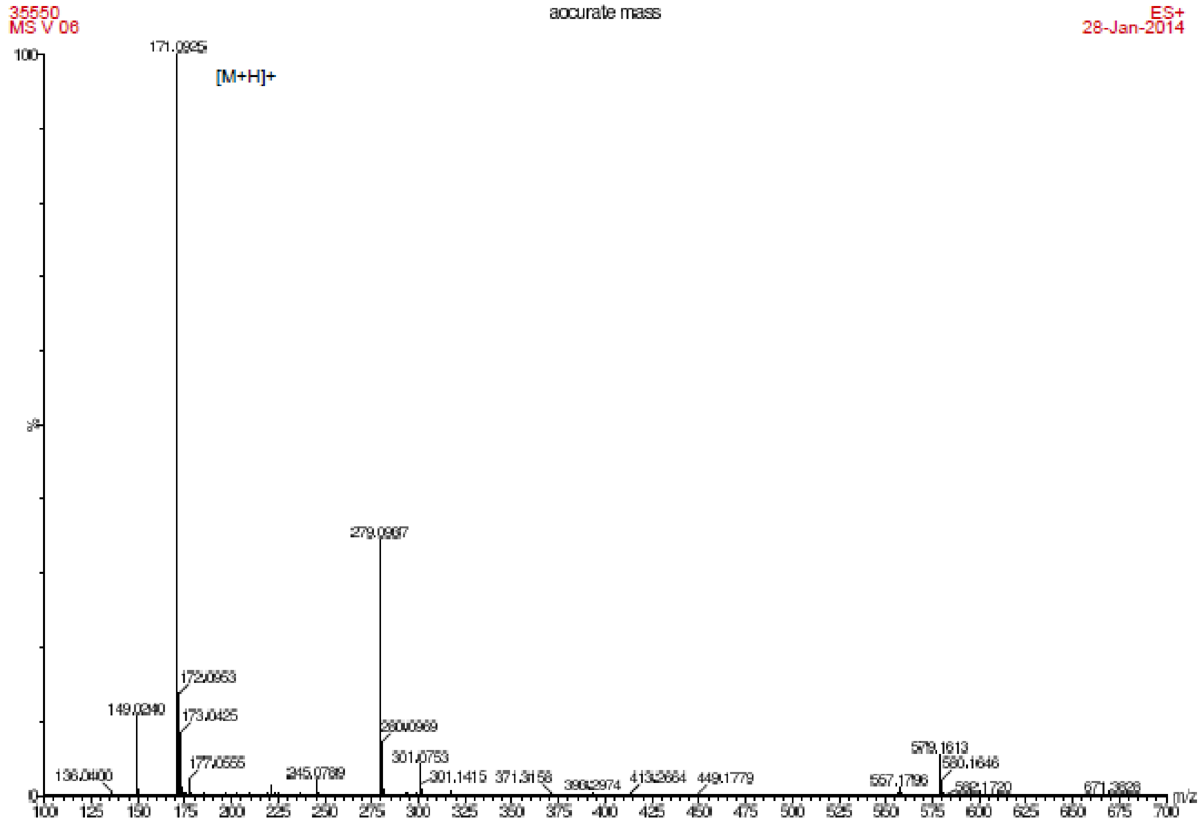

^{1}H NMR (300 MHz, $CDCl_3$)

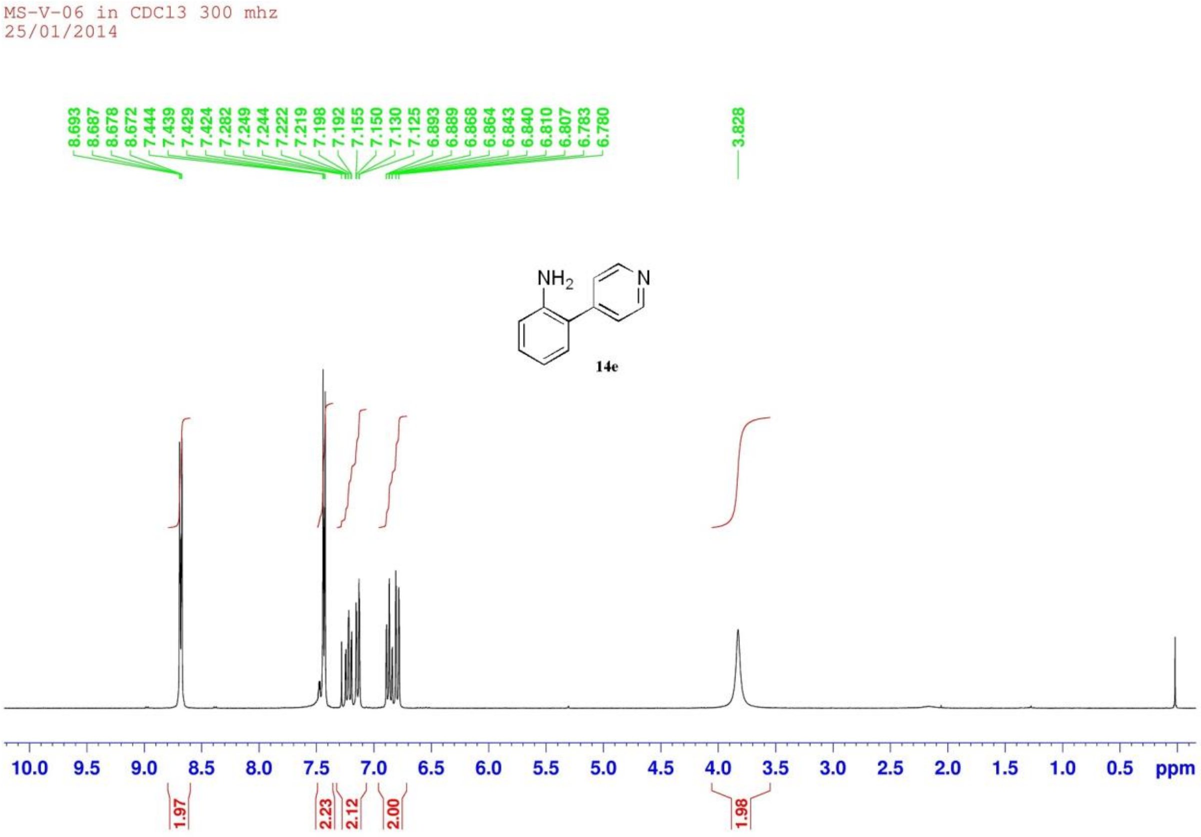

^{13}C NMR (75 MHz, $CDCl_3$)

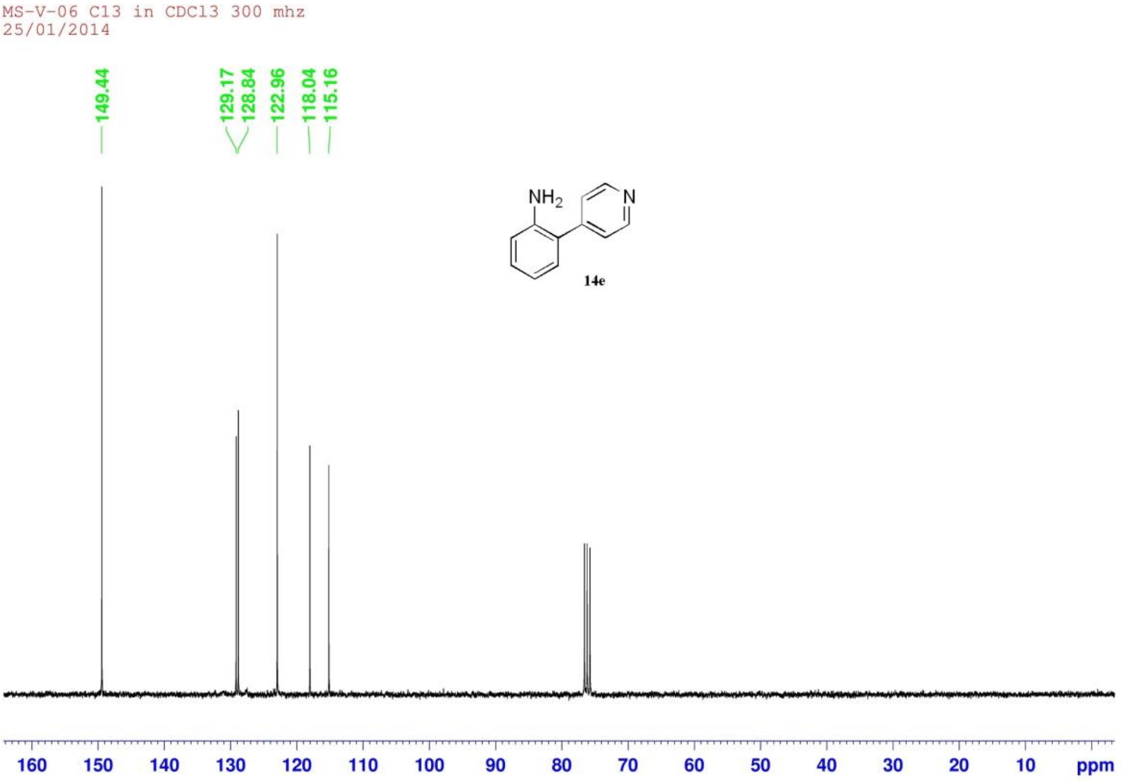

(4-(pyridin-3-yl)phenyl)methanamine (14f)

HRMS (ES+)

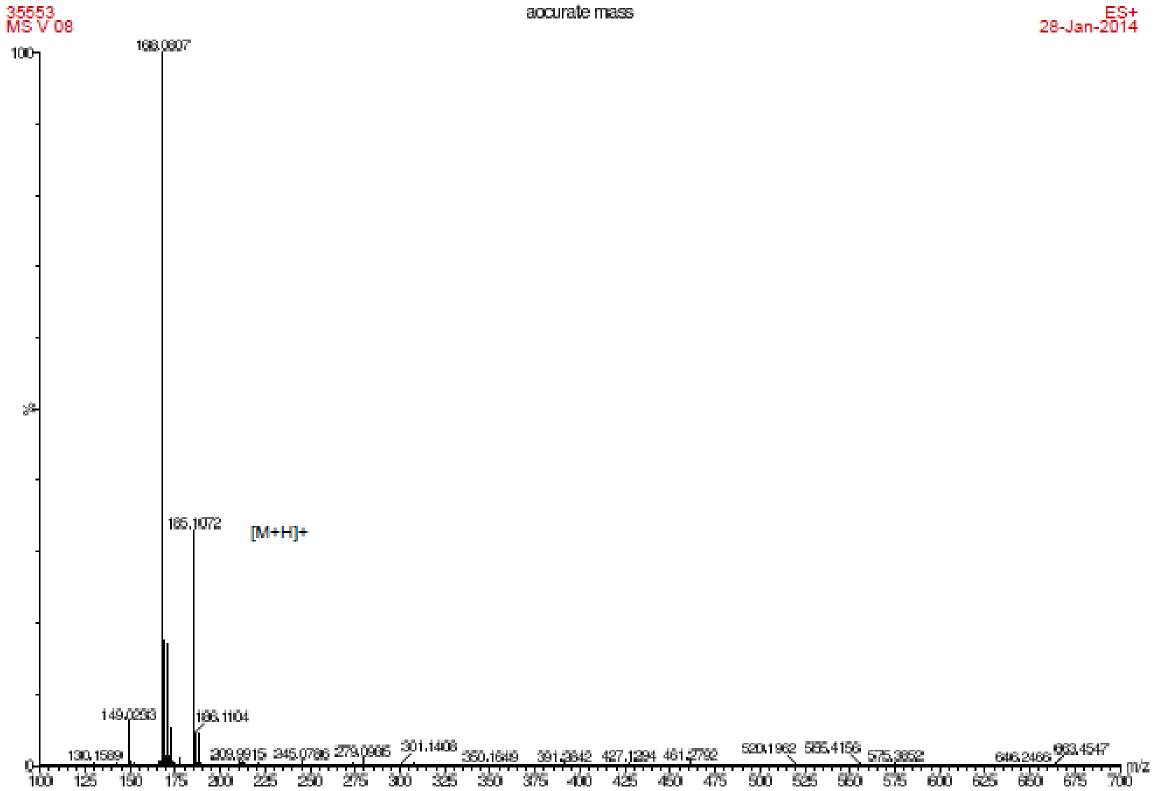

^{1}H NMR (300 MHz, $CDCl_3$)

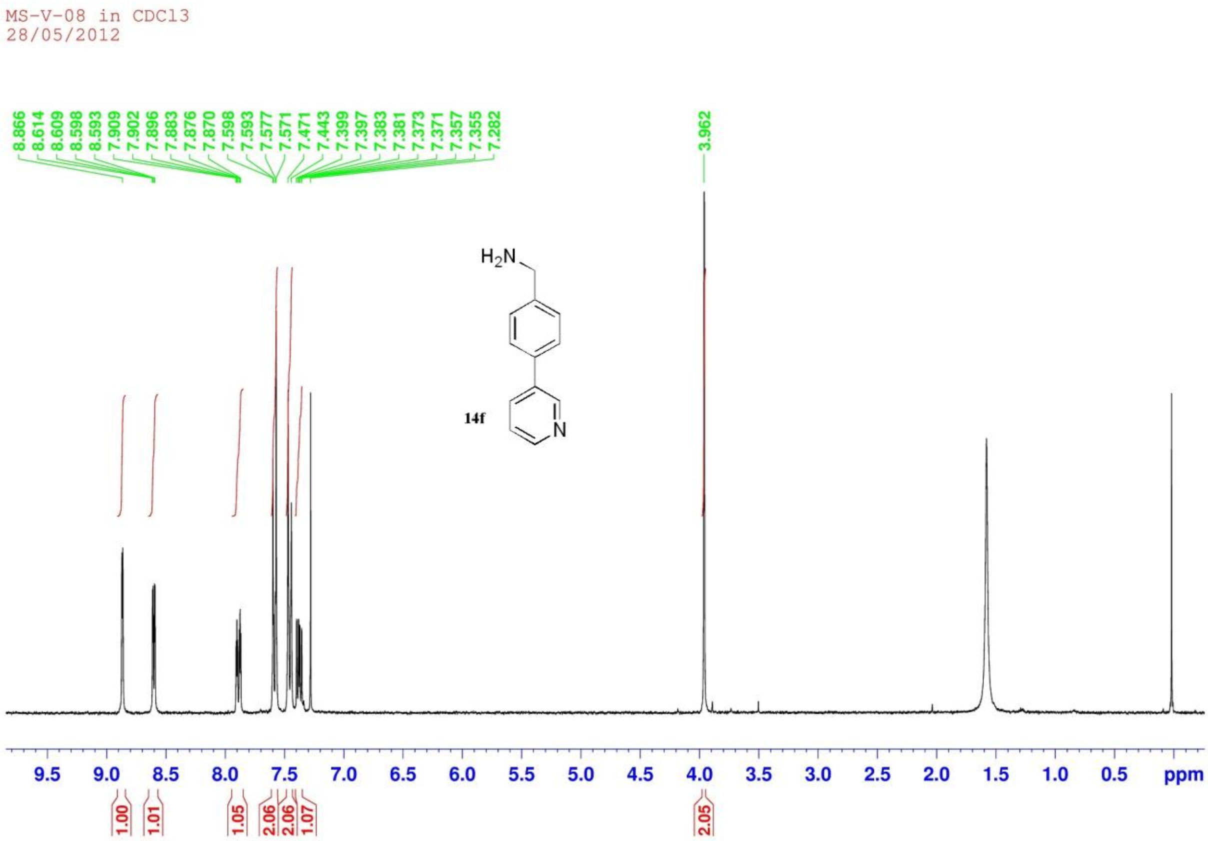

^{13}C NMR (75 MHz, $CDCl_3$)

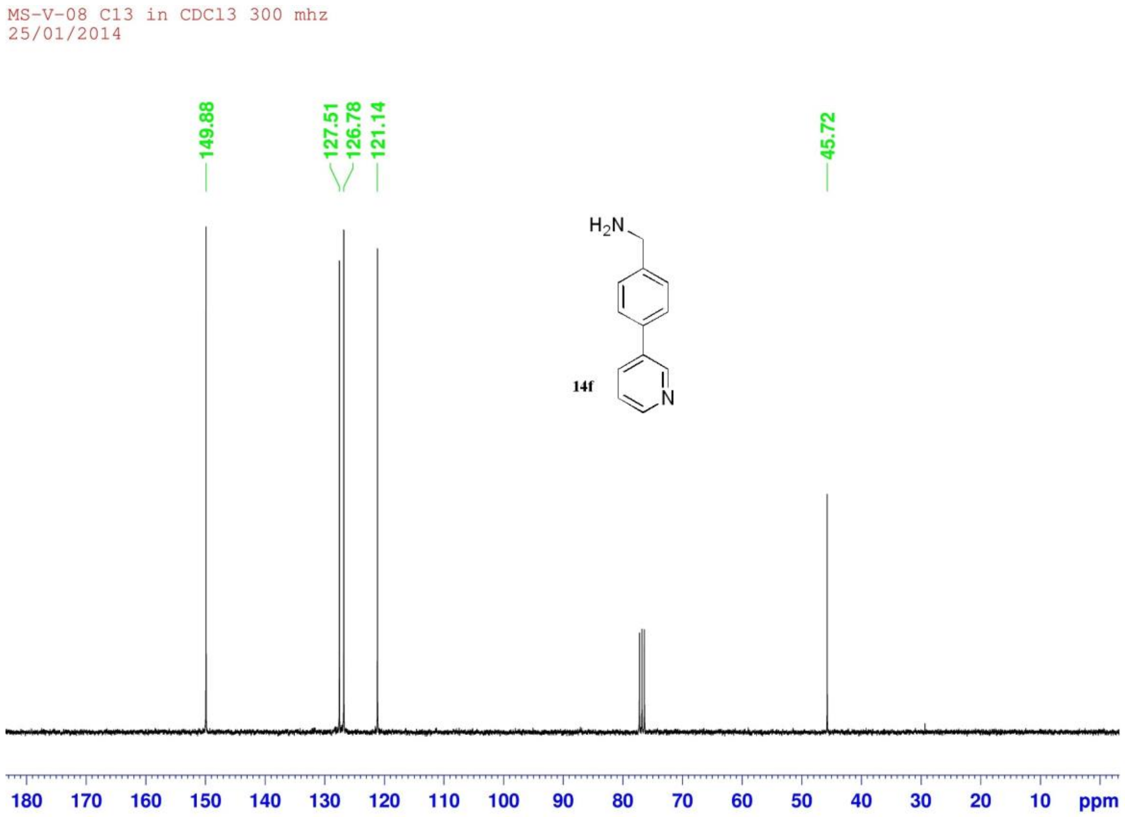

(4-(pyridin-4-yl)phenyl)methanamine (14g)

^{1}H NMR (500 MHz, $CDCl_3$)

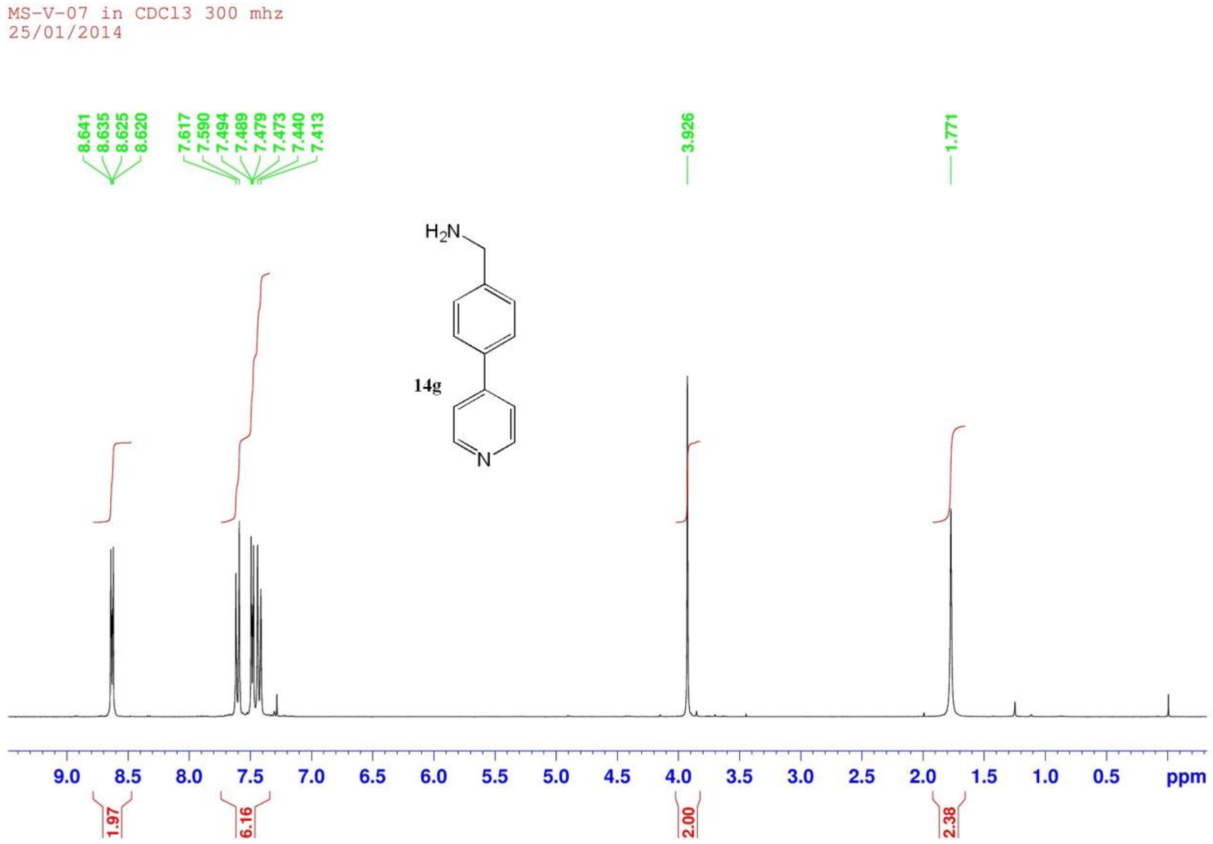

^{13}C NMR (75 MHz, $CDCl_3$)

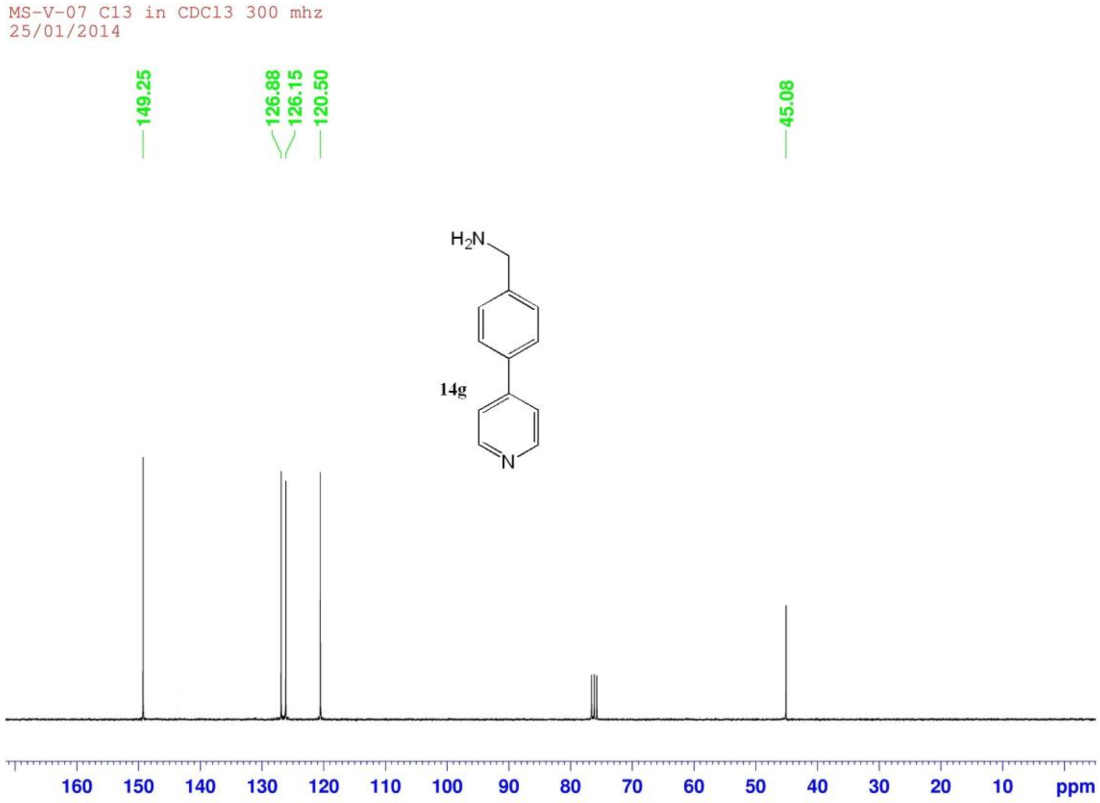

(2-(pyridin-4-yl)phenyl)methanamine (14h)

^{1}H NMR (300 MHz, $CDCl_3$)

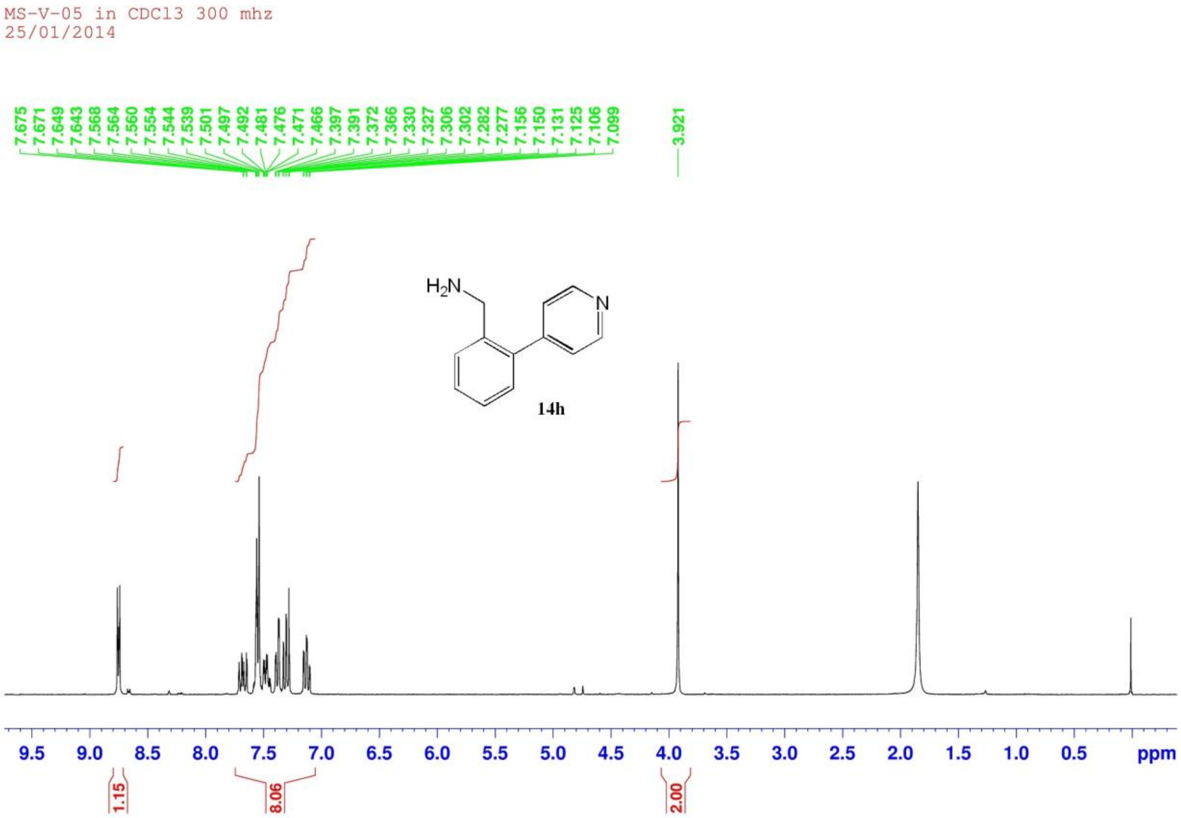

^{13}C NMR (75 MHz, $CDCl_3$)

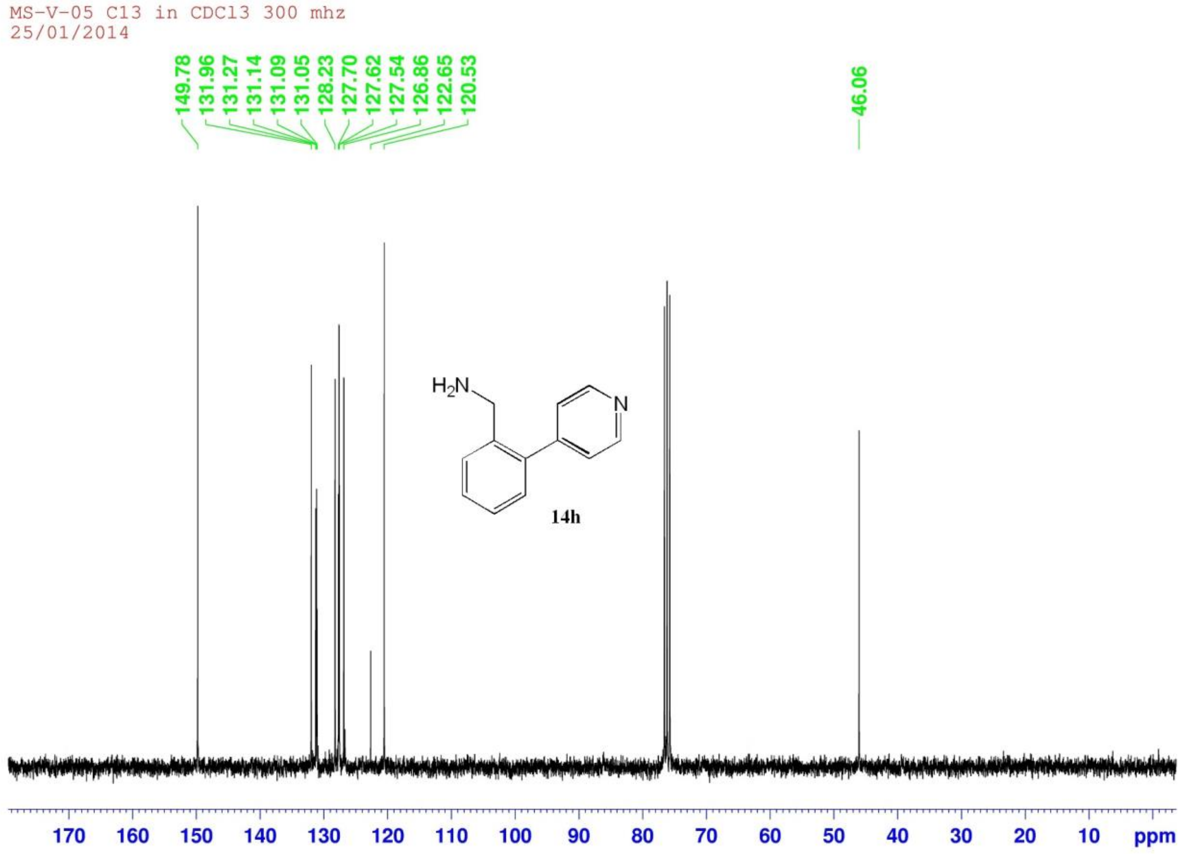

4-methyl-2-(thiophen-3-yl)aniline (14i)

HRMS (ES+)

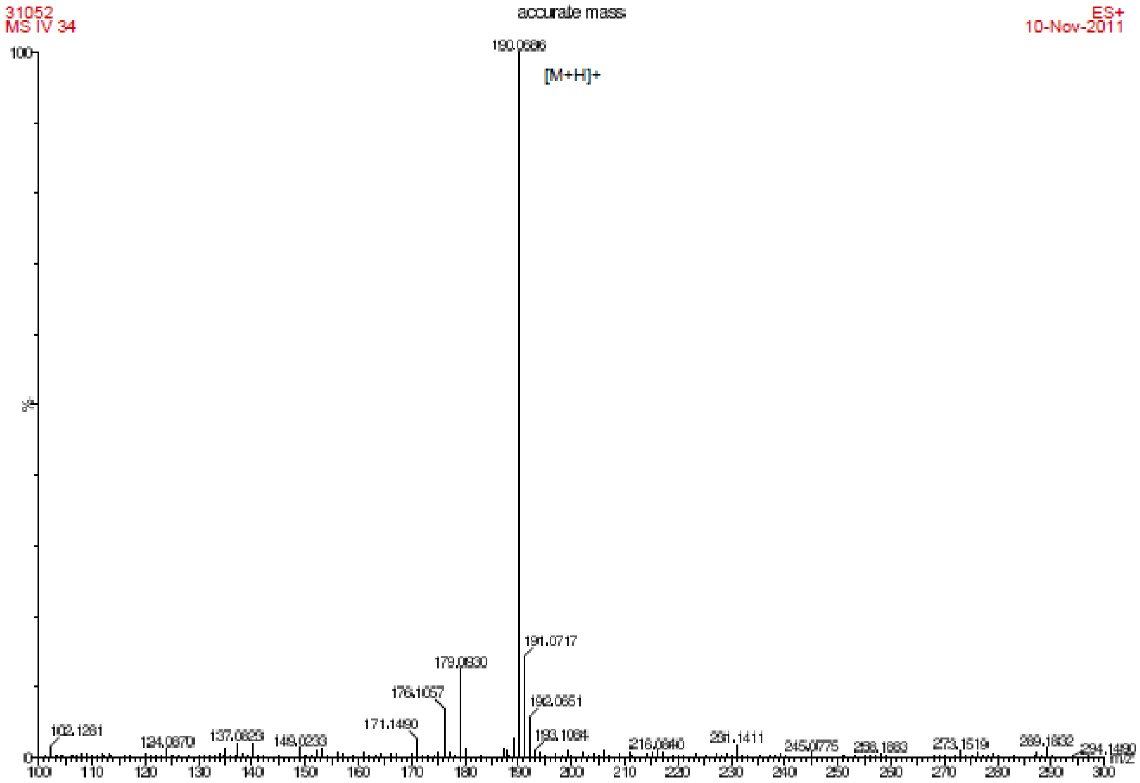

^{1}H NMR (300 MHz, $CDCl_3$)

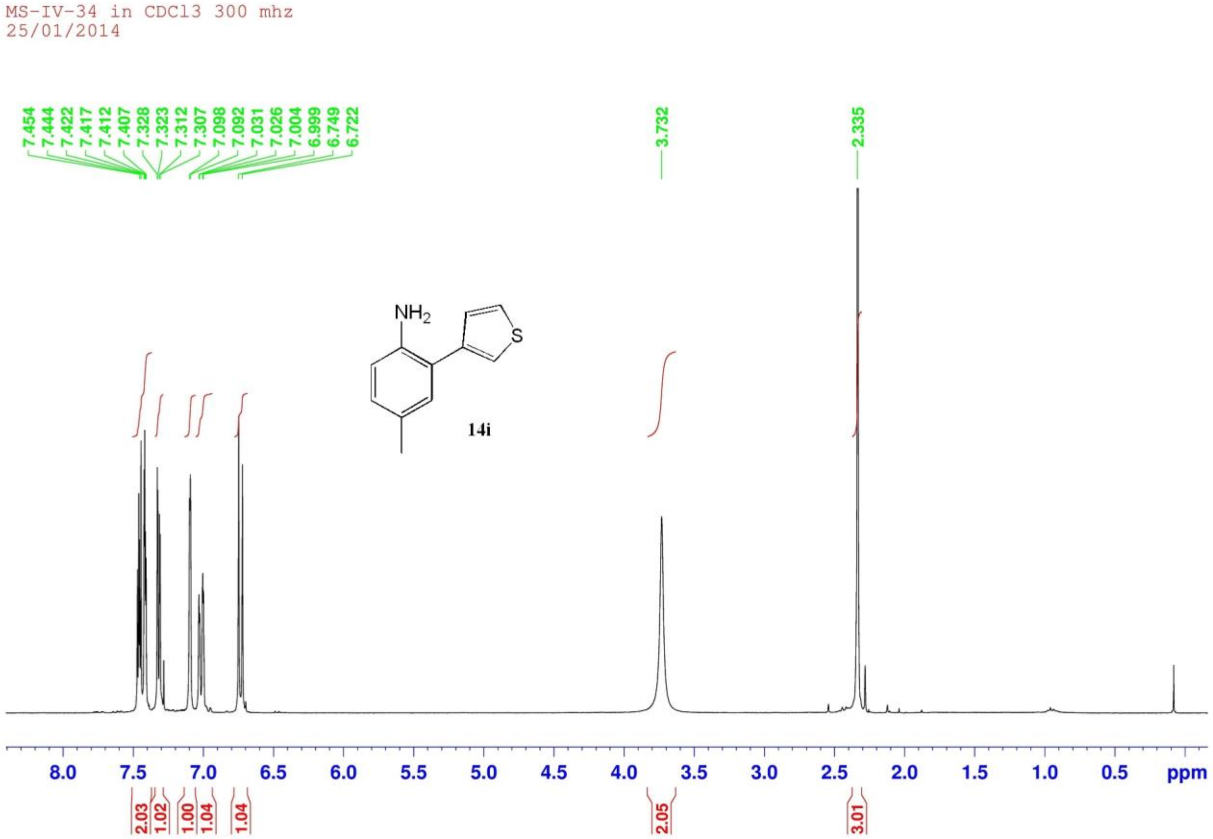

^{13}C NMR (75 MHz, $CDCl_3$)

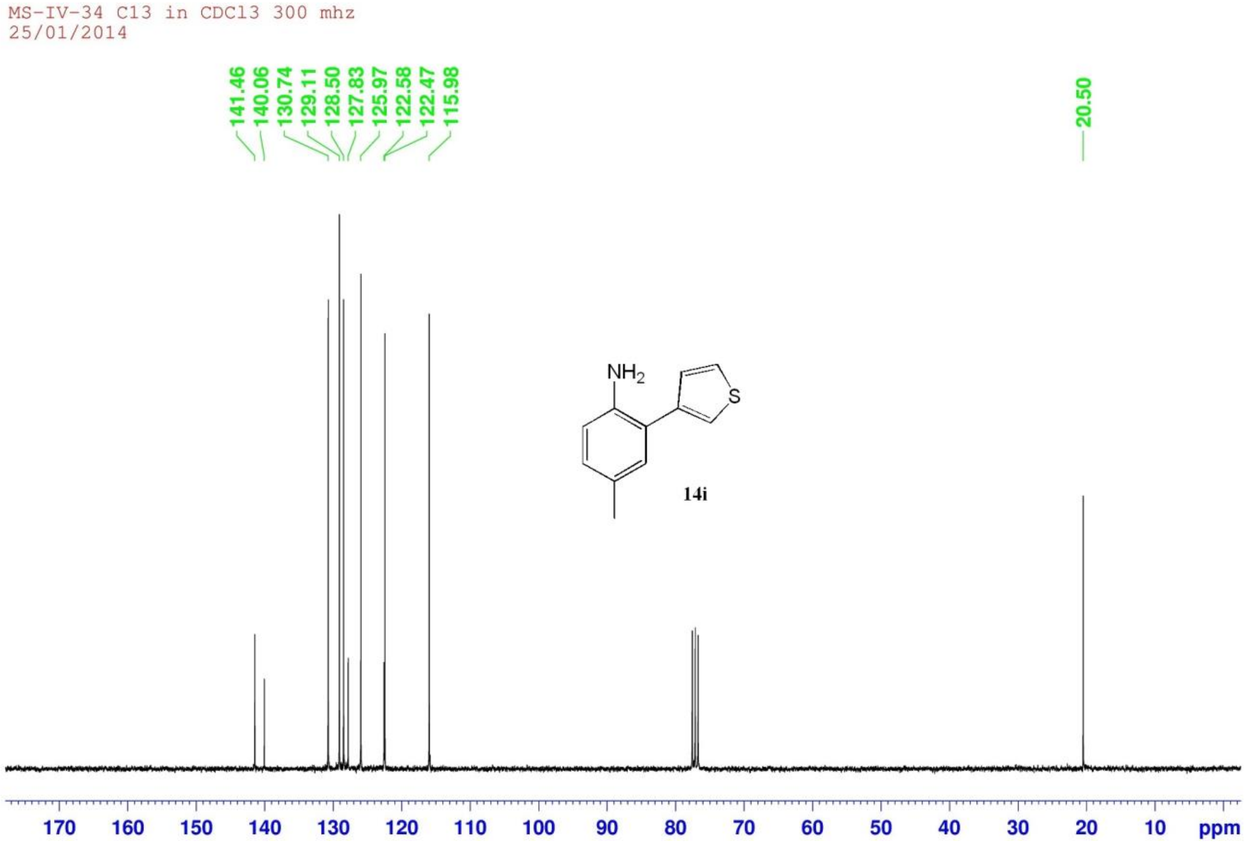

(4-(thiophen-3-yl)phenyl)methanamine (14j)

^{1}H NMR (300 MHz, $CDCl_3$)

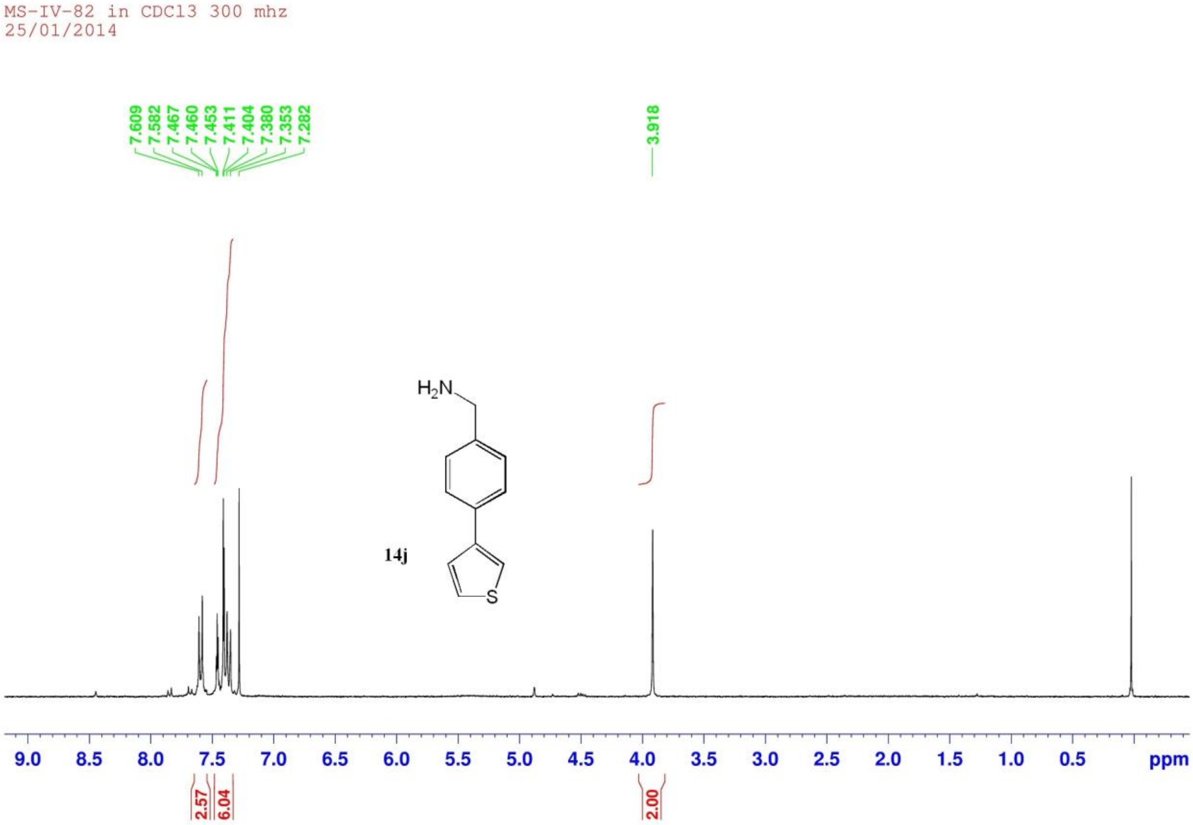

^{13}C NMR (75 MHz, $CDCl_3$)

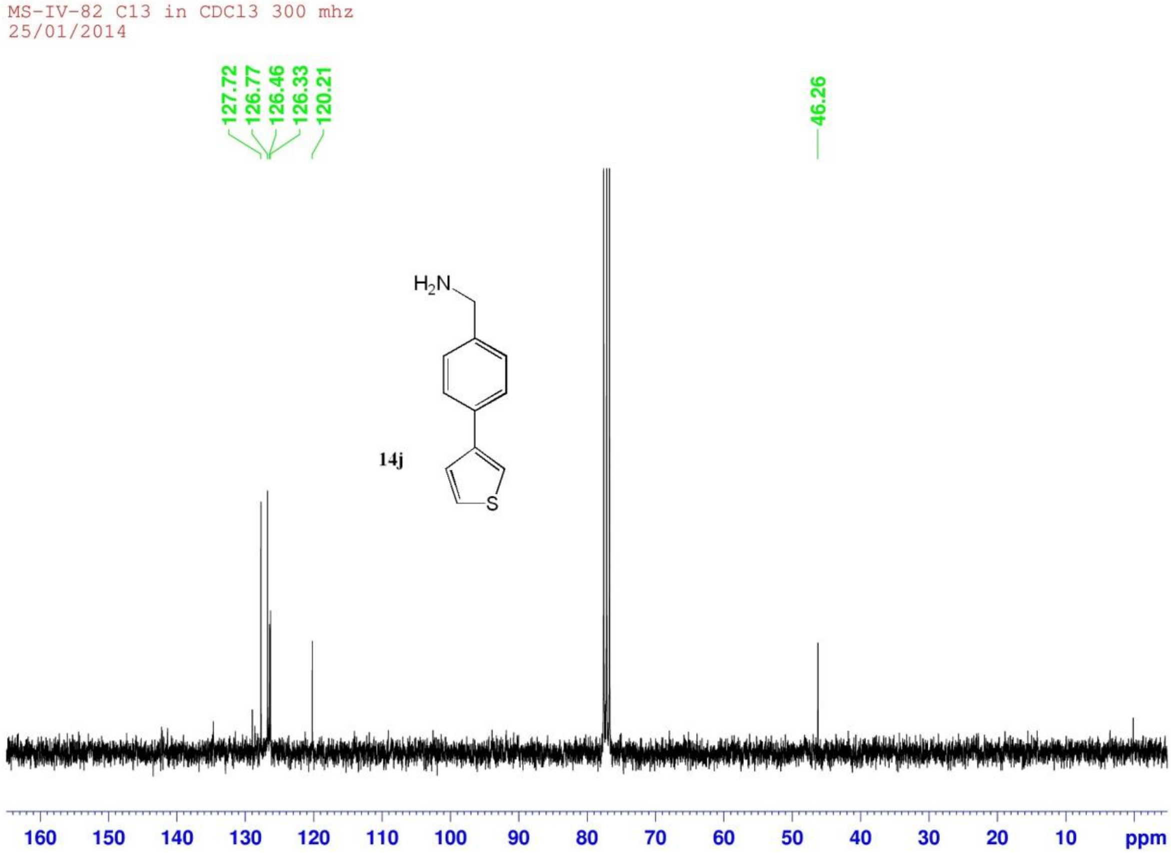

2-(thiophen-3-yl)phenyl)methanamine (4.14k)

^{1}H NMR (300 MHz, $CDCl_3$)

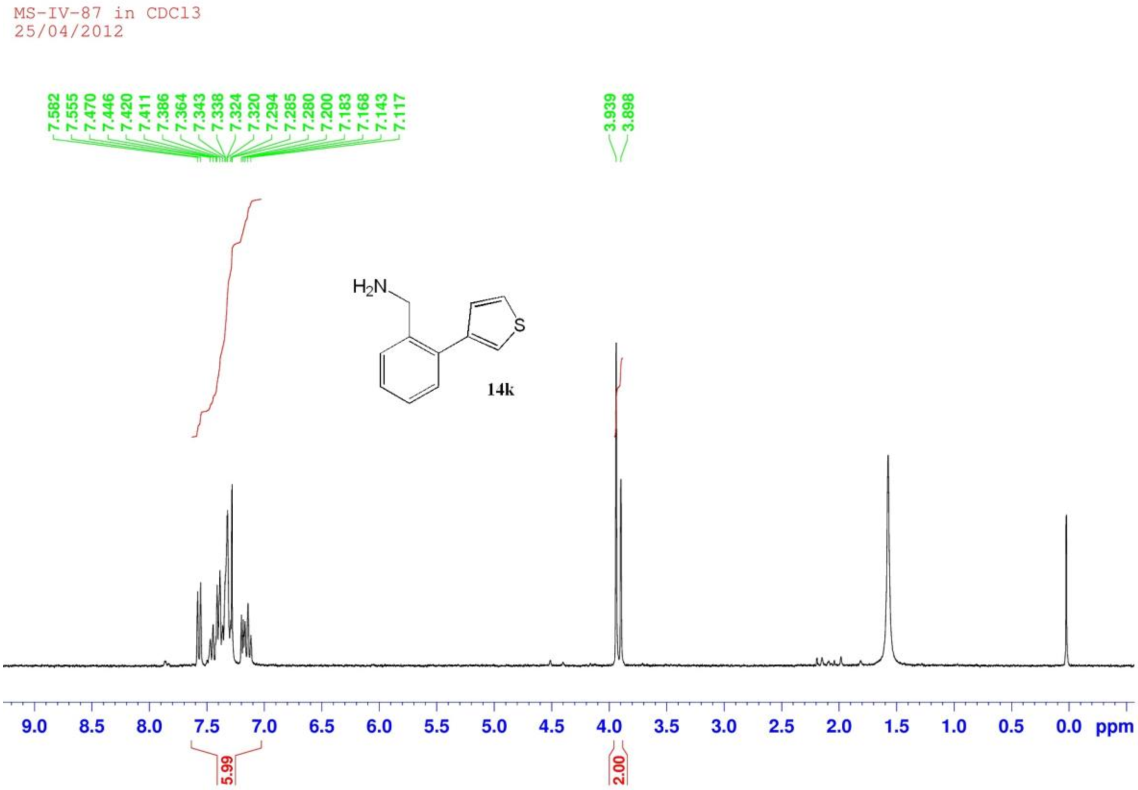

2-(furan-2-yl)-4-methylaniline (14l)

HRMS (ES+)

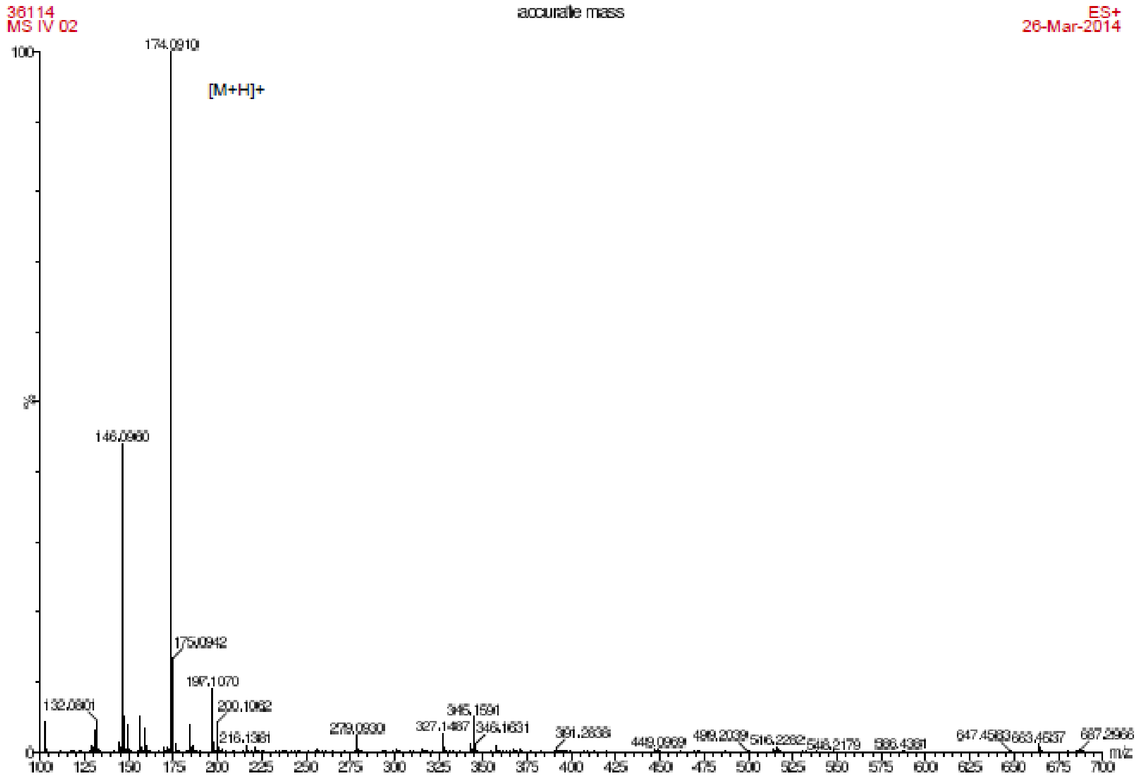

^1H NMR (300 MHz, $CDCl_3$)

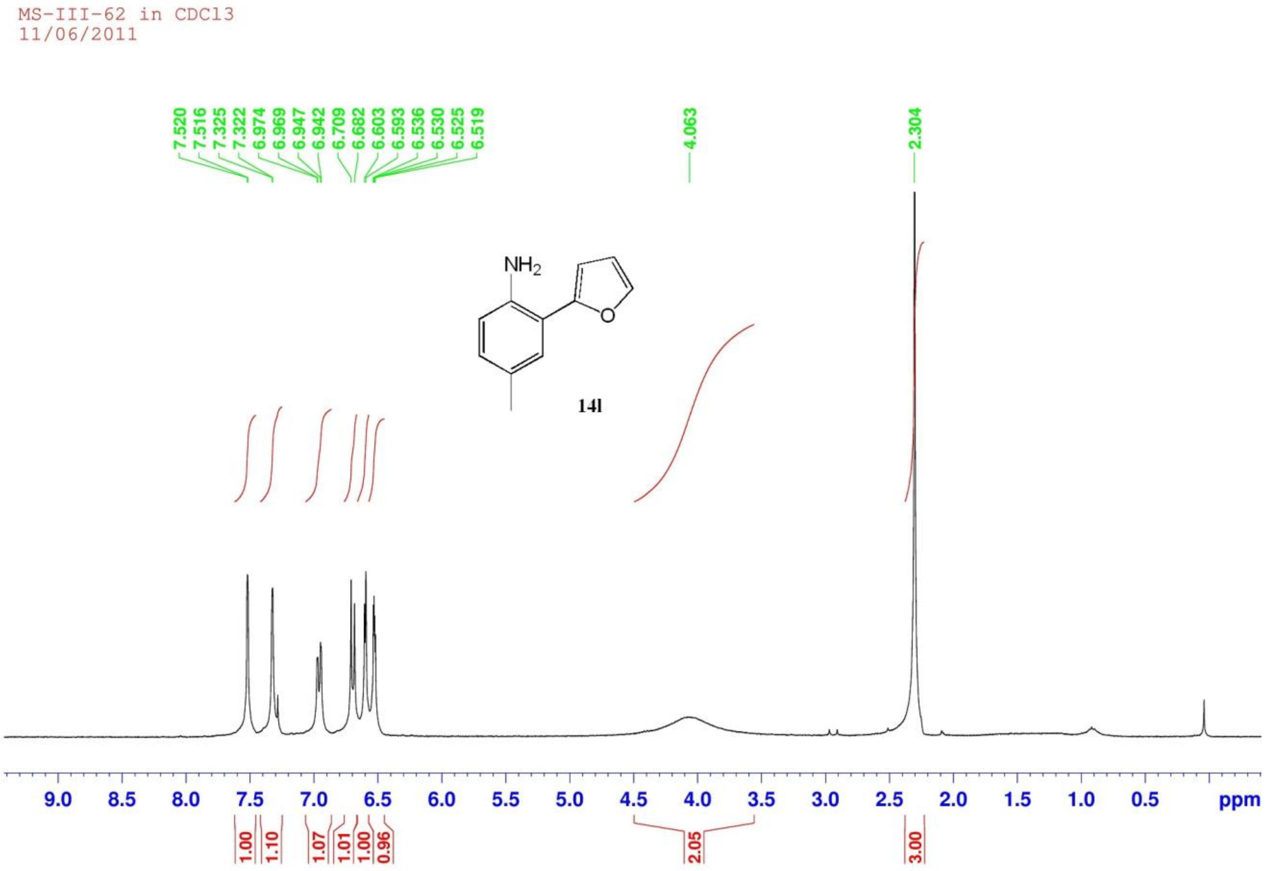

^{13}C NMR (75 MHz, $CDCl_3$)

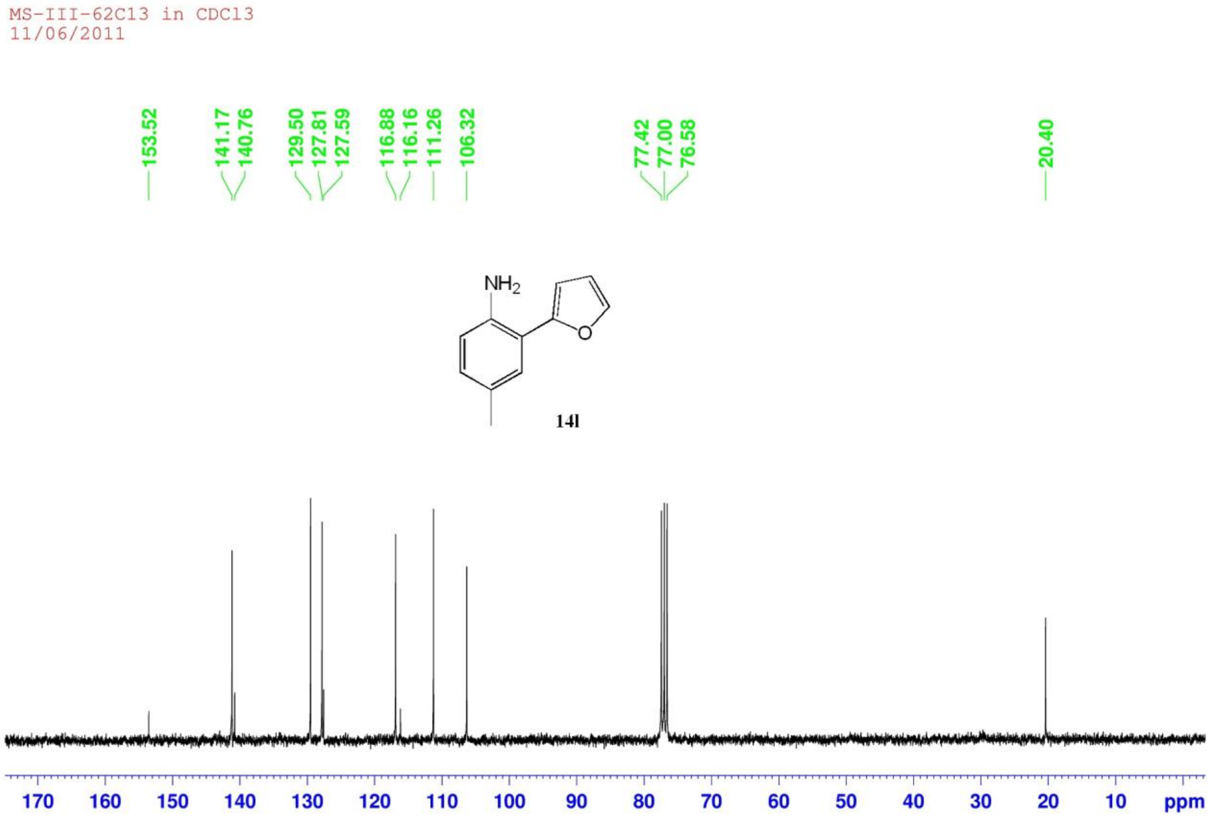

5-methyl-3-(5-methylthiophen-2-yl)pyridin-2-amine (14m)

^{1}H NMR (300 MHz, $CDCl_3$)

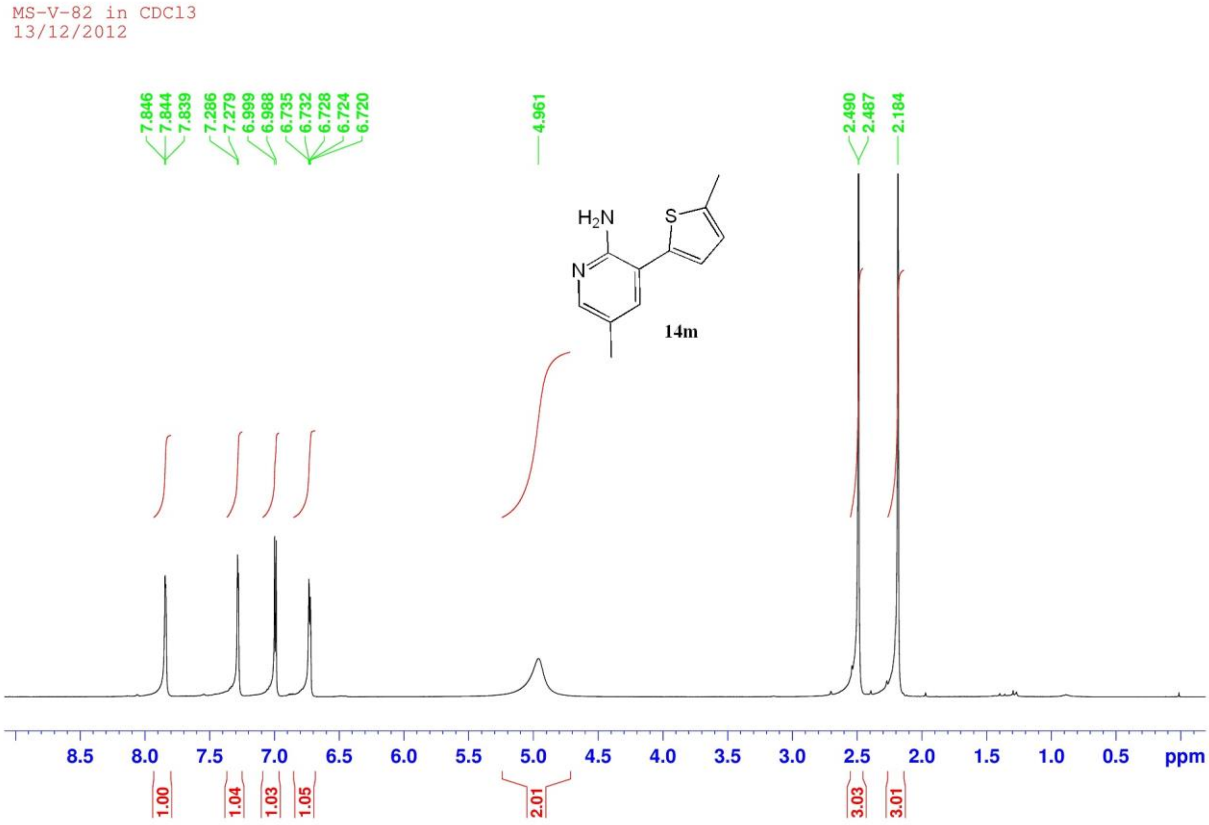

^{13}C NMR (75 MHz, $CDCl_3$)

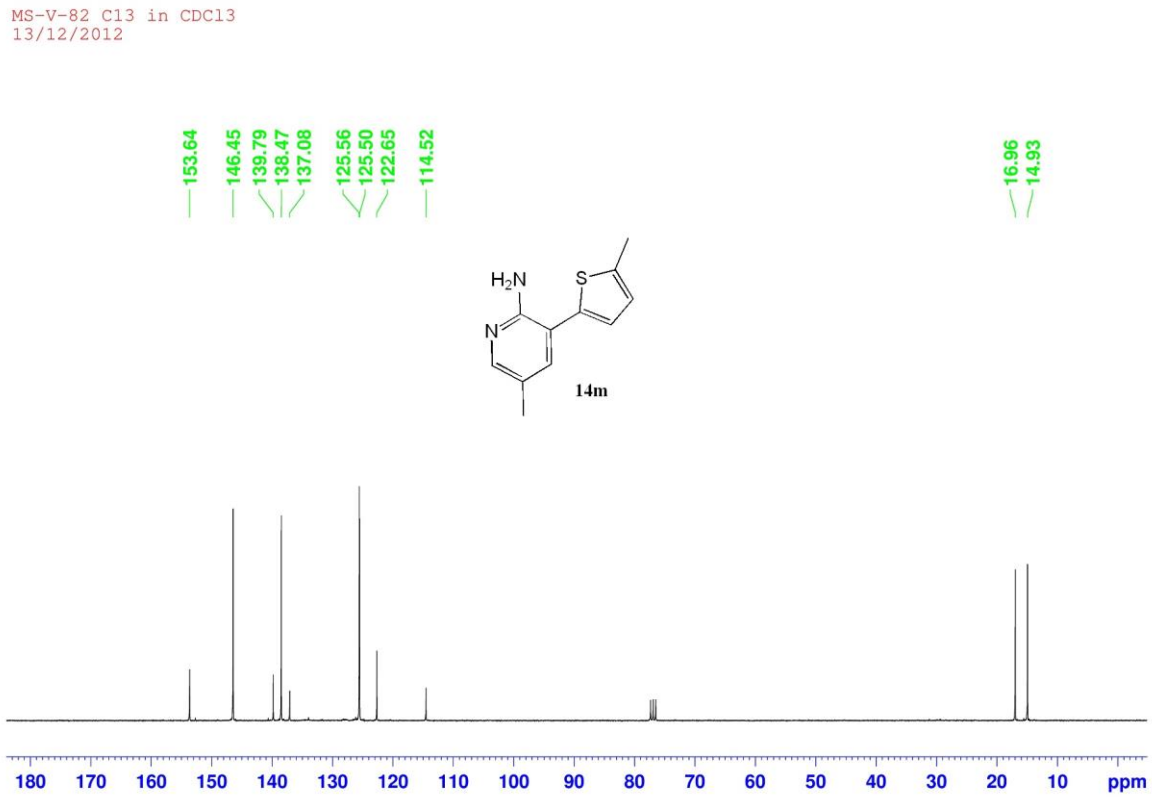

N^5-(4-fluorophenyl)-N^4-(2-(3-methyl-1H-pyrazol-5-yl)phenyl)-1H-imidazole-4,5-dicarboxamide (15b)

HRMS (ES+)

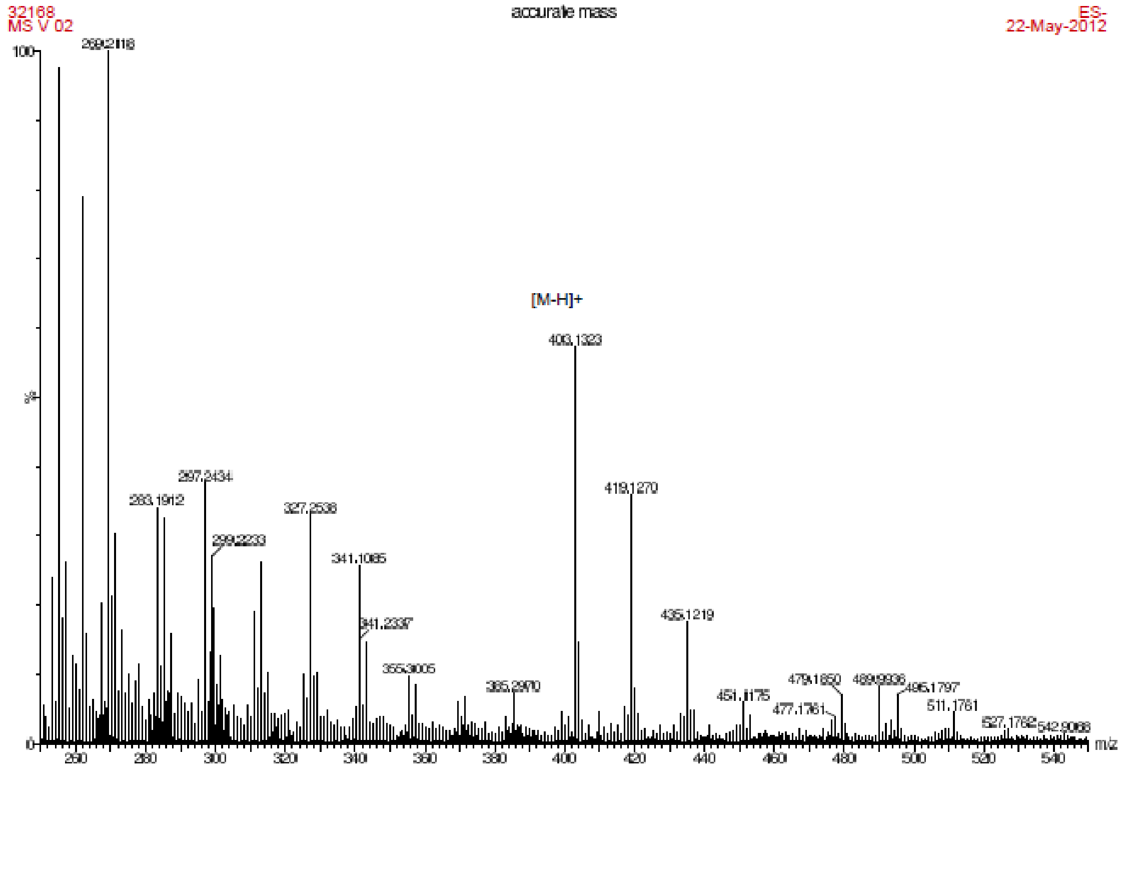

^{1}H NMR (300 MHz, DMSO)

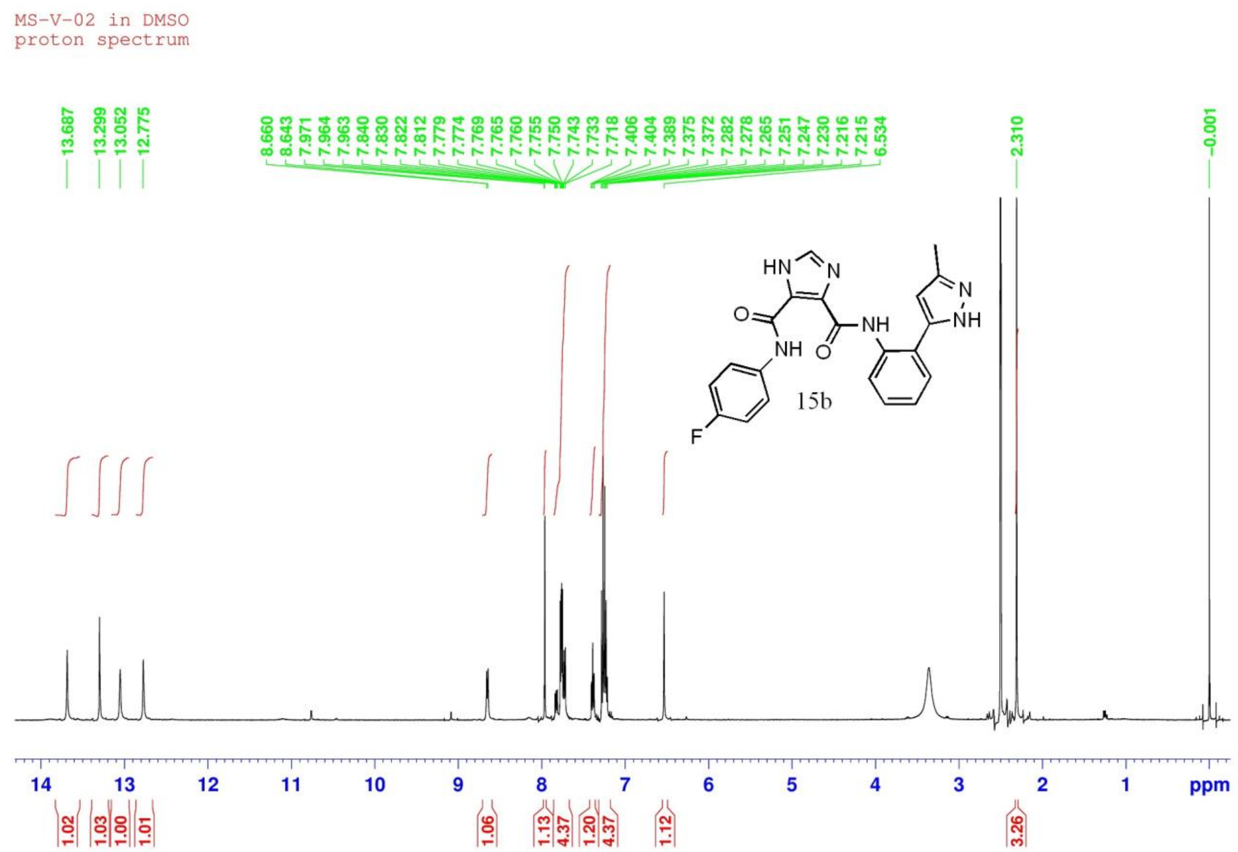

^{13}C NMR (75 MHz, DMSO)

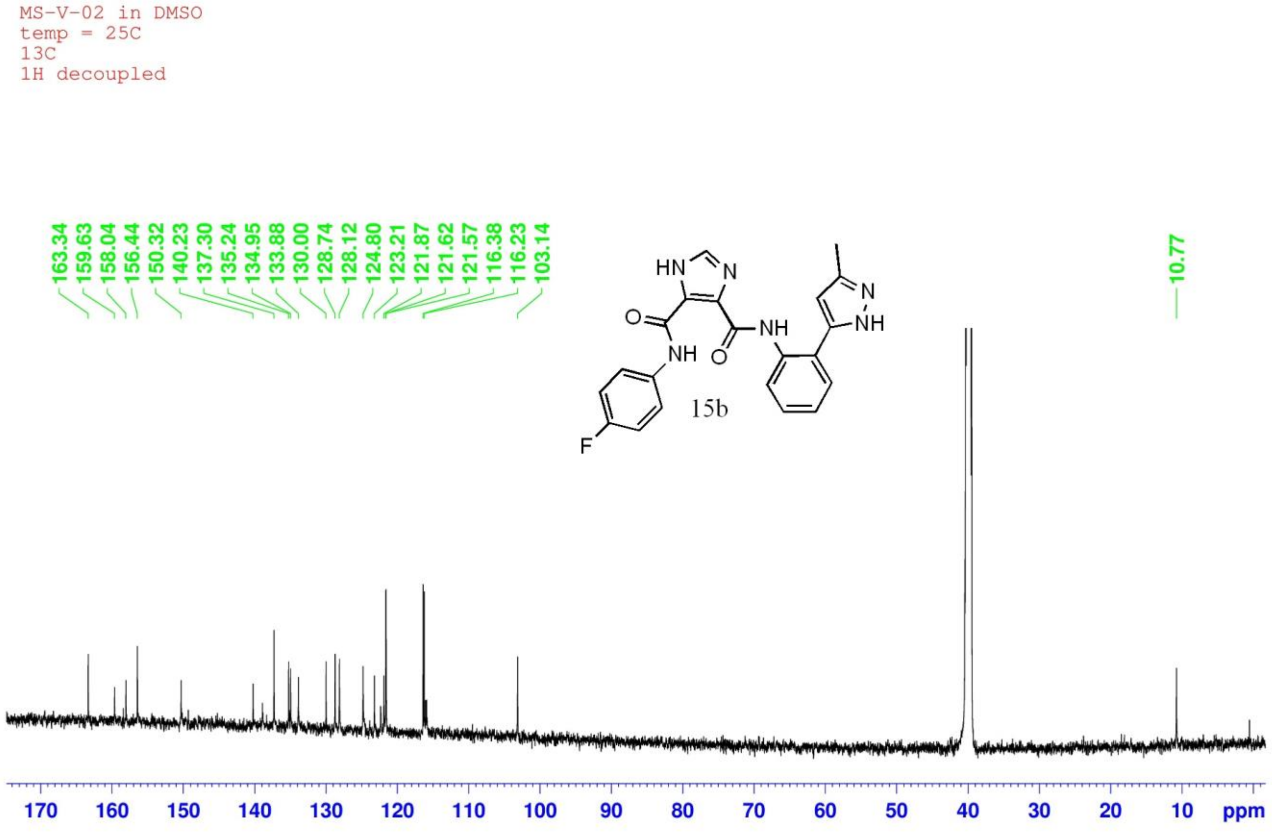

_N_[5]-(4-fluorophenyl)-_N_[4]-(2-(pyridin-4-yl)benzyl)-1H-imidazole-4,5-dicarboxamide (15c)

HRMS (ES+)

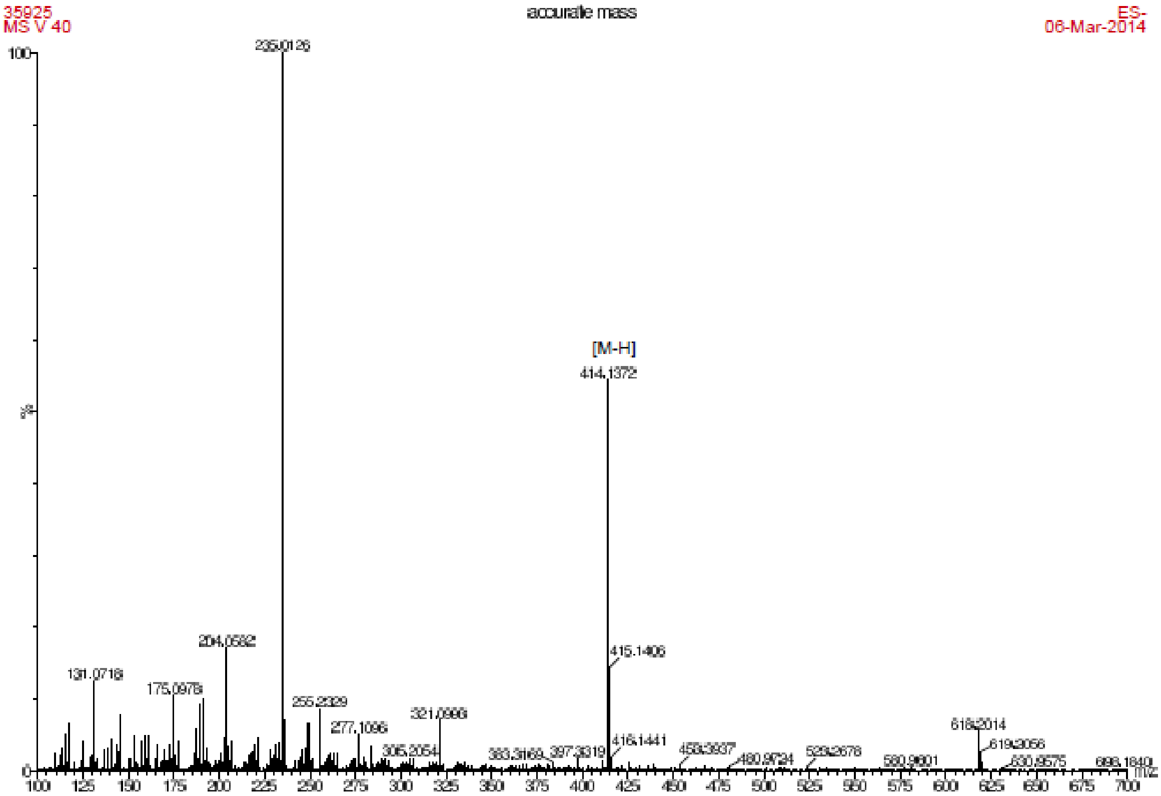

^{1}H NMR (300 MHz, DMSO)

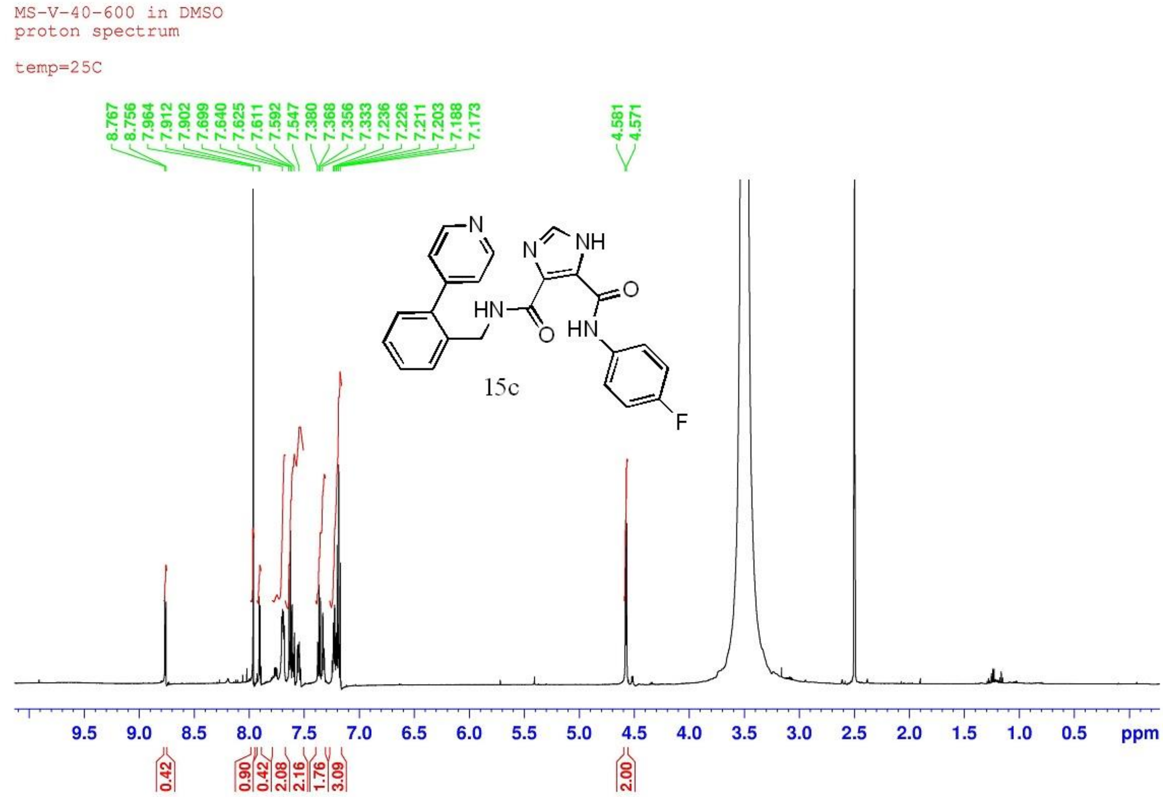

^{13}C NMR (75 MHz, DMSO)

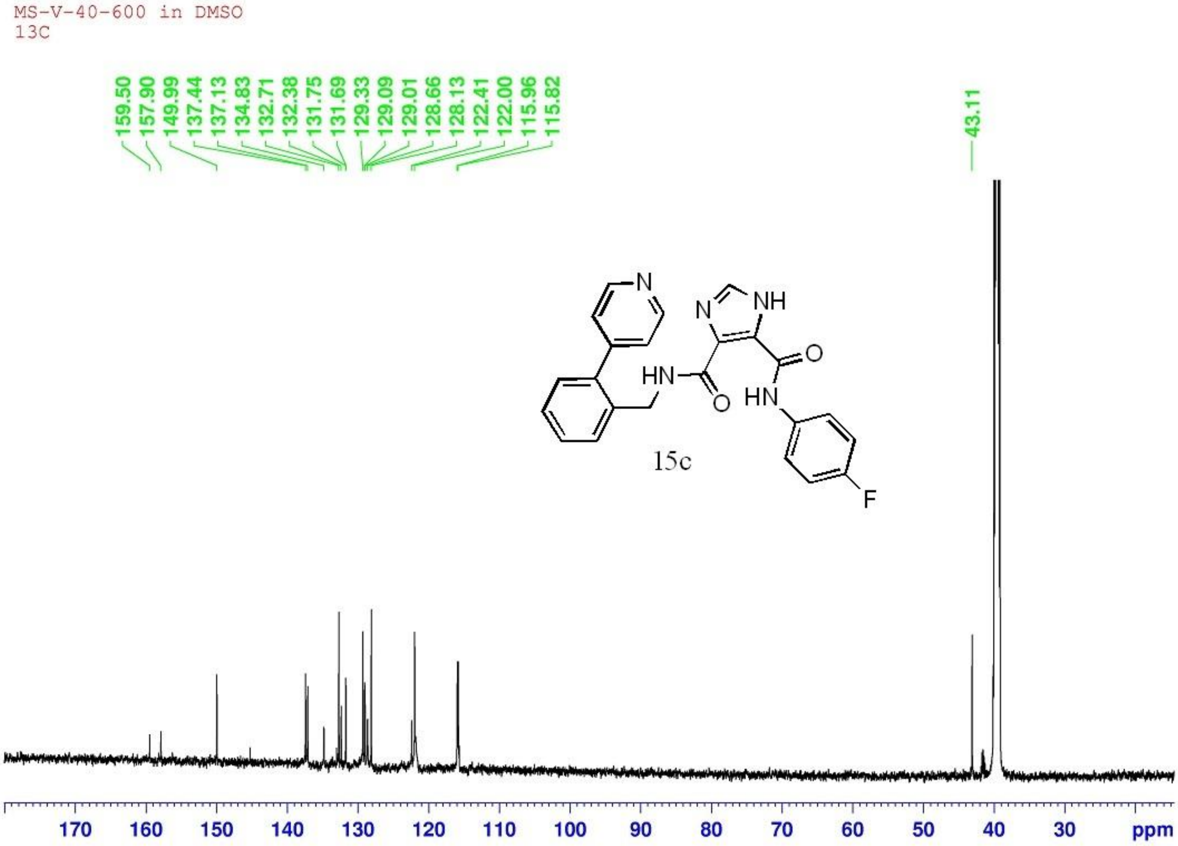

N^5-(4-fluorophenyl)-N^4-(2-(thiophen-3-yl)benzyl)-1H-imidazole-4,5-dicarbox-amide (15d)

HRMS (ES+)

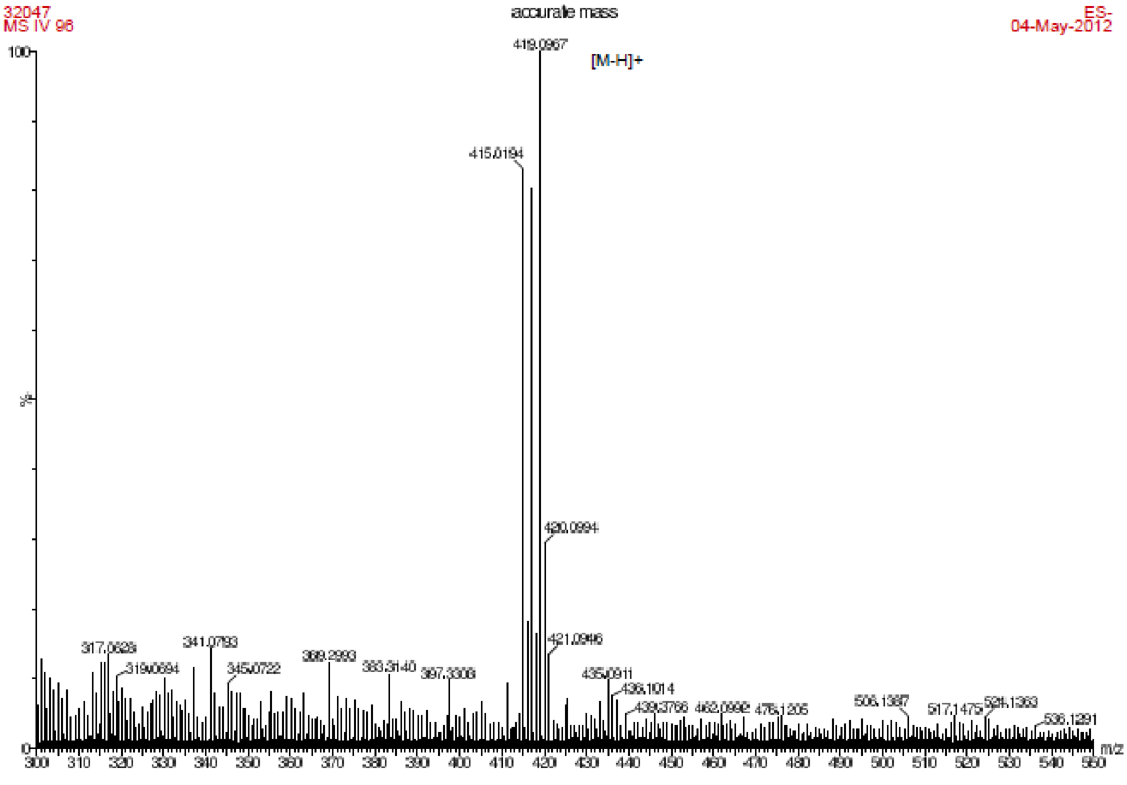

^{1}H NMR (300 MHz, DMSO)

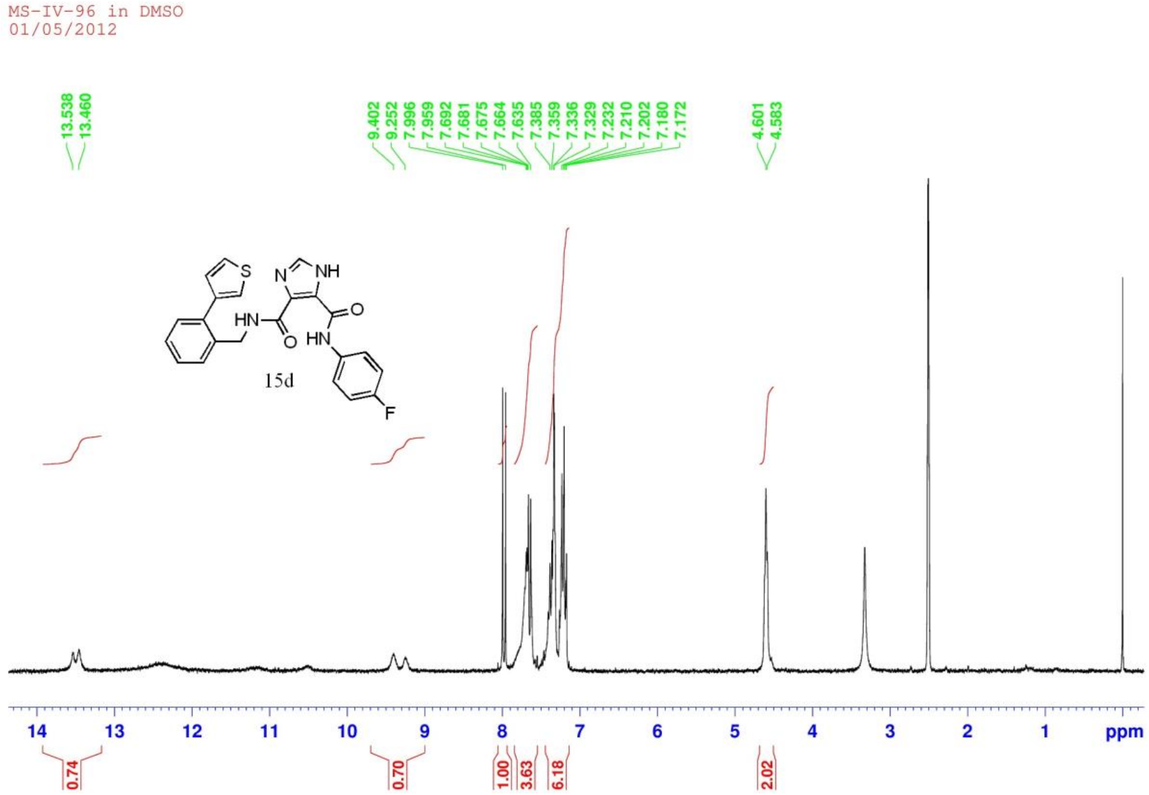

^{13}C NMR (75 MHz, DMSO)

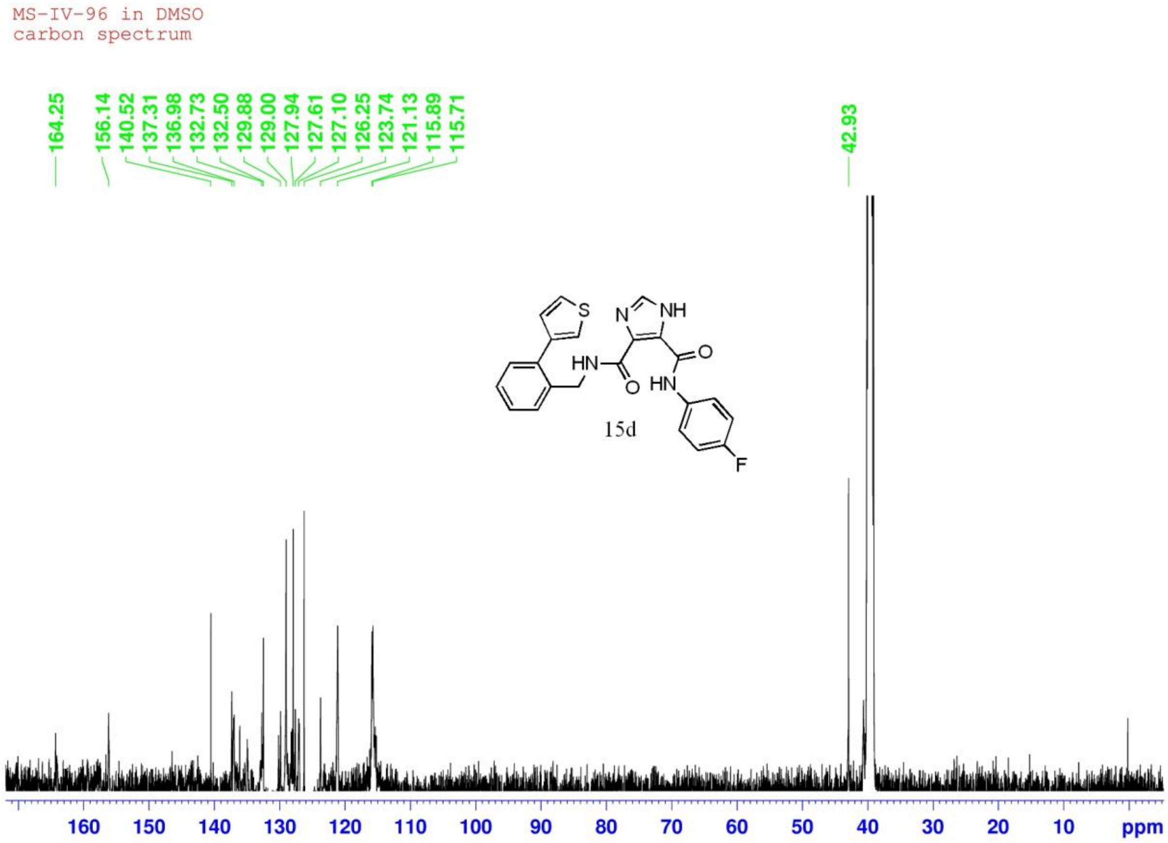

N^5-(4-fluorophenyl)-N^4-(4-(pyridin-3-yl)benzyl)-1H-imidazole-4,5-dicarboxamide (15e)

HRMS (ES+)

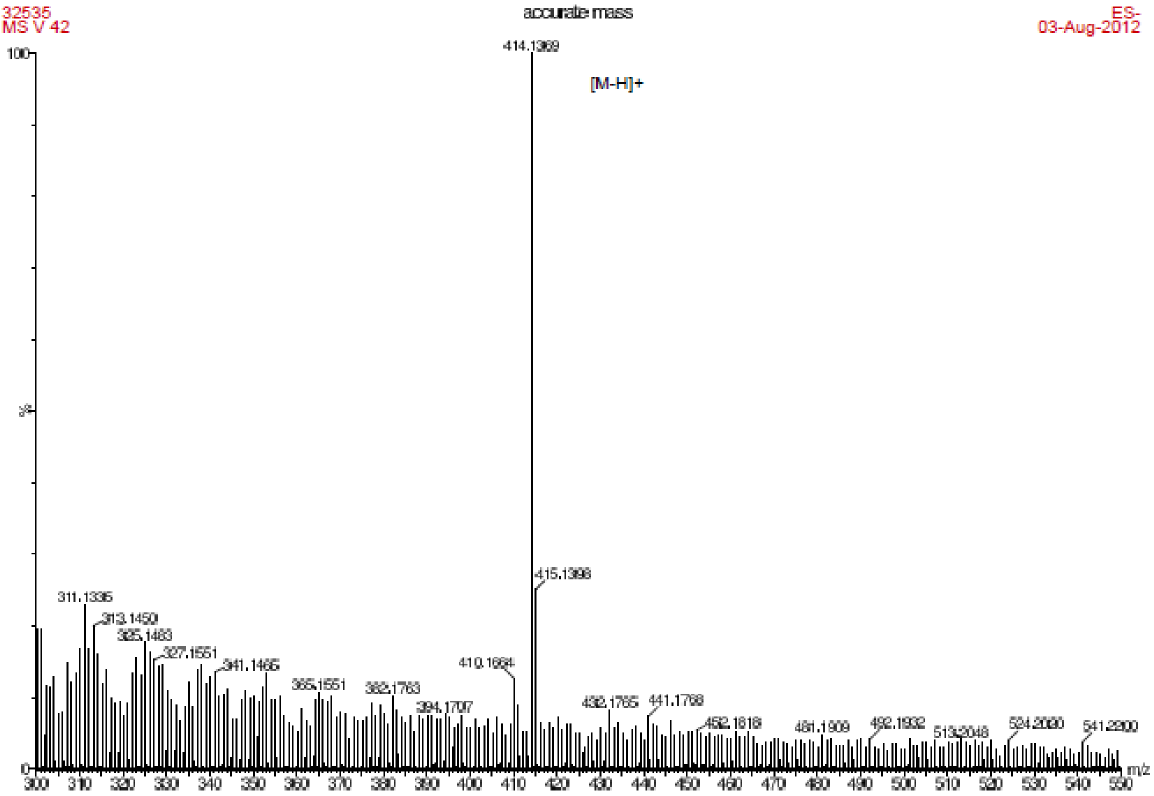

^{1}H NMR (300 MHz, DMSO)

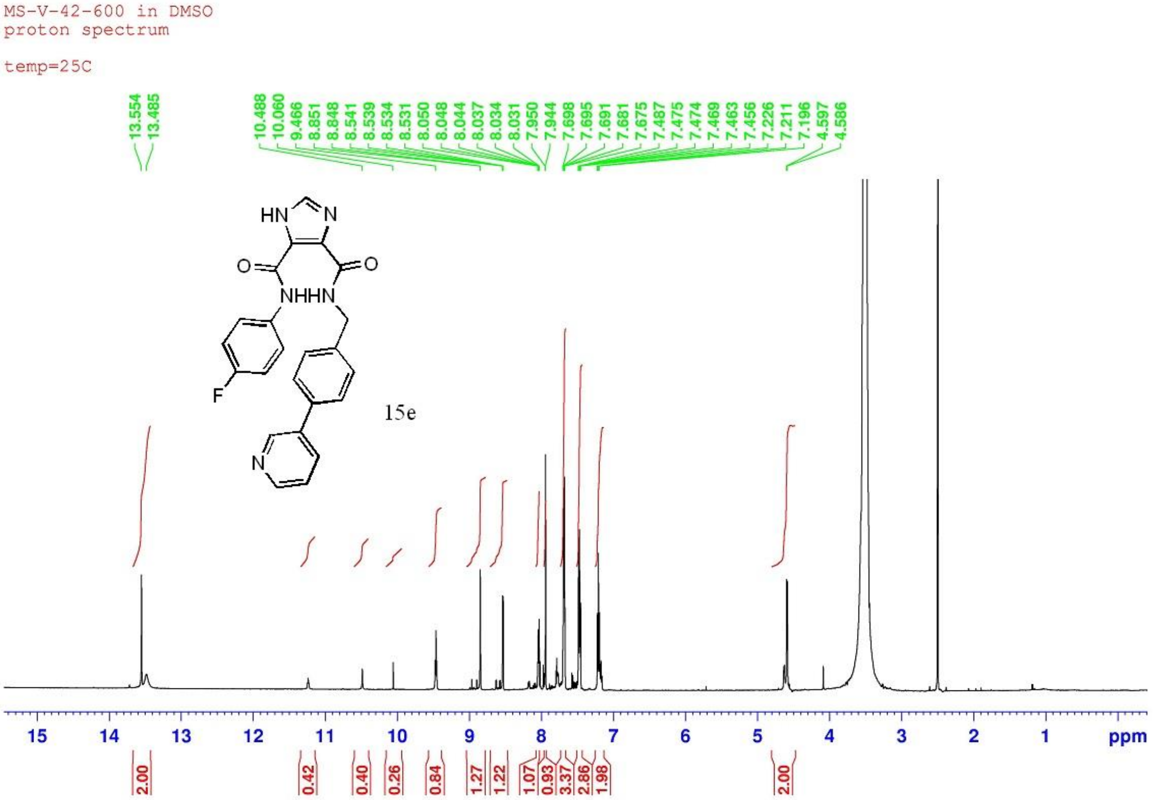

^{13}C NMR (75 MHz, DMSO)

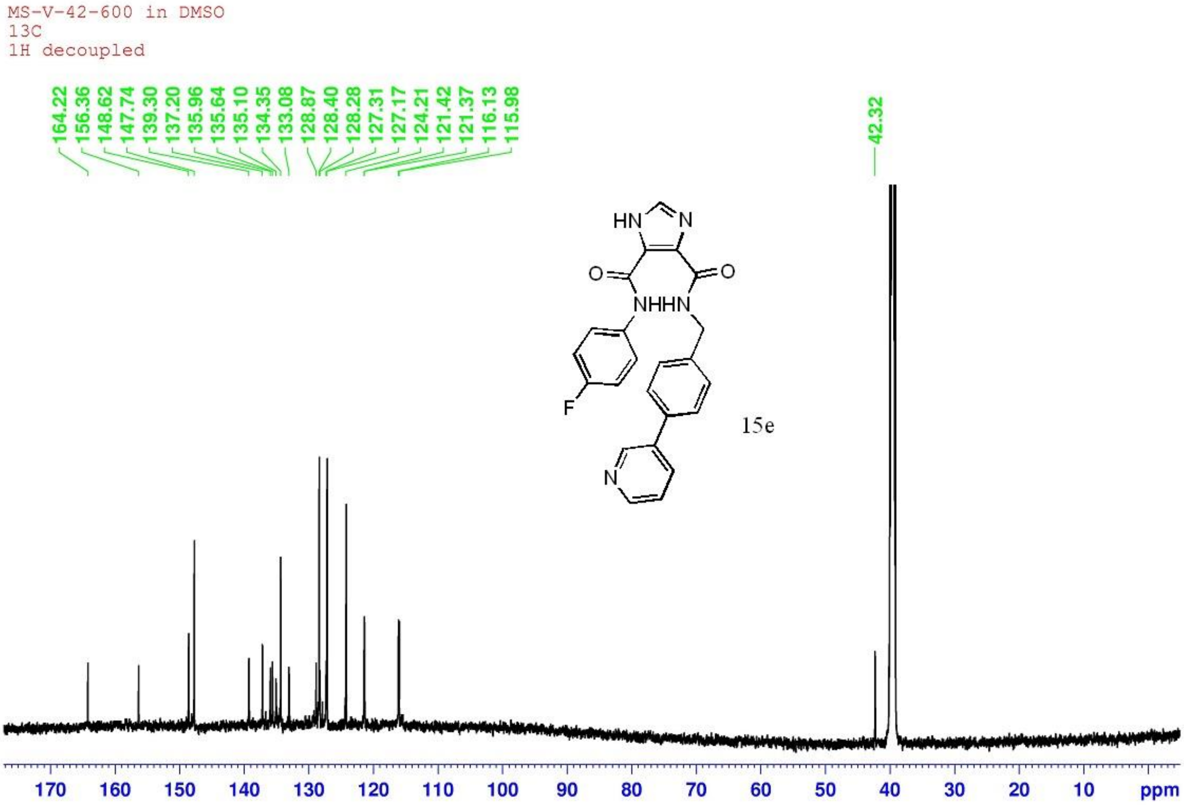

N^5-(4-fluorophenyl)-N^4-(4-(thiophen-3-yl)benzyl)-1H-imidazole-4,5-dicarbox-amide (15f)

HRMS (ES+)

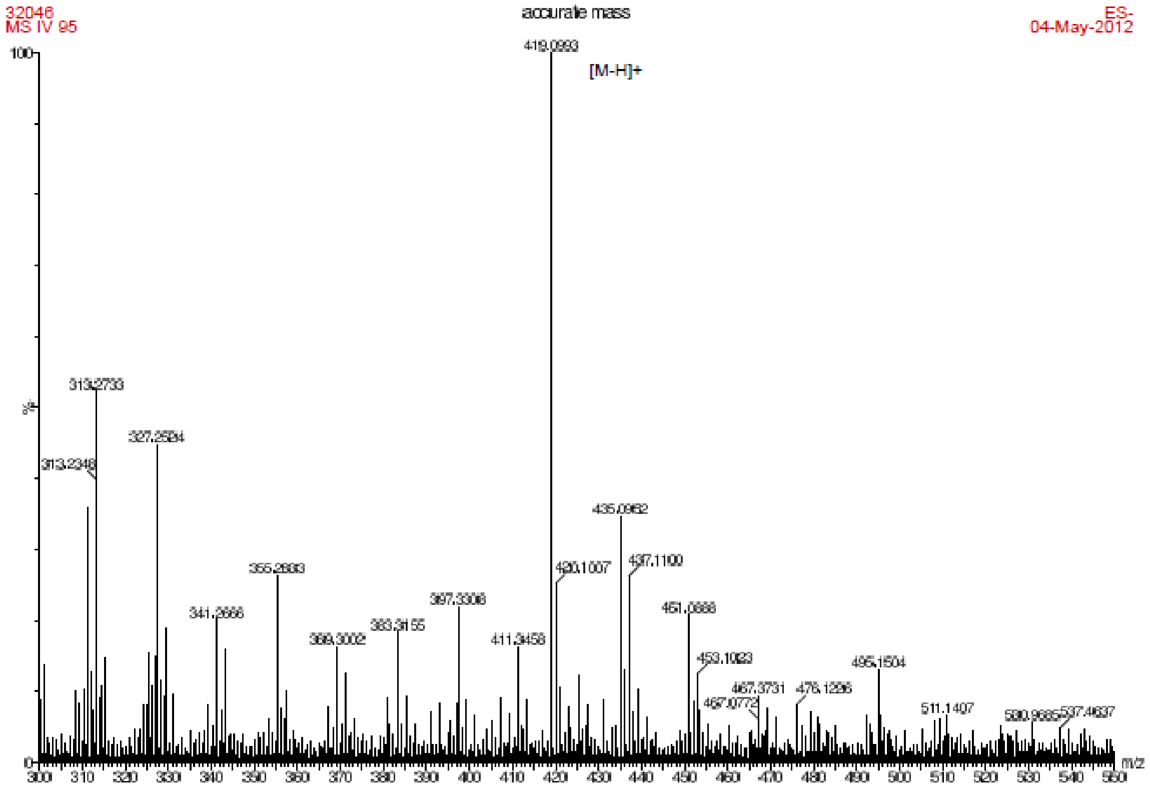

^{1}H NMR (300 MHz, DMSO)

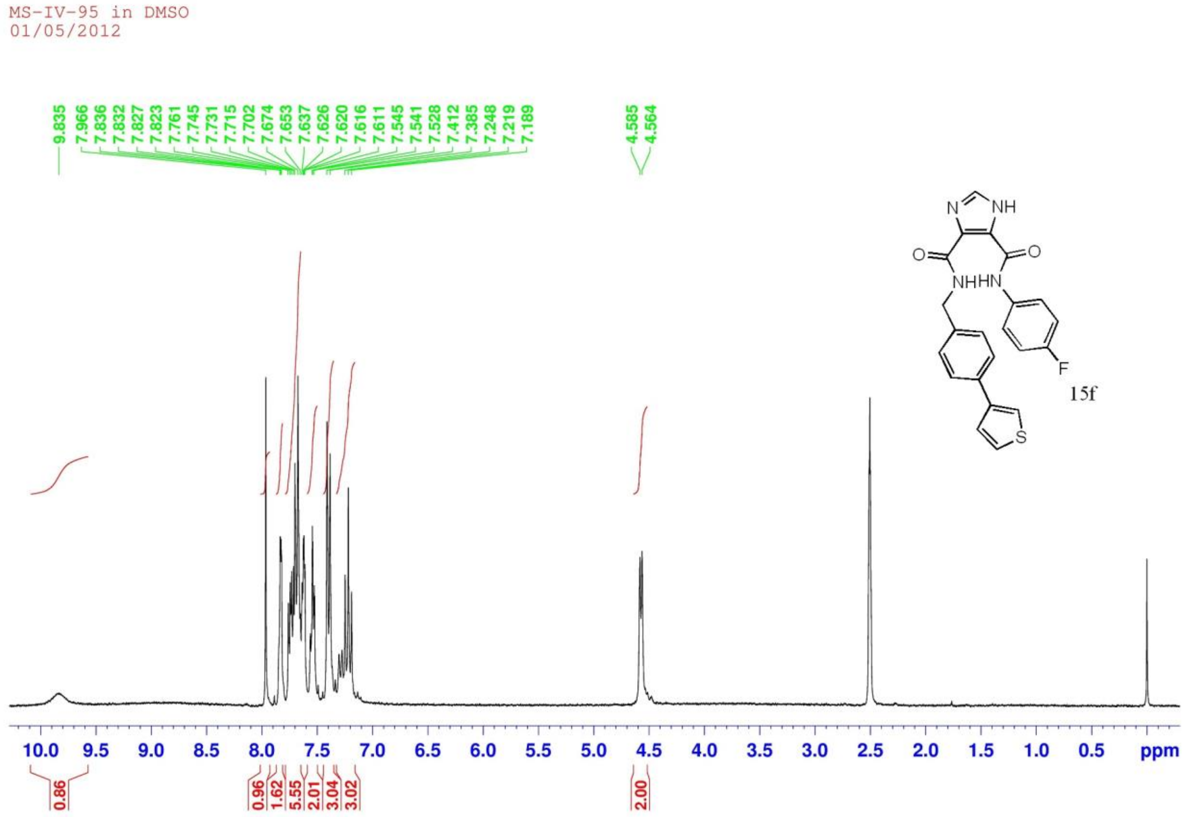

^{13}C NMR (75 MHz, DMSO)

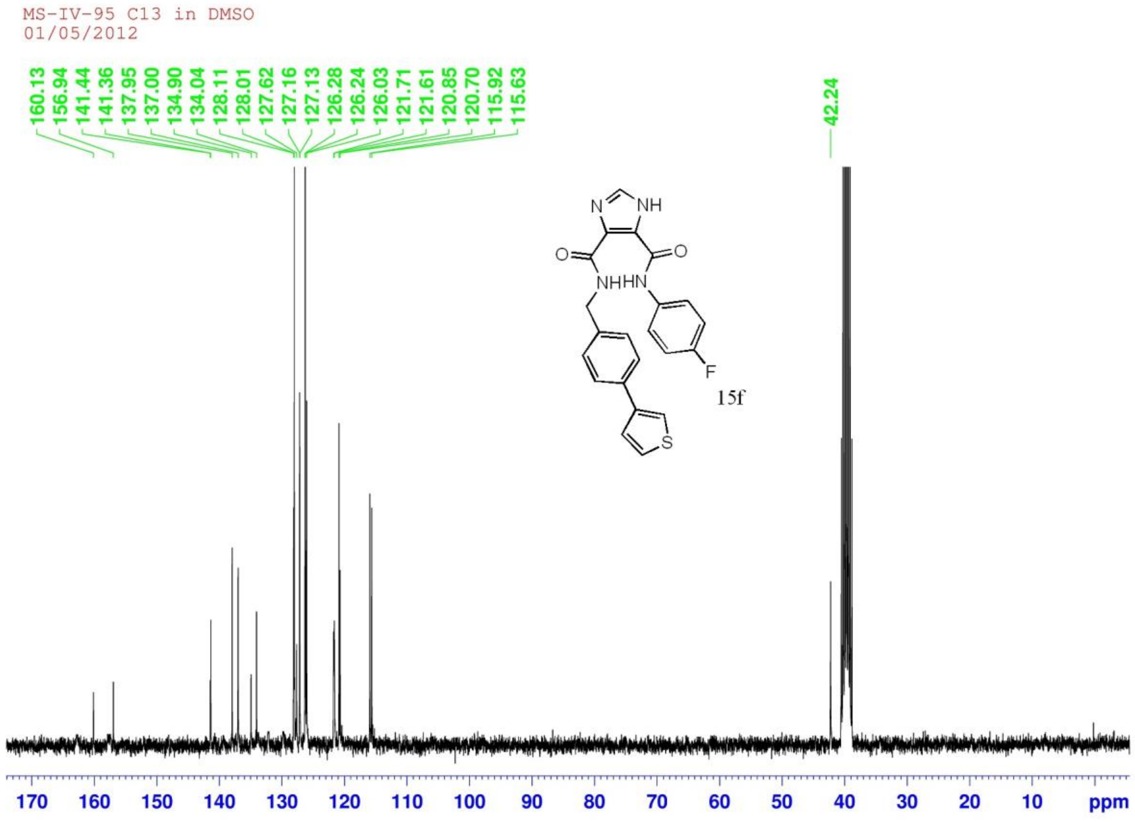

***N*[5]-(4-fluorophenyl)-*N*[4]-(4-(pyridin-4-yl)benzyl)-1H-imidazole-4,5-dicarboxamide (15g)**

HRMS (ES+)

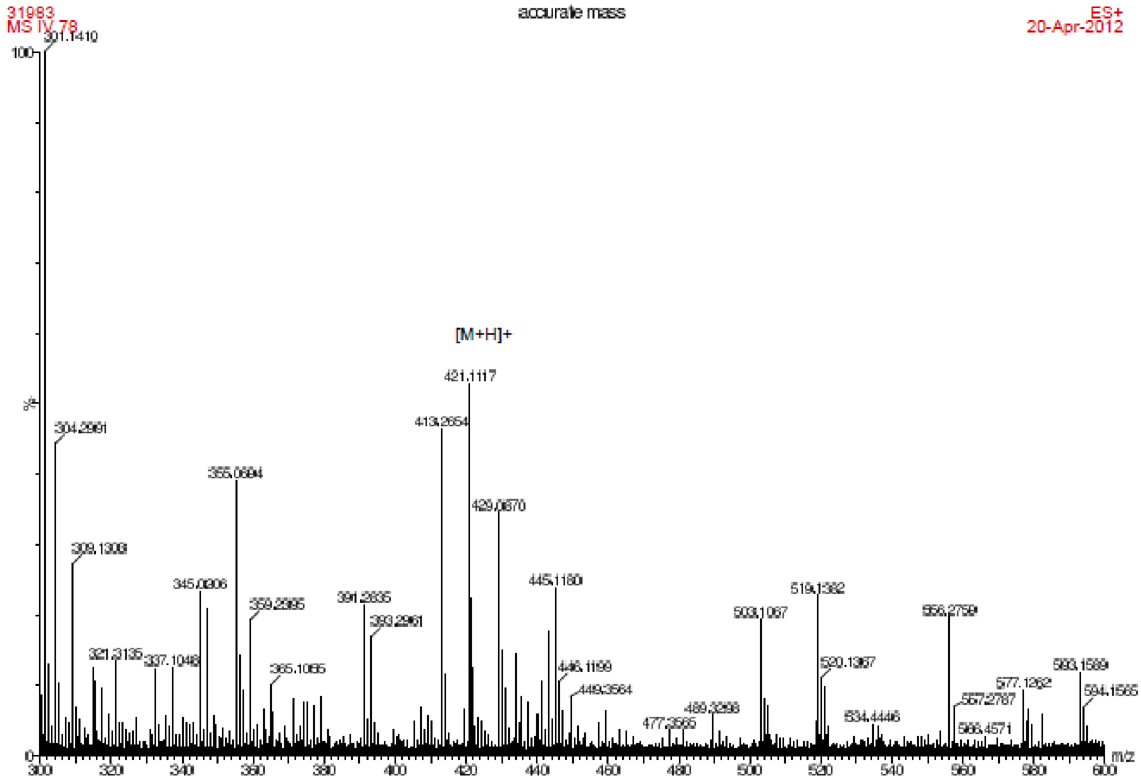

^{1}H NMR (600 MHz, DMSO)

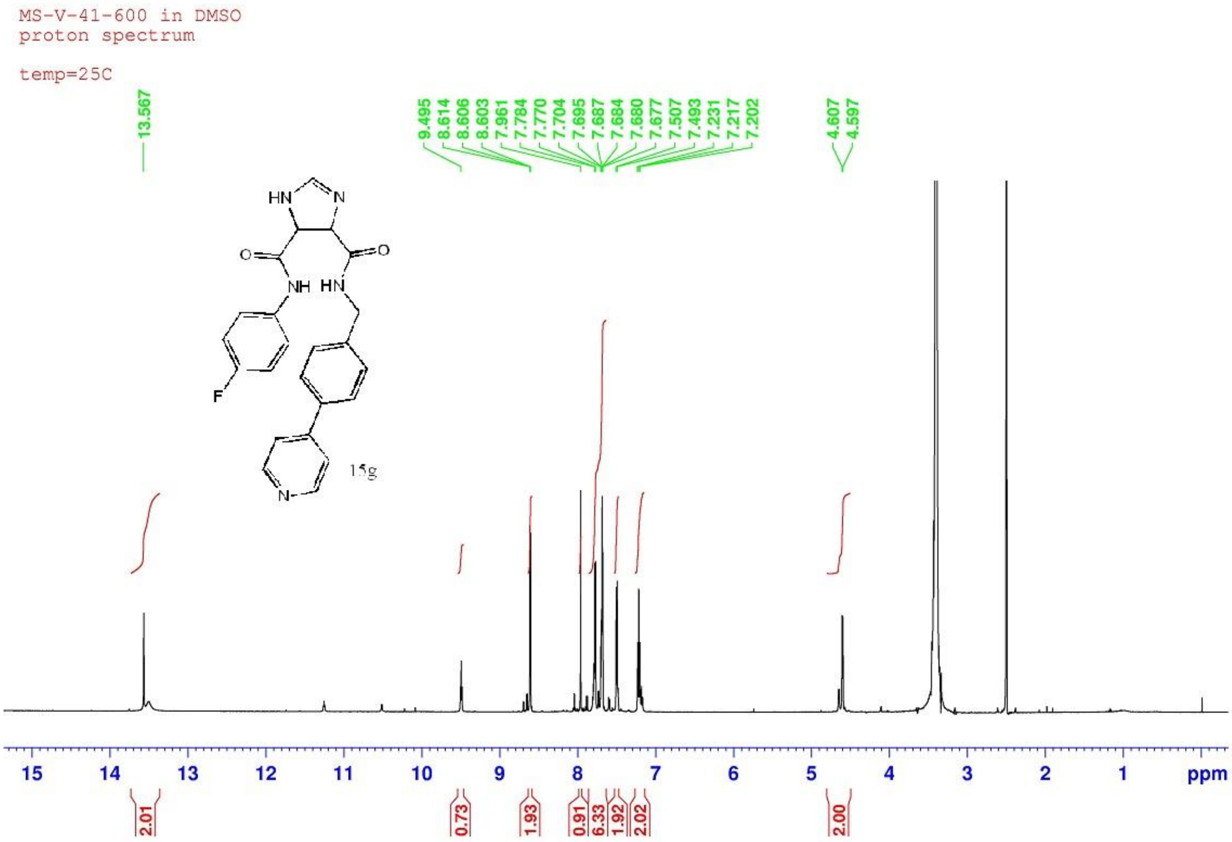

^{13}C NMR (125 MHz, DMSO)

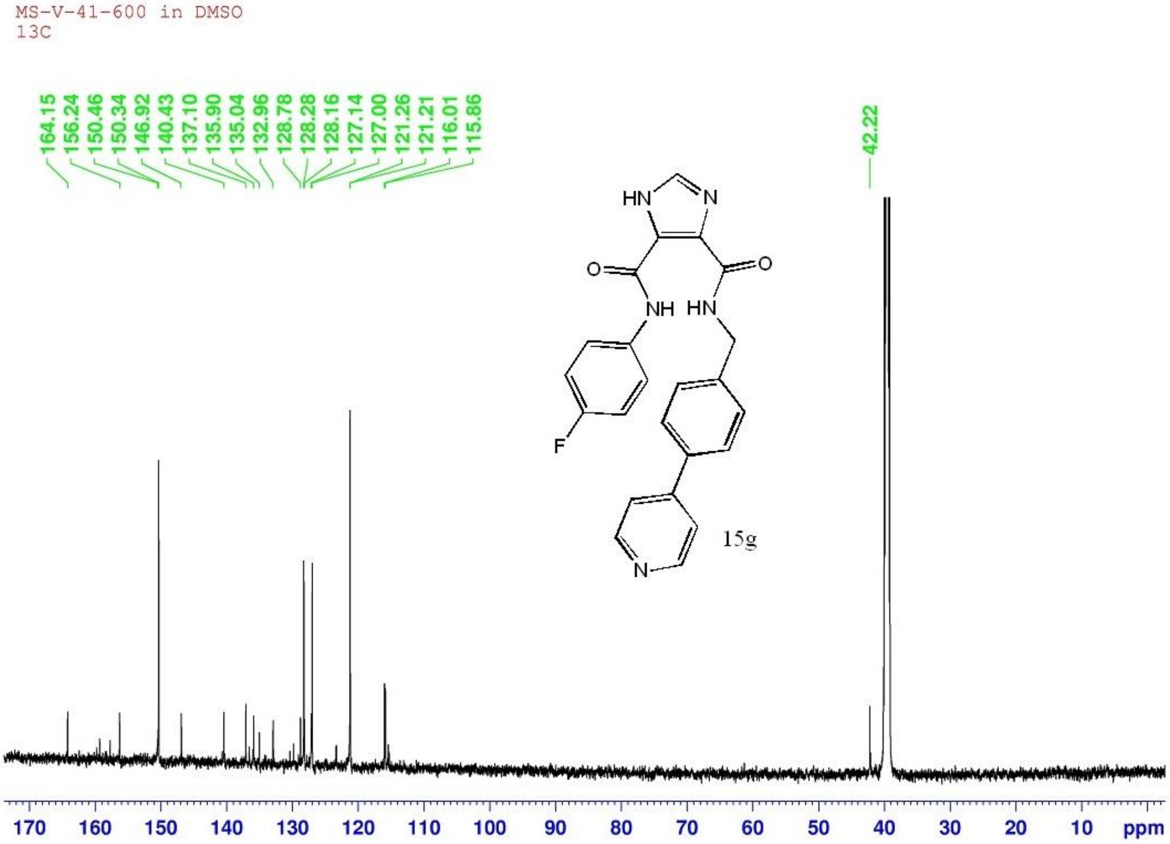

N^5-(4-fluorophenyl)-N^4-(4-methyl-2-(thiophen-3-yl)phenyl)-1H-imidazole-4,5-dicarboxamide (15h)

HRMS (ES+)

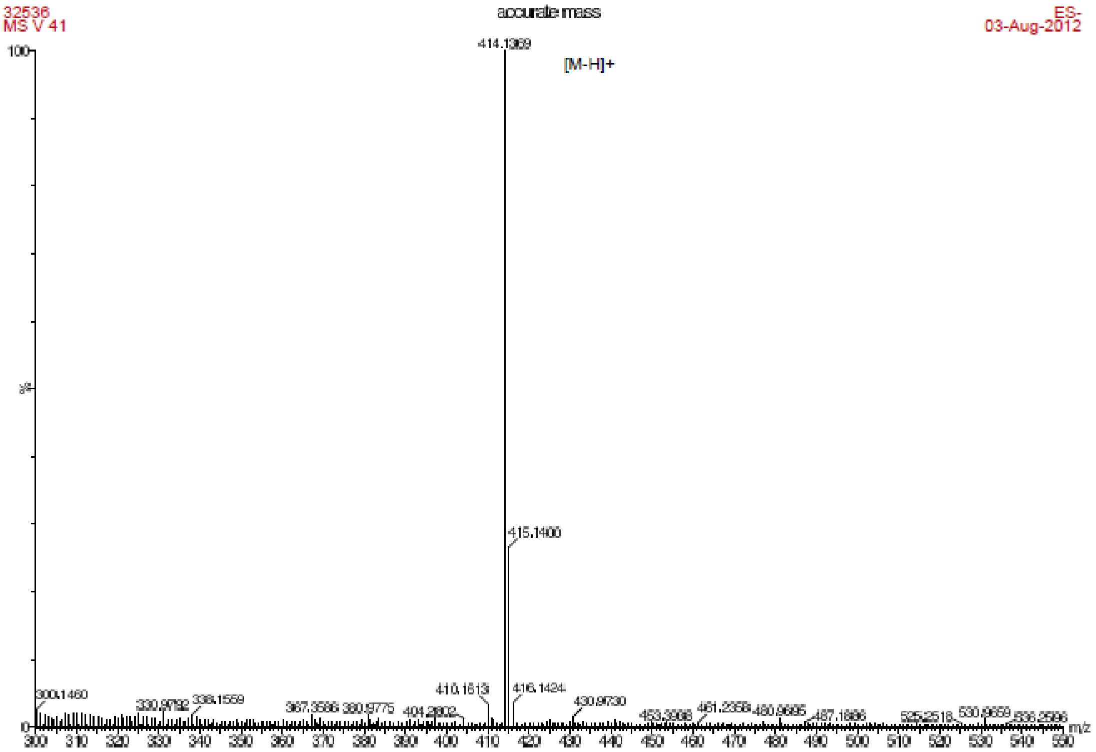

^{1}H NMR (500 MHz, DMSO)

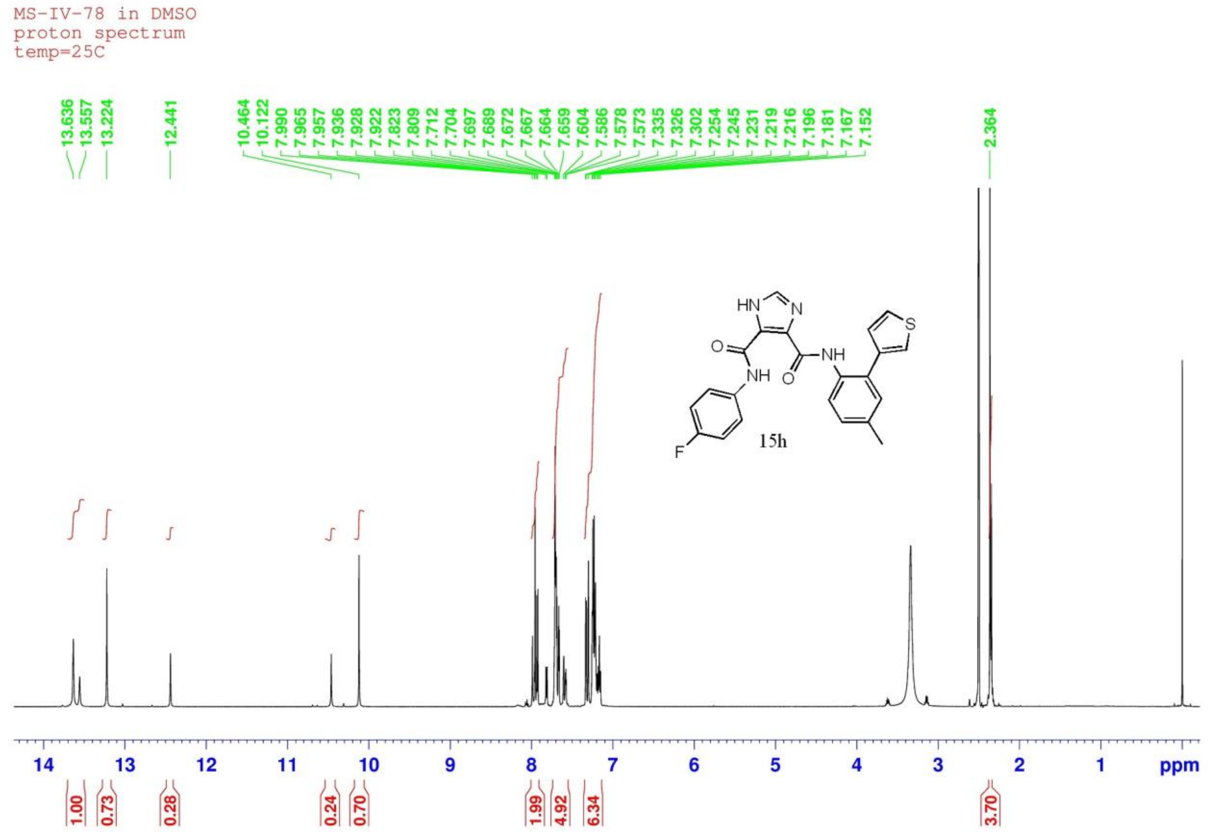

^{13}C NMR (125 MHz, DMSO)

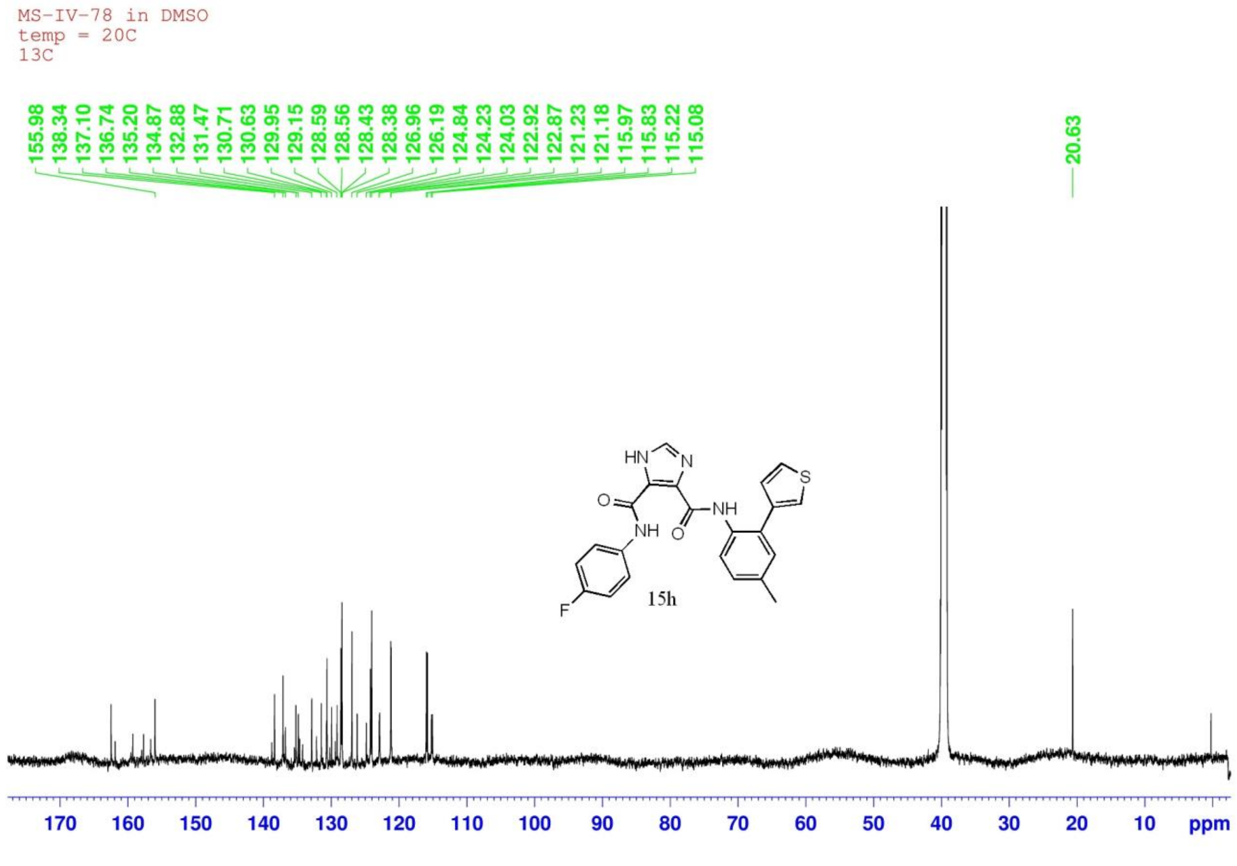

N^5-(4-fluorophenyl)-N^4-(2-(furan-2-yl)-4-methylphenyl)-1H-imidazole-4,5-dicarboxamide (15i)

HRMS (ES+)

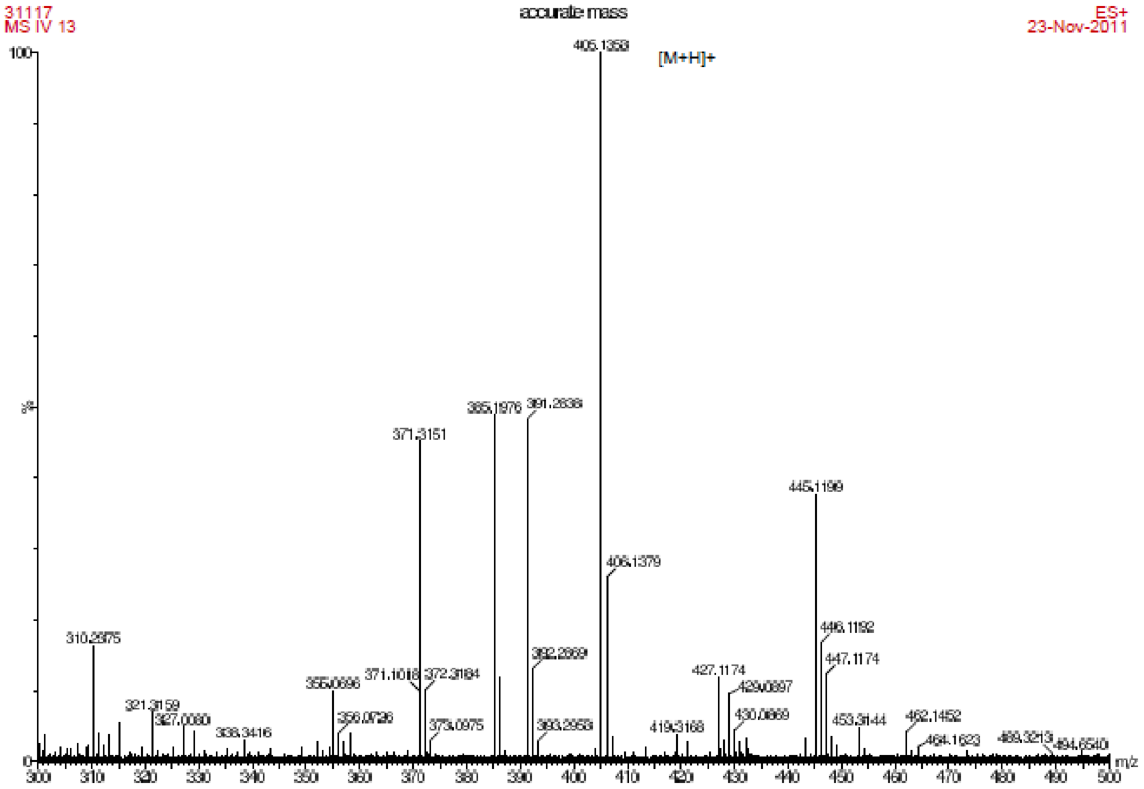

^{1}H NMR (300 MHz, DMSO)

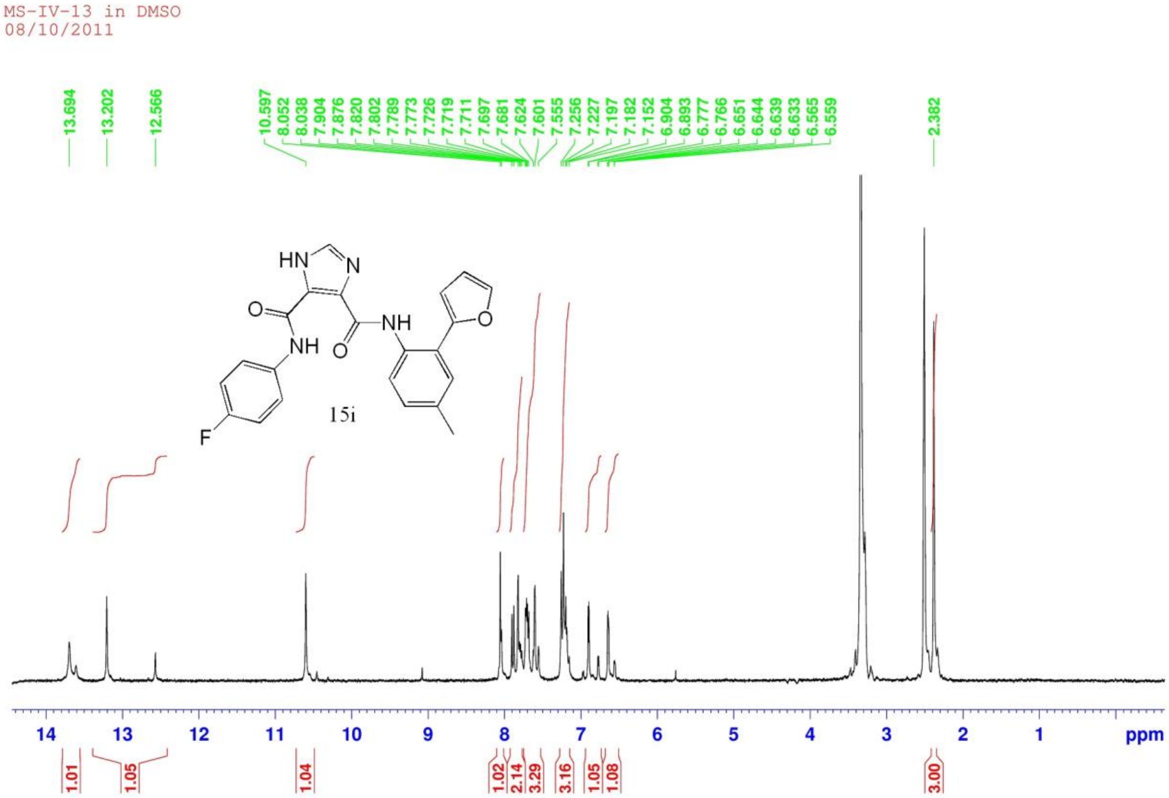

N^4-(4-methyl-2-(thiophen-3-yl)phenyl)-N^5-phenyl-1H-imidazole-4,5-dicarboxamide (15j)

HRMS (ES+)

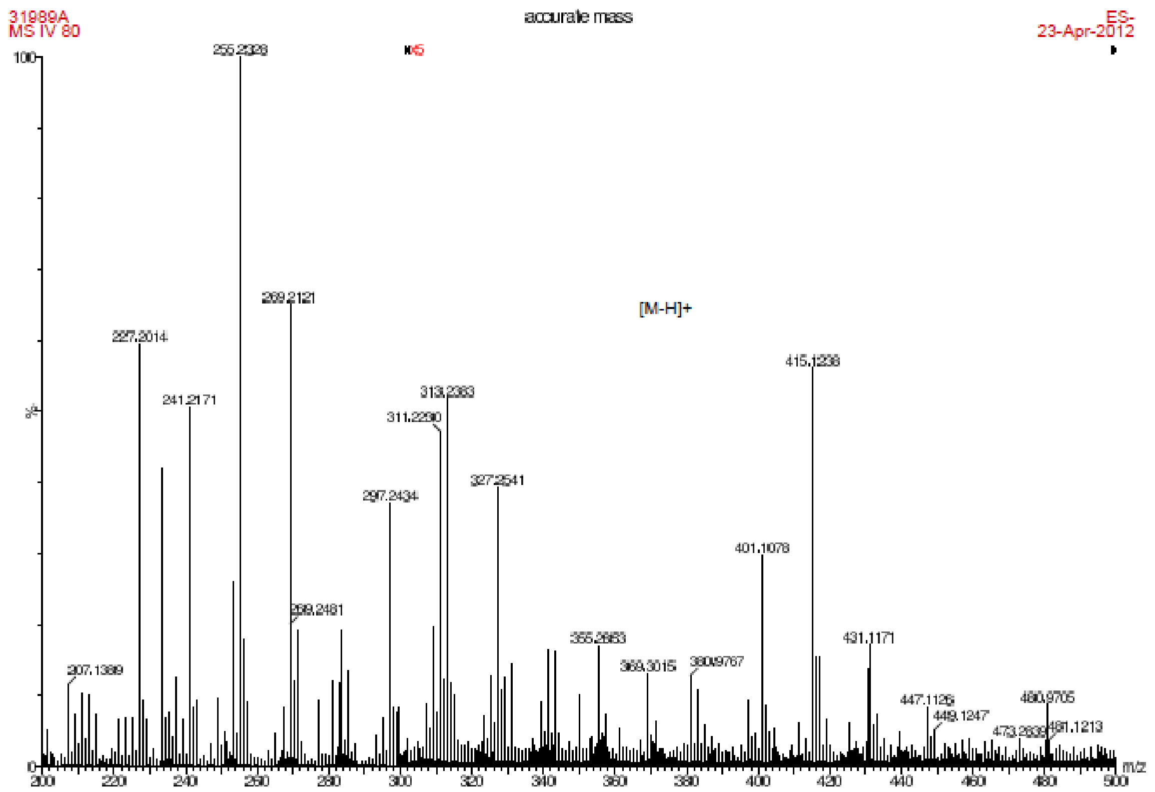

^{1}H NMR (500 MHz, DMSO)

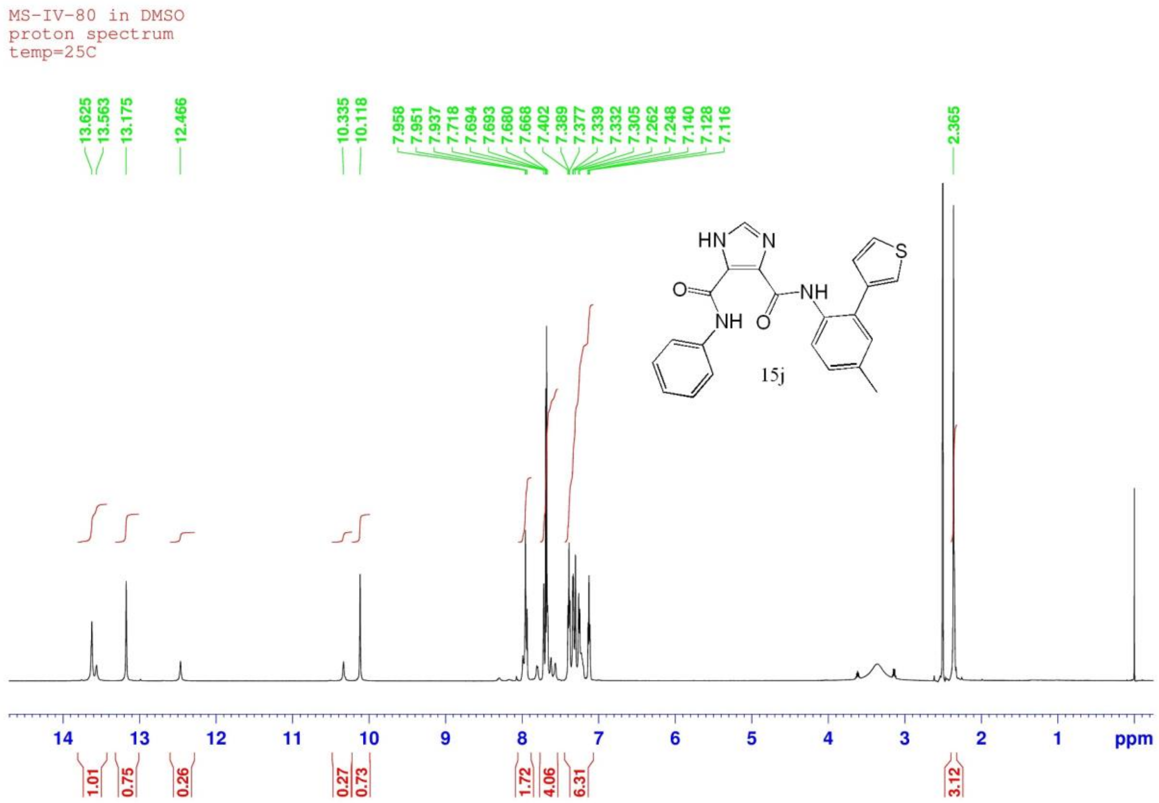

^{13}C NMR (125 MHz, DMSO)

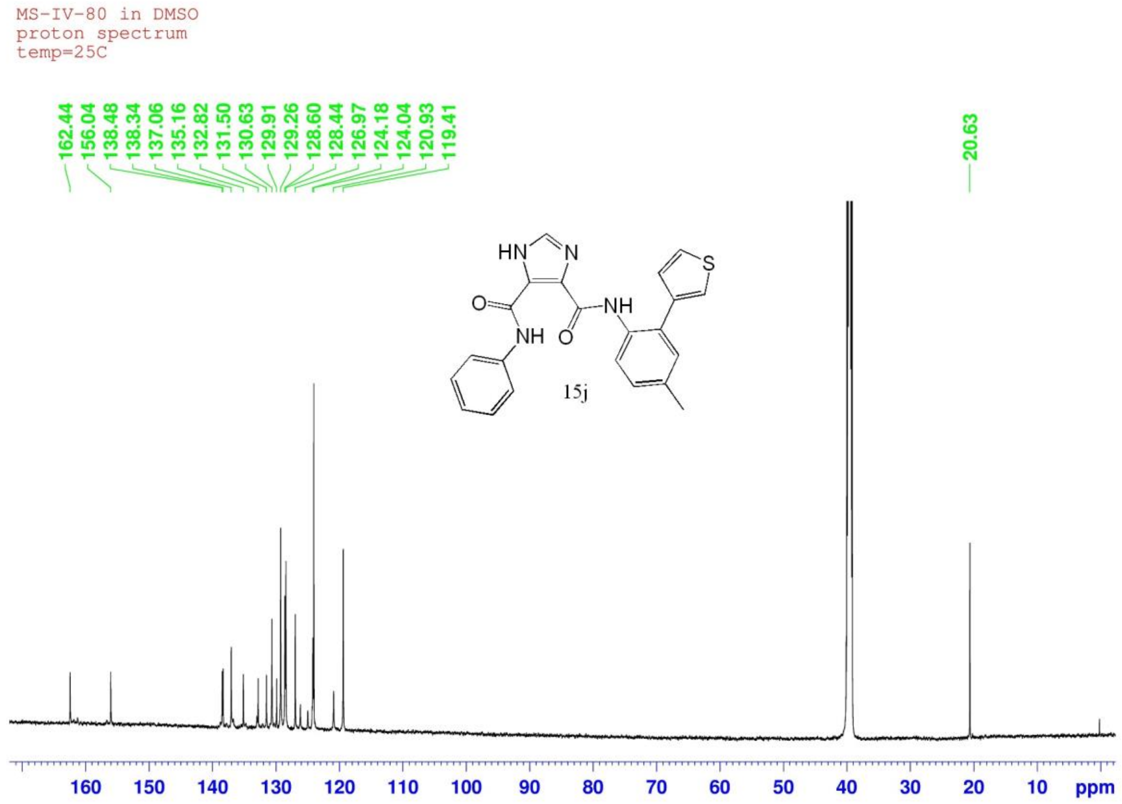

***N*[4]-(4-methyl-2-(thiophen-3-yl)phenyl)-*N*[5]-(p-tolyl)-1H-imidazole-4,5-dicarbox-amide (15k)**

HRMS (ES+)

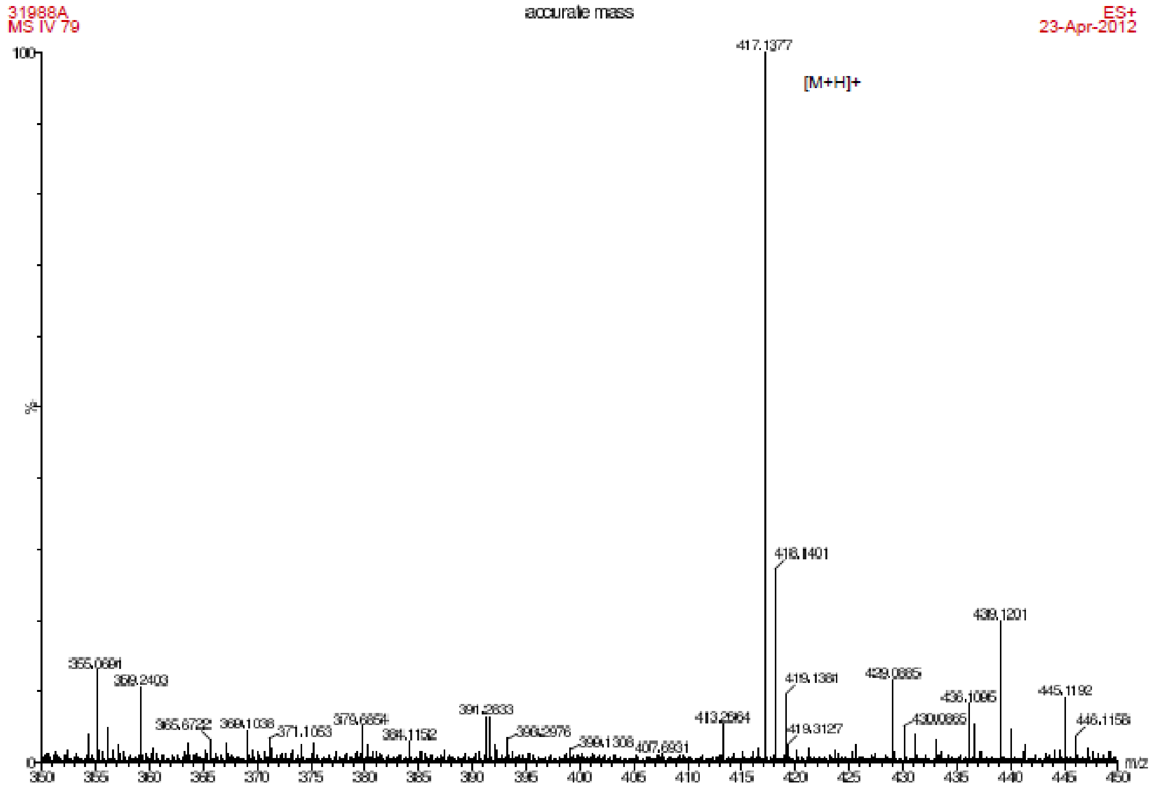

^{1}H NMR (500 MHz, DMSO)

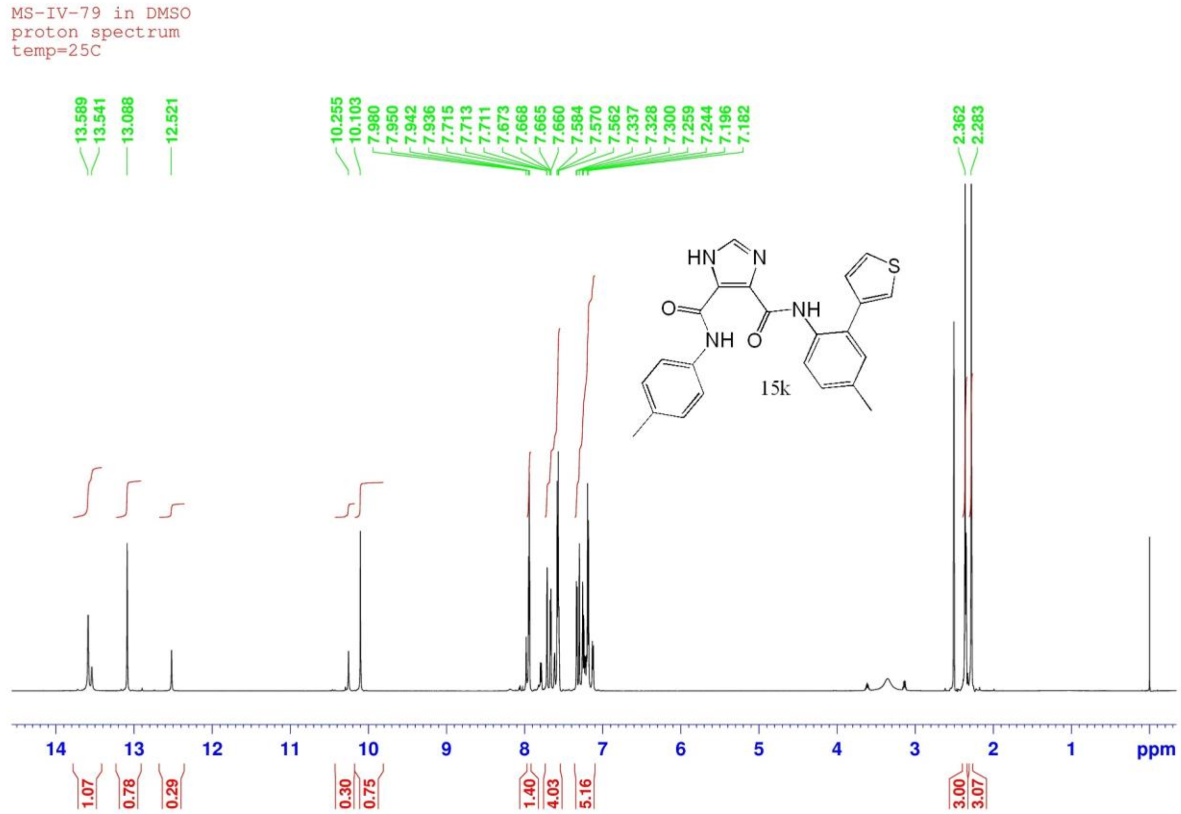

^{13}C NMR (125 MHz, DMSO)

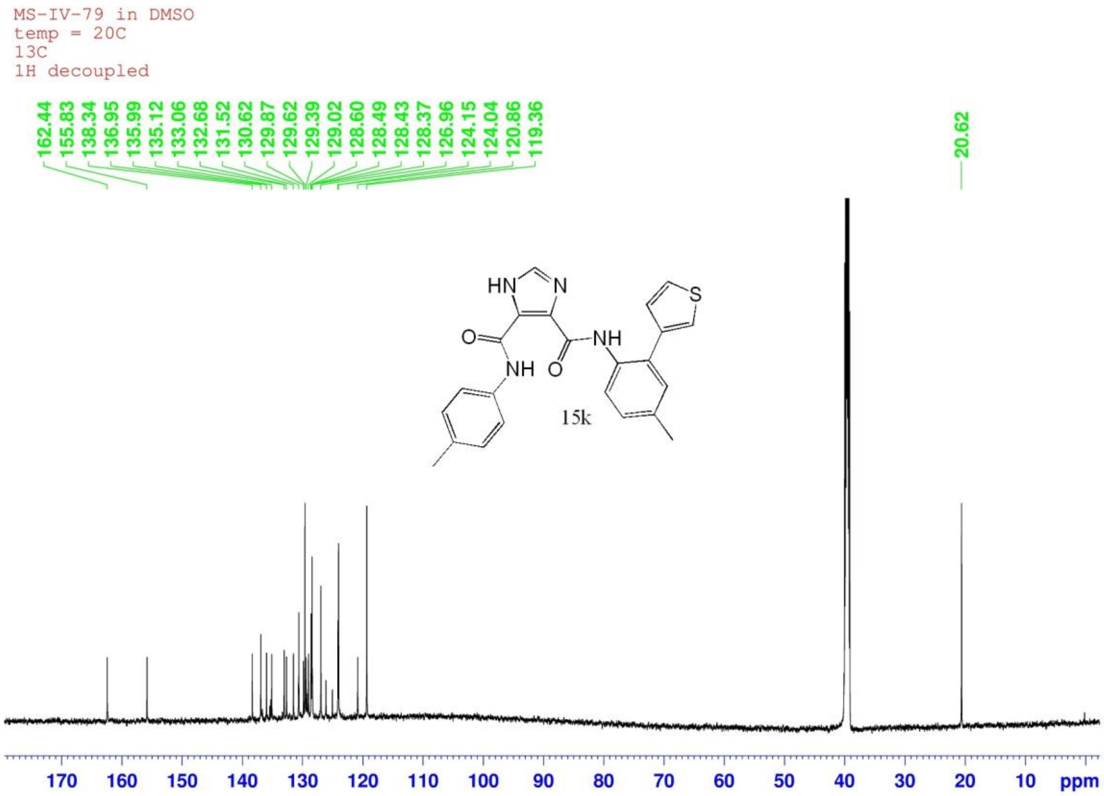

N^4-(2-(furan-2-yl)-4-methylphenyl)-N^5-(p-tolyl)-1H-imidazole-4,5-dicarboxamide (15l)

HRMS (ES+)

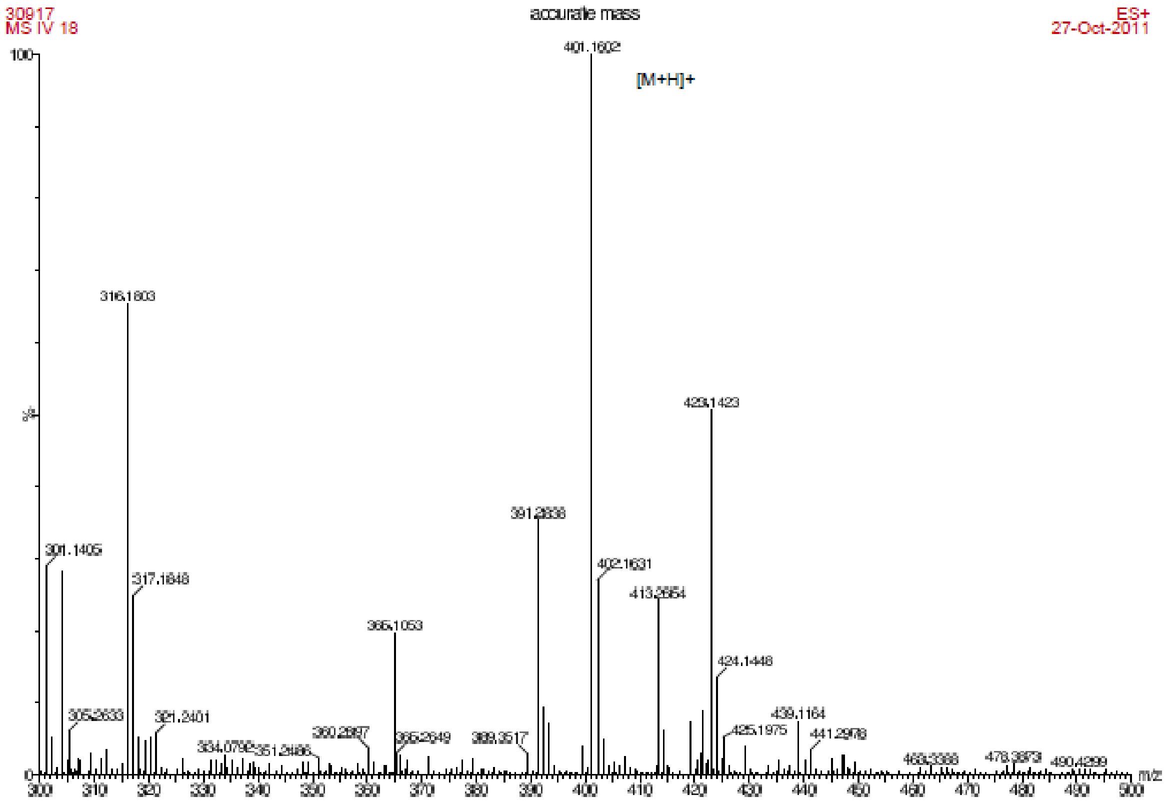

^{1}H NMR (600 MHz, DMSO)

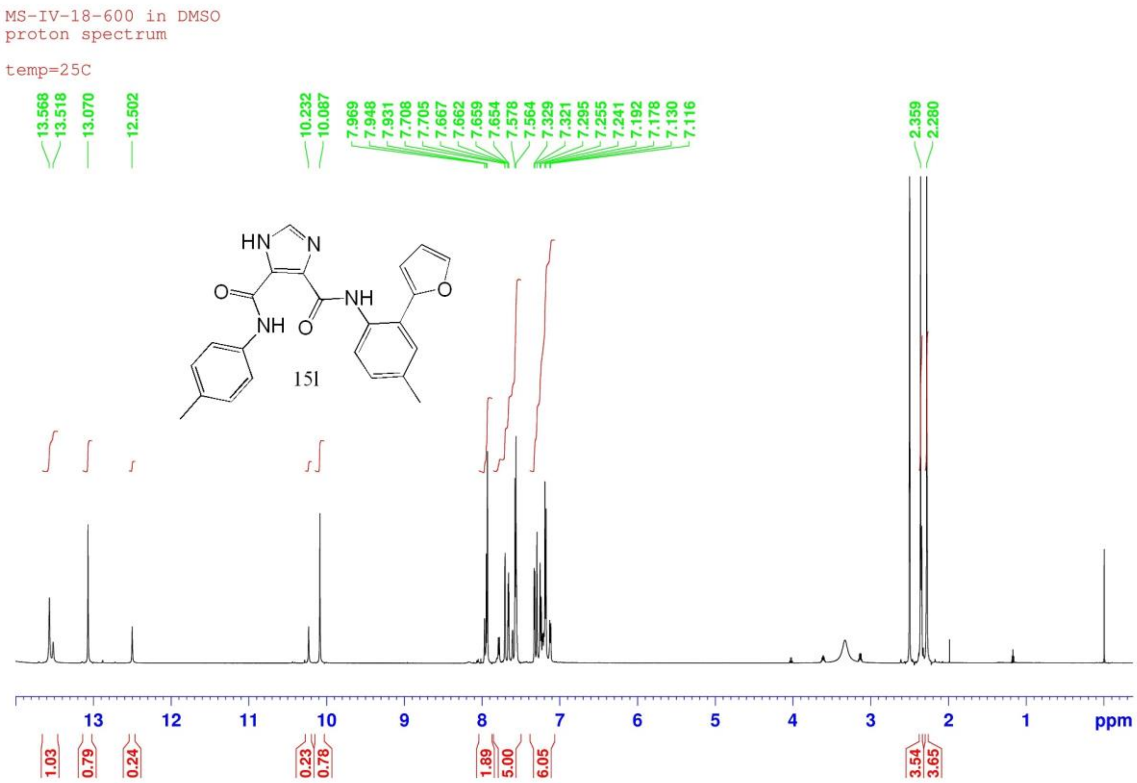

^{13}C NMR (150 MHz, DMSO)

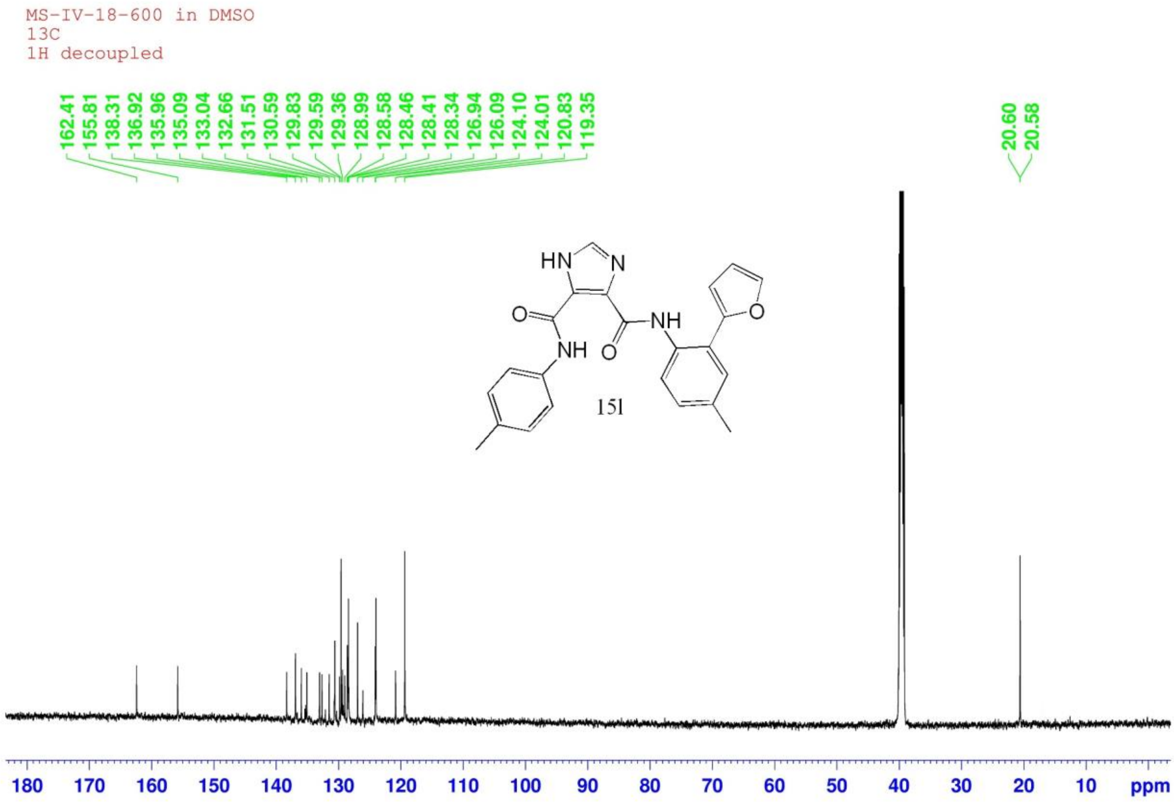

***N*5-benzyl-*N*4-(2-(furan-2-yl)-4-methylphenyl)-1H-imidazole-4,5-dicarboxamide (15m)**

HRMS (ES+)

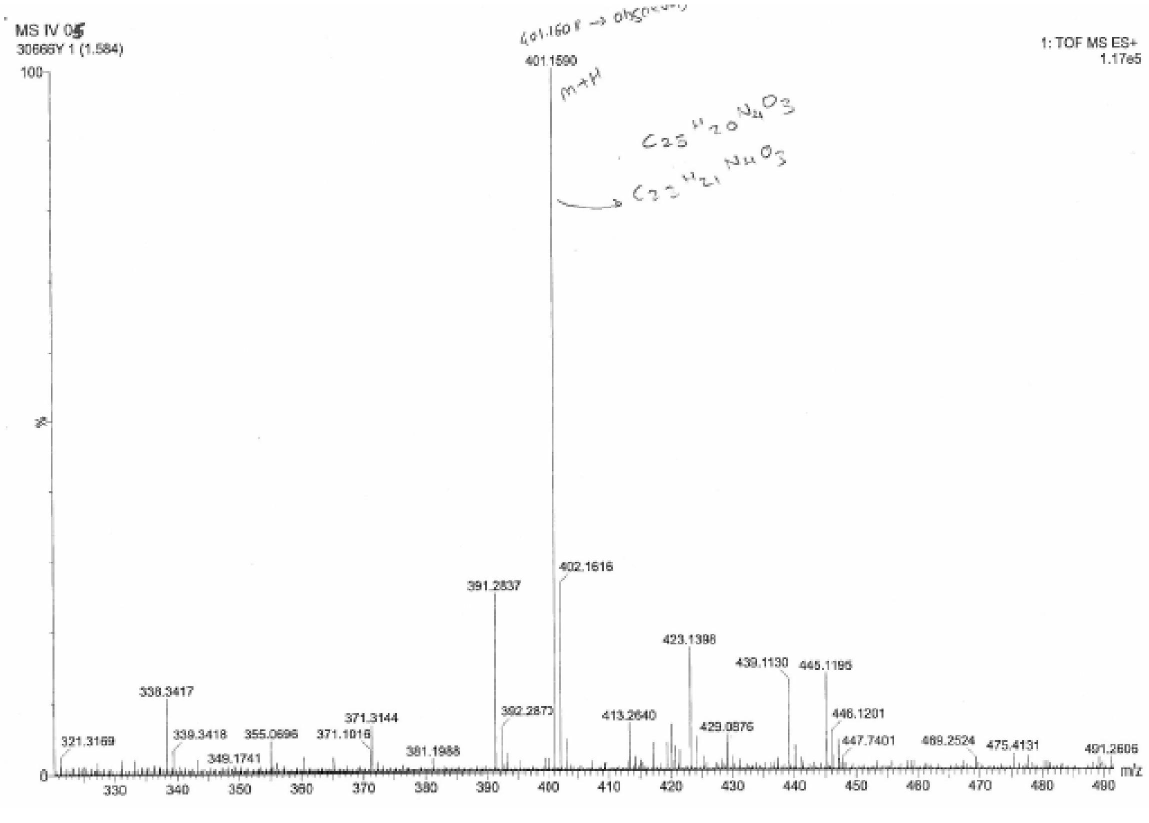

^{1}H NMR (300 MHz, $CDCl_3$)

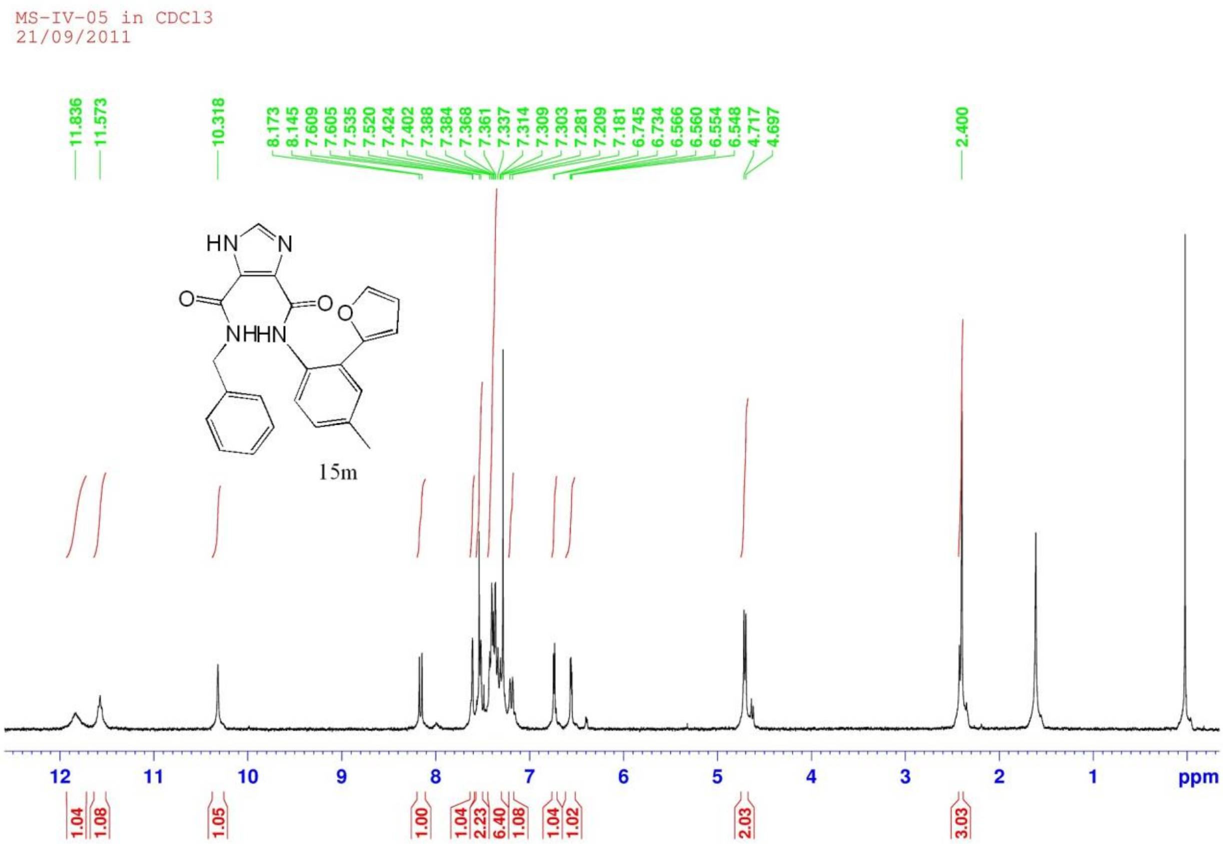

^{13}C NMR (75 MHz, $CDCl_3$)

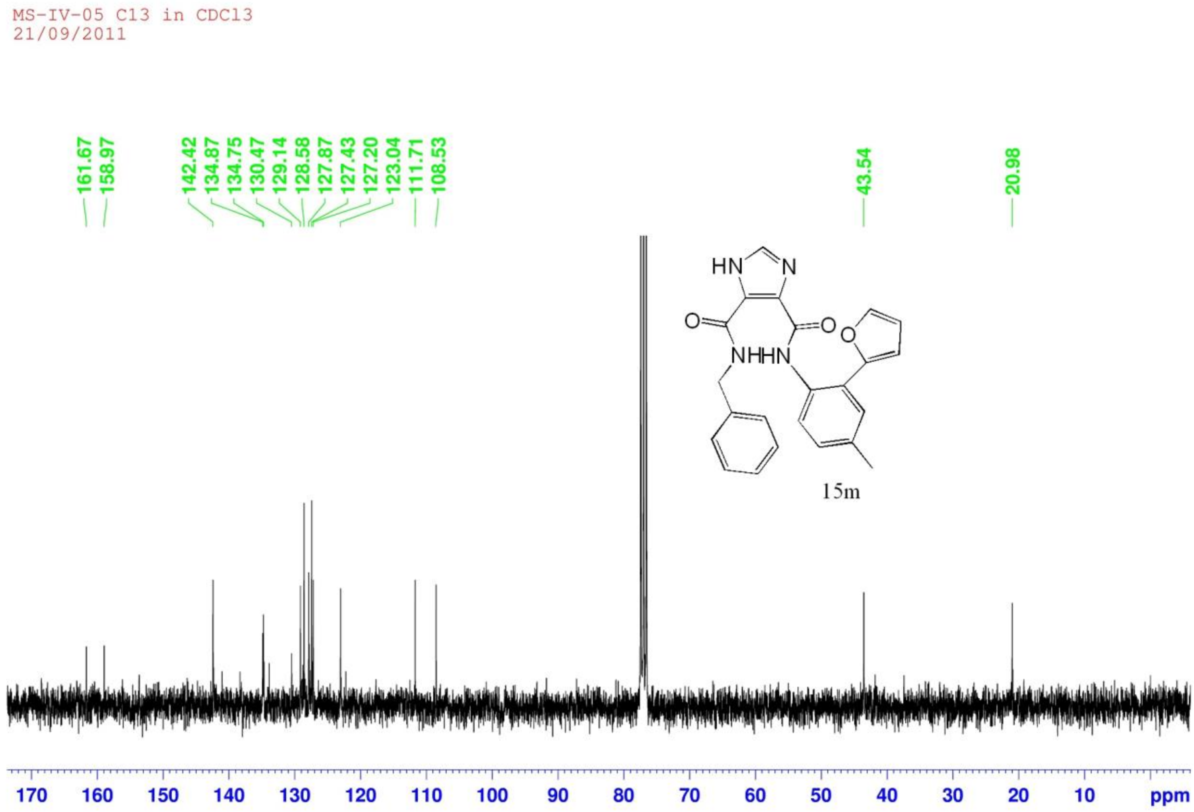

N^5-(4-fluorophenethyl)-N^4-(2-(furan-2-yl)-4-methylphenyl)-1H-imidazole-4,5-dicarboxamide (15n)

HRMS (ES+)

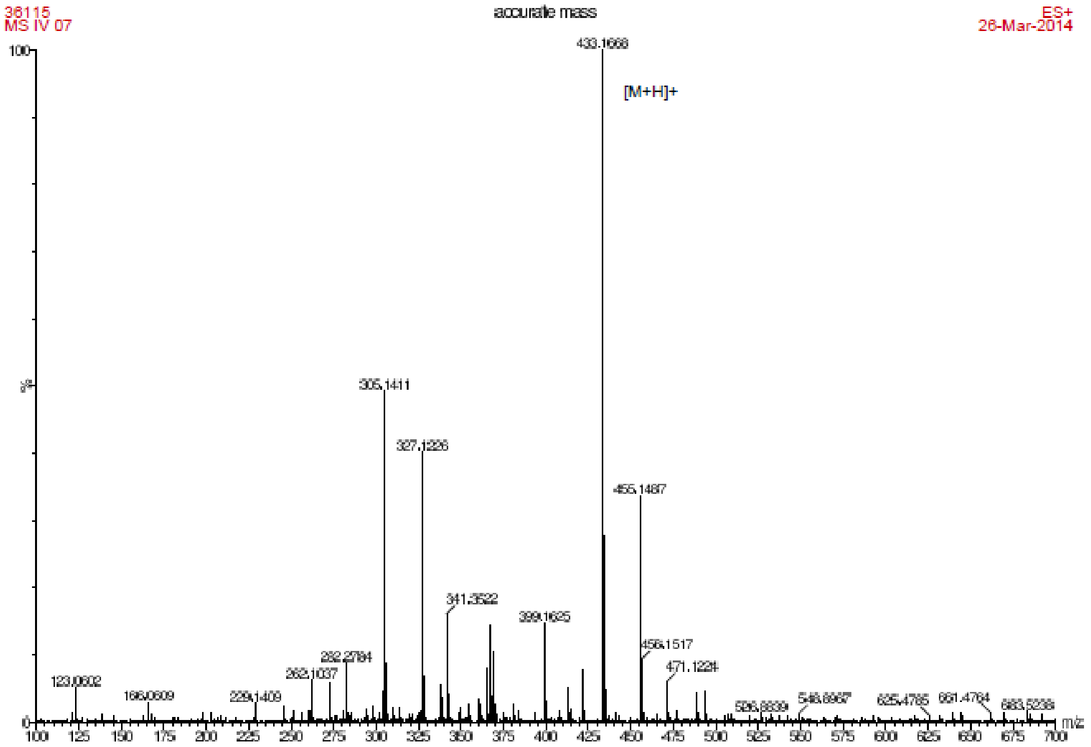

^{1}H NMR (300 MHz, $CDCl_3$)

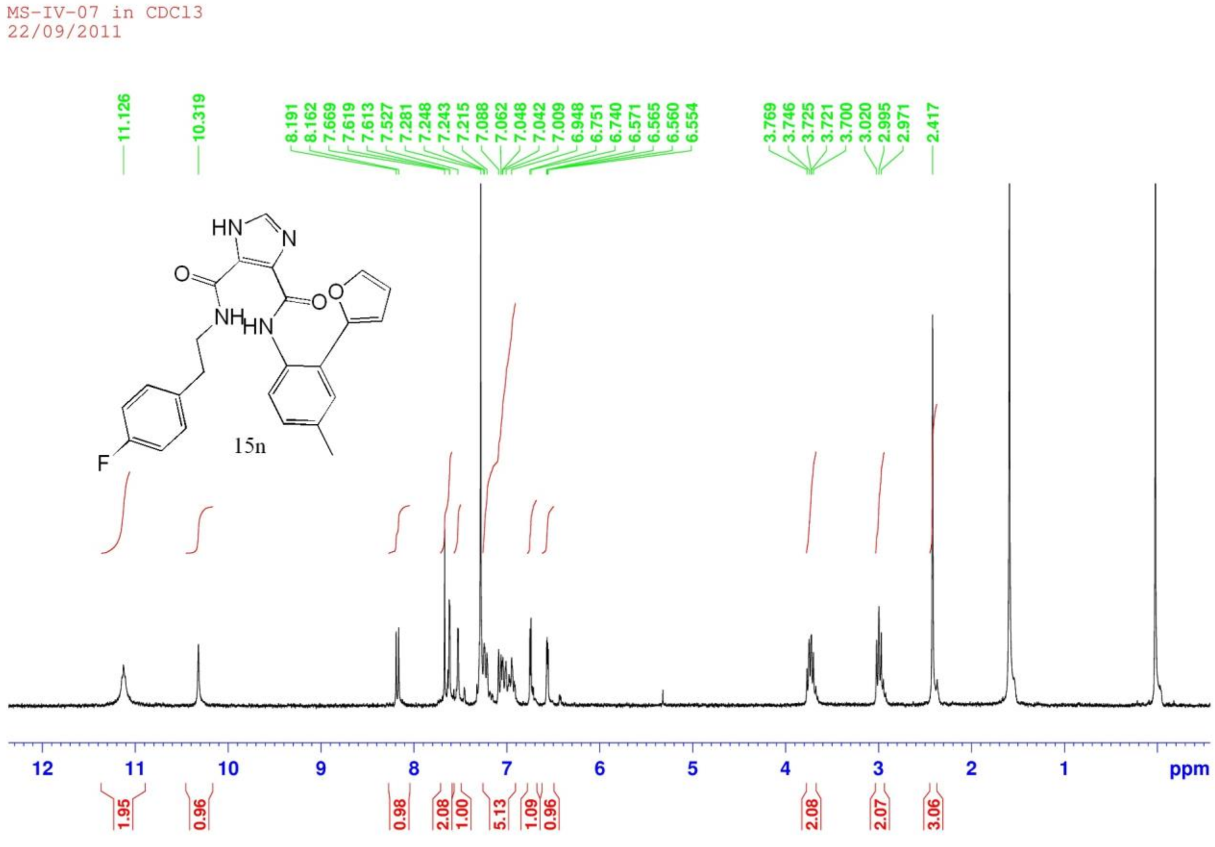

^{13}C NMR (125 MHz, $CDCl_3$)

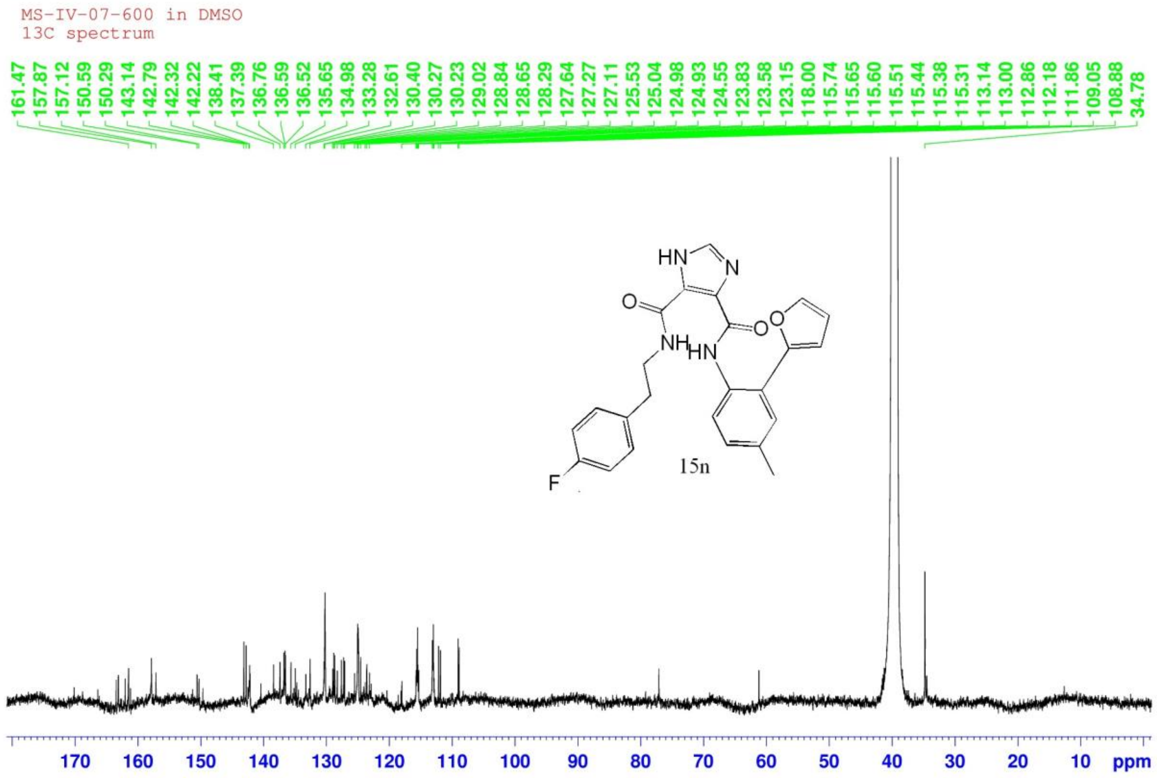

N^5-(5-methyl-3-(5-methylthiophen-2-yl)pyridin-2-yl)-N^4-phenyl-1H-imidazole-4,5-dicarboxamide (15o)

HRMS (ES+)

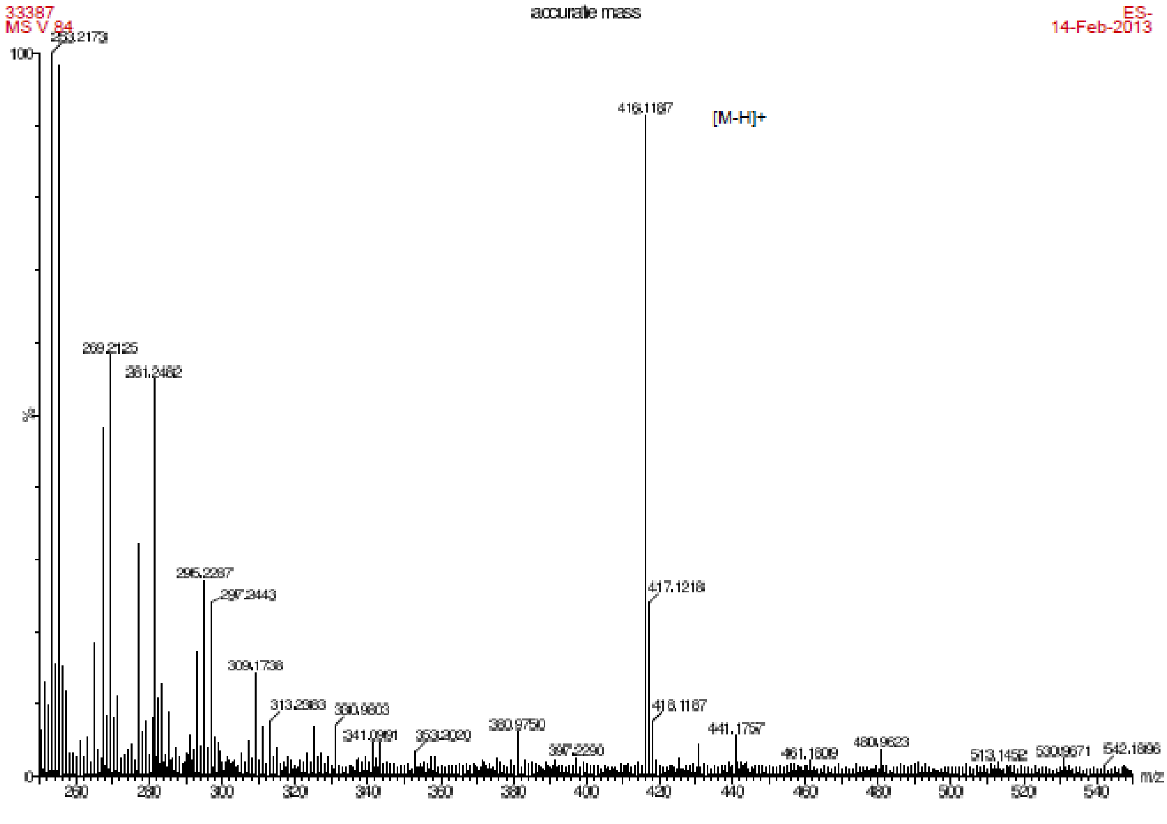

^{1}H NMR (600 MHz, DMSO)

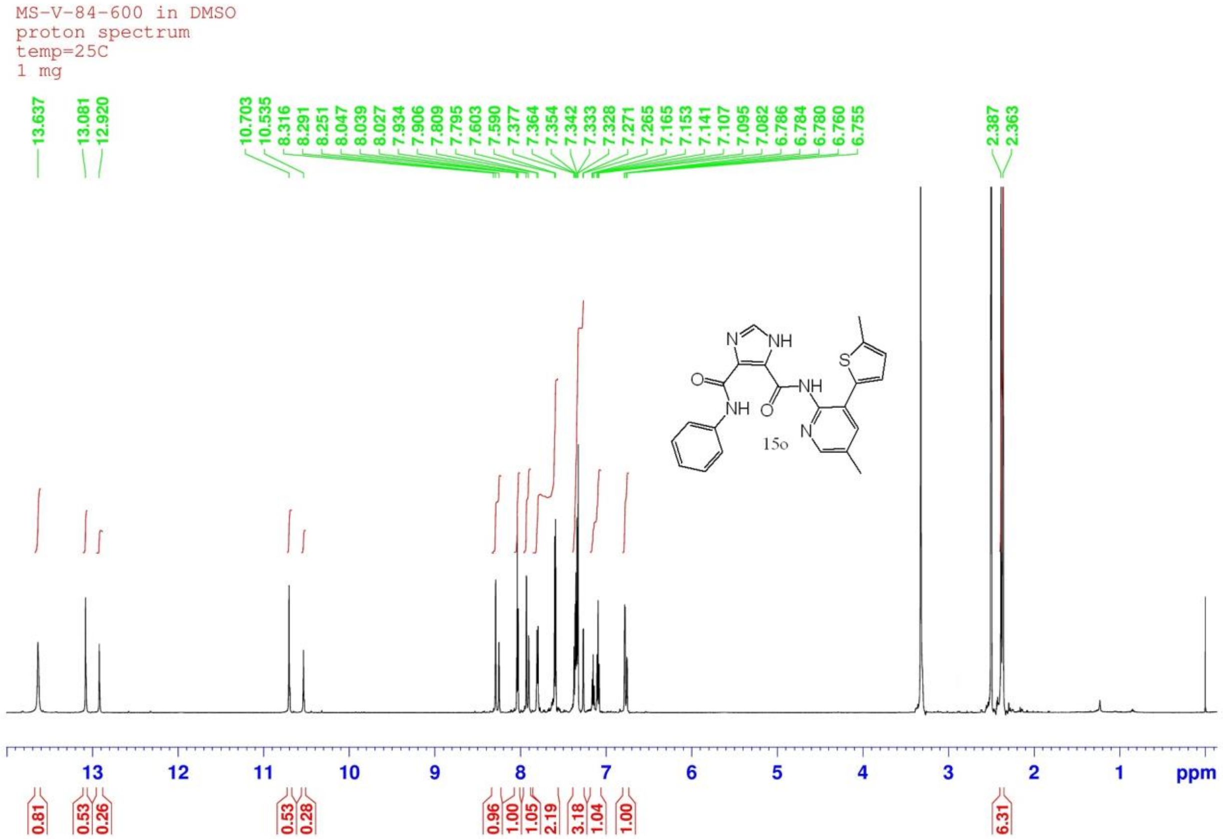

^{13}C NMR (150 MHz, DMSO)

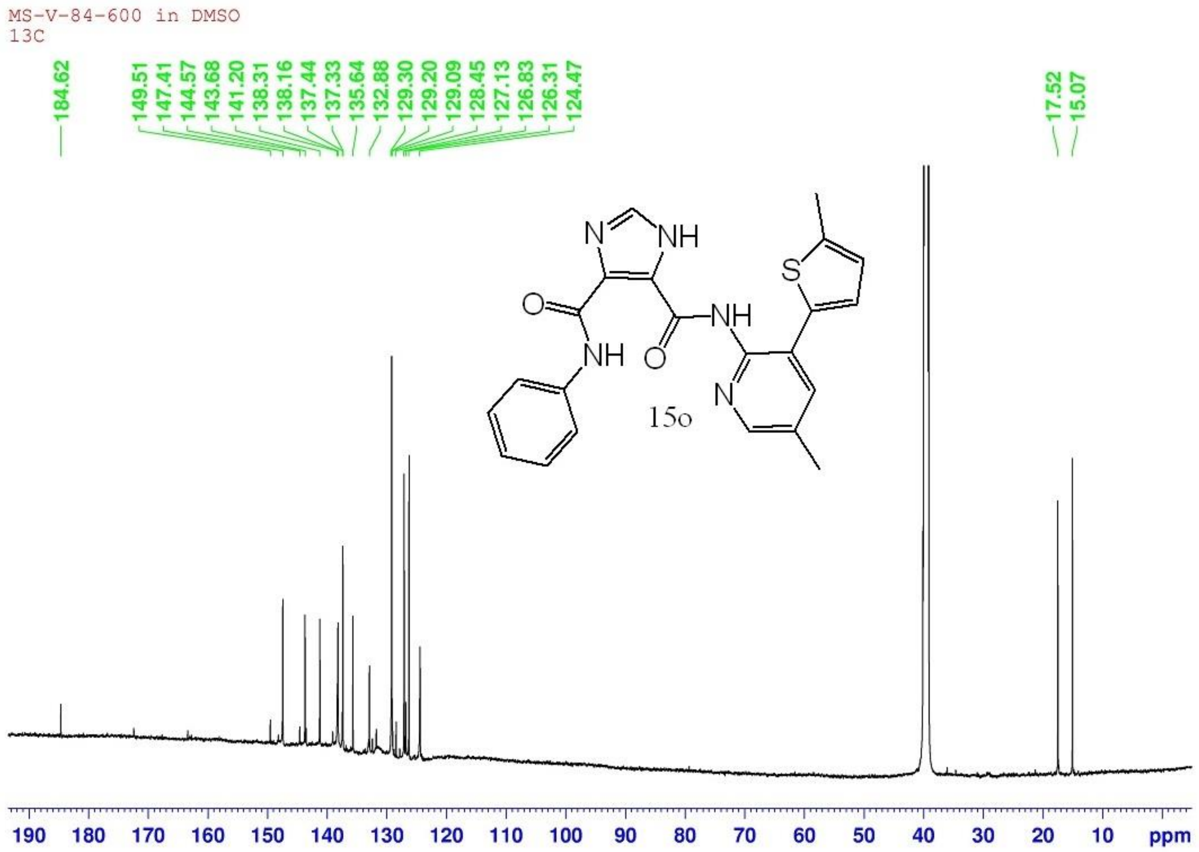

3-((4-fluorophenyl)carbamoyl)pyrazine-2-carboxylic acid (18a)

^{1}H NMR (300 MHz, DMSO)

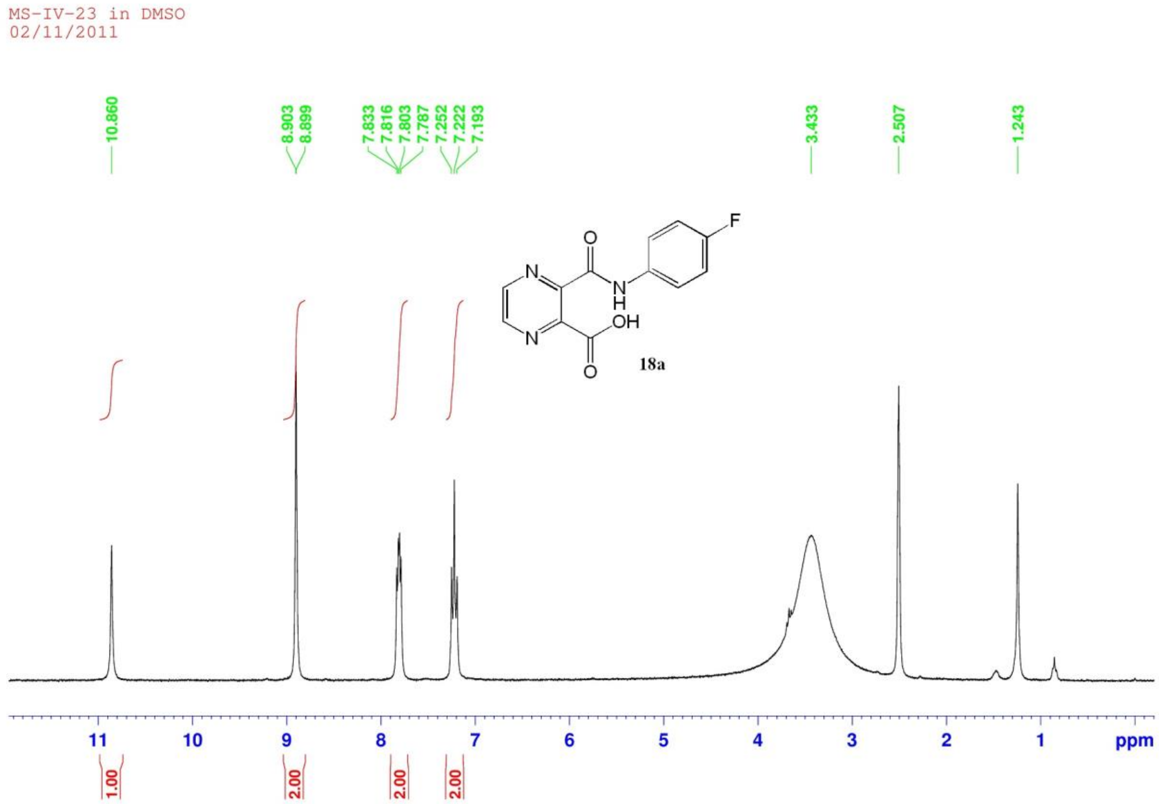

3-(p-tolylcarbamoyl)pyrazine-2-carboxylic acid (18b)

^{1}H NMR (300 MHz, DMSO)

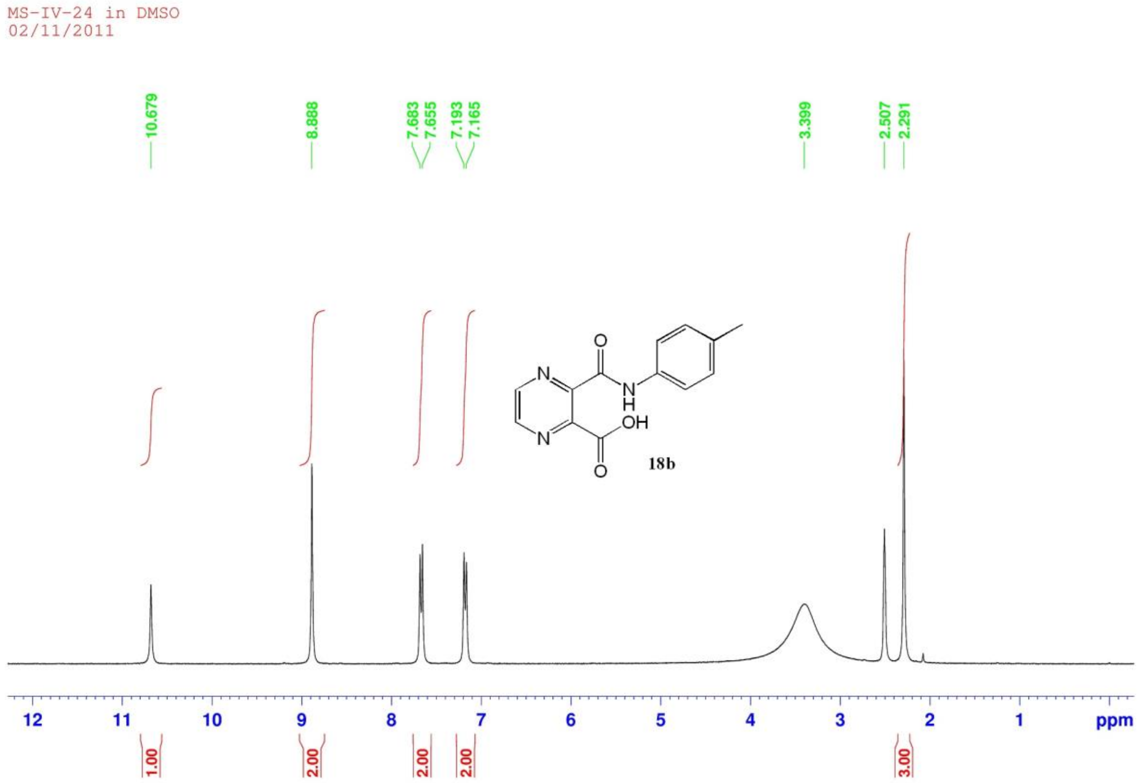

^{13}C NMR (75 MHz, DMSO)

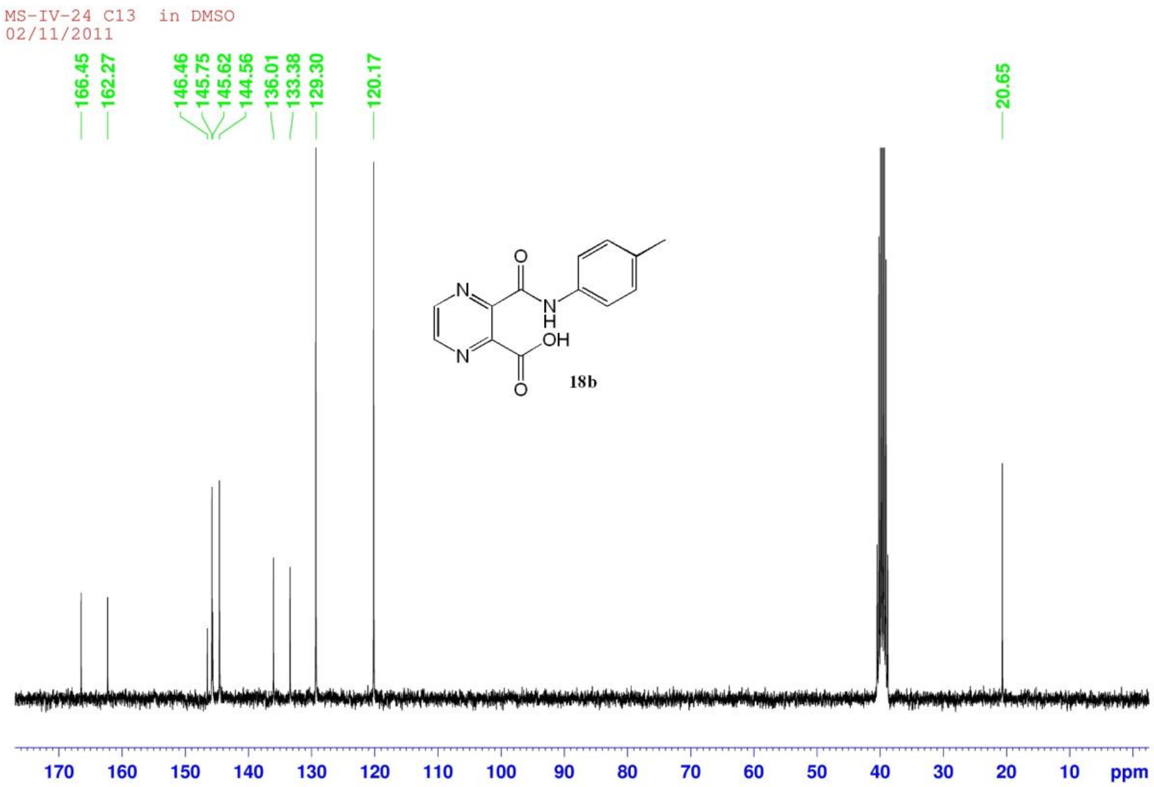

(Z)-7-((4-fluorophenyl)imino)furo[3,4-b]pyrazin-5(7H)-one (19a)

^{1}H NMR (300 MHz, $CDCl_3$)

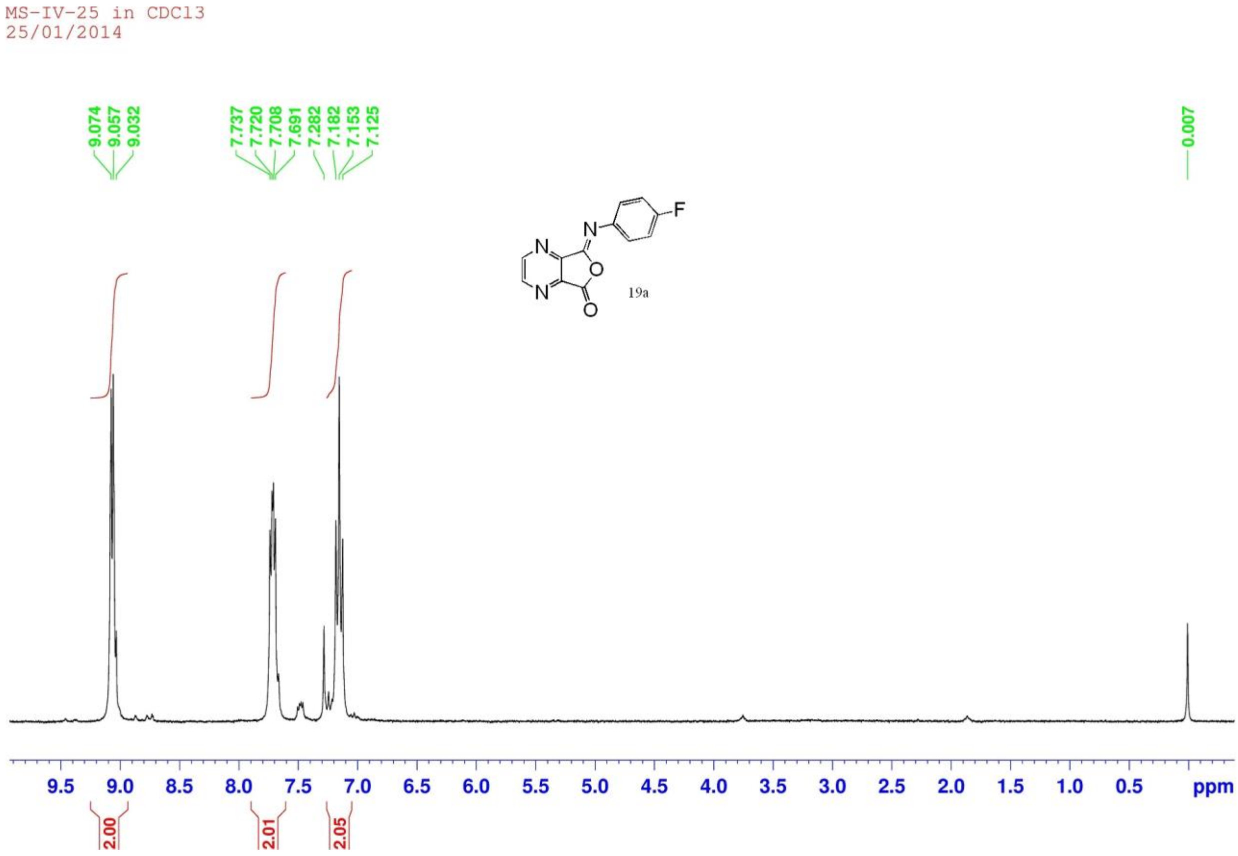

^{13}C NMR (75 MHz, $CDCl_3$)

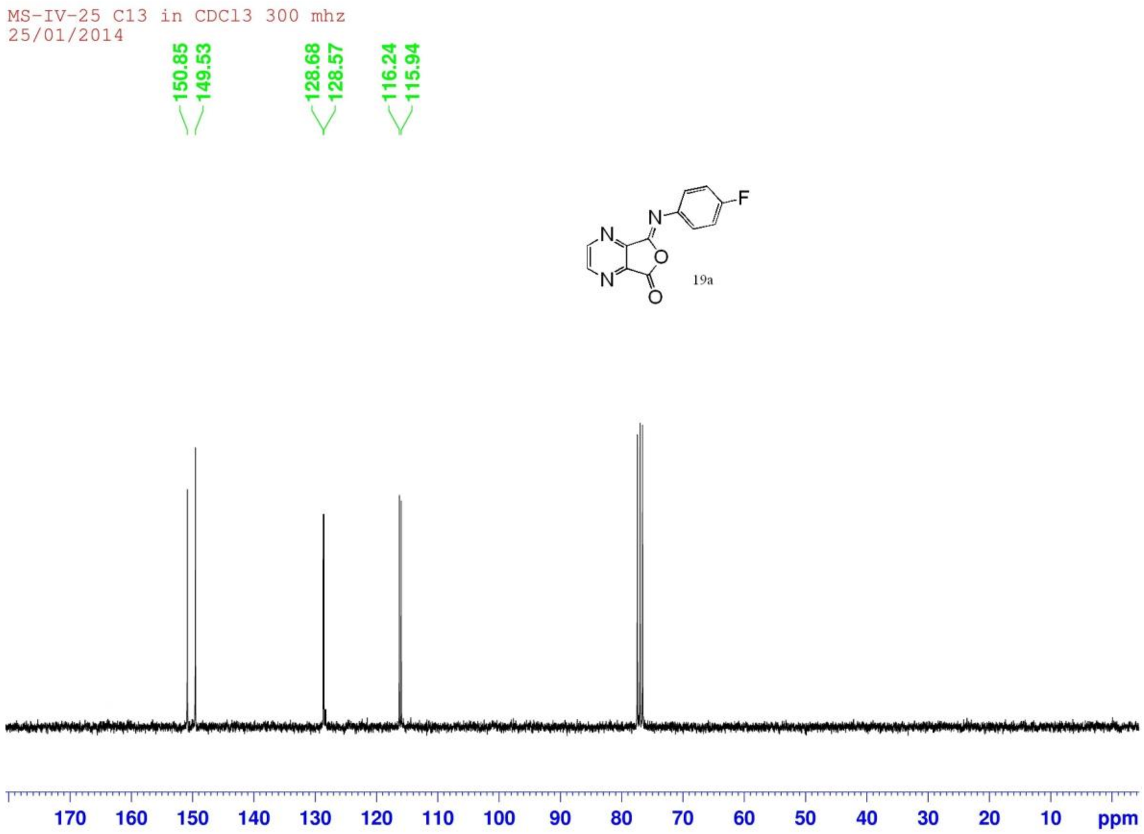

(Z)-7-(p-tolylimino)furo[3,4-b]pyrazin-5(7H)-one (19b)

^{1}H NMR (300 MHz, $CDCl_3$)

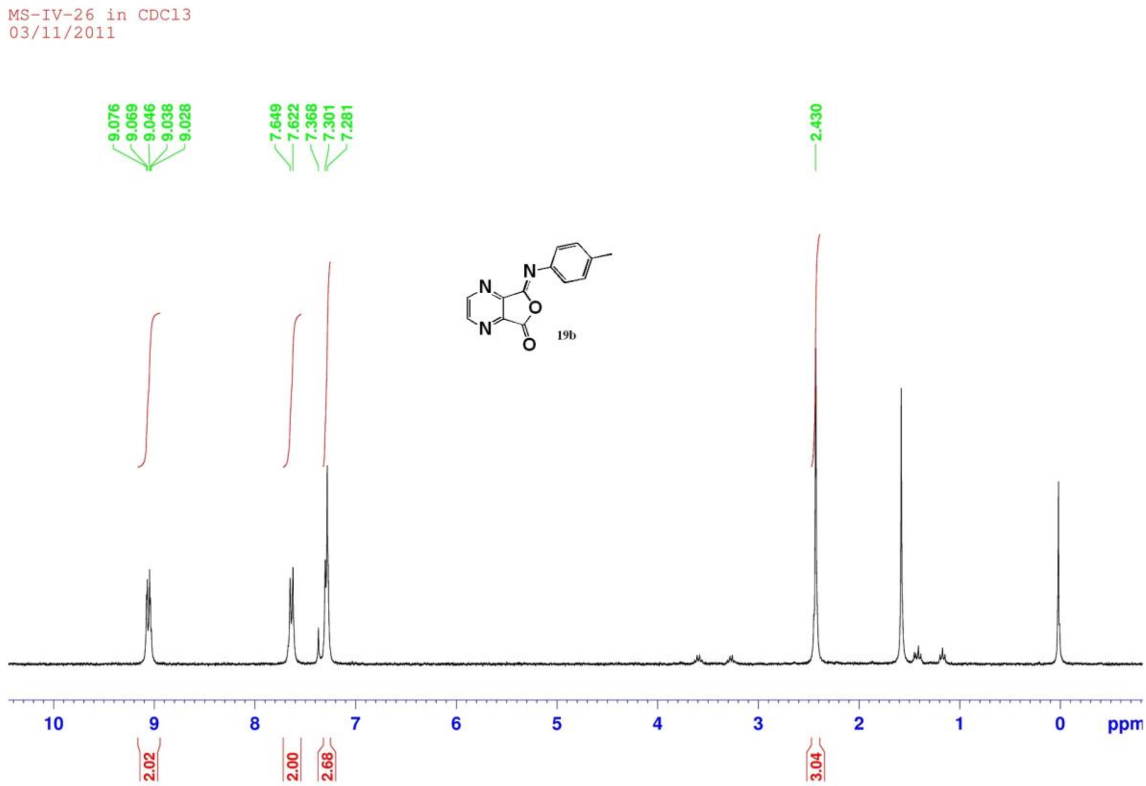

^{13}C NMR (75 MHz, $CDCl_3$)

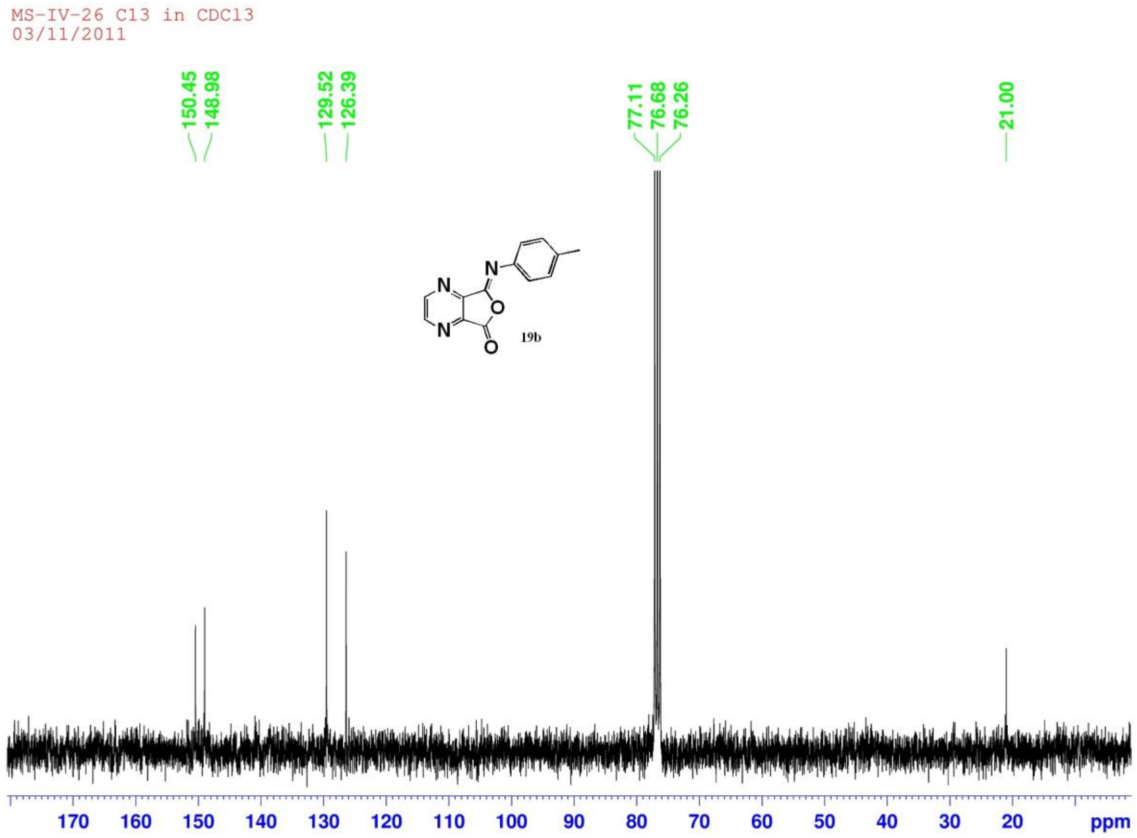

N^2-(4-fluorophenyl)-N^3-(4-(pyridin-4-yl) benzyl) pyrazine-2,3-dicarboxamide (20a)

HRMS (ES+)

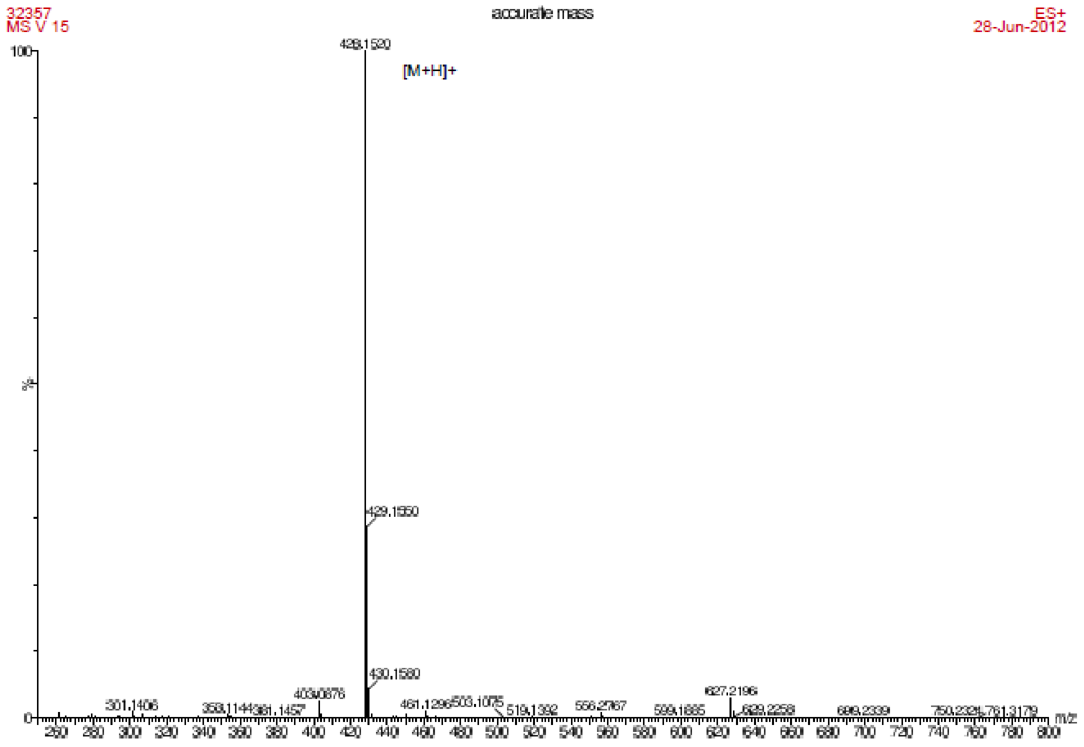

^{1}H NMR (300 MHz, DMSO)

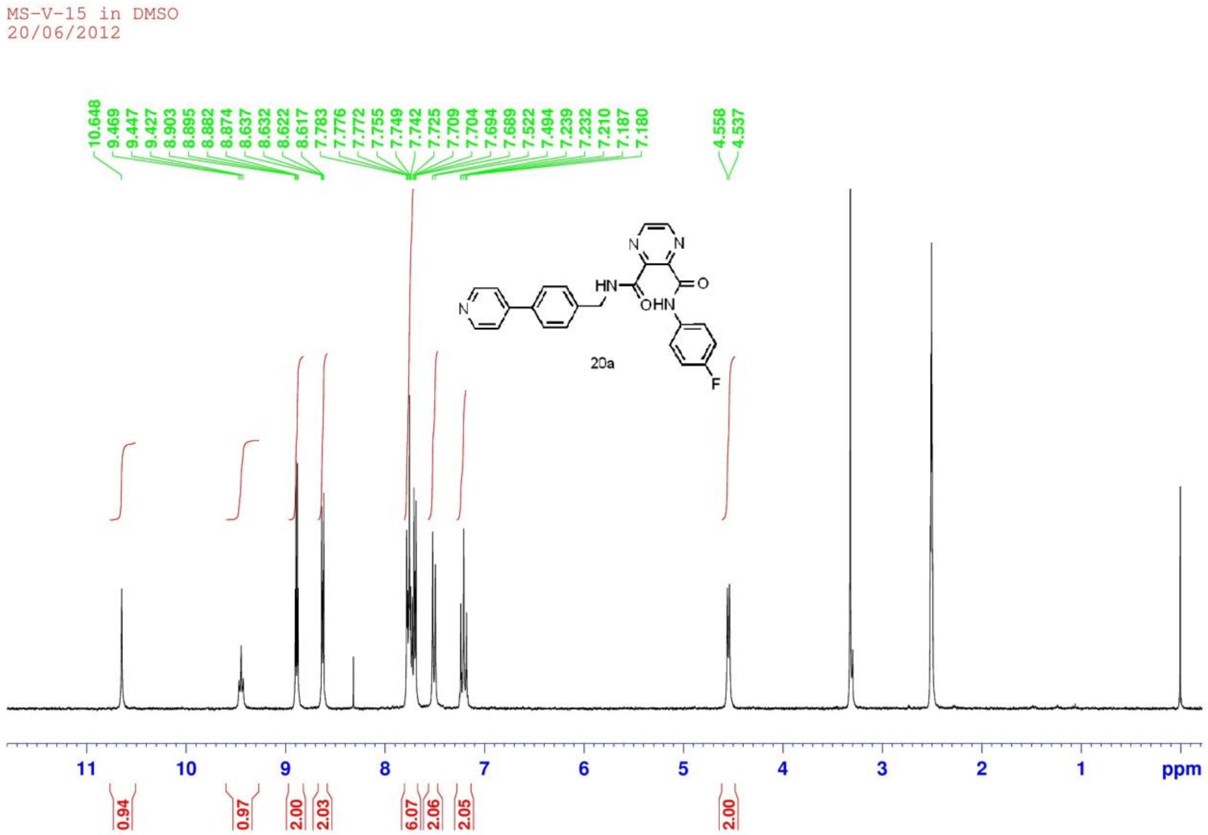

^{13}C NMR (75 MHz, DMSO)

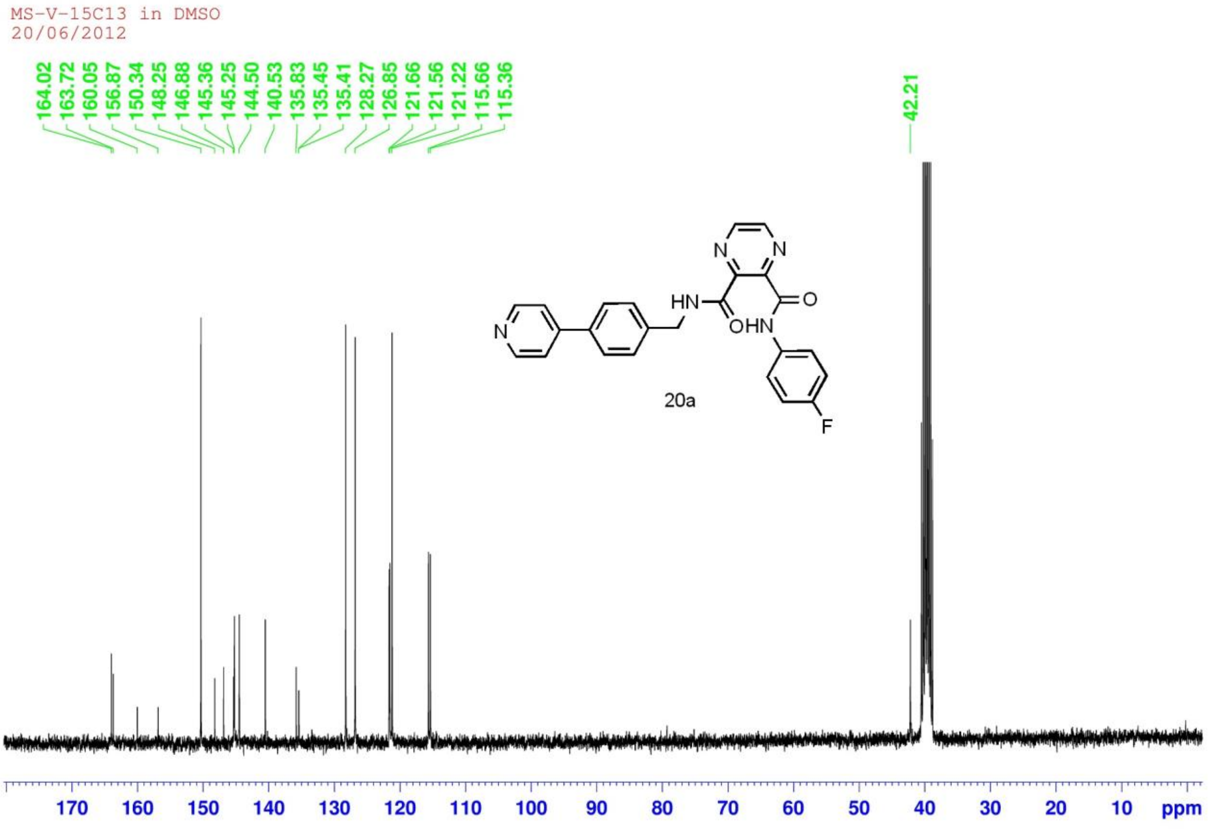

N^2-(4-fluorophenyl)-N^3-(4-methyl-2-(pyridin-3-yl)phenyl)pyrazine-2,3-dicarbox-amide (20b)

HRMS (ES+)

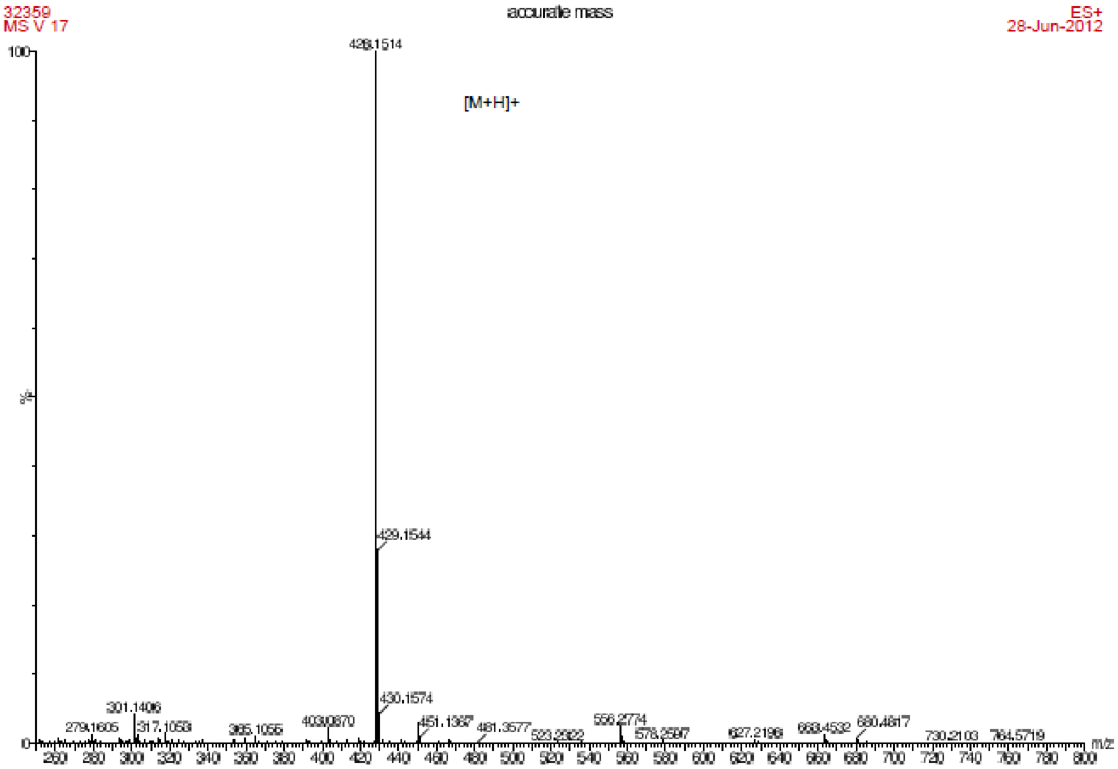

^{1}H NMR (300 MHz, $CDCl_3$)

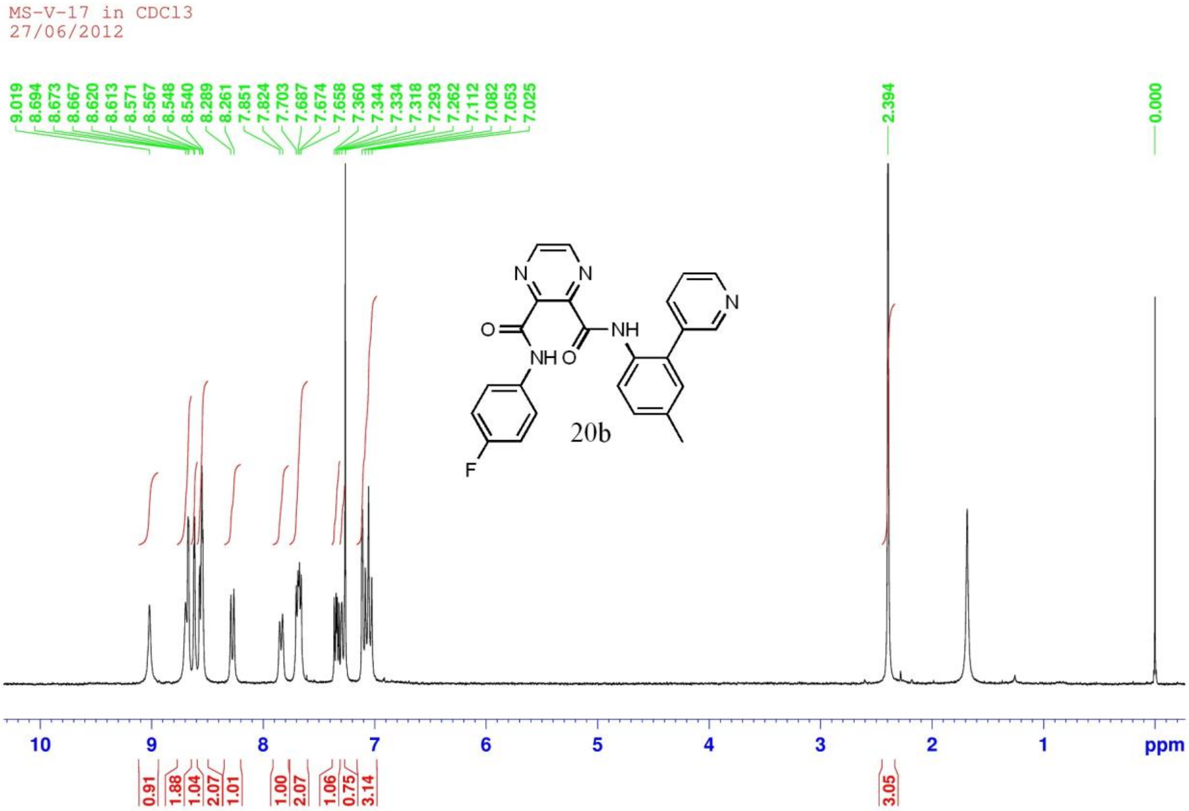

^{13}C NMR (75 MHz, $CDCl_3$)

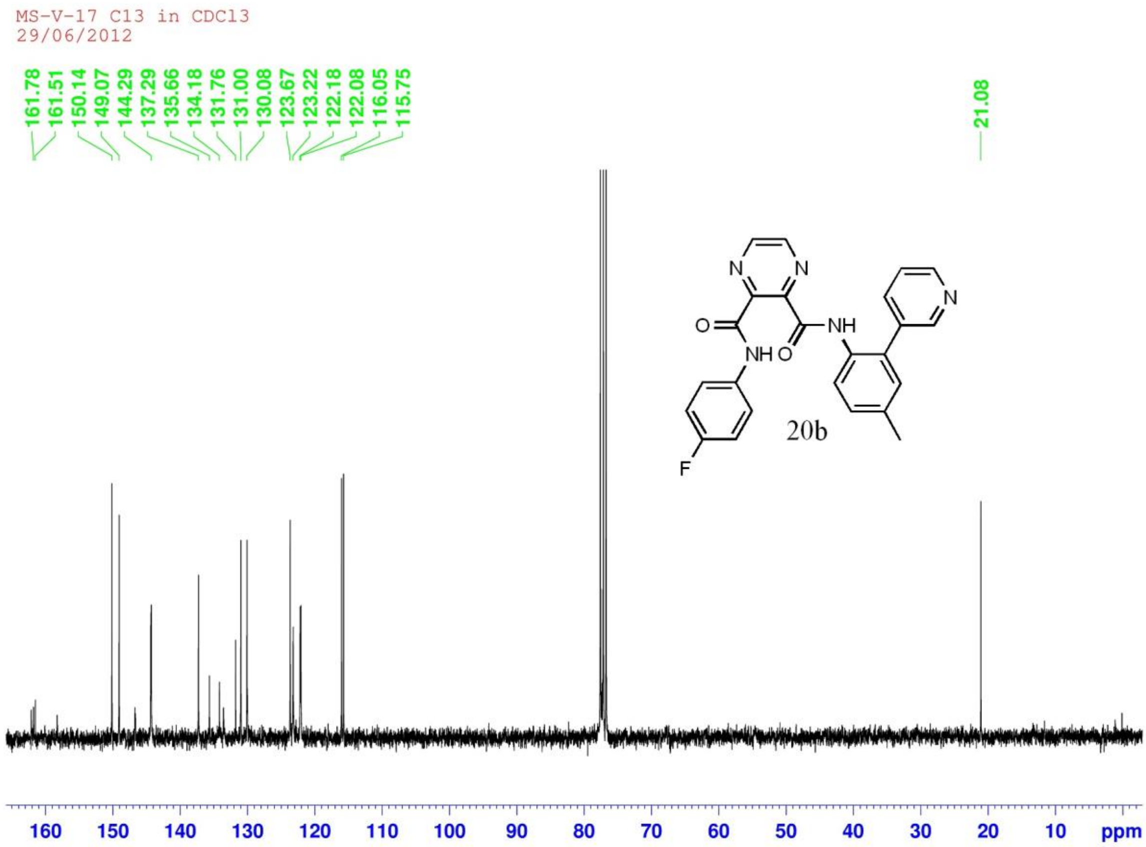

N^2-(4-fluorophenyl)-N^3-(4-(pyridin-3-yl) benzyl) pyrazine-2,3-dicarboxamide (20c)

HRMS (ES+)

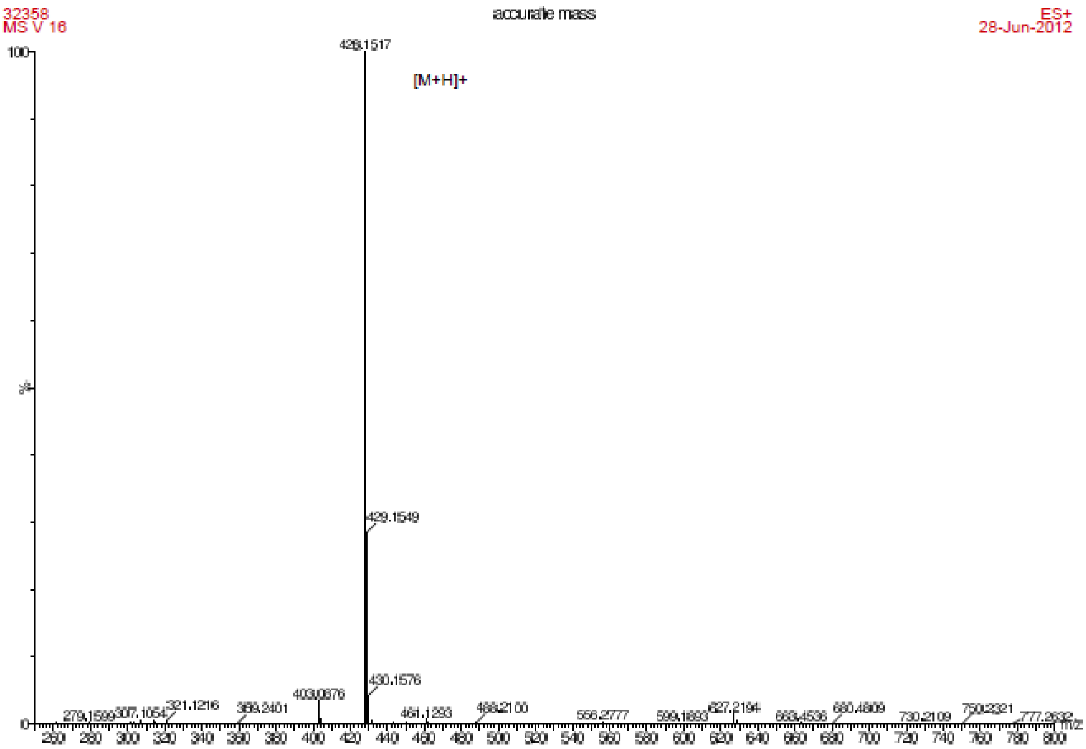

^{1}H NMR (300 MHz, $CDCl_3$)

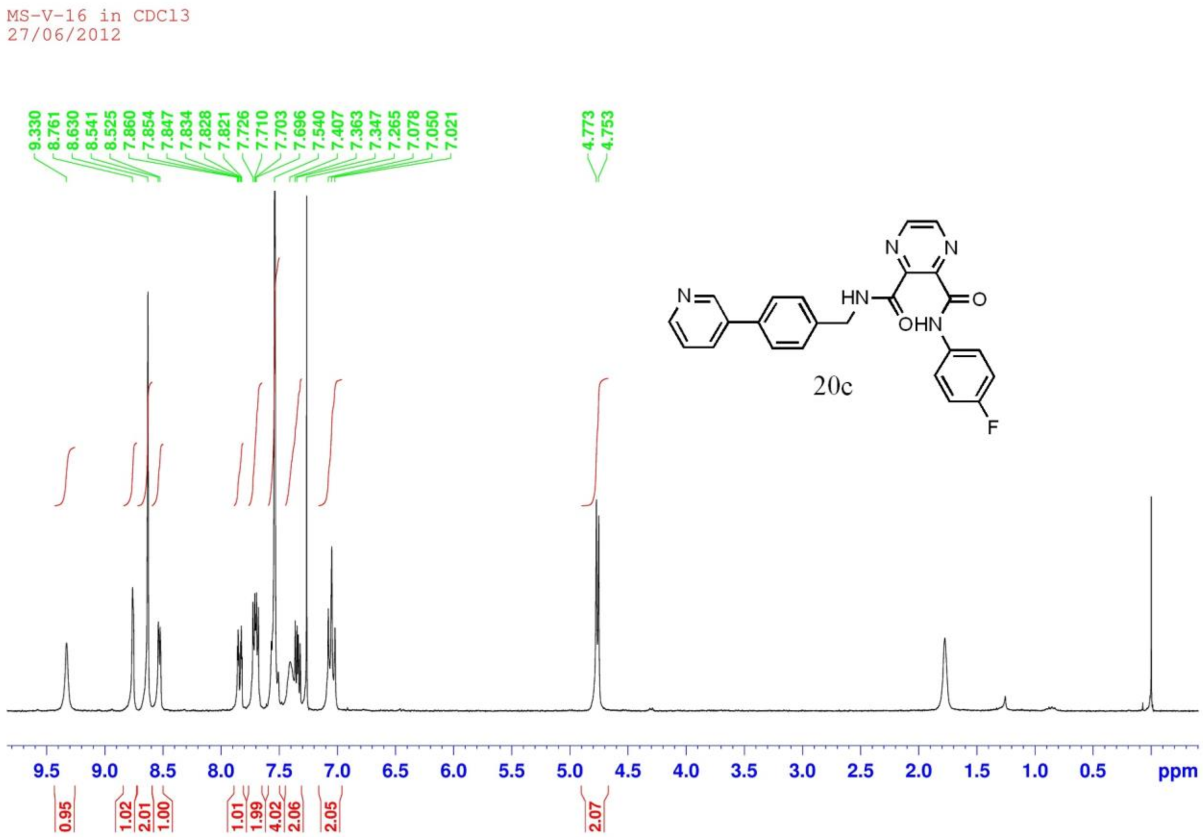

^{13}C NMR (75 MHz, $CDCl_3$)

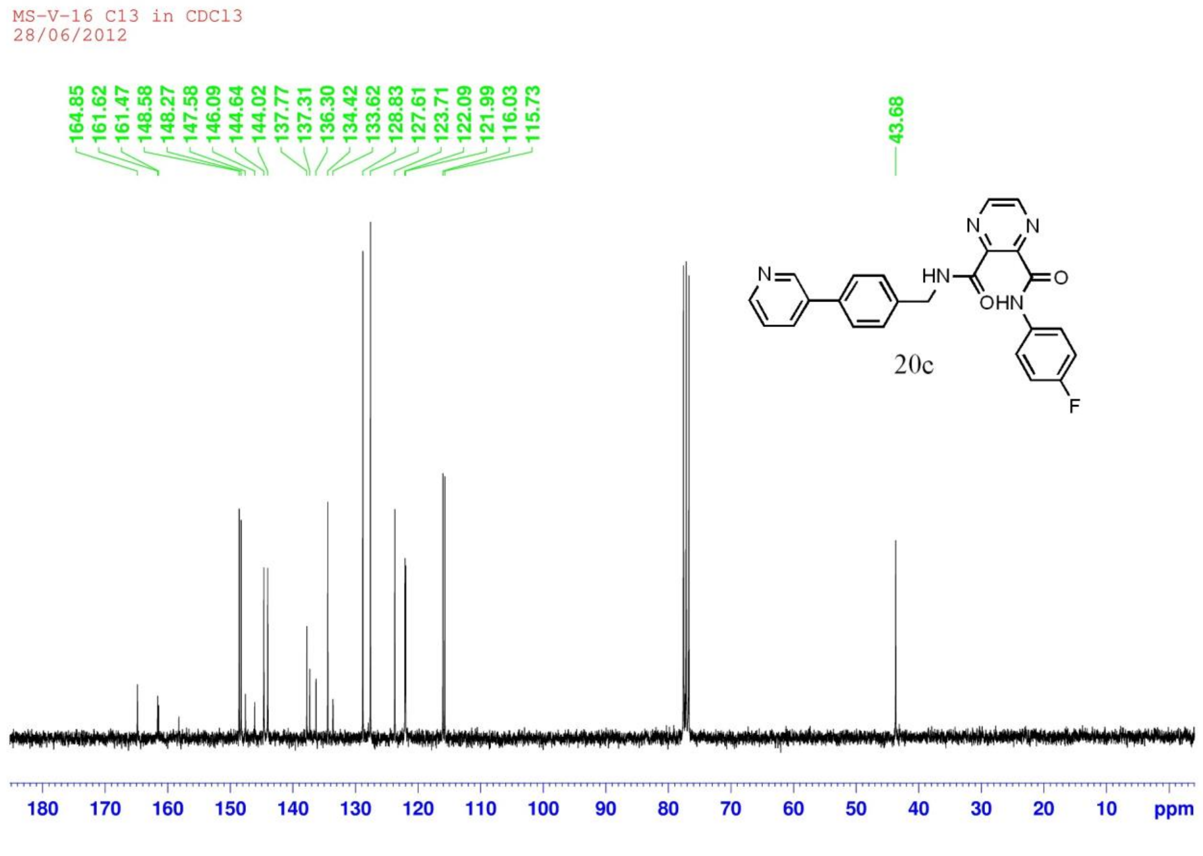

N^2-(4-fluorophenyl)-N^3-(2-(pyridin-4-yl) phenyl) pyrazine-2,3-dicarboxamide (20d)

HRMS (ES+)

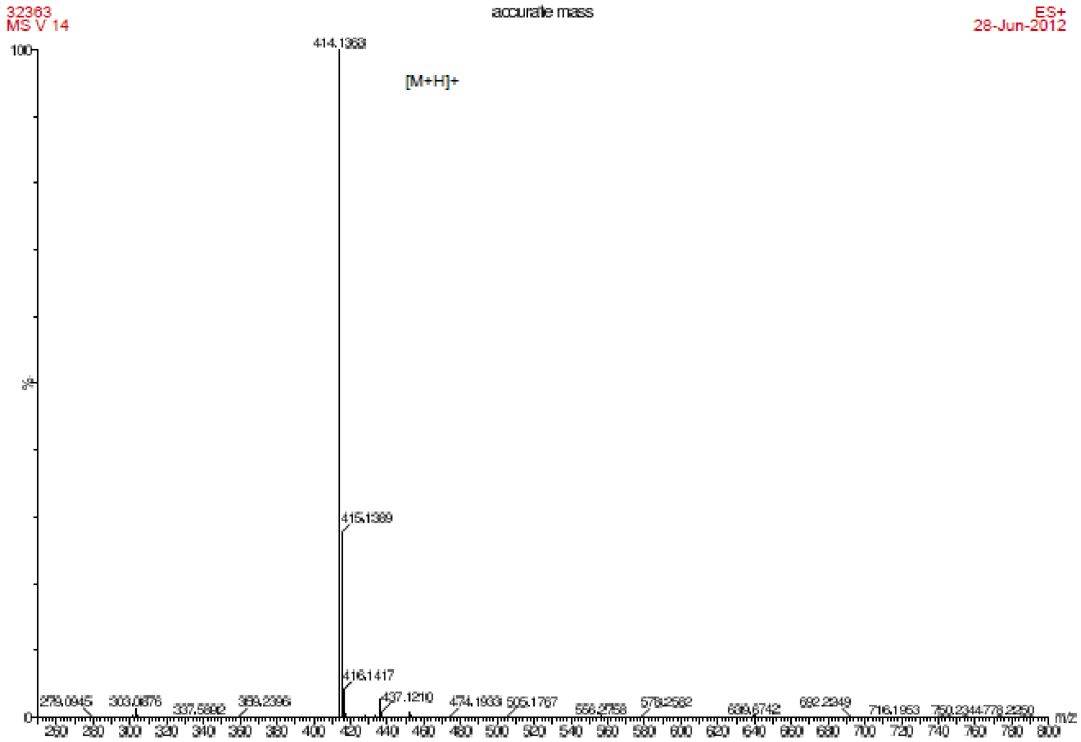

^{1}H NMR (300 MHz, DMSO)

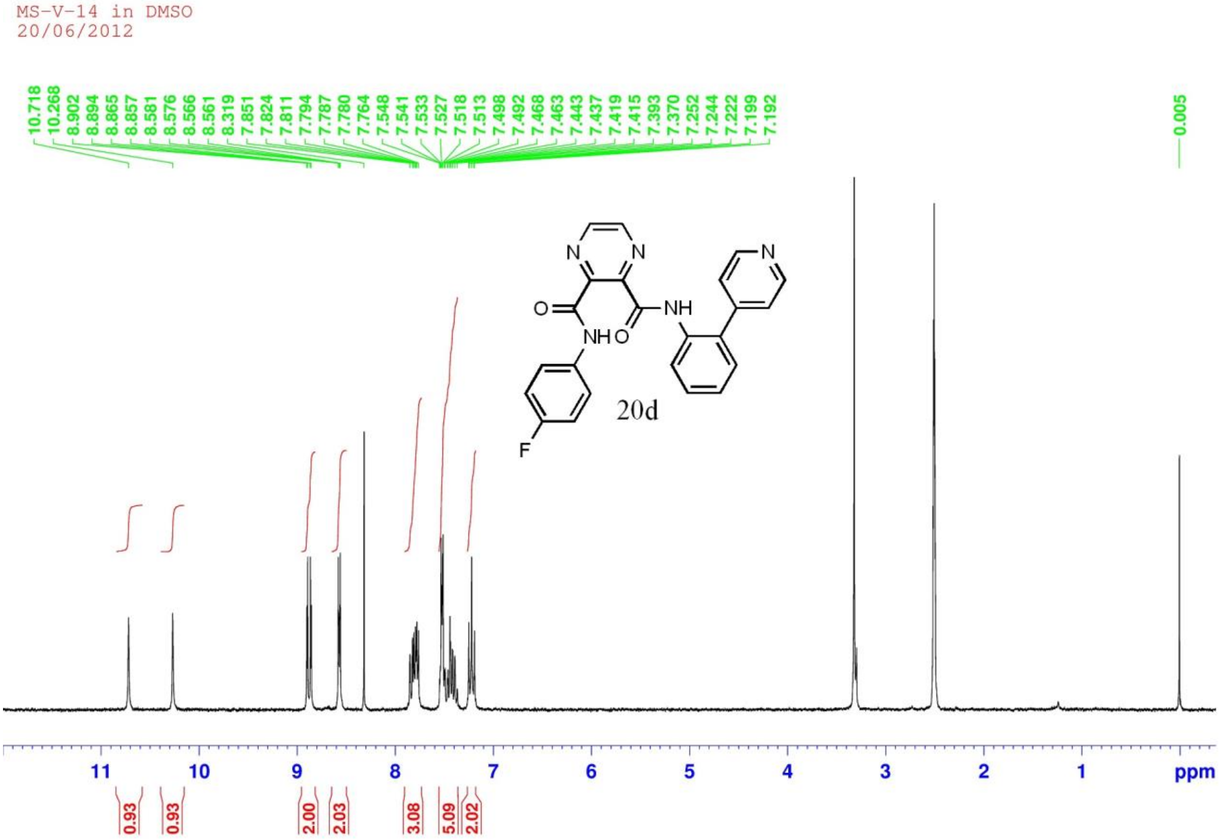

^{13}C NMR (75 MHz, DMSO)

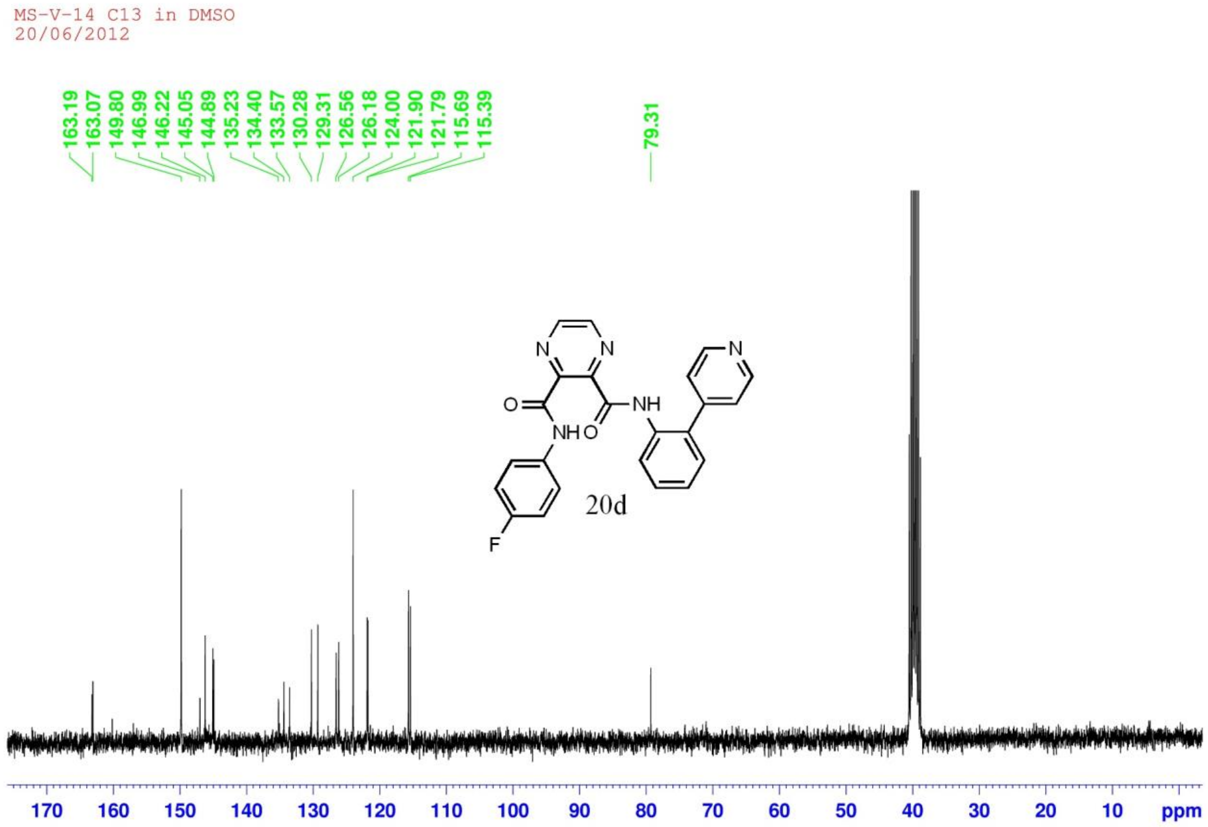

***N*²-(4-fluorophenyl)-*N*³-(2-(pyridin-3-yl) phenyl) pyrazine-2,3-dicarboxamide (20e)**

HRMS (ES+)

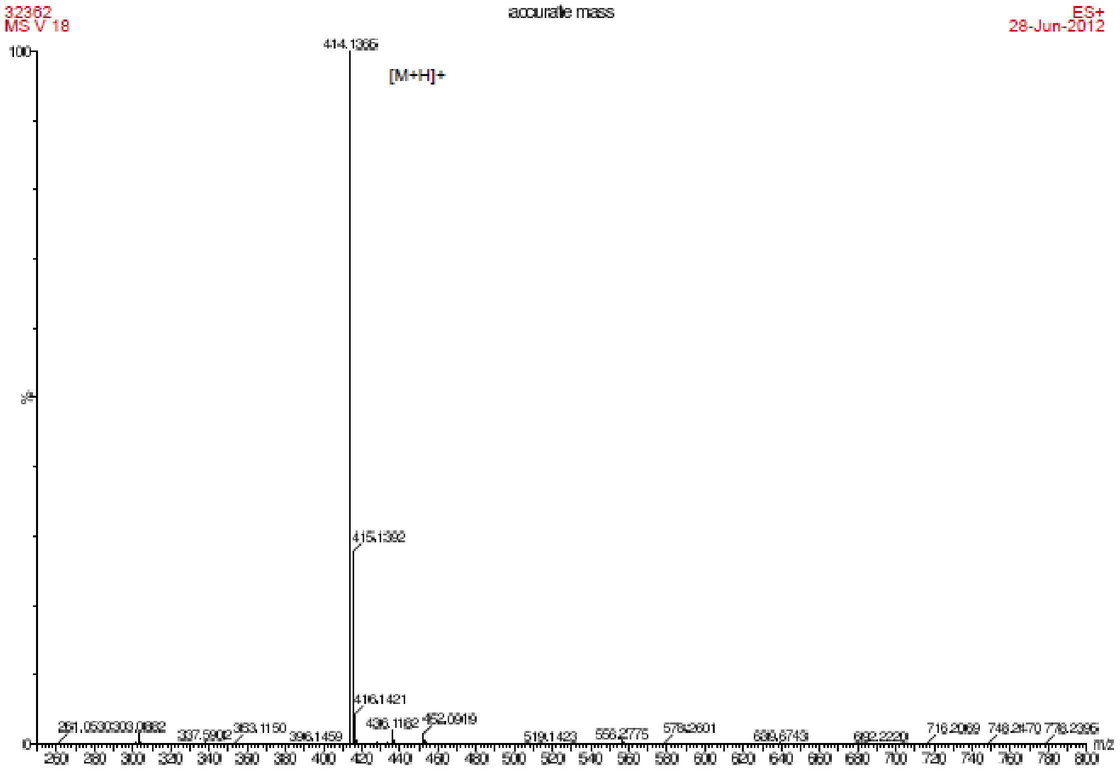

^{1}H NMR (300 MHz, $CDCl_3$)

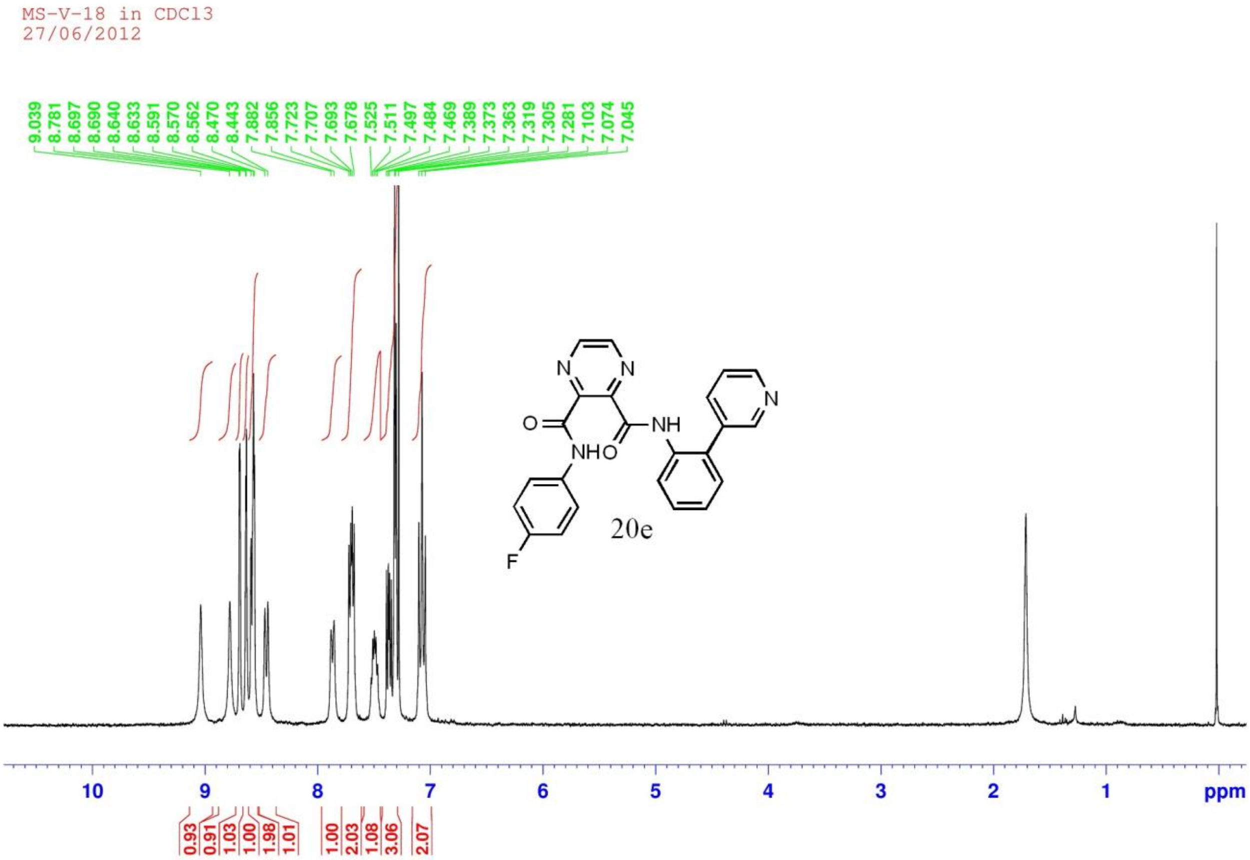

^{13}C NMR (75 MHz, $CDCl_3$)

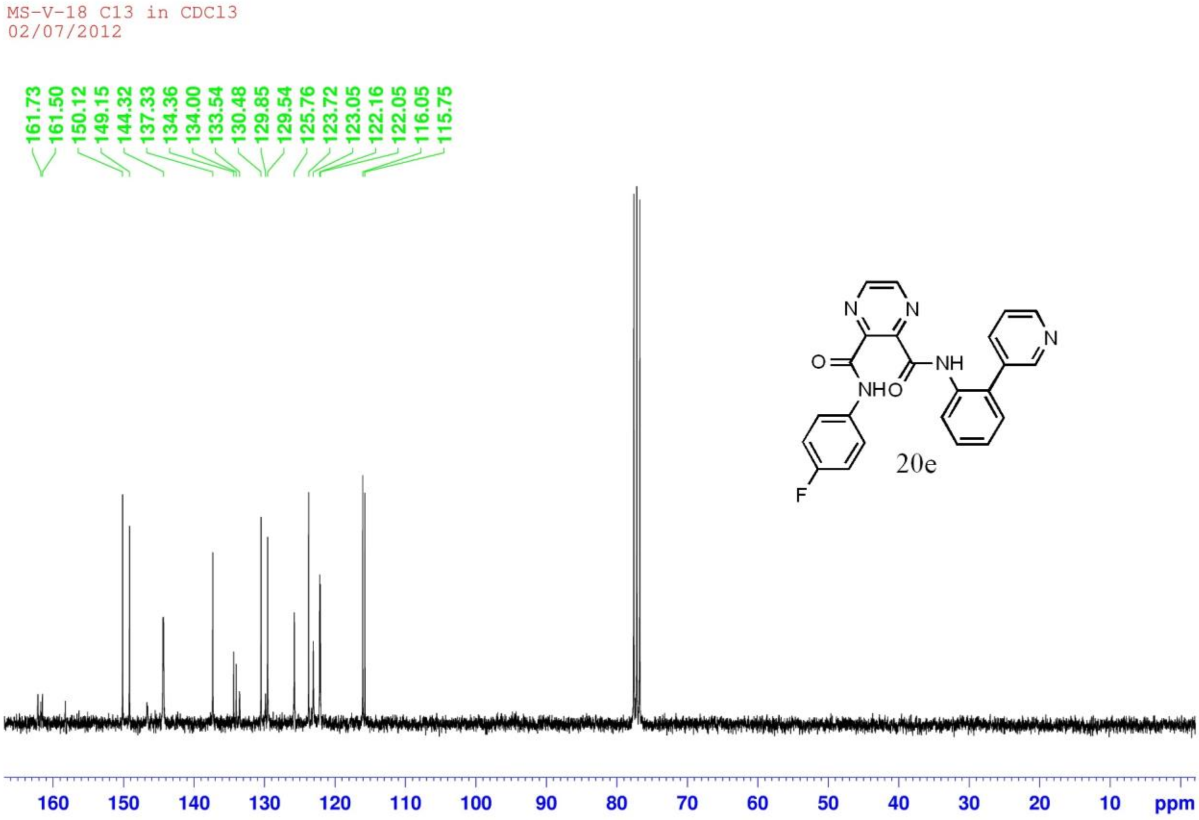

N^2-(4-fluorophenyl)-N^3-(2-(pyridin-4-yl) benzyl) pyrazine-2,3-dicarboxamide (20f)

HRMS (ES+)

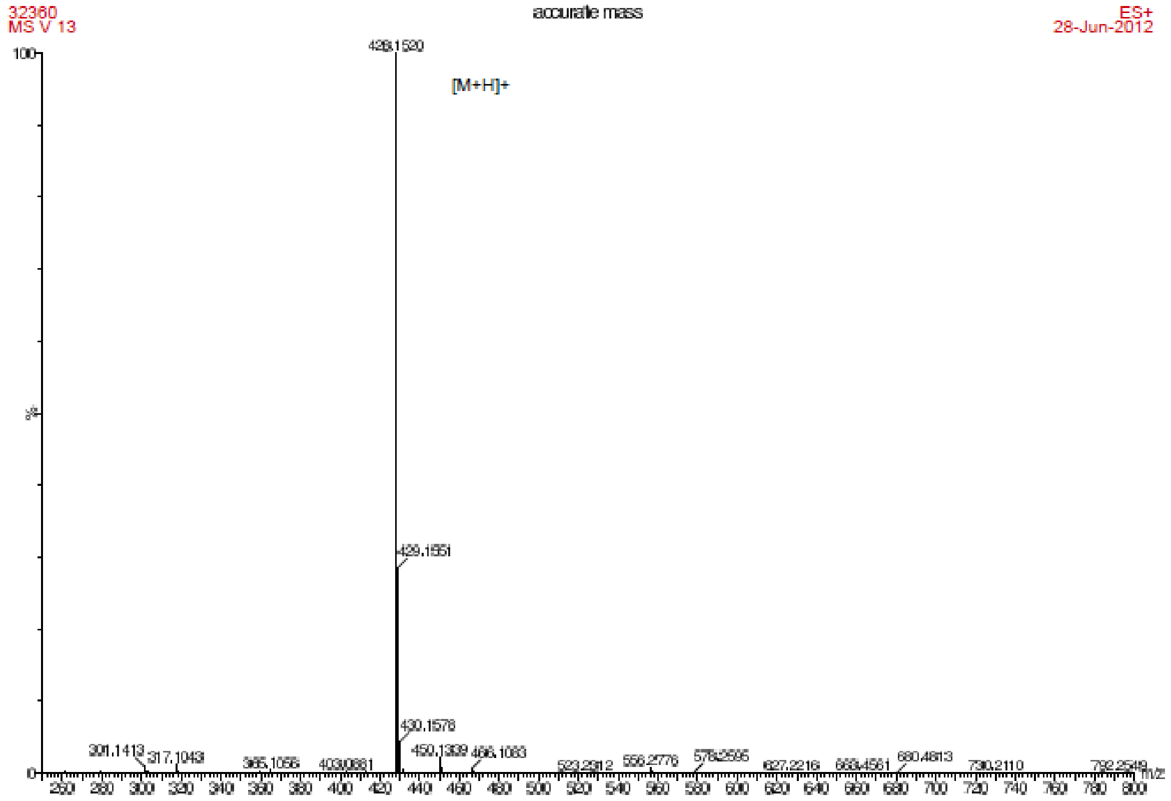

^{1}H NMR (600 MHz, DMSO)

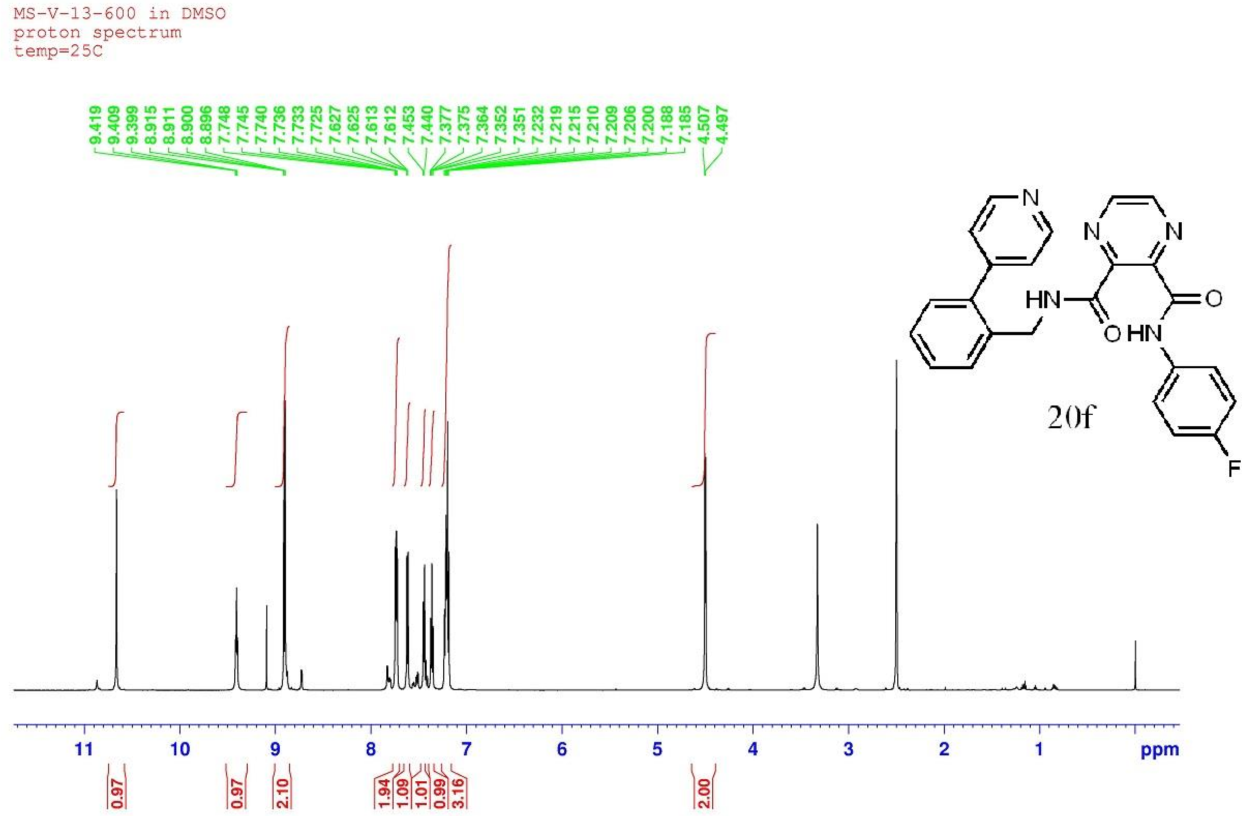

^{13}C NMR (150 MHz, DMSO)

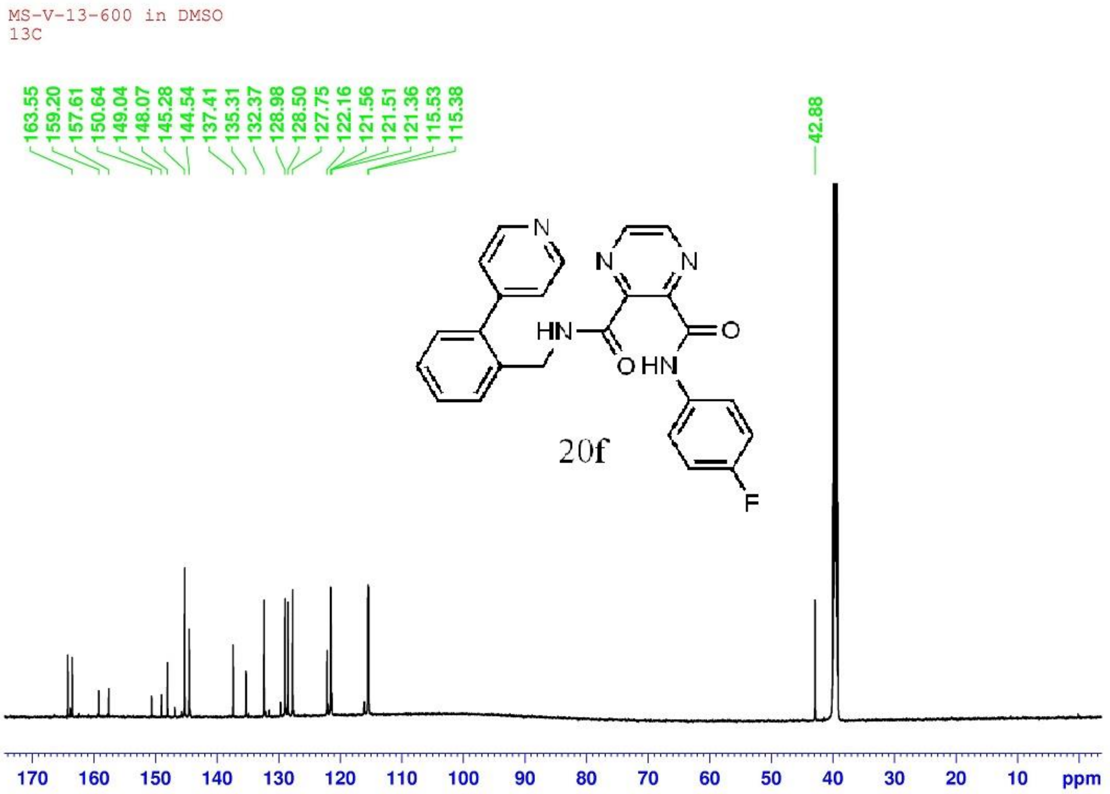

***N*²-(4-fluorophenyl)-*N*³-(4-methyl-2-(pyridin-4-yl)phenyl)pyrazine-2,3-dicarboxamide (20g)**

HRMS (ES+)

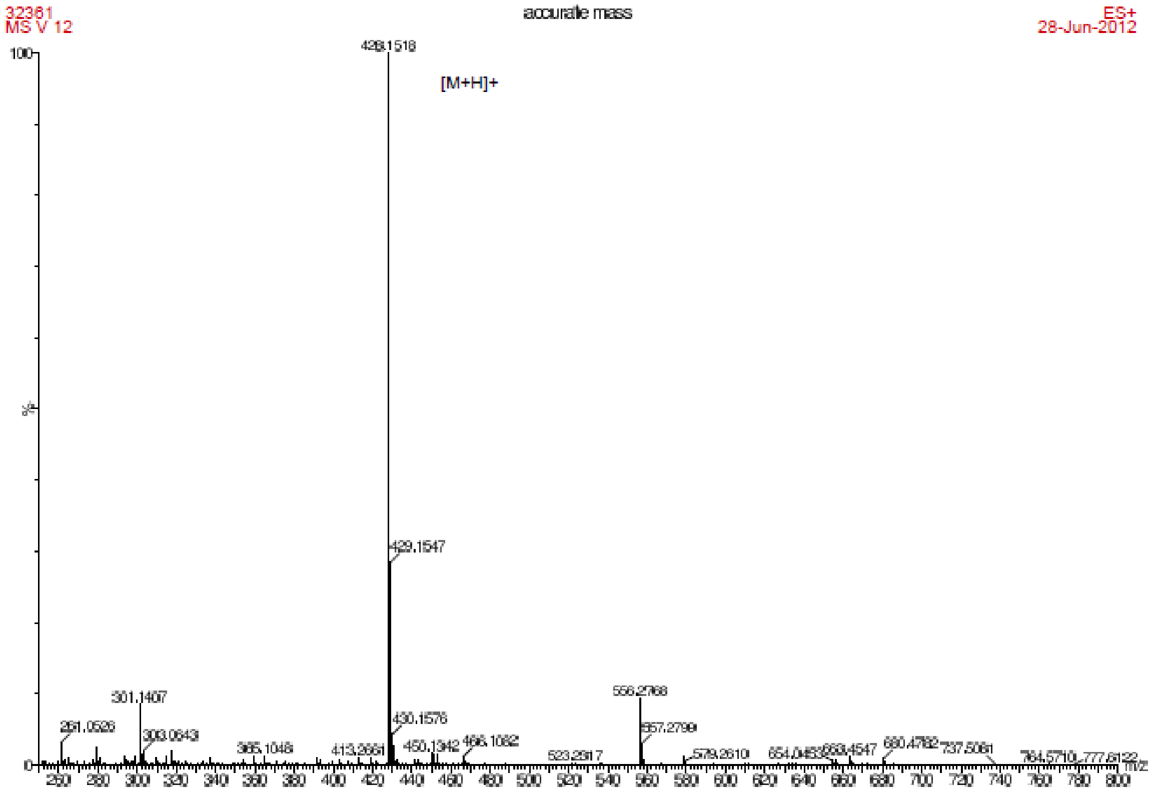

^{1}H NMR (300 MHz, $CDCl_3$)

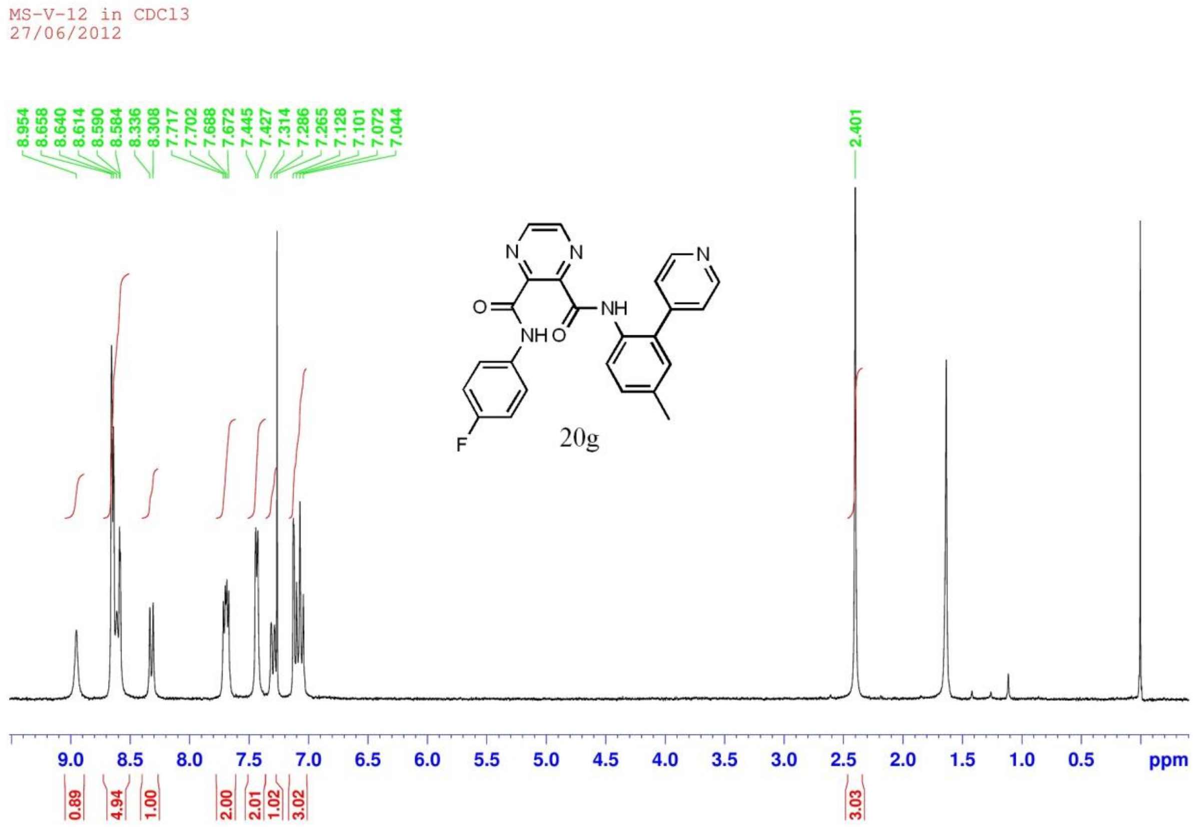

^{13}C NMR (75 MHz, $CDCl_3$)

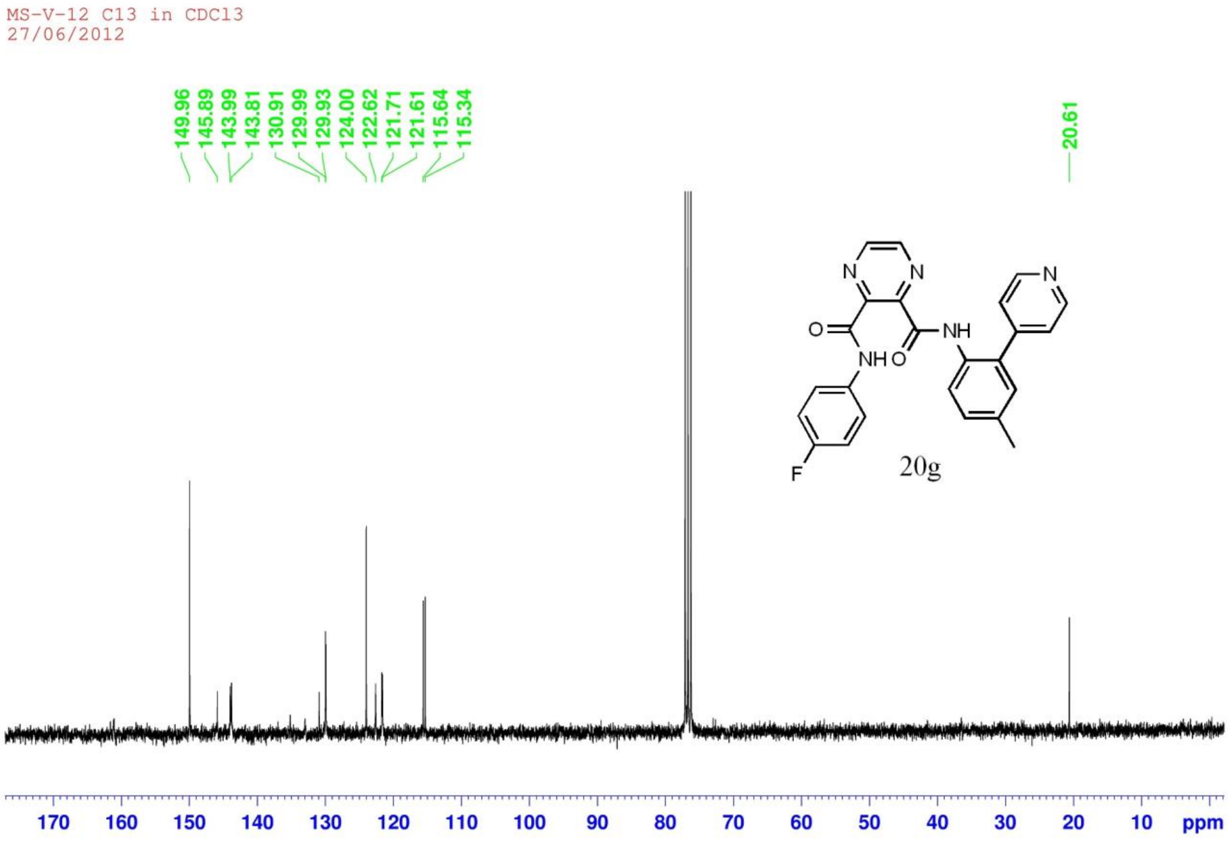

***N*²-(4-(pyridin-4-yl) benzyl)-*N*³-(p-tolyl) pyrazine-2,3-dicarboxamide (20h)**

HRMS (ES+)

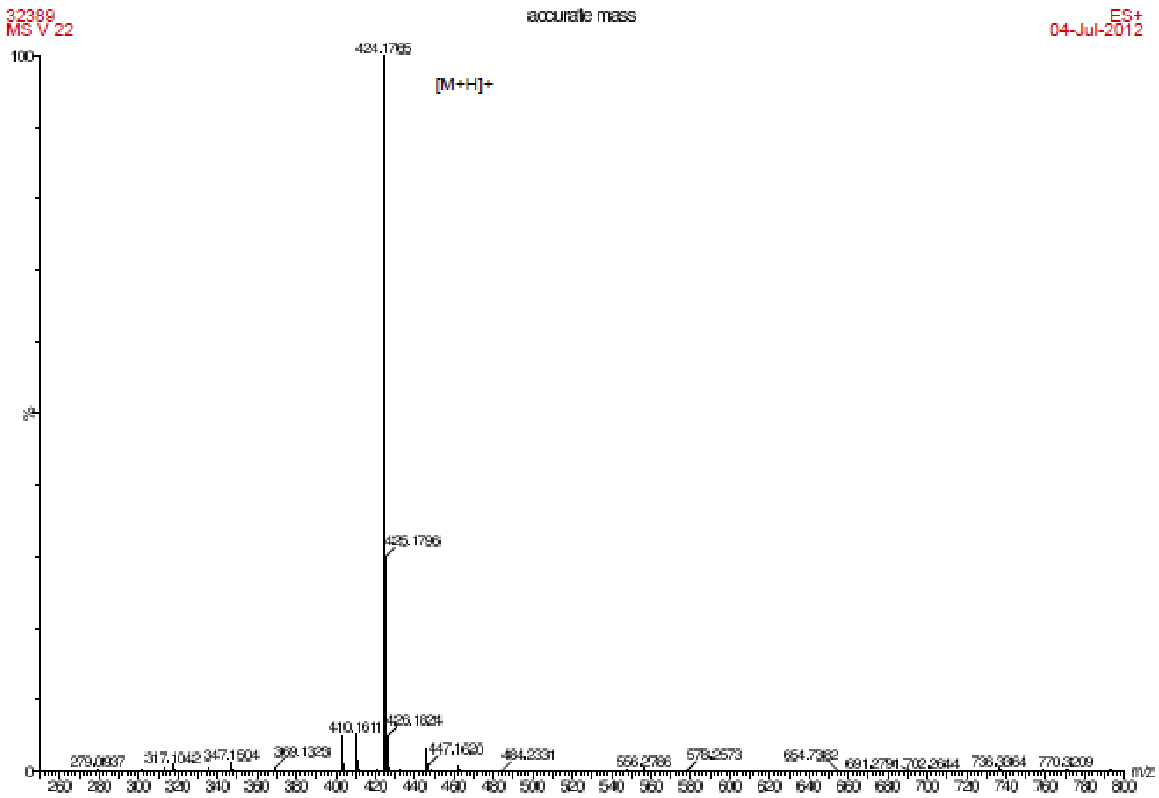

¹H NMR (300 MHz, $CDCl_3$)

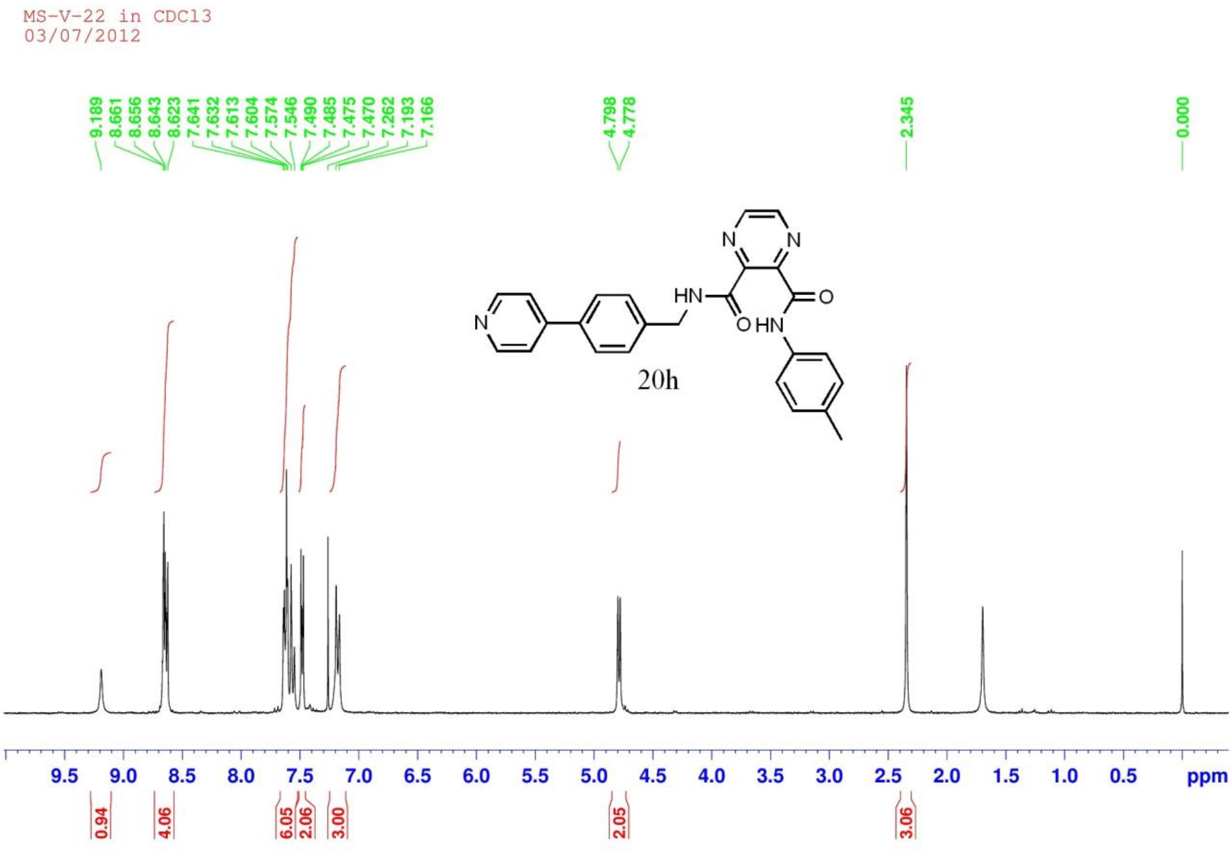

^{13}C NMR (75 MHz, $CDCl_3$)

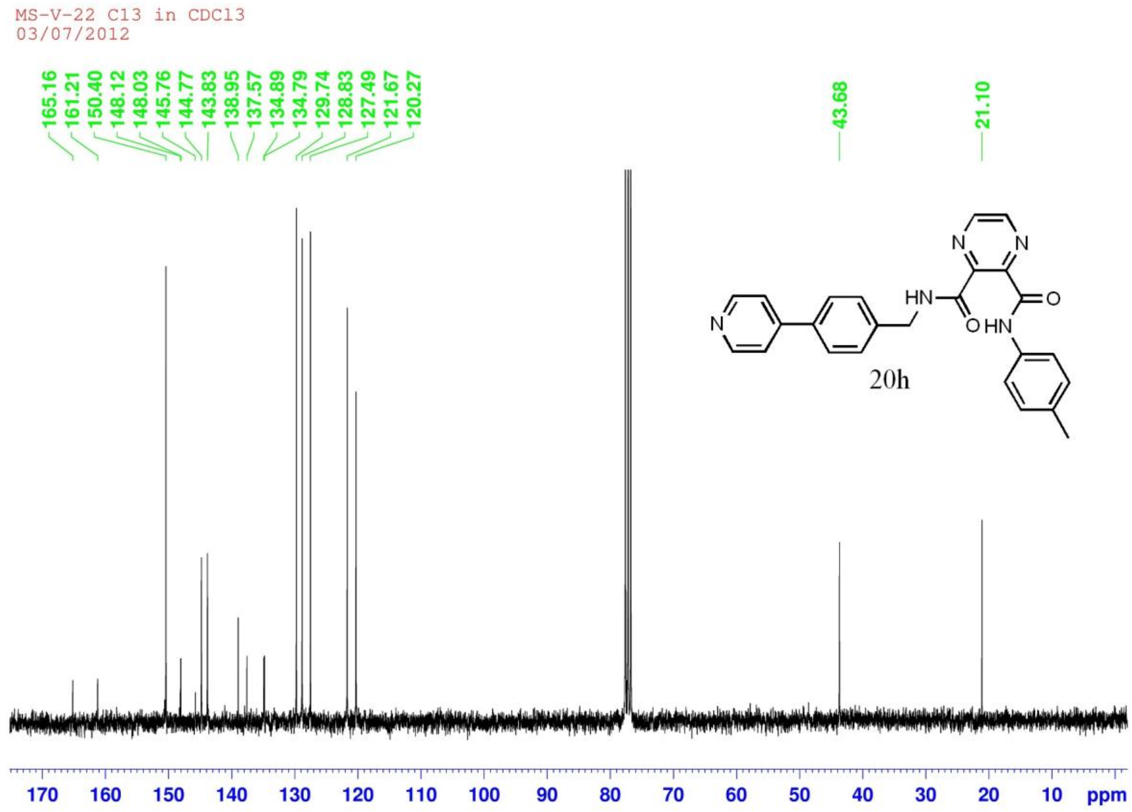

***N*[2]-(4-methyl-2-(pyridin-3-yl) phenyl)-*N*[3]-(p-tolyl) pyrazine-2,3-dicarboxamide (20i)**

HRMS (ES+)

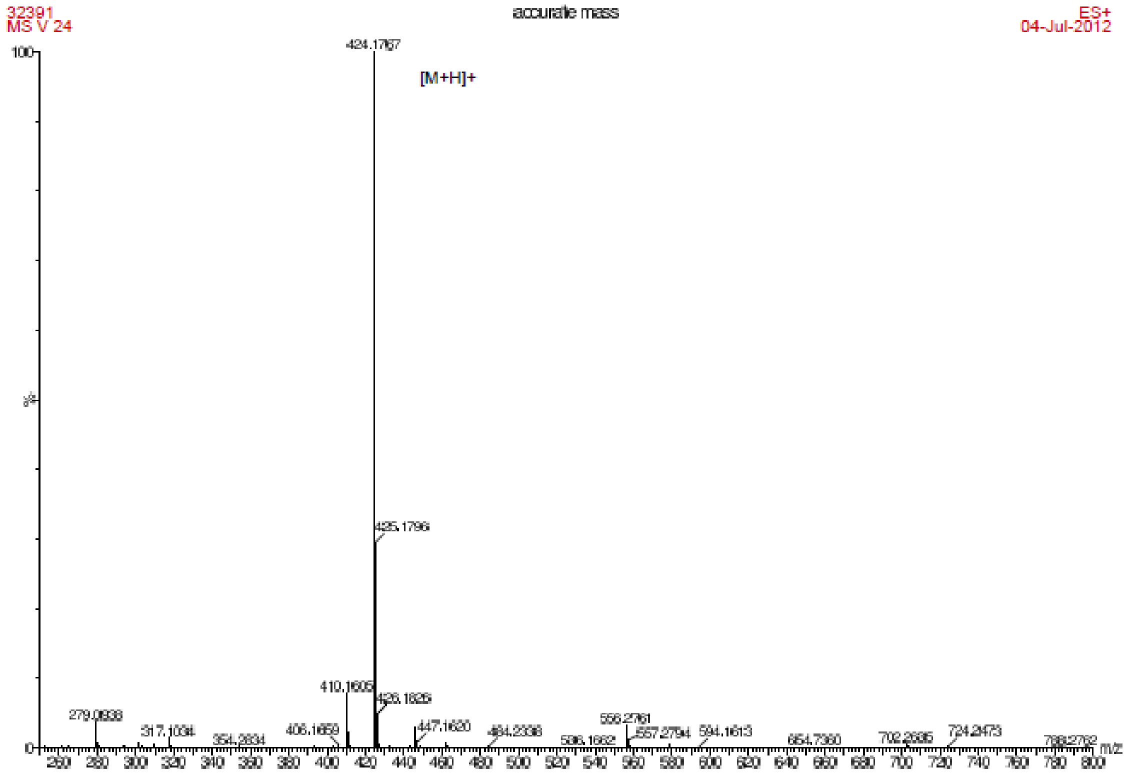

^{1}H NMR (300 MHz, $CDCl_3$)

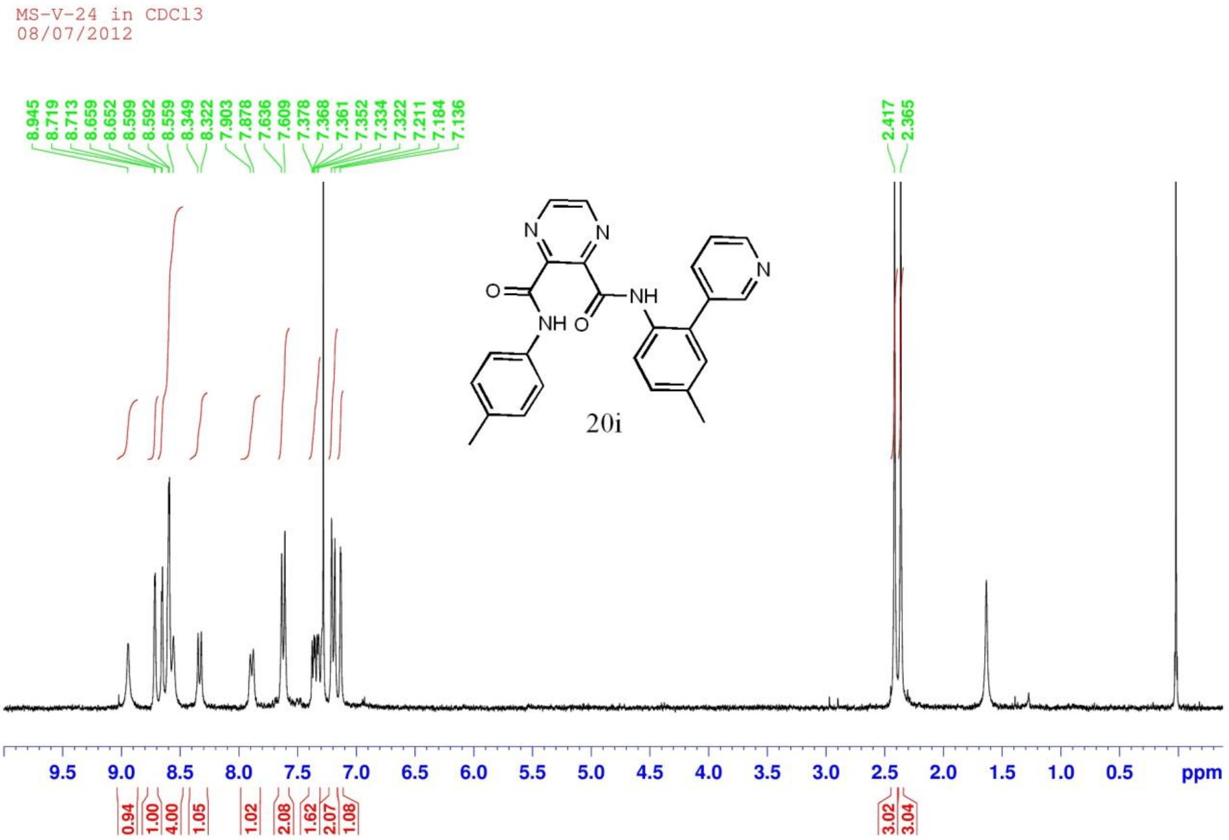

^{13}C NMR (75 MHz, $CDCl_3$)

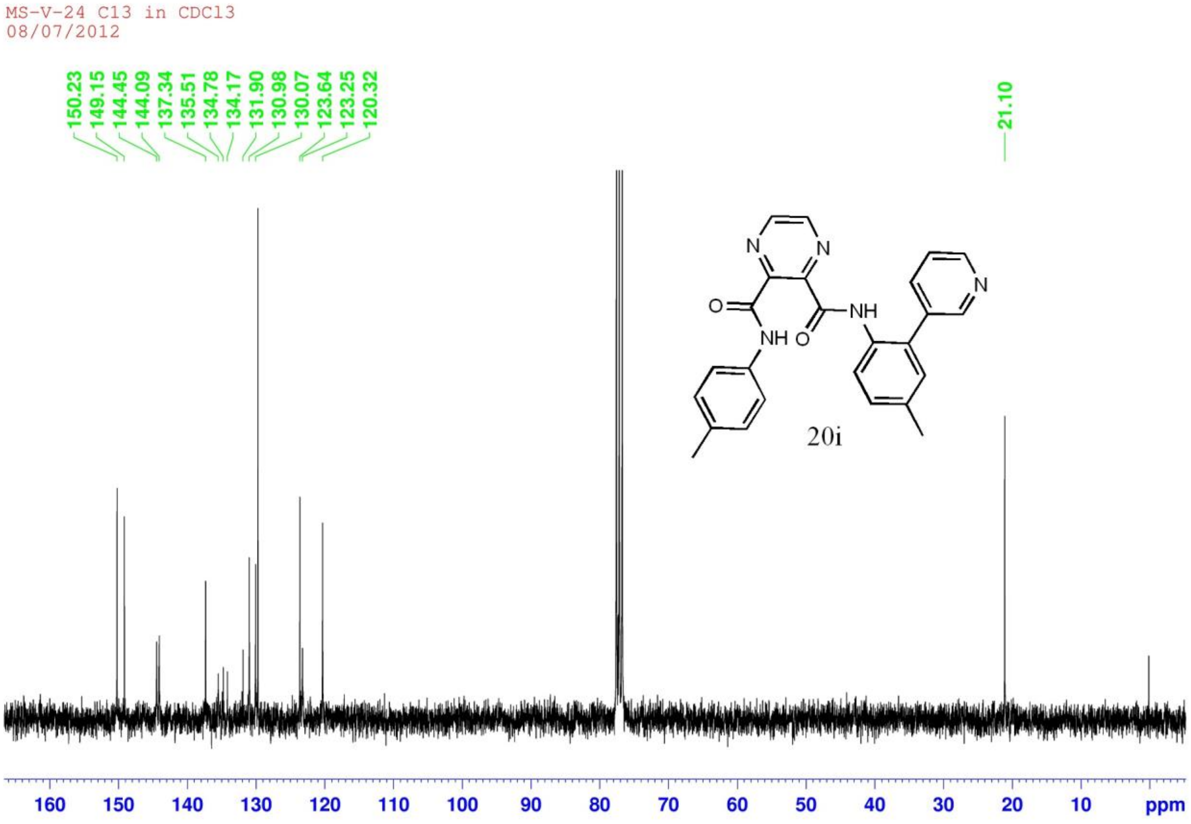

N^2-(4-(pyridin-3-yl) benzyl)-N^3-(p-tolyl) pyrazine-2,3-dicarboxamide (20j)

HRMS (ES+)

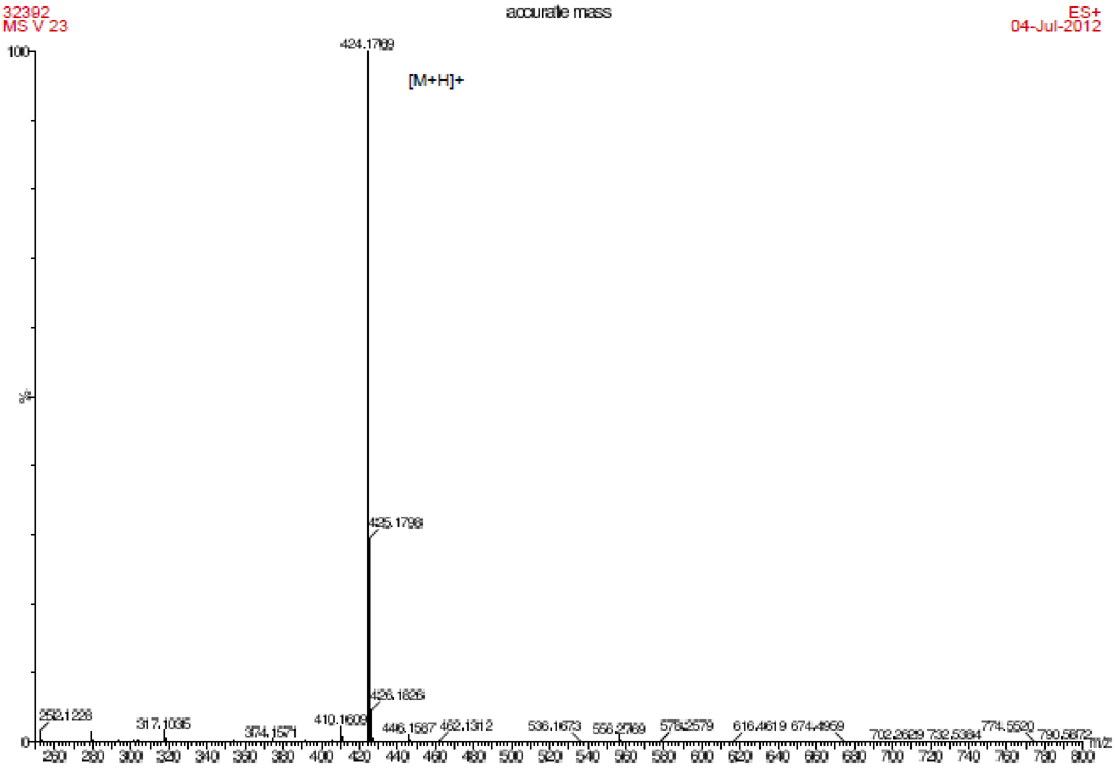

^{1}H NMR (300 MHz, $CDCl_3$)

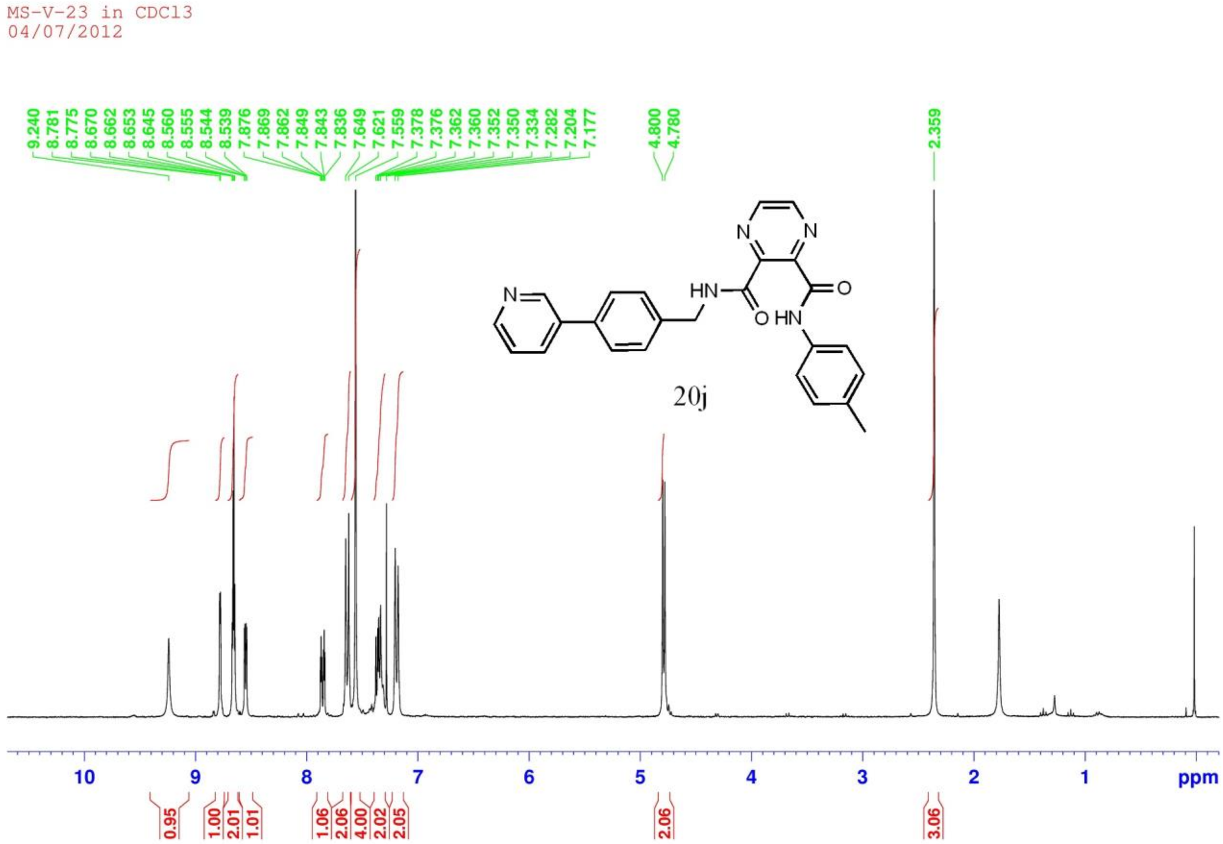

^{13}C NMR (75 MHz, $CDCl_3$)

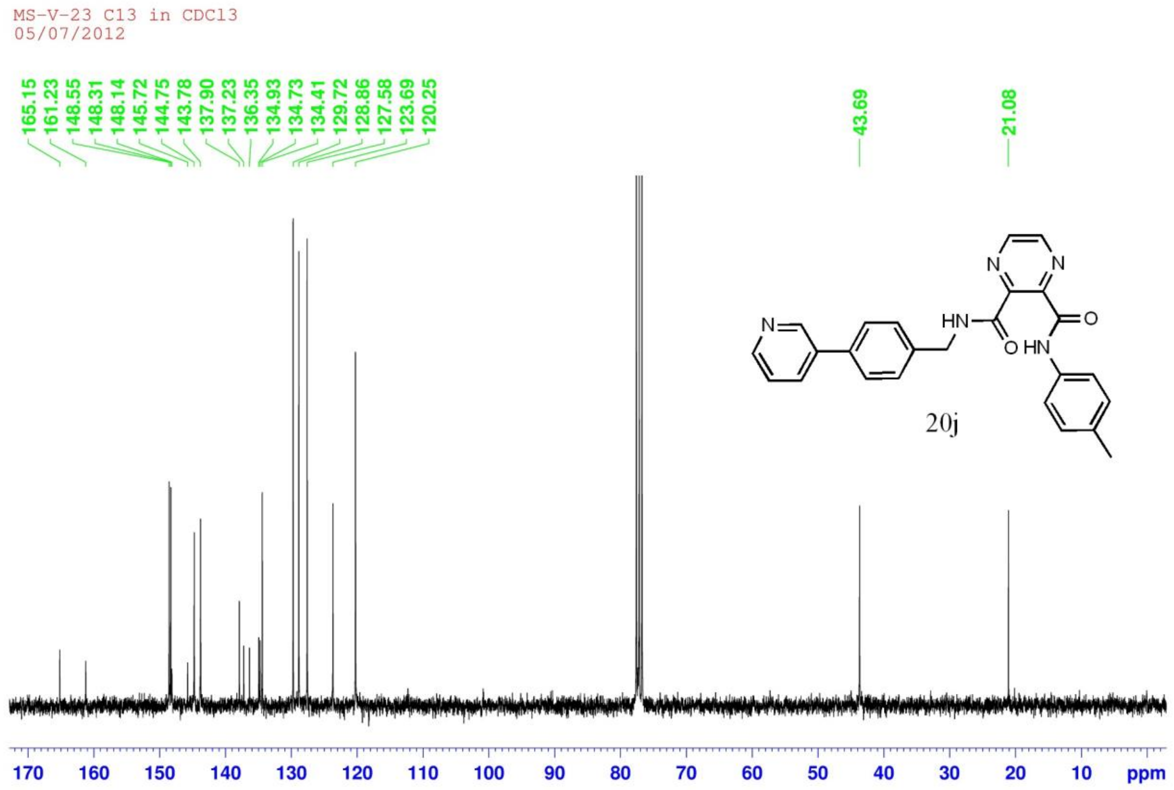

4.4.1.7.11 N^2-(2-(pyridin-4-yl) phenyl)-N^3-(p-tolyl) pyrazine-2,3-dicarboxamide (20k)

HRMS (ES+)

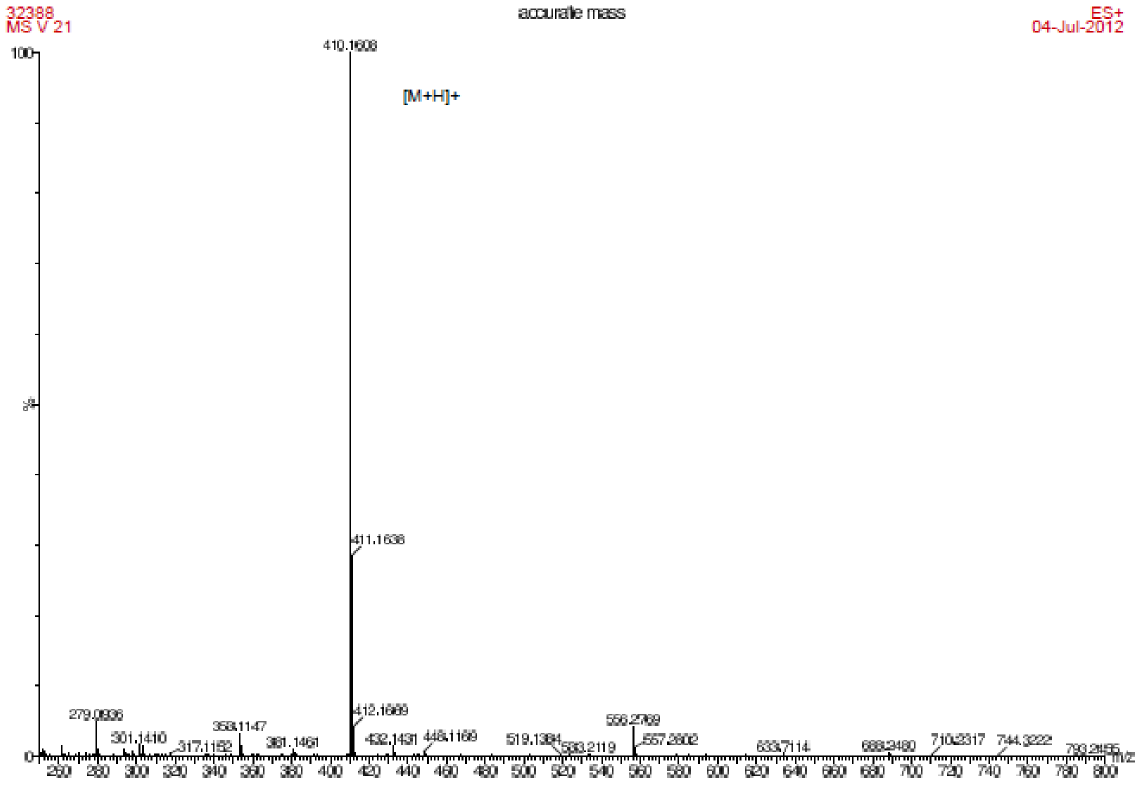

^{1}H NMR (300 MHz, $CDCl_3$)

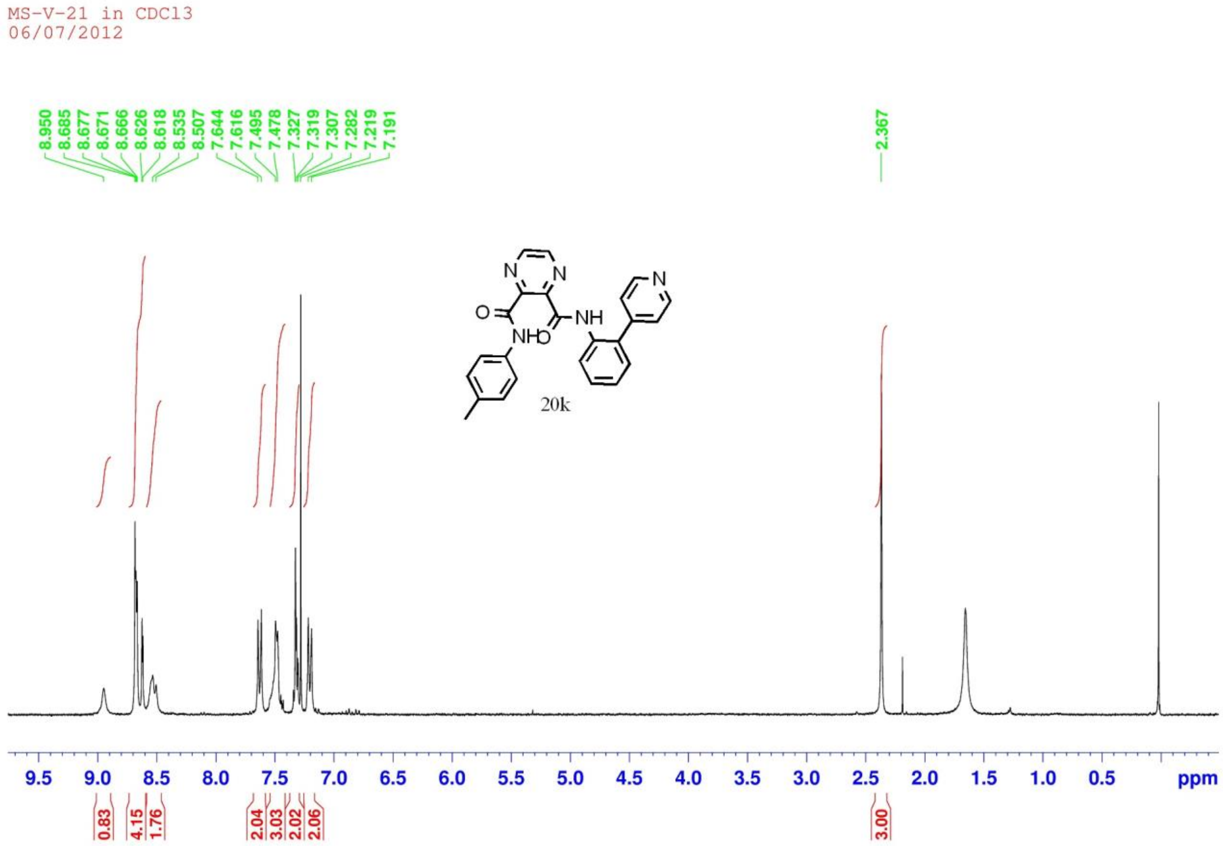

^{13}C NMR (75 MHz, $CDCl_3$)

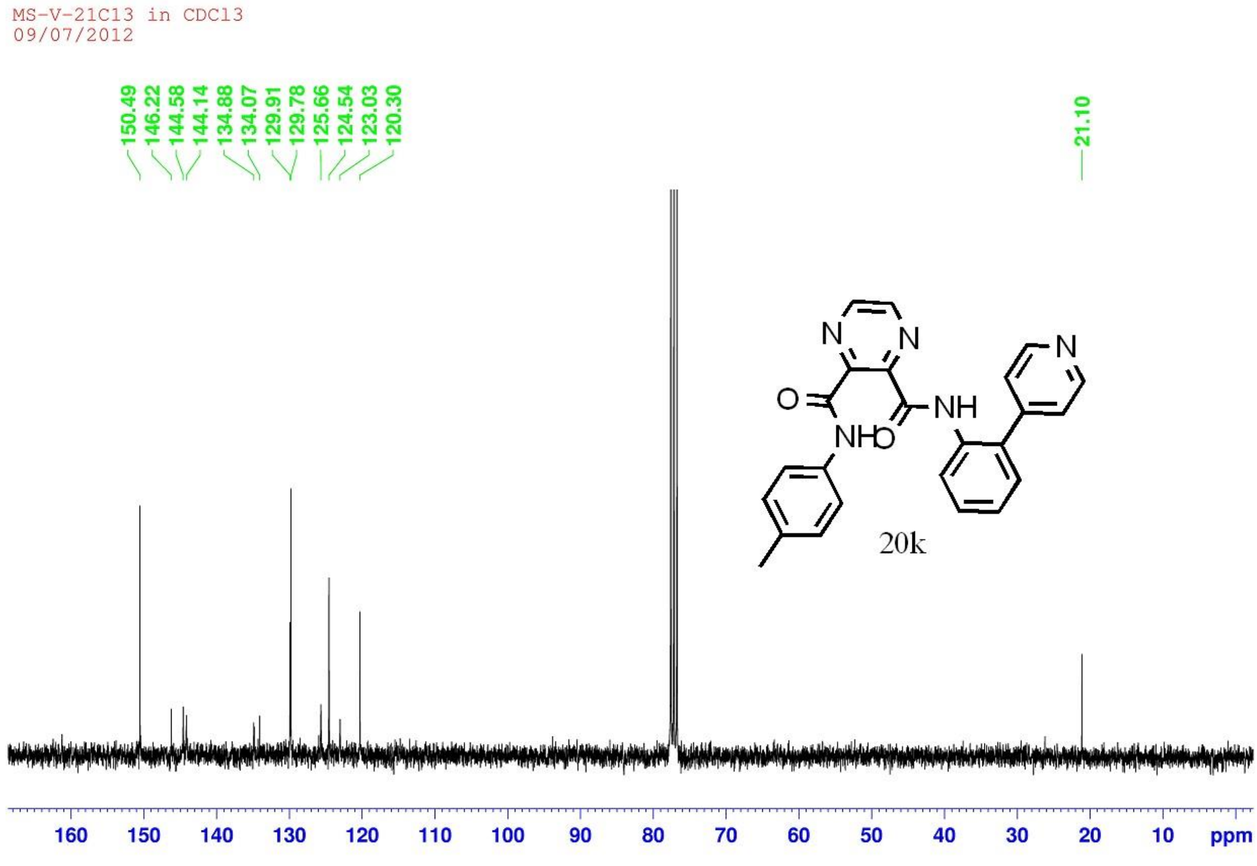

N^2-(2-(pyridin-3-yl) phenyl)-N^3-(p-tolyl) pyrazine-2,3-dicarboxamide (20l)

HRMS (ES+)

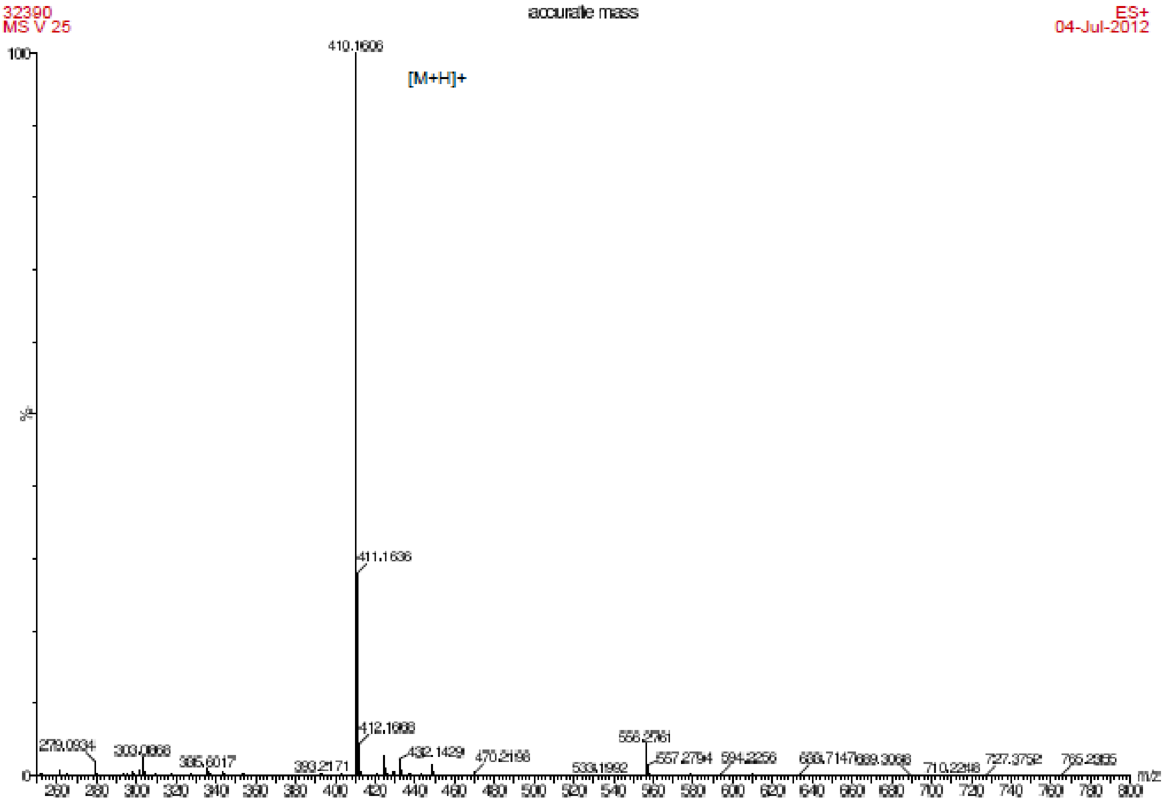

^1H NMR (300 MHz, $CDCl_3$)

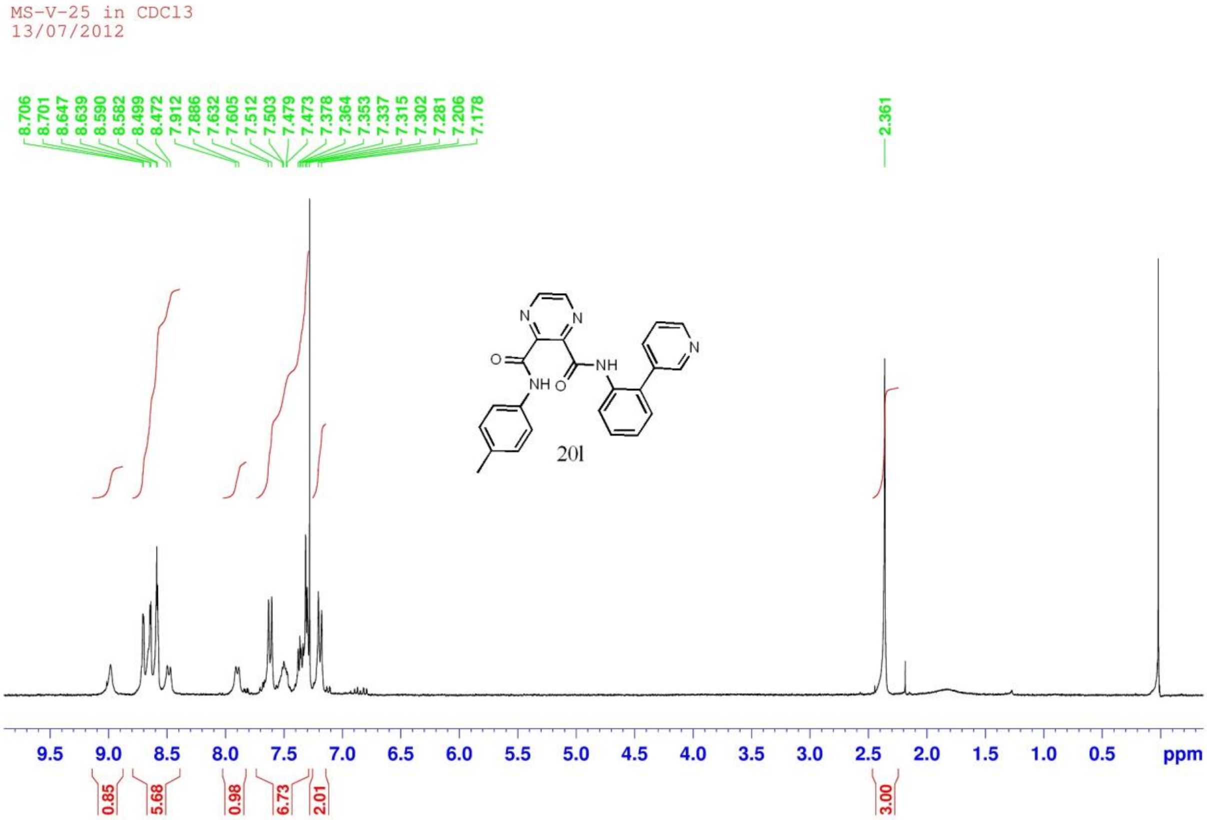

^{13}C NMR (75 MHz, $CDCl_3$)

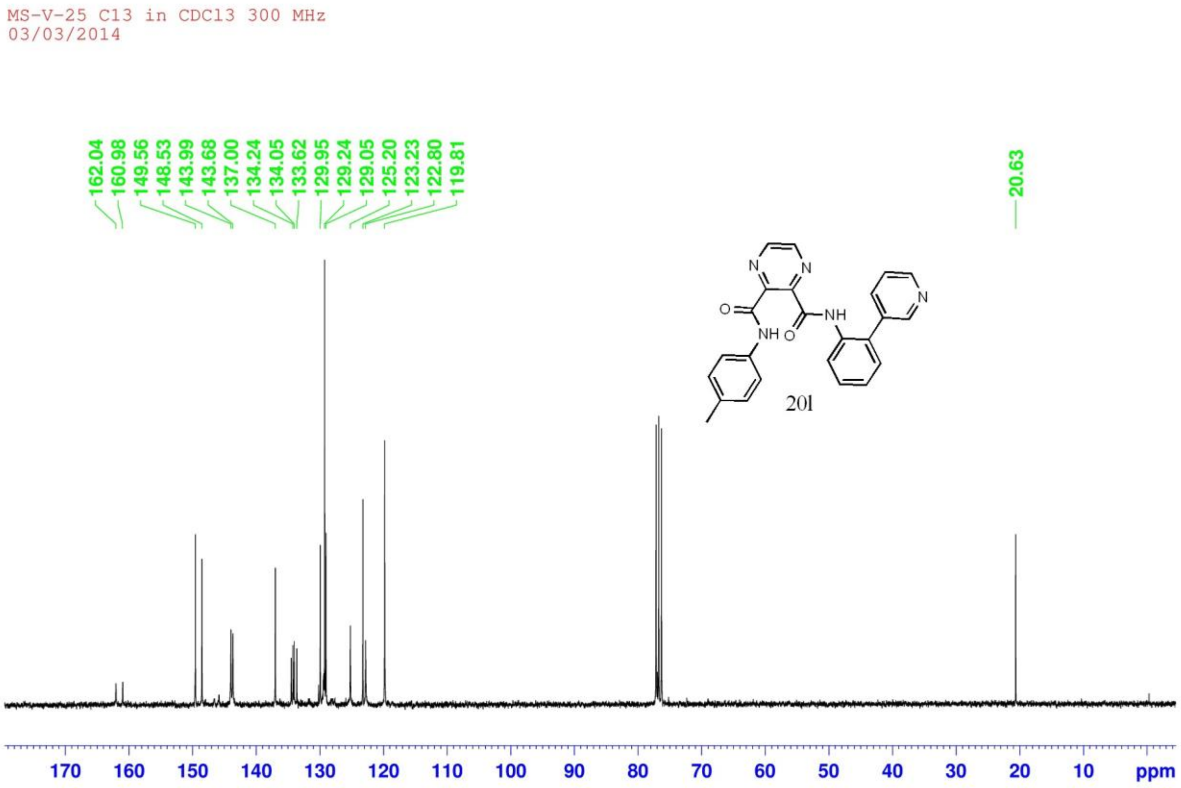

N^2-(4-(pyridin-4-yl) benzyl)-N^3-(p-tolyl) pyrazine-2,3-dicarboxamide (20m)

HRMS (ES+)

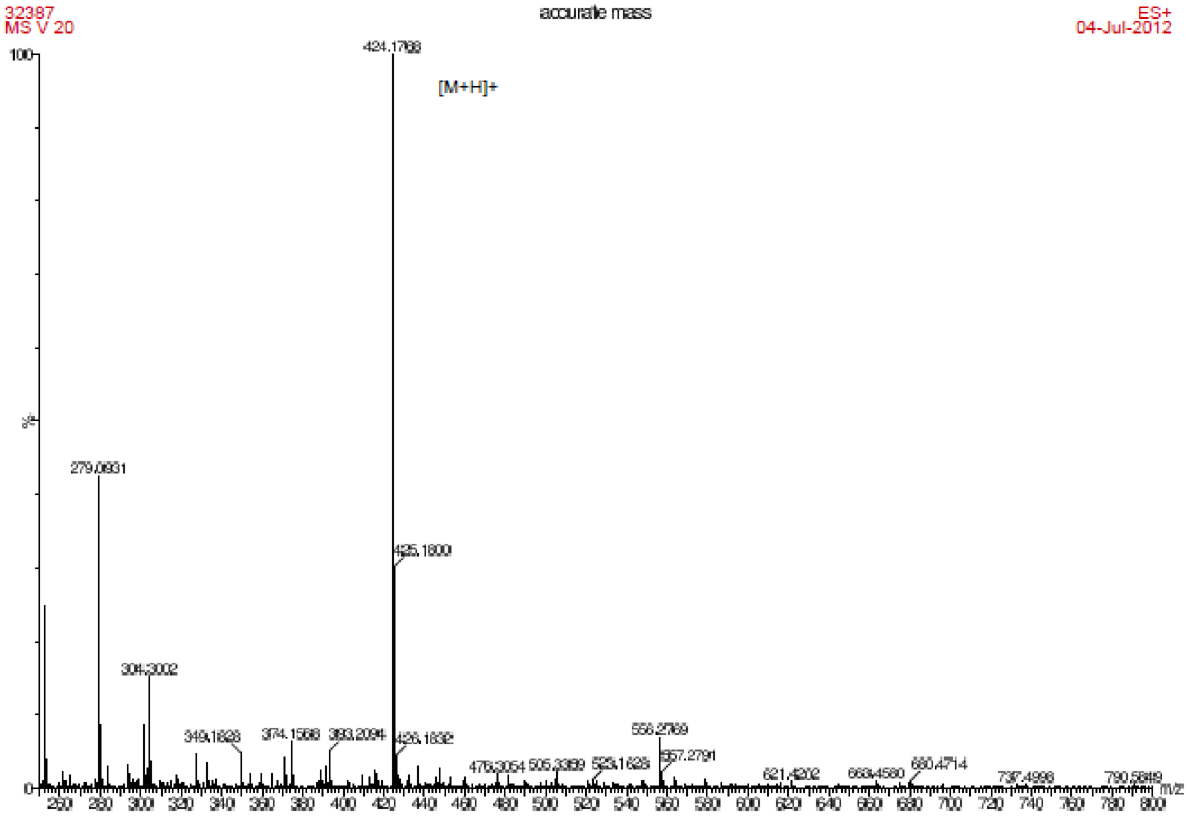

^{1}H NMR (300 MHz, $CDCl_3$)

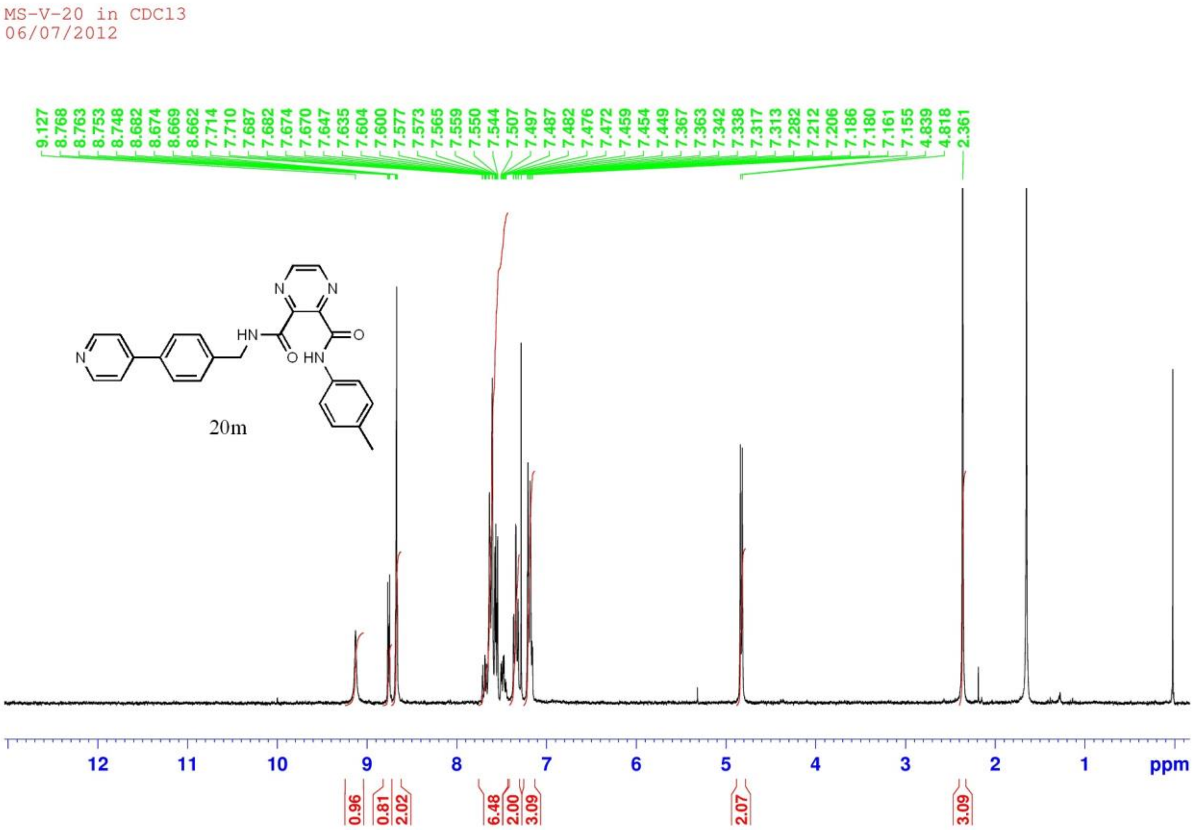

^{13}C NMR (75 MHz, $CDCl_3$)

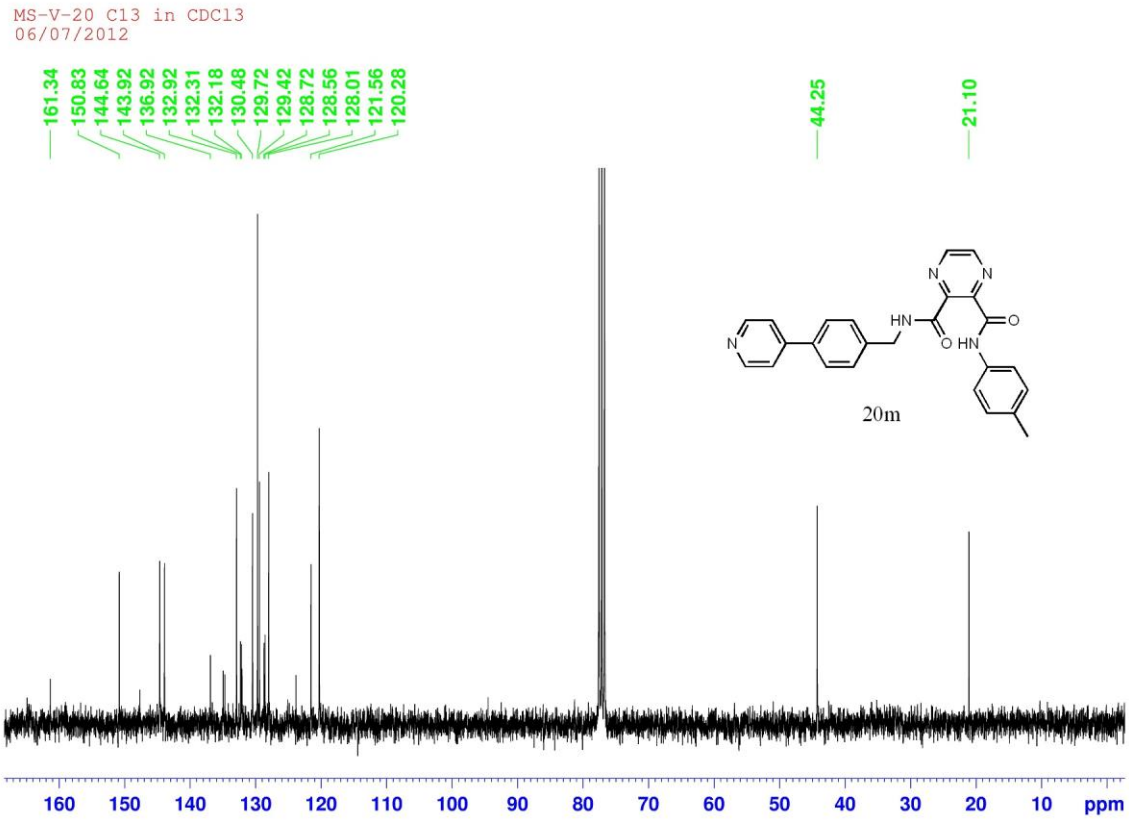

N^2-(4-methyl-2-(pyridin-4-yl) phenyl)-N^3-(p-tolyl) pyrazine-2,3-dicarboxamide (20n)

HRMS (ES+)

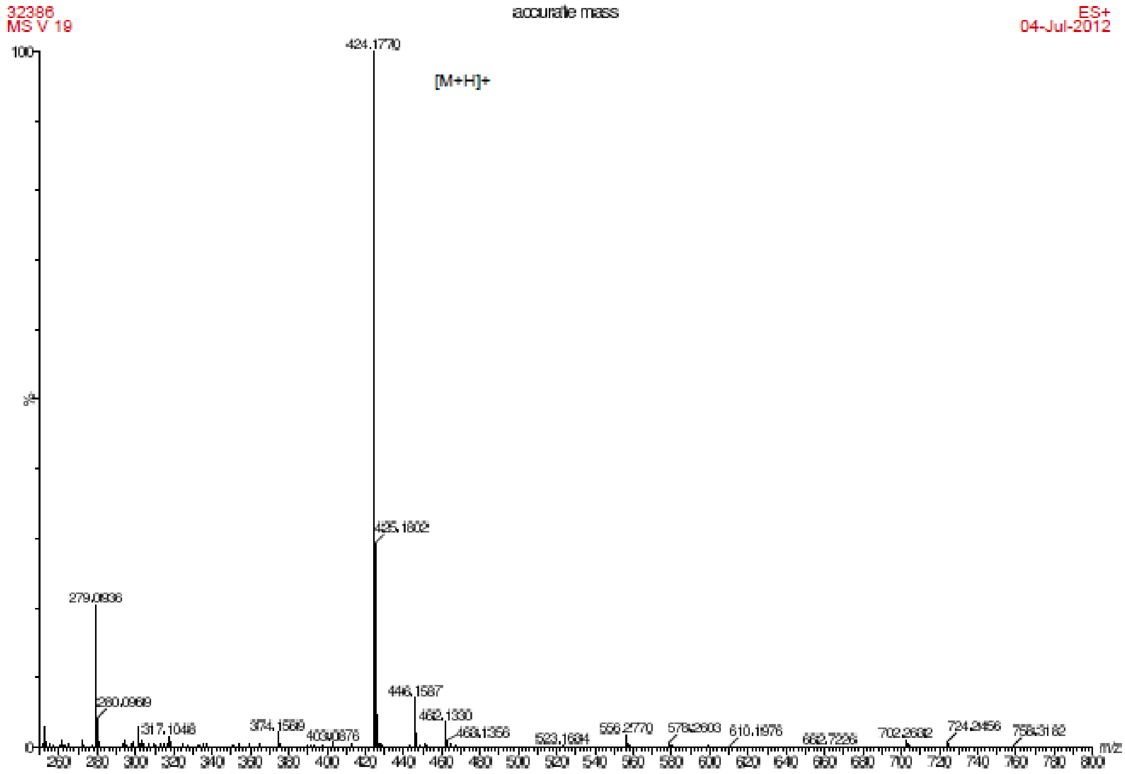

^{1}H NMR (300 MHz, $CDCl_3$)

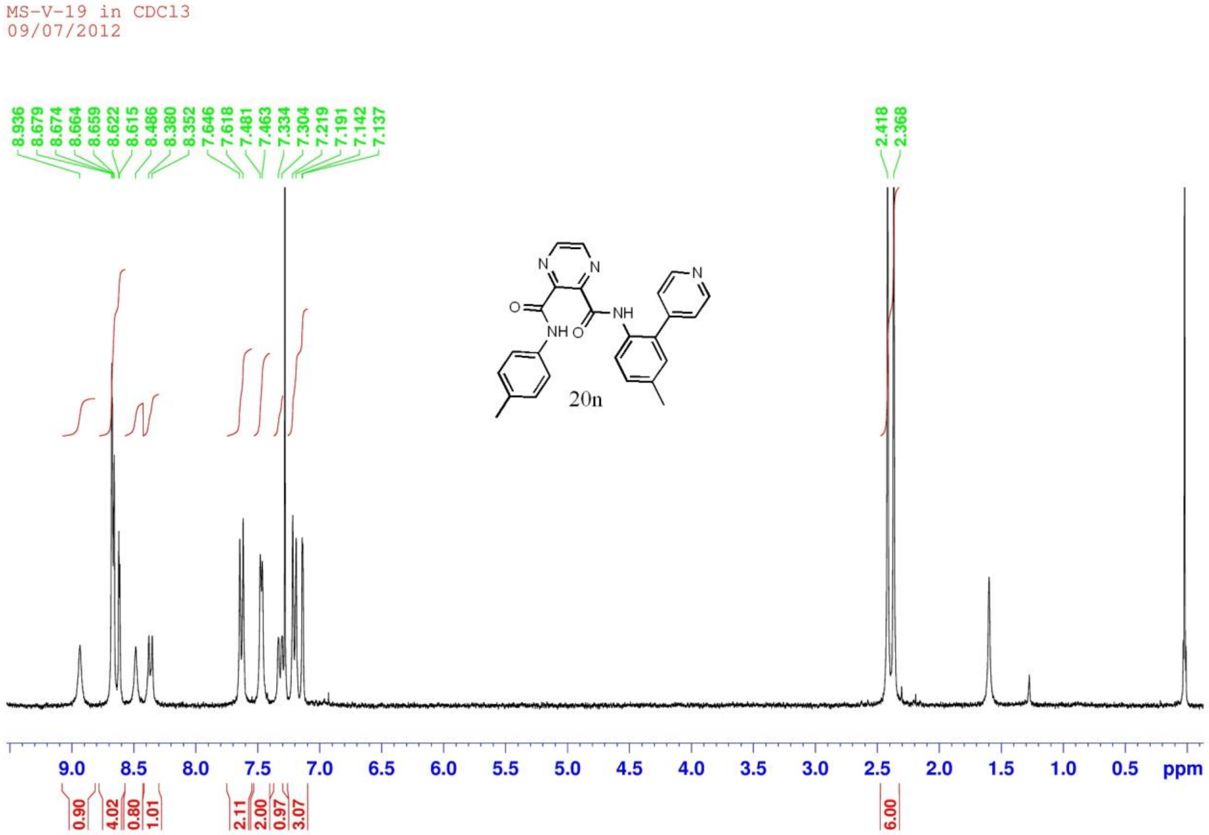

^{13}C NMR (75 MHz, $CDCl_3$)

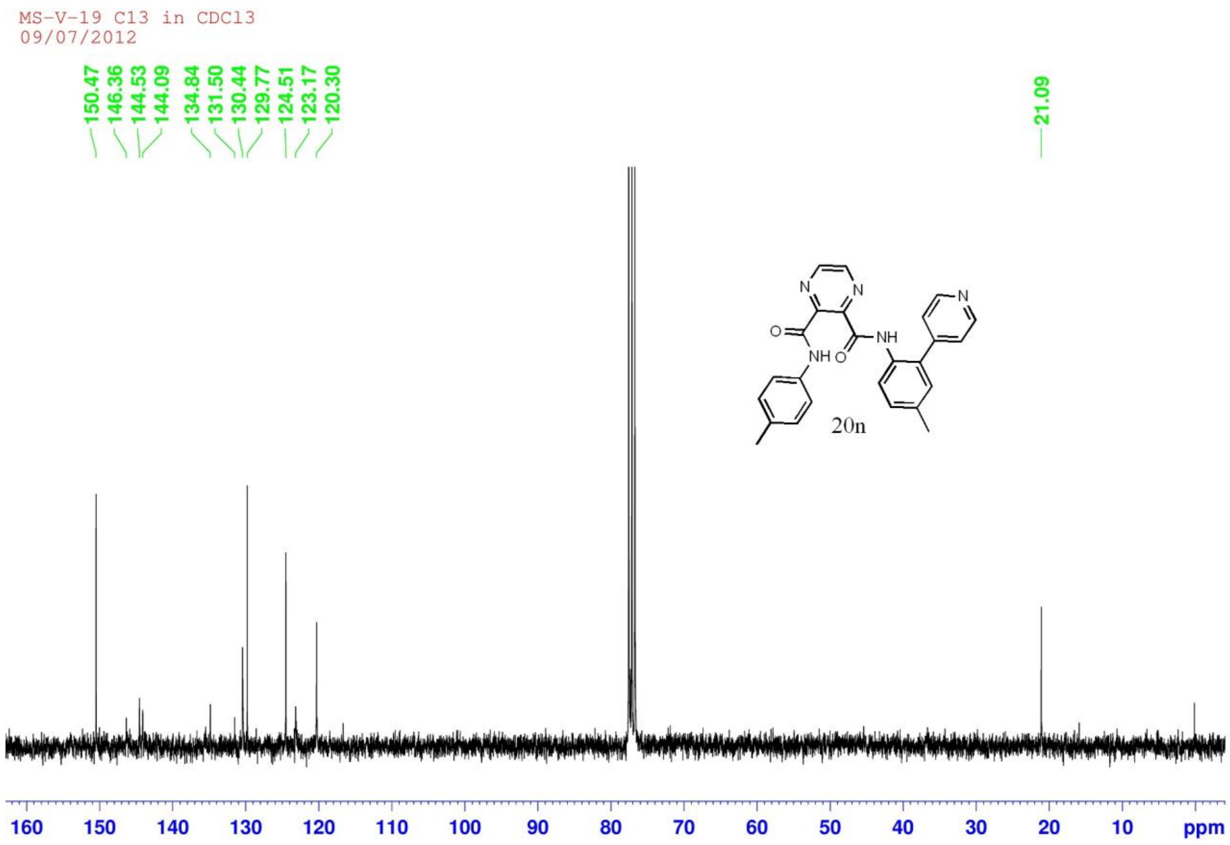

Supplement: Supplementary file 1 [file mmc1.pdf]
